# Ring-opening functionalizations of unstrained cyclic amines enabled by difluorocarbene transfer

**DOI:** 10.1038/s41467-020-18557-8

**Published:** 2020-09-21

**Authors:** Youyoung Kim, Joon Heo, Dongwook Kim, Sukbok Chang, Sangwon Seo

**Affiliations:** 1 Department of Chemistry, Korea Advanced Institute of Science and Technology (KAIST), Daejeon, 34141 Republic of Korea; 2 Center for Catalytic Hydrocarbon Functionalizations, Institute for Basic Science (IBS), Daejeon, 34141 Republic of Korea

**Keywords:** Synthetic chemistry methodology, Combinatorial libraries, Reaction mechanisms

## Abstract

Chemical synthesis based on the skeletal variation has been prolifically utilized as an attractive approach for modification of molecular properties. Given the ubiquity of unstrained cyclic amines, the ability to directly alter such motifs would grant an efficient platform to access unique chemical space. Here, we report a highly efficient and practical strategy that enables the selective ring-opening functionalization of unstrained cyclic amines. The use of difluorocarbene leads to a wide variety of multifaceted acyclic architectures, which can be further diversified to a range of distinctive homologative cyclic scaffolds. The virtue of this deconstructive strategy is demonstrated by successful modification of several natural products and pharmaceutical analogues.

## Abstract (web summary)

Cyclic amines are commonly present in natural products and synthetic compounds, but methods for their skeletal diversification are limited. Here, the authors report a strategy for selective ring-opening functionalization of unstrained cyclic amines to pluripotent products that can be further diversified.

## Introduction

**Fig. 1:**
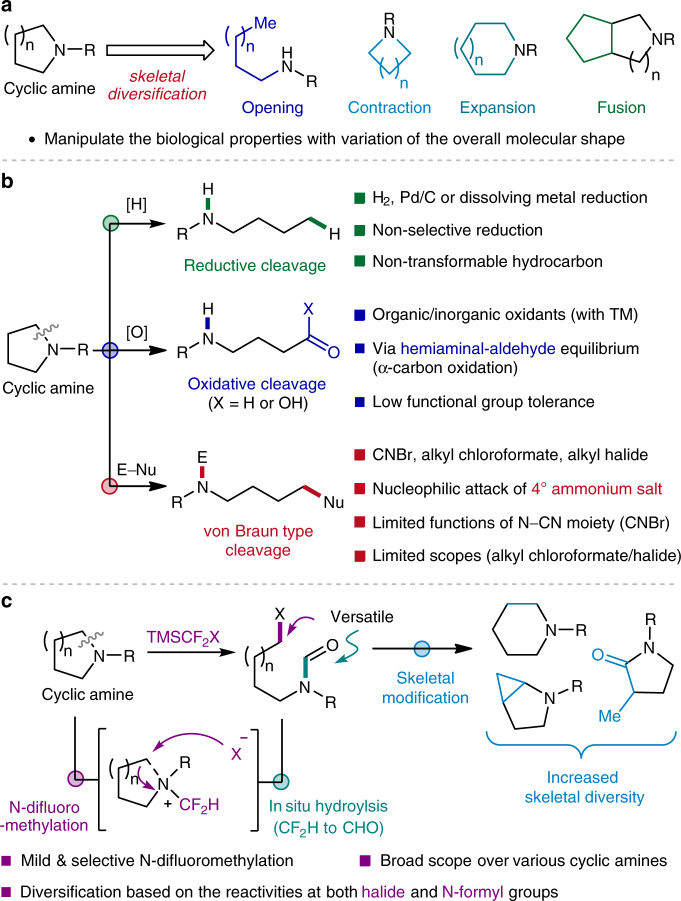
C–N Bond cleavage of unstrained cyclic amines. **a** Importance of skeletal diversification of unstrained cyclic amines. **b** Previously reported strategies for C–N bond cleavage of cyclic amines. **c** This work: Ring-opening halogenation via *N*-difluoromethylative C–N bond cleavage.

Given the ubiquity of cyclic amines in a myriad of natural products and synthetic compounds, a growing emphasis has been placed on how we harness and manipulate such prestigious motifs for the construction of value-added molecules^1–4^. While significant progress has been made in the development of appending processes for their peripheral variation^5–10^, the skeletal diversification by means of ring-opening, -contraction, -expansion or -fusion approaches remains limited (Fig. 1a)^11^. This is particularly pertinent for the unstrained cyclic systems, which is attributed to the fact that the structural reorganization is inevitably accompanied by highly demanding C–N^12,13^ or C–C^14–16^ bond cleavage. To this end, the ability to access these robust σ-bonds in an efficient and selective manner would provide a powerful handle for the diversification of azacyclic skeletons.

Despite recent advances in the realm of C–C bond transformations^17–20^, the selective cleavage of C–N bonds would be a compelling strategy for ring-opening functionalization of cyclic amines. Nonetheless, the process that cleaves and turns C–N bonds into versatile functionalities is still relatively scarce. For instance, the conventional reductive approach, typically employing molecular hydrogen in combination with transition metal catalysts, often leads to non-transformable hydrocarbon frameworks by reductive C–N bond cleavage, thus limiting the degree of scaffold diversity (Fig. 1b, top)^21–24^.

In an ideal scenario, the ring-opening reaction would be followed by the installation of versatile motifs that can be further transformed for diversity-generating chemical synthesis^25–27^. With this regard, the oxidative strategy has enjoyed methodological prosperity in recent years^28–32^. In a classical reaction mode, oxidation of the α-carbons of cyclic amines generates labile hemiaminals, thereby enabling the conversion of otherwise inert C–N bonds into amino-aldehyde or -carboxylic acid moieties (Fig. 1b, middle). While the conventional oxidative strategies often suffer from low functional group compatibility, improved procedures have been recently developed by harnessing transition metals. A conspicuous example includes the elegant work by Sarpong^33^, which furnishes a deconstructive halogenation of cyclic amines via cascade decarboxylative process.

An alternative way of rendering the C–N bonds more accessible is through the formation of quaternary ammonium salts (Fig. 1b, bottom). The increased bond polarity ameliorates the leaving group ability of amino moiety, ultimately allowing the C–N bond cleavage by means of elimination^34^ or nucleophilic substitution reactions^35–37^. This chemistry has been widely utilized in the ring-opening reaction of strained rings^38–42^, however, its use in unstrained cyclic systems is somewhat thwarted by the stability of the involved salts. Of a great potential for further development is the von Braun reaction, which employs cyanogen bromide (CNBr) in ring-opening bromination of azacyclic compounds^43,44^. This reaction outperforms other strategies in terms of efficiency and applicability, but has found relatively narrower synthetic applications^45–48^. This is mainly due to the limited function of the resulting N–CN group and the requirement of harsh conditions for its removal. Additionally, cyanogen bromide is highly toxic, and can undergo explosive trimerization to cyanuric bromide or decompose to hazardous hydrogen cyanide (HCN)^49^. Chloroformates have been utilized as milder reagents^50–54^, however generally applied for strained ring systems^38,41^, with an exceptional report by Cho still limited to *N*-alkyl-substituted 5-membered cyclic amines^55^. One of the main challenges associated with such reagents comes from a poor capability to access the key ammonium salt intermediates, which in turn restricts the employable ring types.

Here we report a highly efficient and practical strategy for the ring-opening functionalization of a wide variety of unstrained cyclic amines, including but not limited to 5- and 6-membered rings (Fig. 1c). The use of difluorocarbene allows a prominently selective *N*-difluoromethylation, with subsequent ring-opening halogenation and in situ hydrolysis furnishing acyclic *N*-formyl haloamines. The resulting pluripotent motifs further enable the construction of diverse arrays of homologated scaffolds by transforming both *N*-formyl and halide functional groups incorporated in products, providing a unique platform for scaffold diversity that cannot be accessed by the conventional von Braun-type reactions. Not only a wider range of cyclic amines can be deconstructively modified, but this strategy can also be applied to complex molecules bearing labile functional groups.

## Results

### Reaction development

Difluorocarbene serves as an excellent reagent in transition metal-free cyclopropanation of alkenes and alkynes, and can also react with various carbon or heteroatom nucleophiles^56,57^. We envisaged that its highly-electrophilic character would permit an efficient and selective generation of key ammonium salt intermediates from a variety of azacyclic cores, whereby the introduced difluoromethyl (CF_2_H) group would then manipulate the ring properties to eventually enable the deconstructive functionalization of challenging C–N bonds. Difluorocarbene can be readily generated by *α*-elimination of CF_2_XY-type reagents, among which (bromodifluoromethyl)trimethylsilane (TMSCF_2_Br) stands out as one of the most practical precursors^58^. It is non-toxic, air- and moisture-stable, easy to handle, and can generate the reactive species under mild conditions.

**Fig. 2:**
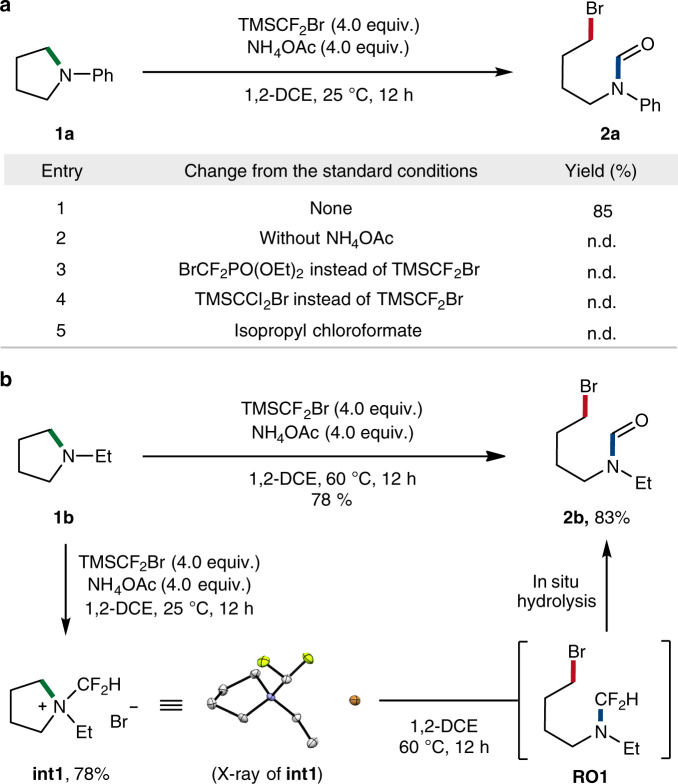
Reaction development. **a** Selected optimization conditions for the ring-opening bromoformylation of *N*-phenylpyrrolidine **1a**; All reactions were performed on a 0.2 mmol scale; 1,2-DCE, 1,2-dichloroethane; n.d., not detected; Isolated yields. **b** Isolation of an ammonium salt intermediate from *N*-ethylpyrrolidine **1b**.

We, therefore, focused on the use of TMSCF_2_Br in our initial investigation towards the deconstructive functionalization of *N*-phenylpyrrolidine **1a**. Pleasingly, after extensive screenings of various reaction parameters, we identified the optimized conditions consisting of TMSCF_2_Br, NH_4_OAc base and 1,2-dichloroethane (1,2-DCE) solvent, which furnished the ring-opening product **2a** in 85% yield at room temperature in 12 h (Fig. 2a, entry 1). Interestingly, the deconstructive product was incorporated with an *N*-formyl moiety, presumably by in situ hydrolysis of the unstable *N*-CF_2_H group^59^. This cascade process is beneficial in synthetic aspects, as the *N*-formyl group not only is readily removable^60^, but also provides a unique opportunity for diverse post-modifications. Control experiments established the importance of base (entry 2), which is required for the generation of difluorocarbene intermediate. Notably, an alternative difluorocarbene source (entry 3), a dichlorocarbene variant^61^ (entry 4) and the conditions developed by Cho^55^ (entry 5) were found to be incompetent (see Supplementary Table 1 for details). Encouraged by the above promising result, we also examined the feasibility of *N*-ethylpyrrolidine **1b** in the ring-opening bromoformylation (Fig. 2b). We could obtain the presupposed intermediacy of quaternary ammonium salt by treating **1b** under the optimized conditions at room temperature (**int1**, 78%). A moderate heating of the isolated intermediate **int1** consequently resulted in a facile ring-cleavage to afford **2b** (83%).

### Substrate scope of 5-membered rings

**Fig. 3:**
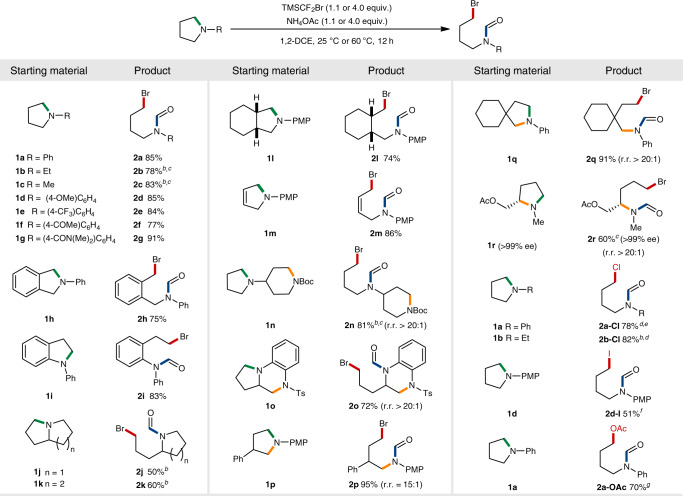
Reaction scope of five-membered cyclic amines. ^*a*^Reaction conditions: Unless otherwise indicated, all reactions were performed on a 0.2 mmol scale using cyclic amine (1.0 equiv.), TMSCF_2_Br (4.0 equiv.), NH_4_OAc (4.0 equiv.) and 1,2-DCE (0.5 mL) at room temperature. regioselectivity ratios (r.r.) were determined by ^1^H NMR spectroscopy of crude mixtures. ^*b*^60 °C. ^*c*^1.1 equiv. of TMSCF_2_Br and 1.1 equiv. of NH_4_OAc. ^*d*^TMSCF_2_Cl (4.0 equiv.) instead of TMSCF_2_Br. ^*e*^KF (4.0 equiv.) as an alternative base, with H_2_O (0.05 mL) as a co-solvent. ^*f*^TMSCF_2_I (4.0 equiv.) instead of TMSCF_2_Br. ^*g*^4.0 equiv. of TMSCF_2_Cl and 16.0 equiv. of NH_4_OAc. PMP *para*-methoxyphenyl.

Having established the optimal conditions, we next evaluated the substrate scope over a range of 5-membered azacyclic compounds (Fig. 3). All substrates examined were smoothly reacted under mild conditions irrespective of the nature of *N*-substituents, furnishing the ring-opening bromoformylation products in excellent selectivity and high efficiency (**2a–2r**). Noteworthy is that 1.1 equiv. of the difluorocarbene precursor was sufficient enough for *N*-alkyl substrates (**2b–2c**). Methoxy, trifluoromethyl, ketone and amide functionalities on *N*-aryl groups were found to be well-tolerated in ring-opening of pyrrolidines (**2d**–**2g**, 77–91%). Benzo-fused and fully saturated bicyclic amines were also effectively transformed (**2h**–**2l**, 50–83%). In case of octahydroindolizine (**1k**), the skeleton was ring-opened exclusively at the 5-membered unit. A dihydropyrrole **1m** was readily ring-opened without affecting the olefin moiety (**2m**, 86%), proving its orthogonality to the previously reported cyclopropanation reactions^58^.

Significantly, reactivity differentiation by basicity of amines or steric influences allows the reaction to occur in excellent regioselectivities. For instance, when there are two nitrogen atoms present in the same or different azacyclic skeletons, those with a more basic amine were exclusively difluoromethylated and subsequently ring-opened (**2n** and **2o**, 81 and 72%). For 2- or 3-substituted pyrrolidines, the ring was selectively cleaved at the sterically less demanding 5-position (**2p**–**2r**, 60–95%). We were delighted to find that the variation of halides was also possible, affording the chlorination or iodination products in moderate to good yields (**2a-Cl**, **2b-Cl** and **2d-I**, 51–82%). In addition, the ring-opening acetoxylation was achieved (**2a-OAc**, 70%) by employing an excessive amount of NH_4_OAc in combination with (chlorodifluoromethyl)trimethylsilane (TMSCF_2_Cl).

### Elucidation of the regioselectivity in C–N bond cleavage

**Fig. 4:**
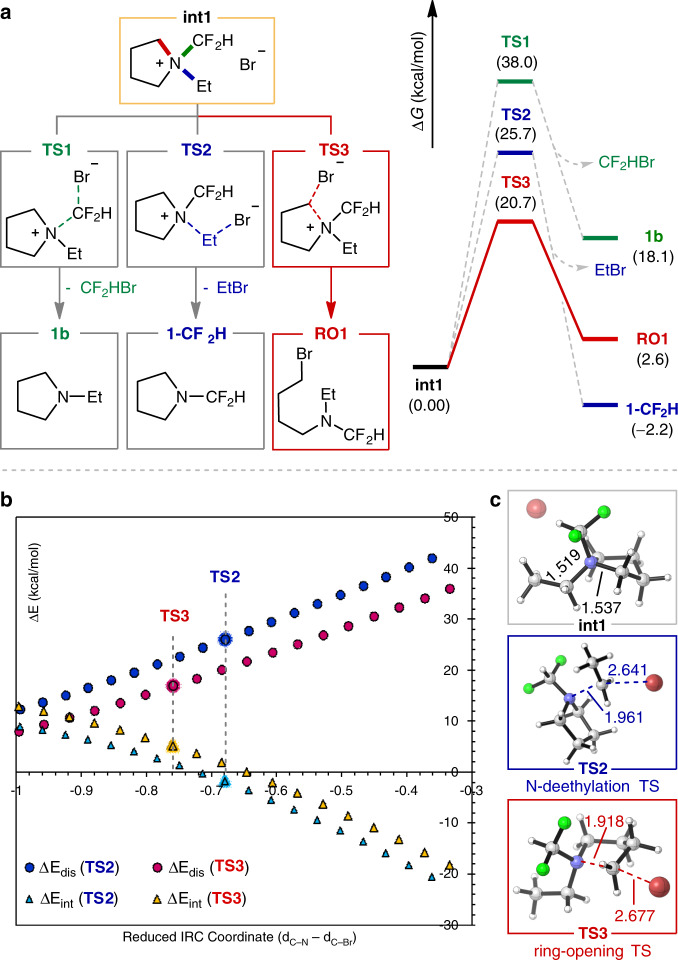
Evaluation of selectivity for 5-membered cyclic amines with DFT calculation. **a** Reaction energy profiles of three possible nucleophilic substitution pathways from *N*-CF_2_H salt (**int1**); green: dedifluoromethylation path leading to **1b** (via TS1), blue: deethylation path leading to **1-CF**_**2**_**H** (via TS2), and red: ring-opening path leading to **RO1** (via TS3). **b** Distortion-interaction analysis along IRC coordinate of **int1**; blue dot: distortion energy of deethylation, red dot: distortion energy of ring-opening, sky blue triangle: interaction energy of deethylation, and yellow triangle: interaction energy of ring-opening. **c** DFT-optimized structures of **int1** and its transition states for deethylation (blue, TS2) and ring-opening (red, TS3).

The finding of a convenient method for ring-opening functionalization of 5-membered cyclic amines led us to probe the origin of selectivity for *N*-alkyl substrates (Fig. 4). Taking a CF_2_H salt **int1** as a model intermediate, three potential substitution pathways were computationally evaluated (Fig. 4a). Transition state analysis was consistent with the observed selectivity for **int1**. The ring-opening process (**TS3**) displayed an activation barrier of 20.7 kcal/mol, whereas those for deethylation (**TS2**) and dedifluoromethylation (**TS1**) were higher in energy by 5 and 17 kcal/mol, respectively. Although the product from the ring-opening pathway (**RO1**) was slightly uphill by 2.6 kcal/mol, this process was expected to be facilitated by the subsequent hydrolysis of the *N*-CF_2_H group to the thermodynamically stable *N*-formyl product **2b** (see Supplementary Fig. 3). The revealed preference of **int1** to undergo the ring-opening pathway (**TS3**) over *N*-deethylation (**TS2**) could be quantified by a distortion-interaction analysis (Fig. 4b). Along with the intrinsic reaction coordinate, distortion energy term of ring-opening is significantly smaller than that of *N*-deethylation, whereas their interaction energy profiles are rather similar. From these outcomes, it could be concluded that the earlier nature of the ring-opening transition state with respect to the C–N breaking event is responsible for the corresponding selectivity. Moreover, the DFT-optimized structures of **int1** clearly show that the cyclic C–N bond is noticeably longer than the ethyl C–N bond presumably because of the ring strain, indicating that the attack of the cyclic C–N bond involves the least structural reorganization towards the corresponding transition state (Fig. 4c).

### Extension to 6-membered cyclic amines

**Fig. 5:**
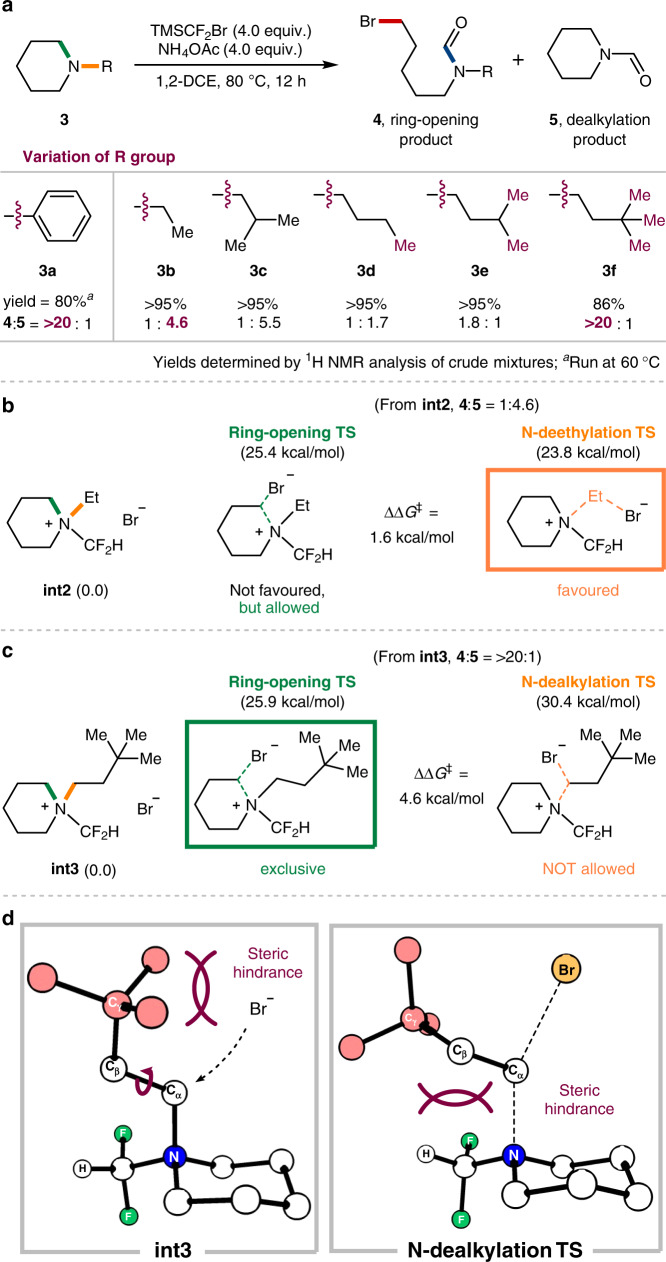
Application to 6-membered cyclic amines and DFT calculations for the elucidation of the observed selectivity. **a** Influence of R group on the site-selectivity in 6-membered rings. **b** Competitive reaction energy profiles of *N*-dealkylation and ring-opening pathways of *N*-ethyl salt **int2**. **c** Competitive reaction energy profiles of *N*-neohexyl salt **int3**. **d** Structural analysis for *N*-dealkylation of **int3**.

With the comprehensive results obtained for the ring-opening functionalization of five-membered cyclic amines, we wondered whether the current methodology could also be applied to 6-membered cyclic amines. Delightfully, the initial attempt with *N*-phenylpiperidine **3a** as a substrate gave a ring cleavage product **4a** under the optimized conditions at 60 °C (80%, Fig. 5a). On the other hand, *N*-alkyl substrates were obstructed by undesired N-dealkylation, which has been a major problem encountered in most of the existing ring-opening strategies^31^. For instance, *N*-ethylpiperidine **3b** afforded the ring-opening product **4b** in 18% yield along with the *N*-dealkylation product **5** in 82% yield (**4b**:**5**, 1:4.6). In addition to the earlier example of octahydroindolizine (**1k**, Fig. 3), these results suggested again that 6-membered rings are less influenced by the presence of a CF_2_H group, presumably due to their inherent ring stability. Intriguingly, we discovered that this issue could be overcome by examining the steric influence of the *N*-alkyl group. In particular, the ring-opening pathway becomes substantially more favourable with increment of substituents at the *γ*-position (**4d**–**4f**). For example, *N*-dealkylation pathway was largely suppressed in the case of N-neohexyl substrate (**3f**), which exclusively gave the ring-opening product **4f** in 86% yield. Meanwhile, *N*-isobutyl substrate (**3c**) showed a similar selectivity to *N*-ethyl substrate (**3b**), indicating that a *β*-substituent has a negligible effect on the selectivity. These outcomes are captivating as the observed trend is opposed to that of general S_N_2 reactions, in which branching carbons farther away from the electrophilic site tend to have a smaller effect on the nucleophilic attack^62^.

Having identified this interesting steric influence, we further sought to investigate the observed selectivity by computational analysis. *N*-Difluoromethyl-*N*-ethyl piperidinium bromide (**int2**) and the analogous *N*-neohexyl salt (**int3**) were representatively chosen for the case study. Consistent with the experimental results, the DFT calculations on the *N*-ethyl salt **int2** show that *N*-dealkylation is marginally preferred over the ring-opening pathway (∆∆*G*^‡^ = 1.6 kcal/mol, Fig. 5b). In sharp contrast, the activation barrier for ring-opening of **int3** is 4.6 kcal/mol lower in energy than the corresponding *N*-dealkylation (Fig. 5c). The structural analysis of the intermediates and their transition states further revealed the primary steric influence by *γ*-substituents (Fig. 5d). The DFT-optimized structure of the *N*-neohexyl salt **int3** clearly shows that the substituent at the *γ*-position of *N*-alkyl group considerably disturbs the N-dealkylation path. As a result, the rotation along the C(*α*)–C(*β*) bond becomes imperative as the nucleophile approaches in the transition state, which then causes an unfavourable steric repulsion with the N–CF_2_H piperidine moiety. Consequently, *N*-dealkylation becomes disfavoured with a significantly increased activation barrier, rendering the ring-opening pathway relatively more favourable.

### Substrate scope of 6-membered and larger rings

**Fig. 6:**
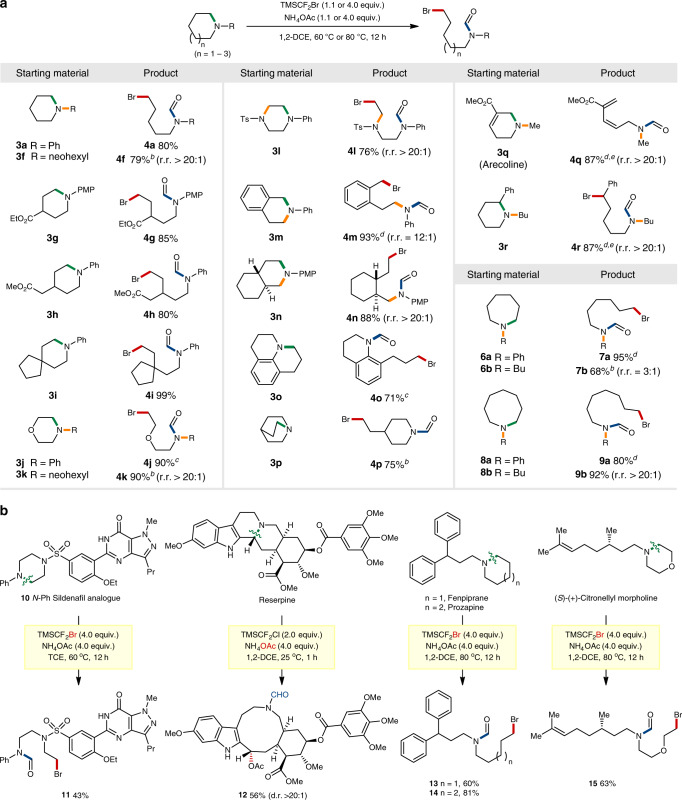
Reaction of six-membered and larger cyclic amines. **a** Substrate scope; ^*a*^reaction conditions: unless otherwise indicated, all reactions were performed on a 0.2 mmol scale using cyclic amine (1.0 equiv.), TMSCF_2_Br (4.0 equiv.), NH_4_OAc (4.0 equiv.) and 1,2-DCE (0.5 mL) at 60 °C. regioselectivity ratios (r.r.) were determined by ^1^H NMR spectroscopy of crude mixtures. ^*b*^80 °C. ^*c*^40 °C. ^*d*^25 °C. ^*e*^1.1 equiv. of TMSCF_2_Br and 1.1 equiv. of NH_4_OAc. PMP *para*-methoxyphenyl. **b** Late-stage ring-opening functionalization of biologically relevant compounds.

With the obtained understanding of reactivity and selectivity, a range of 6-membered cyclic amines were explored for the current ring-opening functionalization strategy (Fig. 6a). Piperidine rings were viable to give the desired products in good to excellent yields (**4a**–**4i**, 79–99%), and both *N*-aryl and *N*-alkyl substituents could be incorporated. Ester functional groups were found to be tolerable, with the 4-substituted piperidines (**3g** and **3h)** giving rise to *γ*- and *δ*-amino esters **4g** and **4h** in 85 and 80% yields, respectively. A spiro bicyclic substrate (**3i**) was also effectively functionalized to give the corresponding product **4i** in 99% yield.

Morpholine was also feasible with both *N*-phenyl (**3j**) and *N*-neohexyl (**3k**) substituents, affording the valuable acyclic products **4j** and **4k** in excellent yields (90%). 1-Phenyl-4-tosylpiperazine (**3l**) was selectively ring-opened at the 2-position (**4l**, 76%, r.r. > 20:1), which was enabled by a selective difluoromethylation at the nitrogen incorporated with a phenyl group. Tetrahydroisoquinoline substrate (**3m**) was reacted at the more electrophilic benzylic site to give the ring-opened product in an excellent yield and high selectivity (**4m**, 93%, r.r. = 12:1). The reaction of *trans*-decahydroisoquinoline (**3n**) was also highly selective, leading to the deconstructive product by bromination at the sterically less demanding site (**4n**, 88%, r.r. > 20:1). Moreover, julolidine (**3o**) and quinuclidine (**3p**) cyclic cores were effectively transformed to the corresponding bromoamines (**4o** and **4p**, 71 and 75%, respectively).

Notably, arecoline **3q** exclusively gave the ring-opening product **4q** (87%) by a Hofmann-type elimination reaction. This relatively simple natural product had previously been reported to favour *N*-demethylation when treated with chloroformates^51^. The reaction of an *N*-butylpiperidine substrate **3r** occurred exclusively at the position appended with a phenyl group (**4r**, 87%). These two examples showed that steric at the *N*-alkyl group is not necessary in cases where the ring is substituted with certain functional groups. Furthermore, the reaction was also found feasible with 7- and 8-membered rings, furnishing the desired linear bromoamine products in excellent yields (**7a**–**7b** and **9a**–**9b**, 68–95%). Overall, the ability to manipulate various cyclic skeletons allowed the synthesis of difficult-to-access acyclic bromoamine motifs with a wide range of *N*-substituents, including those previously underexplored for 6-membered or larger cyclic systems.

To further highlight the broad applicability of our method, late-stage modifications of biologically relevant compounds were examined (Fig. 6b). The exceptional functional group tolerance and excellent regioselectivity were proven with a Sildenafil analogue **10** that contains various nitrogen atoms, which afforded **11** by an exclusive ring-opening of the piperazine scaffold in 43% yield along mostly with unreacted starting material. Reserpine also underwent an efficient C–N bond cleavage by acetoxylation at the benzylic site, leading to the synthetically challenging 10-membered azacyclic product **12** (56%, >20:1 d.r.).

Notably, Fenpiprane and Prozapine, analogous drugs correspondingly containing *N*-alkyl substituted 6- and 7-membered cyclic amines, selectively afforded the ring-opening products **13** and **14** in 60 and 81% yields, respectively. Likewise, (*S*)-(+)-citronellyl morpholine was effectively ring-opened to give **15** in 63% yield. Considering that the basic motifs of these examined compounds are vastly present in many other pharmaceuticals and natural products, this approach is expected to find wide synthetic applications.

### Skeletal diversification

In addition to the excellent substrate generality and applicability demonstrated above, we envisioned that our resulting product could be further used as a pluripotent intermediate for skeletal diversification. The substrate-based strategy relying on the pre-installation of an internal functional group would limit the obtainable scaffolds from a specific ring system. With this regard, the ability to forge various ring structures through a common ring-opening approach would add a new dimension in generating structural-diversity. Taking advantages of the simultaneously introduced alkyl halide and *N*-formyl groups in our products, we anticipated that a series of branching reactions would grant distinct skeletons.

**Fig. 7:**
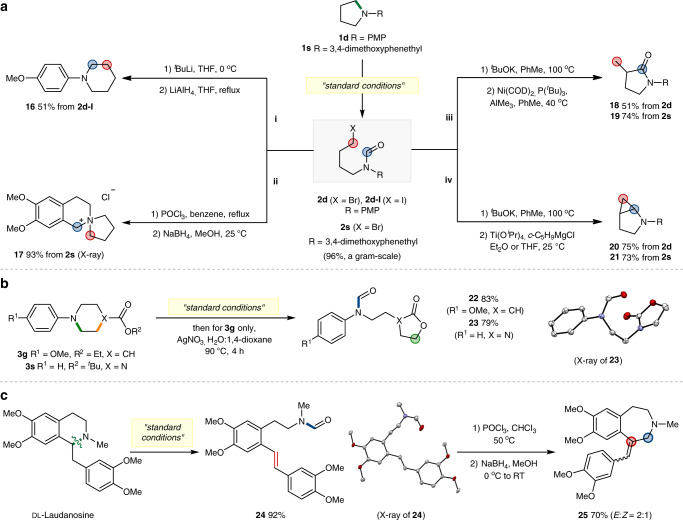
Skeletal diversification. **a** Skeletal remodelling of pyrrolidine rings. i, ring-expansion. ii, synthesis of spiro-bicyclic compound through the Bischler–Napieralski reaction. iii, synthesis of *γ*-lactam products by hydrocarbamoylation of E2 products. iv, synthesis of ring-fused products by the Kulinkovich–de Meijere reaction. **b** Skeletal remodelling of 6-membered rings by using a pre-installed nucleophile. **c** Ring-diversification of natural product: Ring-expansion of DL-Laudanosine.

First, one-pot sequence of lithium-iodine exchange, cyclization and reduction of the iodoformylation product **2d**–**I** gave a ring-expansion product **16** in 51% yield (Fig. 7a, i). This ring-opening-reclosing strategy is attractive in that it utilizes an external C1 source to achieve a homologation process that does not rely on the pre-encoded systems^63^. In the second ring-modification, 1-(3,4-dimethoxyphenethyl)pyrrolidine **1s** was ring-opened (**2s**, 96%, a gram-scale) and subsequently treated under the Bischler–Napieralski reaction conditions to afford a C1-tethered product **17** in 93% yield (Fig. 7a, ii).

The conversion of the bromoalkyl group to olefin functionality via E2 elimination, and succeeding intramolecular reaction with the *N*-formyl moiety offers another opportunity to obtain post-modification derivatives of high synthetic values. For example, Ni-catalysed hydrocarbamolylation^64^ of the newly generated double bond provided *γ*-lactam products **18** and **19** in 51 and 74% yields, respectively (Fig. 7a, iii). In addition, Ti-mediated intramolecular cyclopropanation using the Kulinkovich–de Meijere conditions^65^ gave 2-azabicyclo[3.1.0]hexane products **20** and **21** in 75 and 73% yields from **2d** and **2s**, respectively (Fig. 7a, iv).

We next briefly examined skeletal remodelling based on the use of pre-installed functional groups (Fig. 7b). 4-Carboxylate substituted piperidine **3g** was smoothly converted to a lactone product **22** (83%) by a one-pot ring-opening, hydrolysis and cyclization procedure. Similarly, the reaction of *N*-Boc protected piperazine **3s** afforded a cyclic carbamate product **23** (79%) under the standard ring-opening conditions, in this case, without the need of additional reagents. Moreover, the structure of obtained product **23** was characterized by its X-ray structure to confirm the site selectivity.

Furthermore, we demonstrated that our strategy could be utilized in ring-diversification of natural products. (Fig. 7c). For example, dl-Laudanosine was readily ring-opened by an elimination process to give the olefin intermediate **24** (92%), and the subsequent Bischler–Napieralski reaction and hydride insertion led to a 7-membered analogue **25** in 70% yield. In addition to the excellent efficiency and selectivity achieved with biologically relevant compounds, this unique ability to create a diversified scaffold represents one of the distinguishing features of our current methodology.

In summary, we have developed an efficient strategy for deconstructive modification of unstrained azacyclic systems. The key finding was the use of difluorocarbene as a mild and practical source to form the key ammonium salt intermediates, which not only surpass the existing methodologies in terms of practicability, efficiency, functional group tolerance, chemo- and regioselecitvities, but also provides a unique tool to diversify a wider range of cyclic amines to acyclic analogues and homologated cyclic cores upon post-transformations. The utility of the developed protocol was further demonstrated by the direct structural alteration of azacyclic cores present within complex molecules. This late-stage diversification of natural products and pharmaceutical analogues enabled ready access to underexplored chemical space around bioactive molecules.

## Methods

### General procedure for the ring-opening bromoformylation

To a 2 mL reaction vial containing a mixture of *N*-phenylpyrrolidine (29 μL, 0.2 mmol) and NH_4_OAc (61.7 mg, 0.8 mmol) in 1,2-dichloroethane (1,2-DCE) (0.5 mL) was added (bromodifluoromethyl)trimethylsilane (124 μL, 0.8 mmol). The vial was sealed and stirred at room temperature for 12 h, then filtered through a pad of celite and washed with CH_2_Cl_2_ (5 × 3 mL). The filtrate was concentrated in vacuo, and the desired product was obtained as a colourless oil (43 mg, 85%) after purification by flash chromatography (eluent: EtOAc/*n*-Hexane, 1:5).

## Supplementary information

Supplementary Information

## Data Availability

All data generated and analysed during this study are included in this article and its Supplementary Information, and also available from the authors upon reasonable request. The X-ray crystallographic coordinates for structures reported in this article have been deposited at the Cambridge Crystallographic Data Centre (CCDC), under deposition numbers CCDC 1977901 (**int1**), 1977902 (**23**), 1977903 (**17**), 1977905 (**24**), 2000476 (**int2**) and 2000477 (**int3**). These data can be obtained free of charge via https://www.ccdc.cam.ac.uk/structures/.
