# Supplementary material for: Ring-opening functionalizations of unstrained cyclic amines enabled by difluorocarbene transfer
Source: Nat Commun. 2020 Sep 21;11:4761. doi: 10.1038/s41467-020-18557-8 (PMC7506026; doi:10.1038/s41467-020-18557-8)
Supplement: Supplementary file 1 — Supplementary Information [file 41467_2020_18557_MOESM1_ESM.pdf]

Supplementary Information

Ring-opening functionalizations of unstrained cyclic amines enabled by difluorocarbene transfer

Kim et al.

Supplementary Methods

General procedures

Unless otherwise stated, all commercial reagents were used without additional purification. Analytical thin layer chromatography (TLC) was performed on Merck pre-coated silica gel 60 F254 plates. Visualization on TLC was achieved by the use of UV light (254 nm) or treatment with acidic anisaldehyde stain followed by heating. Silica-gel column chromatography was performed using a CombiFlash® *R*f + system with RediSep® *R*f Silica columns (230 – 400 mesh). Preparative TLC was undertaken on PLC Silica gel 60 F_{254} plates (0.5 mm). ^{1}H NMR was recorded on Agilent Technologies DD2 (600 MHz) or Bruker Avance 400 (400 MHz). Chemical shifts are quoted in parts per million (ppm) referenced to the appropriate solvent peak. The following abbreviations are used to describe peak splitting patterns: br (broad), s (singlet), d (doublet), t (triplet), q (quartet), quint (quintet), m (multiplet). Coupling constants, *J*, are reported in hertz (Hz) and rounded to the nearest 0.5. ^{13}C NMR was recorded on Agilent Technologies DD2 (150 MHz) or Bruker Avance 400 (100 MHz), and was fully decoupled by broad band proton decoupling. Chemical shifts are reported in ppm referenced to the appropriate solvent peak. ^{19}F NMR was recorded on Agilent Technologies DD2 (564 MHz) or Bruker Avance 400 (376 MHz). Infrared (IR) spectra were recorded on Bruker Alpha FT-IR Spectrometer. Frequencies are given in reciprocal centimeters (cm^{-1}) and only selected absorbance is reported. High resolution mass spectra were obtained from the Korea Basic Science Institute (Daegu) by using EI method or from KAIST Research Analysis Center by using ESI method. X-ray diffraction data was collected on a Bruker SMART APEX II coated with Paraton-*N* oil under a stream of N_2 (g) at 120 K. Melting point was measured with Buchi Melting Point M-565. High pressure liquid chromatography (HPLC) analysis was performed at 32 °C with Shimadzu Prominence HPLC system composed of LC20A pump, and SPD-M20A photodiode array detector. Optical rotation of $[\alpha]_D$ values of enaniomerically pure compounds were measured using Jasco P-1020 Polarimeter.

Preparation of starting materials that were previously reported

Starting materials **1e** & **1g**[1], **1m**[2], **1s**[3], **3l**[4], **3m**[5], **6a** & **8a**[6], **10**[7], Fenpiprane & Prozapine[8], (chlorodifluoromethyl)trimethylsilane ($TMSCF_2Cl$)[9] and (iododifluoromethyl)trimethylsilane ($TMSCF_2I$)[10] were prepared according to previously reported methods. DL-Laudanosine was synthesised from norlaudanosine according to a previously reported method[11].

Otherwise stated, all other starting materials were purchased from Sigma-Aldrich, TCI chemical company, Alfa Aesar, Acros Organics, or Fluorochem and used without further purification.

General procedures for the preparation of N-arylated cyclic amines

General Procedure (GP1): Starting materials **1d, 1f**, **1l**, **3g**, and **3n** were prepared according to a modified literature procedure[12].

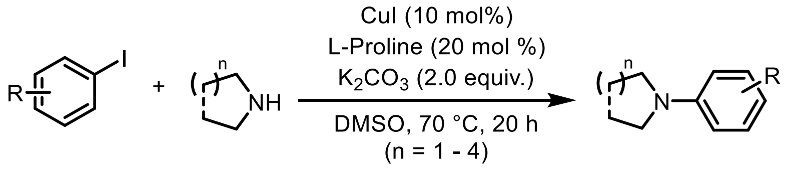

A mixture of iodobenzene (4.3 mmol), cyclic amine (6.4 mmol, 1.5 equiv.), K_2CO_3 (1.2 g, 8.6 mmol, 2.0 equiv.), CuI (82 mg, 0.43 mmol, 10 mol %), and L-proline (99 mg, 0.86 mmol, 20 mol %) in DMSO (2.5 mL) were heated at 70 °C for 20 hours. After the reaction was cooled to room temperature, the reaction was quenched with saturated $NH_4Cl_{(aq)}$ solution and extracted with EtOAc (3 x 50 mL). The organic phase was washed with water and brine, dried over $MgSO_4$, and concentrated *in vacuo*. The residue was purified by column chromatography on silica gel (Hexane/EtOAc) to give the desired N-arylamines.

General Procedure (GP2): Starting materials **1h-i** and **3h** were prepared according to a modified literature procedure[13].

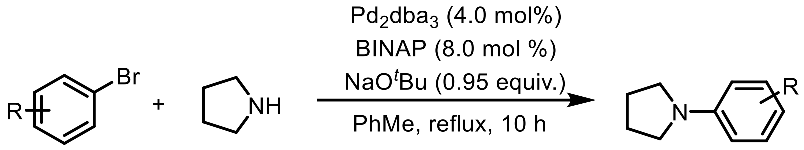

To a reaction flask containing $Pd_2(dba)_3$ (0.11 g, 0.2 mmol, 4.0 mol %) and BINAP (0.25 g, 0.4 mmol, 8.0 mol %) were added freshly distilled toluene (15 mL) under argon atmosphere. The suspension was subsequently stirred at 110 °C for 30 minutes. After cooled to room temperature, NaO^{t}Bu (0.9 g, 9.5 mmol, 0.95 equiv.), bromobenzene (5 mmol), and cyclic amine (10 mmol, 2.0 equiv.) were added into

the solution and stirred under reflux for 10 h. The mixture was then cooled to room temperature, filtered through a pad of celite and washed with DCM (5 x 3mL). The combined organic layers were concentrated *in vacuo*, and the residue was purified by column chromatography on silica gel (Hexane/EtOAc) to give the desired N-arylamines.

Procedure for the preparation of N-protected cyclic amines

5-Tosyl-1,2,3,3a,4,5-hexahydropyrrolo[1,2-*a*]quinoxaline (1o)

To a solution of 1,2,3,3a,4,5-hexahydropyrrolo[1,2-a]quinoxaline (435 mg, 2.5 mmol), 4-(dimethylamino)pyridine (30.5 mg, 0.25 mmol) and triethylamine (0.52 mL, 3.75 mmol) in anhydrous DCM (10 mL) was added tosyl chloride (3.0 mmol). After stirring overnight at room temperature, the solvent was removed under reduced pressure and the residue was partitioned between EtOAc and brine. The organic layer was collected, and the aqueous layer was extracted with EtOAc. The combined organic layers were dried over Na_2SO_4, filtered, and concentrated under reduced pressure. The residue was purified by column chromatography on silica gel (Hexane/EtOAc) to give the desired compound as a purple solid (656 mg, 80%);

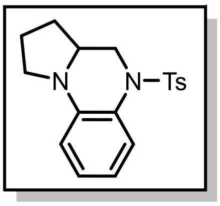

m.p. 175 – 177 °C; **^{1}H NMR** (600 MHz, $CDCl_3$) δ 7.66 (dd, *J* = 8.0, 1.5 Hz, 1H), 7.49 (d, *J* = 8.0 Hz, 2H), 7.18 (d, *J* = 8.0 Hz, 2H), 7.08 (td, *J* = 8.0, 1.5 Hz, 1H), 6.69 – 6.63 (m, 1H), 6.47 (dd, *J* = 8.0, 1.5 Hz, 1H), 4.49 (dd, *J* = 13.5, 4.0 Hz, 1H), 3.36 (t, *J* = 8.5 Hz, 1H), 3.07 (td, *J* = 9.5, 7.0 Hz, 1H), 3.00 – 2.90 (m, 1H), 2.57 (dd, *J* = 13.5, 10.5 Hz, 1H), 2.37 (s, 3H), 2.06 – 1.94 (m, 2H), 1.85 – 1.73 (m, 1H), 1.32 – 1.20 (m, 1H); **^{13}C NMR** (150 MHz, $CDCl_3$) δ 143.6, 138.8, 137.2, 129.8, 127.5, 126.9, 125.8, 121.2, 115.5, 111.8, 54.7, 48.3, 47.6, 30.5, 23.8, 21.8; **IR** (cm^{-1}) 2963, 2911, 1595, 1498, 1344, 1158, 1091, 1068, 1039, 855, 802, 757, 660, 575; **High Resolution MS** (EI): Calculated for $C_{18}H_{20}N_2O_2S$ $[M]^+$: 328.1245, Found: 328.1248.

Preparation of C3-substituted pyrrolidines

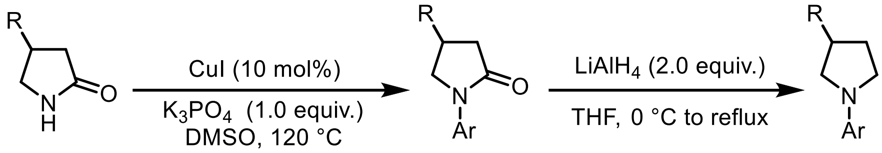

1-(4-Methoxyphenyl)-4-phenylpyrrolidin-2-one

A 50 mL round-bottom flask was charged with 4-phenylpyrrolidin-2-one (806 mg, 5.0 mmol), K_3PO_4 (1.06 g, 5 mmol) and CuI (95 mg, 0.5 mmol). The flask was sealed with a rubber septum, evacuated under vacuum and back-filled with argon. DMSO (10 mL) and 1-iodo-4-methoxybenzene (831 mg, 3.6 mmol) were added, and the reaction mixture was then heated at 120 °C for 24 h. The mixture was cooled to room temperature, diluted with EtOAc (100 mL), and filtered through a short pad of silica. The filtrate was then washed with H_2O, dried over $MgSO_4$, filtered and concentrated *in vacuo*. The title compound was isolated as a brown solid (911 mg, 96%) after column chromatography (0-30% EtOAc/Hexane). The obtained data matched with those reported[14].

1-(4-Methoxyphenyl)-3-phenylpyrrolidine (1p)

To a slurry of $LiAlH_4$ (233 mg, 6.1 mmol) in THF (7 mL) was added a solution of 1-(4-methoxyphenyl)-4-phenylpyrrolidin-2-one (820 mg, 3.1 mmol) in THF (8 mL) at 0 °C under argon atmosphere. The reaction mixture was stirred at 0 °C for 1 h, then at room temperature for 2 h, and refluxed overnight. The mixture was cooled to 0 °C, and H_2O (30 mL) and EtOAc (50 mL) were then slowly added. The organic phase was separated, dried over $MgSO_4$, filtered and concentrated *in vacuo*. The title compound was isolated as a colourless liquid (647 mg, 83%) after silica column chromatography (5% EtOAc/Hexane);

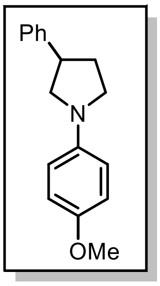

^{1}H NMR (400 MHz, $CDCl_3$) δ 7.41 – 7.28 (m, 5H), 6.93 (d, *J* = 8.5 Hz, 2H), 6.63 (d, *J* = 8.5 Hz, 2H), 3.82 (s, 3H), 3.73 (t, *J* = 8.0 Hz, 1H), 3.61 – 3.43 (m, 3H), 3.38 (t, *J* = 8.0 Hz, 1H), 2.50 – 2.43 (m, 1H), 2.23 – 2.14 (m, 1H); **^{13}C NMR** (100 MHz, $CDCl_3$) δ 150.9, 142.9, 142.6, 128.5, 127.1, 126.5, 115.0, 112.4, 55.9, 55.2, 48.3, 44.1, 33.3; **IR** (cm^{-1}) 2998, 2836, 1682, 1506, 1402, 1356, 1294, 1230, 1031, 831, 746; **High Resolution MS** (EI): Calculated for $C_{17}H_{19}NO$ $[M]^+$: 253.1467, Found: 253.1467.

2-Phenyl-2-azaspiro[4.5]decan-3-one

A 50 mL round-bottom flask was charged with 2-azaspiro[4.5]decan-3-one (1.53 g, 10 mmol), K_3PO_4 (2.12 g, 10 mmol) and CuI (190 mg, 1.0 mmol). The flask was sealed with a rubber septum, evacuated under vacuum and back-filled with argon. DMSO (10 mL) and iodobenzene (0.8 mL, 7.1 mmol) were added, and the reaction mixture was then heated at 120 °C for 24 h. The mixture was cooled to room temperature, diluted in EtOAc (100 mL), and filtered through a short pad of silica. The filtrate was then washed with water, dried over $MgSO_4$, filtered and concentrated *in vacuo*. The title compound was isolated as a pale yellow solid (1.54 g, 94%) after column chromatography (0-30% EtOAc/Hexane);

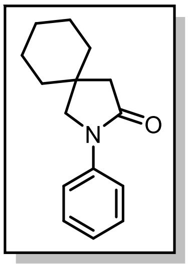

m.p. 104 – 106 °C; **^{1}H NMR** (600 MHz, $CDCl_3$) δ 7.60 (d, *J* = 7.5 Hz, 2H), 7.35 (t, *J* = 7.5 Hz, 2H), 7.12 (t, *J* = 7.5 Hz, 1H), 3.58 (s, 2H), 2.46 (s, 2H), 1.61 – 1.55 (m, 4H), 1.53 – 1.48 (m, 4H), 1.45 – 1.40 (m, 2H); **^{13}C NMR** (150 MHz, $CDCl_3$) δ 173.2, 139.5, 128.7, 124.2, 119.7, 59.8, 45.3, 36.5, 35.6, 25.5, 22.7; **IR** (cm^{-1}) 2919, 2847, 1685, 1597, 1498, 1399, 1316, 1226, 1124, 747, 687, 503; **High Resolution MS** (EI): Calculated for $C_{15}H_{19}NO$ $[M]^+$: 229.1467, Found: 229.1468.

2-Phenyl-2-azaspiro[4.5]decane (1q)

To a slurry of $LiAlH_4$ (228 mg, 6.0 mmol) in THF (7 mL) was added a solution of 2-phenyl-2-azaspiro[4.5]decan-3-one (688 mg, 3.0 mmol) in THF (8 mL) at 0 °C under argon atmosphere. The reaction mixture was stirred at 0 °C for 1 h, then at room temperature for 2 h, and refluxed overnight. The mixture was cooled to 0 °C, and H_2O (10 mL) and EtOAc (10 mL) were then slowly added. The organic phase was separated, dried over $MgSO_4$, filtered and concentrated *in vacuo*. The title compound was isolated as a colourless liquid (512 mg, 79%) after column chromatography (100% Hexane);

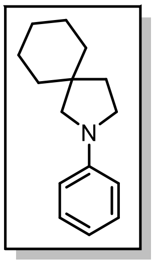

^{1}H NMR (400 MHz, $CDCl_3$) δ 7.28 – 7.22 (m, 2H), 6.68 (t, *J* = 7.0 Hz, 1H), 6.58 (d, *J* = 7.0 Hz, 2H), 3.36 (t, *J* = 7.0 Hz, 2H), 3.14 (s, 2H), 1.85 (t, *J* = 7.0 Hz, 2H), 1.61 – 1.43 (m, 10H); **^{13}C NMR** (100 MHz, $CDCl_3$) δ 148.0, 129.1, 115.1, 111.3, 58.4, 46.2, 42.0, 37.0, 36.1, 26.2, 23.6; **IR** (cm^{-1}) 2919, 2845, 1595, 1505, 1483, 1448, 1369, 992, 744; **High Resolution MS** (EI): Calculated for $C_{15}H_{21}N$ $[M]^+$: 215.1674, Found: 215.1674.

Preparation of C2-substituted pyrrolidines

(*S*)-(1-Methylpyrrolidin-2-yl)methyl acetate (1r)

To a solution of (*S*)-(1-methylpyrrolidin-2-yl)methanol (346 mg, 3.0 mmol), pyridine (483 μL, 6.0 mmol) and 4-dimethylaminopyridine (18 mg, 0.15 mmol) in DCM (10 mL) was added acetic anhydride (566 μL, 6.0 mmol) at 0 °C. After stirring at room temperature for 1 h, the reaction mixture was quenched by an addition of saturated $NaHCO_{3(aq)}$ solution (15 mL) and extracted with DCM (3 × 15 mL). The combined organic layers were dried over Na_2SO_4, filtered, and concentrated under reduced pressure. The residue was purified by column chromatography on silica gel (10% MeOH/DCM) to give the title compound as a yellow liquid (371 mg, 79%);

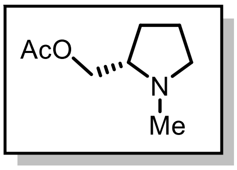

^{1}H NMR (400 MHz, $CDCl_3$) δ 4.07 (dd, *J* = 11.0, 5.0 Hz, 1H), 4.00 (dd, *J* = 11.0, 5.0 Hz, 1H), 3.05 (ddd, *J* = 9.5, 7.5, 2.0 Hz, 1H), 2.45 – 2.40 (m, 1H), 2.38 (s, 3H), 2.22 (td, *J* = 9.5, 7.5 Hz, 1H), 2.05 (s, 3H), 1.95 – 1.86 (m, 1H), 1.80 – 1.65 (m, 2H), 1.63 – 1.54 (m, 1H); **^{13}C NMR** (100 MHz, $CDCl_3$) δ 171.3, 66.6, 63.9, 57.8, 41.5, 28.4, 22.9, 21.1; **IR** (cm^{-1}) 3053, 2984, 1735, 1421, 1264, 908, 733; **High Resolution MS** (EI): Calculated for $C_8H_{15}NO_2$ $[M]^+$: 157.1103, Found: 157.1100; **Specific Rotation** $[\alpha]_D^{20}$ –51.0±0.5 (*c* 1.0, CH_3Cl).

Preparation of C4-substituted piperidine

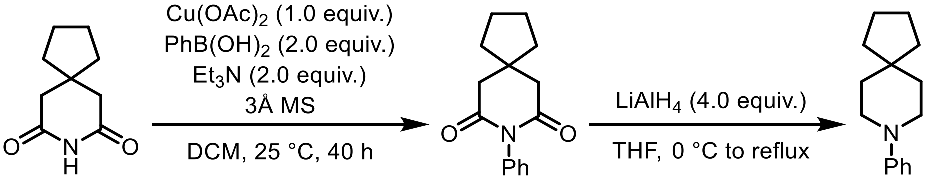

8-Phenyl-8-azaspiro[4.5]decane-7,9-dione

8-Azaspiro[4.5]decane-7,9-dione (836 mg, 5 mmol), phenylboronic acid (1.22 g, 10 mmol), $Cu(OAc)_2$ (908 mg, 5 mmol) and 3Å molecular sieves (500 mg) were weighed into a 50 mL round-bottom flask under argon atmosphere. The flask was sealed with a rubber septum, and to this was added anhydrous DCM (20 mL) and NEt_3 (1.4 mL, 10 mmol). The reaction mixture was stirred for 40 h at room temperature under argon atmosphere, and was then filtered through a short pad of silica, rinsed with EtOAc and concentrated *in vacuo*. The title compound was isolated as a pinkish solid (1.03 g, 84%) after column chromatography (0-50% EtOAc/Hexane);

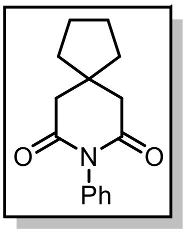

m.p. 139 – 141 °C; **^{1}H NMR** (600 MHz, $CDCl_3$) δ 7.46 (t, *J* = 7.5 Hz, 2H), 7.40 (t, *J* = 7.5 Hz, 1H), 7.09 (d, *J* = 7.5 Hz, 2H), 2.74 (s, 4H), 1.77 – 1.75 (m, 4H), 1.65 – 1.62 (m, 4H); **^{13}C NMR** (150 MHz, $CDCl_3$) δ 172.0, 134.9, 129.0, 128.3, 128.2, 44.8, 39.5, 37.4, 24.0; **IR** (cm^{-1}) 2939, 2873, 1730, 1677, 1488, 1371, 1244, 1146, 697; **High Resolution MS** (EI): Calculated for $C_{15}H_{17}NO_2$ $[M]^+$: 243.1259, Found: 243.1263.

8-Phenyl-8-azaspiro[4.5]decane (3i)

To a slurry of $LiAlH_4$ (445 mg, 12 mmol) in THF (7 mL) was added a solution of 8-phenyl-8-azaspiro[4.5]decane-7,9-dione (730 mg, 3.0 mmol) in THF (8 mL) at 0 °C under argon atmosphere. The reaction mixture was stirred at 0 °C for 1 h, then at room temperature for 2 h, and refluxed overnight. The mixture was cooled to 0 °C, and H_2O (10 mL) and EtOAc (10 mL) were then slowly added. The organic phase was separated, dried over $MgSO_4$, filtered and concentrated *in vacuo*. The title compound was isolated as a white solid (245 mg, 38%) after column chromatography (0-10% EtOAc/Hexane);

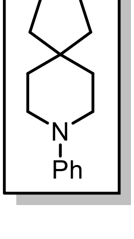

m.p. 40 – 42 °C; **^{1}H NMR** (400 MHz, $CDCl_3$) δ 7.32 – 7.27 (m, 2H), 7.01 (d, *J* = 7.5 Hz, 2H), 6.87 (t, *J* = 7.5 Hz, 1H), 3.22 – 3.19 (m, 4H), 1.71 – 1.63 (m, 8H), 1.54 – 1.48 (m, 4H); **^{13}C NMR** (100 MHz, $CDCl_3$) δ 151.8, 129.0, 119.1, 116.3, 47.4, 40.6, 37.7, 37.3, 24.3; **IR** (cm^{-1}) 3053, 2924, 2859, 1598, 1497, 1143, 896, 736; **High Resolution MS** (EI): Calculated for $C_{15}H_{21}N$ $[M]^+$: 215.1674, Found: 215.1673.

Preparation of N-alkyl cyclic amines

General Procedure (GP3): Starting materials **3b–3f**, **3k**, **3r** and **6b** were prepared following this procedure.

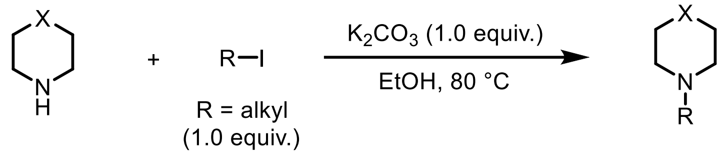

To a mixture of cyclic amine (1.0 equiv.) and K_2CO_3 (1.0 equiv.) in EtOH was added the corresponding alkyl iodide (1.0 equiv.), and the mixture was heated overnight at 80 °C. It was then cooled to room temperature, filtered and concentrated *in vacuo*. The title compound was isolated after distillation or column chromatography.

4-(3,3-Dimethylbutyl)morpholine (3k)

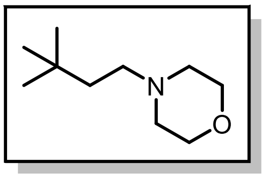

The reaction was performed on a 10 mmol scale and purified by distillation; Colourless liquid (410 mg, 24%); **^{1}H NMR** (400 MHz, $CDCl_3$) δ 3.83 – 3.57 (m, 4H), 2.44 (t, *J* = 4.8 Hz, 4H), 2.37 – 2.27 (m, 2H), 1.44 – 1.34 (m, 2H), 0.90 (s, 9H); **^{13}C NMR** (100 MHz, $CDCl_3$) δ 67.3, 55.4, 54.2, 40.4, 29.9, 29.7; **IR** (cm^{-1}) 2956, 2861, 1116, 906, 729, 651; **High Resolution MS** (ESI): Calculated for $C_{10}H_{22}NO$ $[M+H]^+$: 172.1696, Found: 172.1695.

1-Butyl-2-phenylpiperidine (3r)

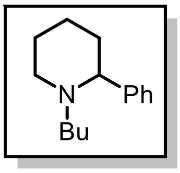

The reaction was performed on a 5.0 mmol scale and purified by column chromatography (20% EtOAc/Hexane); Colourless liquid (951 mg, 88%); **^{1}H NMR** (400 MHz, $CDCl_3$) δ 7.36 – 7.28 (m, 4H), 7.26 – 7.22 (m, 1H), 3.23 – 3.18 (m, 1H), 3.01 (dd, *J* = 11.0, 2.5 Hz, 1H), 2.44 (dt, *J* = 12.5, 8.0 Hz, 1H), 2.05 (td, *J* = 11.5, 3.0 Hz, 1H), 1.90 (dt, *J* = 13.0, 7.0 Hz, 1H), 1.84 – 1.77 (m, 1H), 1.75 – 1.68 (m, 3H), 1.59 (qd, *J* = 13.0, 3.5 Hz, 1H), 1.43 – 1.32 (m, 3H), 1.26 – 1.04 (m, 2H), 0.80 (t, *J* = 7.0 Hz, 3H); **^{13}C NMR** (100 MHz, $CDCl_3$) δ 145.7, 128.4, 127.6, 126.8, 69.3, 55.3, 53.4, 36.9, 28.4, 26.3, 25.3, 20.7, 14.2; **IR** (cm^{-1}) 2953, 2929, 2855, 1451, 1093, 1051, 156, 701; **High Resolution MS** (ESI): Calculated for $C_{15}H_{24}N$ $[M+H]^+$: 218.1903, Found: 218.1897.

Preparation of 1-butylazocane

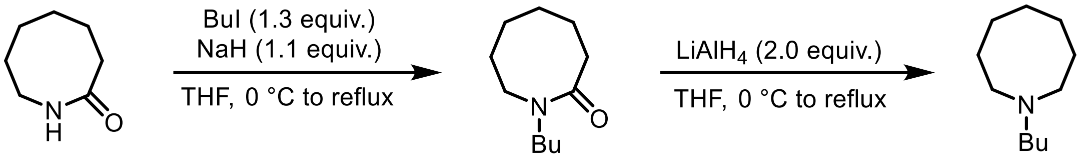

1-Butylazocan-2-one

To a slurry of NaH (331 mg, 8.25 mmol, 60% w/w dispersion in mineral oil) in THF (10 mL) was added a solution of azocan-2-one (960 mg, 7.5 mmol) in THF (10 mL) at 0 °C under argon atmosphere. After stirring at the same temperature for 1 h, 1-iodobutane (1.1 mL, 10 mmol) was added, and the reaction mixture was refluxed for 12 h. It was then cooled to room temperature, quenched with a slow addition of H_2O (50 mL), extracted with EtOAc (3 × 50 mL), dried over $MgSO_4$, filtered and concentrated *in vacuo* to give 1-butylazocan-2-one. The crude product was directly used for the next step without further purification.

1-Butylazocane (8b)

To a slurry of $LiAlH_4$ (1.47 g, 15 mmol) in THF (20 mL) was added the above obtained crude in THF (20 mL) at 0 °C under argon atmosphere. The reaction mixture was stirred at 0 ℃ for 1 h, then at room temperature for 1 h, and refluxed overnight. The mixture was cooled to 0 ℃, and H_2O (10 mL) was then slowly added, followed by EtOAc (10 mL). The organic phase was separated, dried over $MgSO_4$, filtered and concentrated *in vacuo*. The title compound was isolated as a colourless liquid (1.21 g, 95%) after column chromatography (50% EtOAc/Hexane);

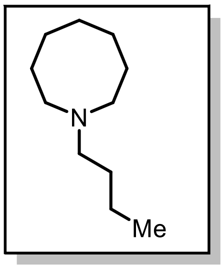

^{1}H NMR (400 MHz, $CDCl_3$) δ 2.54 – 2.49 (m, 4H), 2.42 – 2.39 (m, 2H), 1.65 – 1.60 (m, 2H), 1.59 – 1.52 (m, 8H), 1.45 – 1.36 (m, 2H), 1.35 – 1.28 (m, 2H), 0.90 (t, *J* = 7.0 Hz, 3H); **^{13}C NMR** (100 MHz, $CDCl_3$) δ 58.9, 54.2, 30.7, 28.1, 27.5, 26.5, 20.8, 14.3; **IR** (cm^{-1}) 2916, 2854, 1454, 1359, 1249, 1159, 1095; **High Resolution MS** (ESI): Calculated for $C_{11}H_{24}N$ $[M+H]^+$: 170.1903, Found: 170.1901.

Preparation of (*S*)-4-(3,7-dimethyloct-6-en-1-yl)morpholine

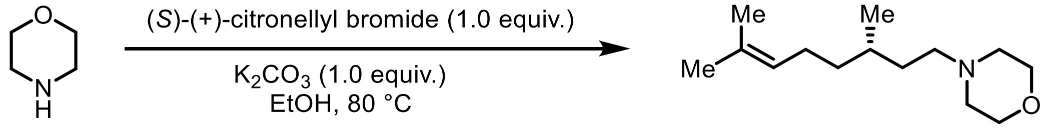

To a mixture of morpholine (437 µL, 5.0 mmol) and K_2CO_3 (690 mg, 5.0 mmol) in EtOH (13 mL) was added (*S*)-(+)-citronellyl bromide (987 µL, 5.0 mmol), and the mixture was heated overnight at 80 °C. It was then cooled to room temperature, filtered and concentrated *in vacuo*. The title compound was isolated as a colourless liquid (1.03 g, 91%) after column chromatography (50% EtOAc/Hexane);

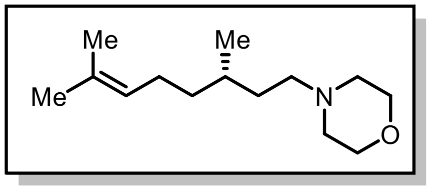

^{1}H NMR (400 MHz, $CDCl_3$) δ 5.11 – 5.05 (m, 1H), 3.72 – 3.69 (m, 4H), 2.46 – 2.39 (m, 4H), 2.37 – 2.27 (m, 2H), 2.03 – 1.88 (m, 2H), 1.67 (s, 3H), 1.59 (s, 3H), 1.56 – 1.39 (m, 2H), 1.36 – 1.26 (m, 2H), 1.20 – 1.11 (m, 1H), 0.88 (d, *J* = 6.5 Hz, 3H); **^{13}C NMR** (100 MHz, $CDCl_3$) δ 131.3, 124.9, 67.2, 57.4, 54.0, 37.3, 33.7, 31.2, 25.8, 25.6, 19.8, 17.8; **IR** (cm^{-1}) 2955, 2916, 2852, 1447, 1375, 1262, 1119, 1004, 868; **High Resolution MS** (ESI): Calculated for $C_{14}H_{28}NO$ $[M+H]^+$: 226.2165, Found: 226.2170; **Specific Rotation** $[\alpha]_D^{20}$ –10.8±1.0 (*c* 1.0, CH_3Cl).

Procedure for the optimization study

To a 2.0 mL reaction vial containing a magnetic stir bar were added *N*-phenylpyrrolidine (0.2 mmol), base and solvent (0.5 mL), followed by electrophilic source. The reaction vial was sealed and stirred at the indicated temperature for 12 h. The reaction mixture was cooled to room temperature and filtered through a plug of celite and then washed with EtOAc. The solvents were removed under reduced pressure and the crude yield was measured by ^{1}H NMR using dibromomethane as an internal standard.

Supplementary Table 1. Optimization of the Reaction Conditions[a].

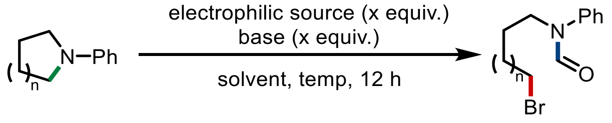

entry	n	electrophilic source (equiv.)	base (equiv.)	solvent	temp (°C)	yield (%)
1	1	$TMSCF_2Br$ (4.0)	NaOAc (4.0)	$PhCF_3$	25	5
2	1	$TMSCF_2Br$ (4.0)	NaOAc (4.0)	$PhCF_3$	40	12
3	1	$TMSCF_2Br$ (4.0)	NaOAc (4.0)	$PhCF_3$	60	24
4	1	$TMSCF_2Br$ (4.0)	NaOAc (4.0)	$PhCF_3$	80	28
5	1	$TMSCF_2Br$ (4.0)	-	$PhCF_3$	80	n.d.
6	1	$TMSCF_2Br$ (4.0)	NaOAc (4.0)	MeCN	25	11
7	1	$TMSCF_2Br$ (4.0)	NaOAc (4.0)	CyH	25	trace
8	1	$TMSCF_2Br$ (4.0)	NaOAc (4.0)	1,2-DCE	25	11
**9**	**1**	**$TMSCF_2Br$ (4.0)**	**NH_4OAc (4.0)**	**1,2-DCE**	**25**	**88**
10	1	$TMSCF_2Br$ (4.0)	NH_4OAc (4.0)	PhMe	25	85
**11**[b]	**1**	**$TMSCF_2Br$ (4.0)**	**CsF (4.0)**	**1,2-DCE**	**25**	**90**
12	1	$TMSCF_2Br$ (2.0)	NH_4OAc (2.0)	1,2-DCE	25	42
13	1	$TMSCCl_2Br$ (4.0)	NH_4OAc (4.0)	1,2-DCE	25	n.d.
14	1	$BrCF_2PO(OEt)_2$ (4.0)	NH_4OAc (4.0)	1,2-DCE	25	n.d.
15[c]	1	$BrCF_2PO(OEt)_2$ (4.0)	CsF (4.0)	1,2-DCE	25	trace
16[c]	1	$BrCF_2PO(OEt)_2$ (4.0)	CsF (4.0)	1,2-DCE	60	5
17	2	$TMSCF_2Br$ (4.0)	NH_4OAc (4.0)	1,2-DCE	25	n.d.[d]
**18**	**2**	**$TMSCF_2Br$ (4.0)**	**NH_4OAc (4.0)**	**1,2-DCE**	**60**	**80**
**19**	**3**	**$TMSCF_2Br$ (4.0)**	**NH_4OAc (4.0)**	**1,2-DCE**	**25**	**80**
20	1	isopropyl chloroformate (1.4)	-	1,2-DCE	25	n.d
21	2	isopropyl chloroformate (1.4)	-	1,2-DCE	25	n.d
22	2	isopropyl chloroformate (1.4)	-	1,2-DCE	80	n.d
23	3	isopropyl chloroformate (1.4)	-	1,2-DCE	25	n.d

[a]Standard reaction conditions: *N*-phenylpyrrolidine (0.2 mmol), base and difluorocarbene source in solvent (0.5 mL) at the indicated temperature for 12 h. Yield was determined by ^{1}H NMR analysis of the crude reaction mixture using dibromomethane as an internal standard; n.d., not detected. 1,2-DCE, 1,2-dichloroethane. CyH, cyclohexane. [b]H_2O was used as a co-solvent (0.05 mL). [c]MeOH was used as an additive (32 μL, 4.0 equiv). [d]95% of *N*-difluoromethyl-*N*-phenyl-piperidinium bromide was formed.

Procedures for the preparation of N-CF_2H ammonium salts

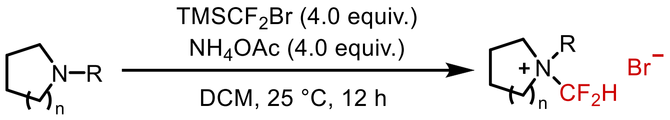

To a 2.0 mL reaction vial containing a magnetic stir bar were added cyclic amine (0.2 mmol), NH_4OAc (61.7 mg, 0.8 mmol) and dichloromethane (0.5 mL), followed by $TMSCF_2Br$ (124 μL, 0.8 mmol). The reaction vial was sealed and stirred at 25 °C for 12 h. The crude product was filtered through a pad of celite, washed with DCM (10 mL × 3) and concentrated under reduced pressure. The residue was then diluted in EtOAc, decanted, and filtered to give the title compound.

***N*-(Difluoromethyl)-*N*-ethylpyrrolidinium bromide (int1)**

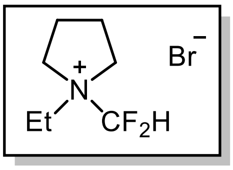

White solid (36 mg, 78%); **m.p.** 109 – 110 °C; **^{1}H NMR** (600 MHz, $CDCl_3$) δ 8.29 (t, J = 59.0 Hz, 1H), 4.34 – 4.13 (m, 2H), 4.00 (q, J = 7.5 Hz, 2H), 3.93 – 3.77 (m, 2H), 2.53 – 2.15 (m, 4H), 1.53 (t, J = 7.5 Hz, 3H); **^{13}C NMR** (150 MHz, $CDCl_3$) δ 114.6 (t, J = 275.0 Hz), 59.3, 56.9, 24.2, 10.0; **^{19}F NMR** (564 MHz, CD_2Cl_2) δ -111.1 (d, J = 59.0 Hz); **IR** (cm^{-1}) 2994, 2976, 1660, 1465, 1400, 1379, 1138, 1117, 1099, 806, 665, 563; **High Resolution MS** (FAB): Calculated for $C_7H_{14}F_2N$ [M-Br]$^+$: 150.1089, Found: 150.1093.

The structure of the title compound was further confirmed by X-ray crystallographic analysis (see X-ray Crystallographic Data).

***N*-(Difluoromethyl)-*N*-ethylpiperidin-1-ium bromide (int2)**

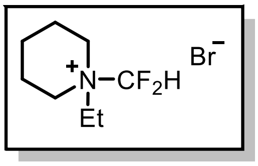

White solid (41 mg, 85%); **m.p.** 158 – 160 °C; **^{1}H NMR** (400 MHz, $CDCl_3$) δ 8.32 (t, J = 58.4 Hz, 1H), 4.19 (t, J = 12.2 Hz, 2H), 3.93 (q, J = 7.4 Hz, 2H), 3.78 (dd, J = 10.2, 6.2 Hz, 2H), 2.11 – 1.72 (m, 6H), 1.49 (t, J = 7.4 Hz, 3H); **^{13}C NMR** (100 MHz, $CDCl_3$) δ 115.4 (t, J = 277.9 Hz), 54.4 (2C), 50.4, 20.3, 19.6 (2C), 9.4; **^{19}F NMR** (376 MHz, $CDCl_3$) δ -113.5 (d, J = 59.0 Hz); **IR** (cm^{-1}) 2956, 1456, 1391, 1267, 1140, 1122, 966, 882, 729, 699; **High Resolution MS** (ESI): Calculated for $C_8H_{16}F_2N$ [M-Br]$^+$: 164.1245, Found: 164.1245.

The structure of the title compound was further confirmed by X-ray crystallographic analysis (see X-ray Crystallographic Data).

N-(Difluoromethyl)-N-(3,3-dimethylbutyl)piperidinium bromide (int3)

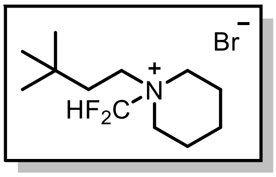

White solid (49 mg, 82%); **m.p.** 168 – 170 °C; **^{1}H NMR** (400 MHz, $CDCl_3$) δ 8.47 (t, *J* = 58.5 Hz, 1H), 4.53 (t, *J* = 12.4 Hz, 2H), 3.67 (d, *J* = 10.9 Hz, 4H), 2.10 (d, *J* = 14.6 Hz, 2H), 2.00 – 1.77 (m, 4H), 1.73 – 1.65 (m, 2H), 1.02 (s, 9H); **^{13}C NMR** (100 MHz, $CDCl_3$) δ 115.3 (t, *J* = 278.5 Hz), 54.9 (2C), 51.9, 35.7, 30.6, 29.2 (3C), 20.2, 19.7 (2C); **^{19}F NMR** (376 MHz, $CDCl_3$) δ -112.8 (d, *J* = 58.4 Hz); **IR** (cm^{-1}) 2959, 2933, 1470, 1371, 1131, 1055, 986, 820, 580; **High Resolution MS** (ESI): Calculated for $C_{12}H_{24}F_2N$ $[M-Br]^+$: 220.1871, Found: 220.1871.

The structure of the title compound was further confirmed by X-ray crystallographic analysis (see X-ray Crystallographic Data).

General procedures for deconstructive C–N bromoformylation of cyclic amines

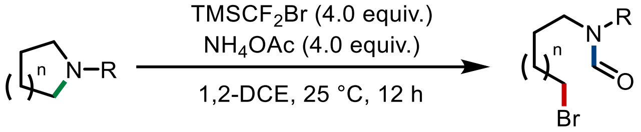

To a 2.0 mL reaction vial containing a magnetic stir bar were added cyclic amine (0.2 mmol), NH_4OAc (61.7 mg, 0.8 mmol) and 1,2-dichloroethane (0.5 mL), followed by $TMSCF_2Br$ (124 µL, 0.8 mmol). The reaction vial was sealed and stirred at 25 °C for 12 h, unless otherwise noted. The crude product was filtered through a pad of celite, washed with EtOAc (10 mL x 3) and concentrated under reduced pressure. The residue was purified by column chromatography on silica gel (Hexane/EtOAc) to give the desired compound.

***N*-(4-Bromobutyl)-*N*-phenylformamide (2a)**

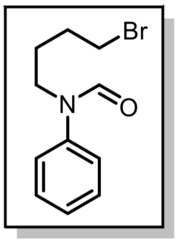

Colourless liquid (43 mg, 85%); *A 17:1 mixture of rotamers, Spectral data of major rotamer is reported*; **^{1}H NMR** (600 MHz, $CDCl_3$) δ 8.37 (s, 1H), 7.42 (t, *J* = 7.5 Hz, 2H), 7.31 (t, *J* = 7.5 Hz, 1H), 7.17 (d, *J* = 7.5 Hz, 2H), 3.86 (t, *J* = 7.0 Hz, 2H), 3.39 (t, *J* = 6.5 Hz, 2H), 1.86 (m, 2H), 1.70 (m, 2H); **^{13}C NMR** (150 MHz, $CDCl_3$) δ 162.4, 140.7, 129.8, 127.0, 124.3, 43.8, 33.0, 29.7, 26.2; **IR** (cm^{-1}) 2933, 2865, 1669, 1594, 1495, 1355, 1266, 764, 699; **High Resolution MS** (EI): Calculated for $C_{11}H_{14}BrNO$ $[M]^+$: 255.0259, Found: 255.0262.

***N*-(4-Bromobutyl)-*N*-ethylformamide (2b)**

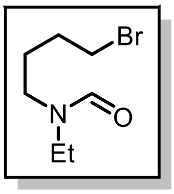

The reaction was performed at 60°C using $TMSCF_2Br$ (34 µL, 0.22 mmol, 1.1 equiv.) and NH_4OAc (17 mg, 0.22 mmol, 1.1 equiv.); Colourless liquid (32 mg, 78%); *Data recorded on a 1.3:1 mixture of rotamers*; **^{1}H NMR** (400 MHz, $CDCl_3$) δ 8.08 (s, 1H major), 8.02 (s, 1H minor), 3.46 – 3.40 (m, 2H major + 2H minor), 3.36 (q, *J* = 7.0 Hz, 2H minor), 3.34 (t, *J* = 7.0 Hz, 2H major), 3.28 (q, *J* = 7.0 Hz, 2H major), 3.25 (t, *J* = 7.0 Hz, 2H minor), 1.91 – 1.79 (m, 2H major + 2H minor), 1.76 – 1.65 (m, 2H major + 2H minor), 1.20 (t, *J* = 7.0 Hz, 3H major), 1.13 (t, *J* = 7.0 Hz, 3H minor); **^{13}C NMR** (100 MHz, $CDCl_3$) δ 162.7 (major), 162.5 (minor), 46.4 (minor), 42.2 (major), 40.7 (major), 37.1 (minor), 33.4 (major), 32.9 (minor), 29.9 (major), 29.6 (minor), 27.4 (minor), 26.0 (major), 14.9 (major), 12.8 (minor); **IR** (cm^{-1}) 2966, 2935, 2866, 1662, 1429, 1398, 1256; **High Resolution MS** (EI): Calculated for $C_7H_{14}BrNO$ $[M]^+$: 207.0259, Found: 207.0255.

***N*-(4-Bromobutyl)-*N*-methylformamide (2c)**

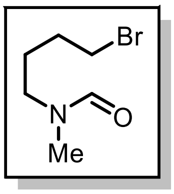

The reaction was performed at 60°C using TMSCF$_2$Br (34 µL, 0.22 mmol, 1.1 equiv.) and NH$_4$OAc (17 mg, 0.22 mmol, 1.1 equiv.); Colourless liquid (32 mg, 83%); *Data recorded on a 1.3:1 mixture of rotamers*; **^{1}H NMR** (600 MHz, CDCl$_3$) δ 8.04 (s, 1H major + 1H minor), 3.49 – 3.40 (m, 2H major + 2H minor), 3.37 (t, *J* = 7.0 Hz, 2H minor), 3.27 (t, *J* = 7.0 Hz, 2H major), 2.94 (s, 3H minor), 2.87 (s, 3H major), 1.91 – 1.80 (m, 2H major + 2H minor), 1.80 – 1.67 (m, 2H major + 2H minor); **^{13}C NMR** (150 MHz, CDCl$_3$) δ 162.7 (minor), 162.5 (major), 48.7 (major), 43.0 (minor), 34.4 (minor), 33.2 (minor), 32.7 (major), 29.6 (major), 29.4 (minor), 29.3 (major), 26.5 (major), 25.1 (minor); **IR** (cm^{-1}) 2916. 2848, 1711, 1672, 1360, 1221, 966, 747, 531; **High Resolution MS** (EI): Calculated for $C_6H_{12}BrNO$ [M]$^+$: 193.0102, Found: 193.0103.

***N*-(4-Bromobutyl)-*N*-(4-methoxyphenyl)formamide (2d)**

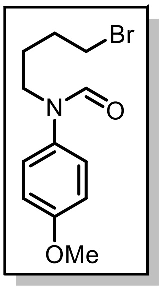

Yellow liquid (48 mg, 85%); *A 13:1 mixture of rotamers, Spectral data of major rotamer is reported*; **^{1}H NMR** (400 MHz, CDCl$_3$) δ 8.25 (s, 1H), 7.08 (d, *J* = 9.0 Hz, 2H), 6.92 (d, *J* = 9.0 Hz, 2H), 3.81 (s, 3H), 3.77 (t, *J* = 7.0 Hz, 2H), 3.38 (t, *J* = 6.5 Hz, 2H), 1.85 (app. quint., *J* = 6.5 Hz, 2H), 1.66 (app. quint., *J* = 7.0 Hz, 2H); **^{13}C NMR** (100 MHz, CDCl$_3$) δ 162.5, 158.7, 133.4, 126.4, 114.8, 55.5, 44.2, 33.0, 29.7, 26.1; **IR** (cm^{-1}) 2917, 2876, 2842, 2800, 1672, 1512, 1203, 982, 745; **High Resolution MS** (EI): Calculated for $C_{12}H_{16}BrNO_2$ [M]$^+$: 285.0364, Found: 285.0363.

***N*-(4-Bromobutyl)-*N*-[4-(trifluoromethyl)phenyl]formamide (2e)**

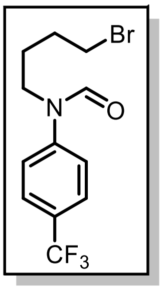

Colourless liquid (54 mg, 84%); *A 11:1 mixture of rotamers, Spectral data of major rotamer is reported*; **^{1}H NMR** (400 MHz, CDCl$_3$) δ 8.47 (s, 1H), 7.70 (d, *J* = 8.5 Hz, 2H), 7.30 (d, *J* = 8.5 Hz, 2H), 3.91 (t, *J* = 7.0 Hz, 2H), 3.40 (t, *J* = 6.5 Hz, 2H), 1.94 – 1.81 (m, 2H), 1.78 – 1.67 (m, 2H); **^{13}C NMR** (100 MHz, CDCl$_3$) δ 162.1, 143.9, 129.0 (q, *J* = 33.1 Hz), 127.2 (q, *J* = 4.0 Hz), 123.9 (q, *J* = 272.0 Hz), 123.5, 43.6, 32.9, 29.8, 26.3; **^{19}F NMR** (376 MHz, CDCl$_3$) δ -62.5; **IR** (cm^{-1}) 2941, 2872, 1677, 1612, 1322, 1112, 1069, 842; **High Resolution MS** (EI): Calculated for $C_{12}H_{13}BrF_3NO$ [M]$^+$: 323.0133, Found: 323.0134.

***N*-(4-Acetylphenyl)-*N*-(4-bromobutyl)formamide (2f)**

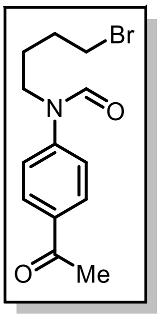

Colourless liquid (46 mg, 77%); *A 11:1 mixture of rotamers, Spectral data of major rotamer is reported*; **^{1}H NMR** (400 MHz, $CDCl_3$) δ 8.51 (s, 1H), 8.01 (d, *J* = 8.5 Hz, 2H), 7.37 – 7.15 (m, 2H), 3.90 (t, *J* = 7.0 Hz, 2H), 3.38 (t, *J* = 6.5 Hz, 2H), 2.60 (s, 3H), 1.93 – 1.78 (m, 2H), 1.76 – 1.67 (m, 2H); **^{13}C NMR** (100 MHz, $CDCl_3$) δ 196.8, 162.1, 144.9, 135.2, 130.3, 122.6, 43.4, 33.0, 29.8, 26.7, 26.3; **IR** (cm^{-1}) 2932, 2870, 1673, 1598, 1357, 1266, 841, 751; **High Resolution MS** (EI): Calculated for $C_{13}H_{16}BrNO_2$ $[M]^+$: 297.0364, Found: 297.0361.

4-[*N*-(4-Bromobutyl)formamido]-*N*,*N*-dimethylbenzamide (2g)

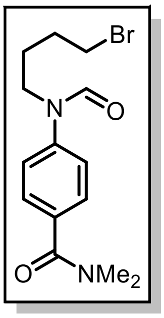

Yellow liquid (59 mg, 91%); *A 13:1 mixture of rotamers, Spectral data of major rotamer is reported*; **^{1}H NMR** (600 MHz, $CDCl_3$) δ 8.39 (s, 1H), 7.47 (d, *J* = 8.0 Hz, 2H), 7.19 (d, *J* = 8.0 Hz, 2H), 3.85 (t, *J* = 7.0 Hz, 2H), 3.36 (t, *J* = 6.5 Hz, 2H), 3.09 (s, 3H), 2.99 (s, 3H), 1.87 – 1.78 (m, 2H), 1.72 – 1.62 (m, 2H); **^{13}C NMR** (150 MHz, $CDCl_3$) δ 170.5, 162.2, 141.7, 134.9, 128.9, 123.6, 43.6, 39.7, 35.5, 33.0, 29.7, 26.2; **IR** (cm^{-1}) 2933, 2869, 1671, 1626, 1391, 1262, 1083, 748; **High Resolution MS** (EI): Calculated for $C_{14}H_{19}BrN_2O_2$ $[M]^+$: 326.0630, Found: 326.0628.

***N*-[2-(Bromomethyl)benzyl]-*N*-phenylformamide (2h)**

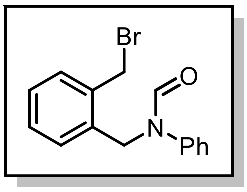

Yellow liquid (45 mg, 75%); *A 19:1 mixture of rotamers, Spectral data of major rotamer is reported*; **^{1}H NMR** (600 MHz, $CDCl_3$) δ 8.58 (s, 1H), 7.38 – 7.35 (m, 2H), 7.36 – 7.31 (m, 1H), 7.29 – 7.26 (m, 1H), 7.24 – 7.20 (m, 2H), 7.17 – 7.14 (m, 2H), 7.14 – 7.11 (m, 1H), 5.16 (s, 2H), 4.57 (s, 2H); **^{13}C NMR** (150 MHz, $CDCl_3$) δ 162.8, 141.1, 136.0, 135.4, 131.2, 130.1, 129.5, 129.3, 128.5, 127.5, 124.5, 46.0, 31.6; **IR** (cm^{-1}) 3034, 2873, 1667, 1594, 1494, 1353, 1218, 758, 696, 604; **High Resolution MS** (ESI): Calculated for $C_{15}H_{14}BrNONa$ $[M+Na]^+$: 326.0151, Found: 326.0156.

_N_-[2-(2-Bromoethyl)phenyl]-_N_-phenylformamide (2i)

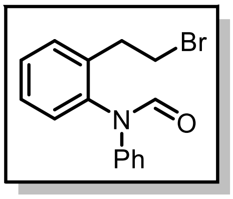

Pale blue liquid (50 mg, 83%); *Data recorded on a 2:1 mixture of rotamers*; **^{1}H NMR** (600 MHz, $CDCl_3$) δ 8.84 (s, 1H major), 8.49 (s, 1H minor), 7.44 – 7.18 (m, 7H major + 9H minor), 7.10 (d, *J* = 8.0 Hz, 2H major), 3.39 (app. s, 2H major), 3.26 (t, *J* = 7.5 Hz, 2H minor), 3.03 (t, *J* = 8.0 Hz, 2H major), 2.99 (t, *J* = 7.5 Hz, 2H minor); **^{13}C NMR** (150 MHz, $CDCl_3$) δ 162.1 (major), 162.0 (minor), 141.7 (major), 140.0 (minor), 139.4 (minor), 137.7 (major), 137.1 (minor), 136.8 (major), 131.6 (minor), 130.8 (minor), 130.3 (minor), 129.9 (major), 129.7 (major), 129.3 (minor), 129.2 (major), 129.0 (major), 128.7 (minor), 128.6 (major), 126.5 (major), 126.1 (minor), 123.7 (minor), 122.4 (major), 35.2 (major), 34.5 (minor), 31.0 (minor), 30.8 (major); **IR** (cm^{-1}) 3052, 2897, 1681, 1592, 1491, 1264, 731, 697, 675; **High Resolution MS** (EI): Calculated for $C_{15}H_{14}BrNO$ $[M]^+$: 303.0259, Found: 303.0258.

2-(3-Bromopropyl)pyrrolidine-1-carbaldehyde (2j)

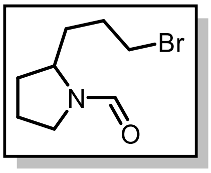

The reaction was performed at 60°C; Yellow liquid (22 mg, 50%); *Data recorded on a 1.3:1 mixture of rotamers*; **^{1}H NMR** (400 MHz, $CDCl_3$) δ 8.30 (s, 1H major), 8.27 (s, 1H minor), 4.09 – 4.01 (m, 1H minor), 3.89 – 3.80 (m, 1H major), 3.65 – 3.53 (m, 1H major + 1H minor), 3.49 – 3.32 (m, 3H major + 3H minor), 2.10 – 1.55 (m, 8H major + 8H minor); **^{13}C NMR** (100 MHz, $CDCl_3$) δ 161.6 (minor), 161.4 (major), 57.6 (major), 54.9 (minor), 46.8 (minor), 43.8 (major), 34.5 (major), 33.5 (minor), 32.9 (major), 32.5 (minor), 30.5 (major), 30.4 (minor), 29.4 (minor), 29.1 (major), 23.7 (minor), 22.5 (major); **IR** (cm^{-1}) 2927, 2885, 1718, 1639, 1426, 1386, 1179, 1071; **High Resolution MS** (EI): Calculated for $C_8H_{14}BrNO$ $[M]^+$: 219.0259, Found: 219.0257.

2-(3-Bromopropyl)piperidine-1-carbaldehyde (2k)

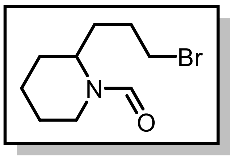

The reaction was performed at 60 ℃; Colourless liquid (28 mg, 60%); *Data recorded on a 1.2:1 mixture of rotamers*; **^{1}H NMR** (600 MHz, $CDCl_3$) δ 8.04 (s, 1H major), 8.02 (s, 1H minor), 4.57 – 4.48 (m, 1H major), 4.27 – 4.19 (m, 1H minor), 3.62 – 3.50 (m, 2H major + 2H minor), 3.47 – 3.39 (m, 1H minor), 3.37 (dd, *J* = 13.6, 4.7 Hz, 1H major), 3.14 (td, *J* = 13.2, 3.0 Hz, 1H major), 2.66 (td, *J* = 13.2, 2.9 Hz, 1H minor), 2.02 – 1.94 (m, 1H minor), 1.94 – 1.87 (m, 1H major), 1.80 – 1.55 (m, 7H major + 9H minor), 1.47 – 1.33 (m, 2H major); **^{13}C NMR** (150 MHz, $CDCl_3$) δ 161.6 (major), 161.1 (minor), 54.4 (minor), 46.7 (major), 45.1 (major), 44.7 (minor), 42.5 (major), 36.3 (minor), 30.3 (minor), 29.5 (minor), 29.2 (major), 28.6 (major), 27.5 (minor), 26.8 (major), 26.7 (major), 25.2 (minor), 20.1 (minor), 19.8 (major); **IR** (cm^{-1}) 2940, 2864, 1656, 1428, 1399, 1267, 1129, 1039, 666, 645; **High Resolution MS** (ESI): Calculated for $C_9H_{16}BrNONa$ $[M+Na]^+$: 256.0307, Found: 256.0304.

***N*-{[(1*S*,2*R*)-2-(Bromomethyl)cyclohexyl]methyl}-*N*-(4-methoxyphenyl)formamide (2l)**

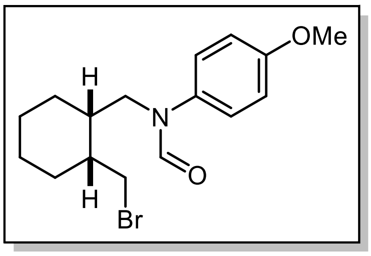

Yellow liquid (50 mg, 74%); *A 14:1 mixture of rotamers, Spectral data of major rotamer is reported*; **^{1}H NMR** (600 MHz, $CDCl_3$) δ 8.30 (s, 1H), 7.12 (d, *J* = 8.5 Hz, 2H), 6.93 (d, *J* = 8.5 Hz, 2H), 4.03 (dd, *J* = 14.0, 9.5 Hz, 1H), 3.82 (s, 3H), 3.56 (dd, *J* = 14.0, 6.0 Hz, 1H), 3.43 (app. t, *J* = 9.0 Hz, 1H), 3.37 (app. t, *J* = 9.0 Hz, 1H), 2.06 – 1.96 (m, 1H), 1.96 – 1.88 (m, 1H), 1.66 – 1.55 (m, 3H), 1.53 – 1.46 (m, 1H), 1.44 – 1.37 (m, 1H), 1.37 – 1.23 (m, 3H); **^{13}C NMR** (150 MHz, $CDCl_3$) δ 163.1, 158.7, 133.3, 126.2, 115.0, 55.7, 43.3, 40.7, 35.4 (2C), 27.6, 26.8, 23.7, 22.8; **IR** (cm^{-1}) 2921, 2856, 1666, 1510, 1249, 1033, 832, 570; **High Resolution MS** (EI): Calculated for $C_{16}H_{22}BrNO_2$ $[M]^+$: 339.0834, Found: 339.0833.

(*Z*)-*N*-(4-Bromobut-2-en-1-yl)-*N*-(4-methoxyphenyl)formamide (2m)

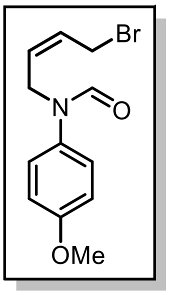

Brown liquid (49 mg, 86%); *A 17:1 mixture of rotamers, Spectral data of major rotamer is reported*; **^{1}H NMR** (400 MHz, $CDCl_3$) δ 8.27 (s, 1H), 7.09 (d, *J* = 9.0 Hz, 2H), 6.91 (d, *J* = 9.0 Hz, 2H), 5.86 (app. dtt, *J* = 10.0, 8.5, 1.5 Hz, 1H), 5.57 (dt, *J* = 10.0, 7.0 Hz, 1H), 4.44 (dd, *J* = 7.0, 1.5 Hz, 2H), 3.96 (d, *J* = 8.5 Hz, 2H), 3.81 (s, 3H); **^{13}C NMR** (100 MHz, $CDCl_3$) δ 162.1, 158.7, 133.3, 129.0, 128.8, 126.3, 114.8, 55.5, 41.8, 25.9; **IR** (cm^{-1}) 2922, 2859, 1665, 1509, 1246, 1208, 1033, 831; **High Resolution MS** (EI): Calculated for $C_{12}H_{14}BrNO_2$ $[M]^+$: 283.0208, Found: 283.0208.

***tert*-Butyl 4-[*N*-(4-bromobutyl)formamido]piperidine-1-carboxylate (2n)**

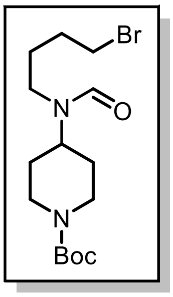

The reaction was performed at 60°C using TMSCF$_2$Br (34 µL, 0.22 mmol, 1.1 equiv.) and NH$_4$OAc (17 mg, 0.22 mmol, 1.1 equiv.); Colourless liquid (59 mg, 81%); *Data recorded on a 2:1 mixture of rotamers*; **^{1}H NMR** (400 MHz, CDCl$_3$) δ 8.17 (s, 1H major), 8.07 (s, 1H minor), 4.31 – 4.05 (m, 2H major + 3H minor), 3.41 (t, *J* = 6.5 Hz, 2H major), 3.39 (t, *J* = 6.5 Hz, 2H minor), 3.38 – 3.33 (m, 1H major), 3.25 – 3.17 (m, 2H major + 2H minor), 2.80 – 2.66 (m, 2H major + 2H minor), 1.89 – 1.81 (m, 2H major + 2H minor), 1.75 – 1.62 (m, 6H major + 6H minor), 1.45 (s, 9H major), 1.44 (s, 9H minor); **^{13}C NMR** (100 MHz, CDCl$_3$) δ 163.0 (minor), 162.3 (major), 154.5 (minor), 154.4 (major), 80.0 (major), 79.7 (minor), 56.7 (major), 50.9 (minor), 44.1 (minor), 43.2 (major + minor), 40.6 (major), 33.1 (major), 32.6 (minor), 31.7 (major), 30.2 (minor), 30.0 (major), 29.63 (minor), 29.57 (minor), 28.4 (minor), 28.3 (major), 27.6 (major); **IR** (cm^{-1}) 2930, 2860, 1667, 1424, 1364, 1165, 1129, 762, 732; **High Resolution MS** (EI): Calculated for $C_{15}H_{27}BrN_2O_3$ $[M]^+$: 362.1205, Found: 362.1207.

2-(3-Bromopropyl)-4-tosyl-3,4-dihydroquinoxaline-1(2*H*)-carbaldehyde (2o)

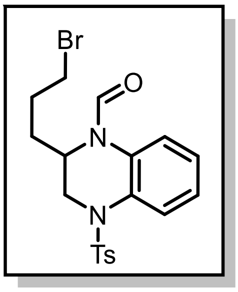

Colourless liquid (63 mg, 72%); **^{1}H NMR** (400 MHz, CDCl$_3$) δ 8.06 (s, 1H), 7.73 (d, *J* = 7.5 Hz, 1H), 7.51 (d, *J* = 8.0 Hz, 2H), 7.25 – 7.16 (m, 4H), 7.02 (d, *J* = 7.5 Hz, 1H), 4.54 (app. quint., *J* = 6.5 Hz, 1H), 4.11 (dd, *J* = 14.0, 6.5 Hz, 1H), 3.77 (dd, *J* = 14.0, 7.0 Hz, 1H), 3.33 (m, 2H), 2.38 (s, 3H), 1.86 – 1.74 (m, 2H), 1.66 – 1.57 (m, 2H); **^{13}C NMR** (100 MHz, CDCl$_3$) δ 160.6, 144.6, 136.2, 130.6, 130.4, 130.1, 126.9, 126.8, 126.3, 124.9, 119.7, 50.6, 49.3, 32.8, 30.4, 28.5, 21.7; **IR** (cm^{-1}) 2923, 2873, 1671, 1496, 1349, 1162, 1089, 674; **High Resolution MS** (EI): Calculated for $C_{19}H_{21}BrN_2O_3S$ $[M]^+$: 436.0456, Found: 436.0459.

***N*-(4-Bromo-2-phenylbutyl)-*N*-(4-methoxyphenyl)formamide (2p)**

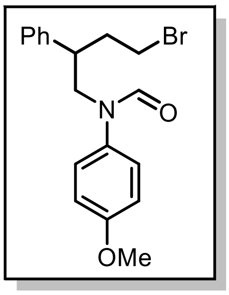

Brown liquid (69 mg, 95%, r.r. = 15:1); *Spectral data of major regioisomer is reported*; **^{1}H NMR** (400 MHz, CDCl$_3$) δ 8.15 (s, 1H), 7.32 – 7.18 (m, 3H), 7.13 – 7.08 (m, 2H), 6.92 – 6.82 (m, 4H), 4.13 (dd, *J* = 13.5, 8.0 Hz, 1H), 3.86 (dd, *J* = 13.5, 8.0 Hz, 1H), 3.79 (s, 3H), 3.26 (dd, *J* = 11.0, 6.5, 4.5 Hz, 1H), 3.11 – 3.04 (m, 1H), 2.98 (td, *J* = 9.5, 6.5 Hz, 1H), 2.21 – 2.06 (m, 2H); **^{13}C NMR** (100 MHz, CDCl$_3$) δ 162.9, 158.6, 140.0, 133.3, 128.6, 128.0, 127.2, 126.6, 114.6, 55.5, 49.7, 42.0, 36.4, 31.3; **IR** (cm^{-1}) 2969, 2931, 2859, 1664, 1423, 1365, 1165, 1128, 732; **High Resolution MS** (EI): Calculated for $C_{18}H_{20}BrNO_2$ $[M]^+$: 361.0677, Found: 361.0681.

The relative regiochemistry of the title compound was corroborated by ^{1}H NMR, NOESY and HMBC experiments (see Spectral Data).

***N*-{[1-(2-Bromoethyl)cyclohexyl]methyl}-*N*-phenylformamide (2q)**

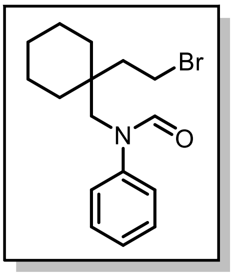

Colourless liquid (59 mg, 91%); *A 14:1 mixture of rotamers, Spectral data of major rotamer is reported*; **^{1}H NMR** (400 MHz, $CDCl_3$) δ 8.34 (s, 1H), 7.44 – 7.38 (m, 2H), 7.32 – 7.27 (m, 1H), 7.22 – 7.19 (m, 2H), 3.78 (s, 2H), 3.40 – 3.35 (m, 2H), 1.86 – 1.81 (m, 2H), 1.48 – 1.34 (m, 4H), 1.31 – 1.11 (m, 6H); **^{13}C NMR** (100 MHz, $CDCl_3$) δ 164.0, 142.5, 129.7, 126.9, 124.5, 50.8, 40.3, 38.2, 34.1, 28.6, 25.7, 21.2; **IR** (cm^{-1}) 2926, 2854, 1721, 1674, 1593, 1493, 1456, 1162, 914, 732; **High Resolution MS** (EI): Calculated for $C_{16}H_{22}BrNO$ $[M]^+$: 323.0885, Found: 323.0881.

The relative regiochemistry of the title compound was corroborated by ^{1}H NMR, ^{13}C NMR and HSQC experiments, in which the singlet CH_2 proton peak (3.78 ppm) and triplet CH_2 peak (3.80 ppm) correlate with the carbon signal at 50.8 ppm and 28.6 ppm, respectively. The former falls in a range of chemical shifts expected for amido alkanes, while the latter is one expected for benzyl bromides (see Spectral Data).

(*S*)-5-Bromo-2-(*N*-methylformamido)pentyl acetate (2r)

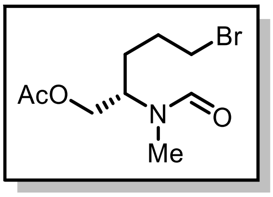

The reaction was performed at 25°C using $TMSCF_2Br$ (34 µL, 0.22 mmol, 1.1 equiv.) and NH_4OAc (17 mg, 0.22 mmol, 1.1 equiv.); Yellow liquid (32 mg, 60%); *Data recorded on a 2:1 mixture of rotamers*; **^{1}H NMR** (600 MHz, $CDCl_3$) δ 8.13 (s, 1H minor), 8.03 (s, 1H major), 4.74 – 4.59 (m, 1H minor), 4.27 – 4.00 (m, 2H major + 2H minor), 3.69 (tt, *J* = 9.5, 4.5 Hz, 1H major), 3.50 – 3.33 (m, 2H major + 2H minor), 2.86 (s, 3H minor), 2.77 (s, 3H major), 2.05 (s, 3H major), 2.05 (s, 3H minor), 1.91 – 1.78 (m, 2H major + 2H minor), 1.77 – 1.63 (m, 2H major + 2H minor); **^{13}C NMR** (150 MHz, $CDCl_3$) δ 170.8 (minor), 170.7 (major), 163.9 (minor), 163.3 (major), 63.2 (minor), 63.0 (major), 56.7 (major), 49.4 (minor), 33.2 (minor), 32.7 (major), 30.1 (minor), 28.89 (minor), 28.88 (major), 26.8 (major), 26.1 (minor), 24.9 (major), 20.9 (minor), 20.8 (major); **IR** (cm^{-1}) 2963, 2867, 1737, 1665, 1228, 1043, 734; **High Resolution MS** (EI): Calculated for $C_9H_{16}BrNO_3$ $[M]^+$: 265.0314, Found: 265.0310; **Specific Rotation** $[\alpha]_D^{20}$ –14.0±1.4 (*c* 0.5, CH_3Cl); **HPLC Analysis** CHIRALPAK OJH, 32 °C, hexane:iPrOH = 80:20, 1.0 mL/min, 220 nm, t_{R1} [major enantiomer (minor rotamer)] = 10.20 min, t_{R2} [minor enantiomer (minor rotamer)] = 11.60 min, t_{R3} [major enantiomer (major rotamer)] = 13.34 min, t_{R4} [minor enantiomer (major rotamer)] = 14.80 min, major enantiomer: minor enantiomer > 99% ee.

The relative regiochemistry of the title compound was corroborated by ^{1}H NMR, NOESY and HMBC experiments (see Spectral Data);

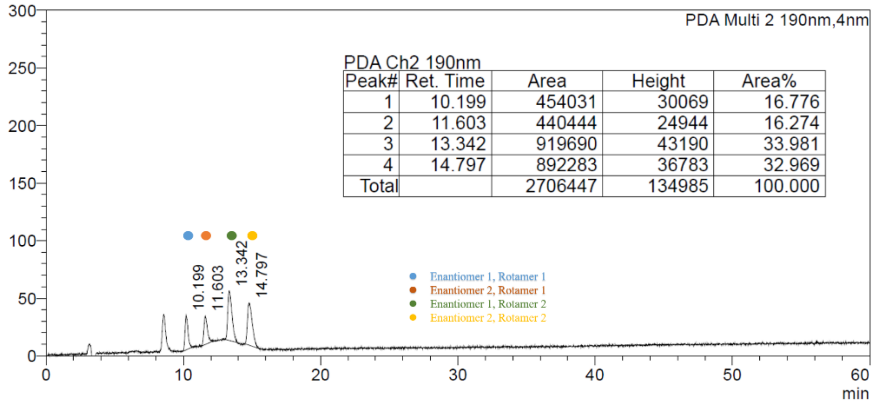

PDA Ch2 190nm

Peak#	Ret. Time	Area	Height	Area%
1	10.199	454031	30069	16.776
2	11.603	440444	24944	16.274
3	13.342	919690	43190	33.981
4	14.797	892283	36783	32.969
Total		2706447	134985	100.000

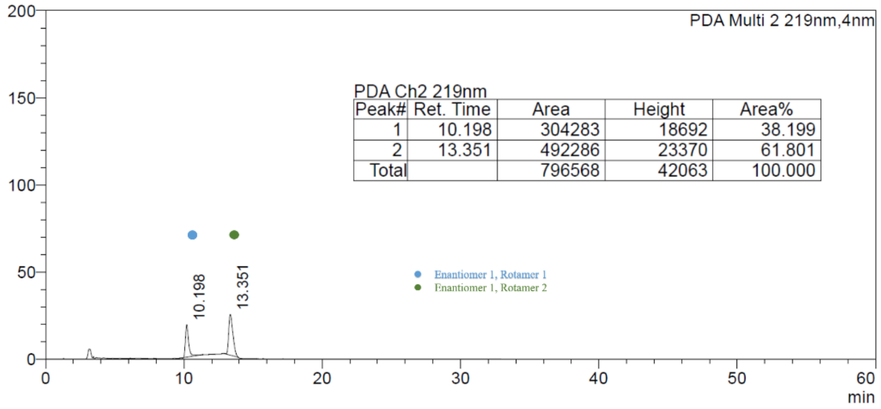

PDA Ch2 219nm

Peak#	Ret. Time	Area	Height	Area%
1	10.198	304283	18692	38.199
2	13.351	492286	23370	61.801
Total		796568	42063	100.000

Supplementary Figure 1. HPLC traces of racemic and scalemic compounds of **2r**

***N*-(4-Chlorobutyl)-*N*-phenylformamide (2a-Cl)**

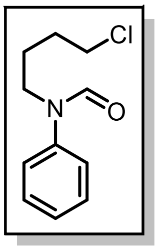

The reaction was performed using TMSCF$_2$Cl (126 mg, 0.8 mmol), KF (46.4 mg, 0.8 mmol), 1,2-DCE (0.5 mL) and H$_2$O (0.05 mL); Colourless liquid (33 mg, 78%); *A 19:1 mixture of rotamers, Spectral data of major rotamer is reported*; **^{1}H NMR** (400 MHz, CDCl$_3$) δ 8.37 (s, 1H), 7.45 – 7.39 (m, 2H), 7.33 – 7.29 (m, 1H), 7.19 – 7.15 (m, 2H), 3.86 (t, *J* = 7.0 Hz, 2H), 3.52 (t, *J* = 6.5 Hz, 2H), 1.81 – 1.66 (m, 4H); **^{13}C NMR** (100 MHz, CDCl$_3$) δ 162.4, 140.6, 129.7, 127.0, 124.2, 44.4, 43.9, 29.6, 24.9; **IR** (cm^{-1}) 2940, 2870, 1668, 1594, 1495, 1357, 1263, 729, 696; **High Resolution MS** (EI): Calculated for $C_{11}H_{14}ClNO$ [M]$^+$: 211.0764, Found: 211.0765.

***N*-(4-Chlorobutyl)-*N*-ethylformamide (2b-Cl)**

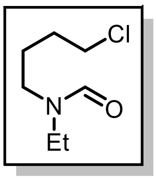

The reaction was performed at 60°C using TMSCF$_2$Cl (126 mg, 0.8 mmol); Yellow liquid (27 mg, 82%); *Data recorded on a 1.3:1 mixture of rotamers*; **^{1}H NMR** (400 MHz, CDCl$_3$) δ 8.07 (s, 1H major), 8.01 (s, 1H minor), 3.56 (t, *J* = 6.5 Hz, 2H major), 3.55 (t, *J* = 6.5 Hz, 2H minor), 3.35 (q, *J* = 7.0 Hz, 2H minor), 3.34 (t, *J* = 7.0 Hz, 2H major), 3.28 (q, *J* = 7.0 Hz, 2H major), 3.24 (t, *J* = 7.0 Hz, 2H minor), 1.81 – 1.65 (m, 4H major + 4H minor), 1.19 (t, *J* = 7.0 Hz, 3H major), 1.13 (t, *J* = 7.0 Hz, 3H minor); **^{13}C NMR** (100 MHz, CDCl$_3$) δ 162.6 (major), 162.4 (minor), 46.4 (minor), 44.5 (major), 44.3 (minor), 42.0 (major), 40.7 (major), 36.9 (minor), 29.6 (major), 29.3 (minor), 26.0 (minor), 24.6 (major), 14.8 (major), 12.6 (minor); **IR** (cm^{-1}) 2974, 2973, 2871, 1656, 1430, 1398, 1257, 1103, 730, 645; **High Resolution MS** (EI): Calculated for $C_7H_{14}ClNO$ [M]$^+$: 163.0764, Found: 163.0766.

***N*-(4-Iodobutyl)-*N*-(4-methoxyphenyl)formamide (2d-I)**

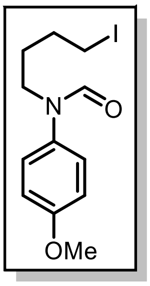

The reaction was performed on a 0.1 mmol scale, using TMSCF$_2$I (100 mg, 0.4 mmol), NH$_4$OAc (30.9 mg, 0.4 mmol) and 1,2-DCE (0.5 mL); Brown liquid (17 mg, 51%); *A 11:1 mixture of rotamers, Spectral data of major rotamer is reported*; **^{1}H NMR** (600 MHz, CDCl$_3$) δ 8.26 (s, 1H), 7.09 (d, *J* = 9.0 Hz, 2H), 6.93 (d, *J* = 9.0 Hz, 2H), 3.82 (s, 3H), 3.77 (t, *J* = 7.0 Hz, 2H), 3.17 (t, *J* = 7.0 Hz, 2H), 1.84 – 1.79 (m, 2H), 1.65 – 1.60 (m, 2H); **^{13}C NMR** (150 MHz, CDCl$_3$) δ 162.5, 158.7, 133.4, 126.4, 114.9, 55.5, 44.0, 30.4, 28.4, 5.9; **IR** (cm^{-1}) 2931, 2834, 1668, 1511, 1247, 1029, 835; **High Resolution MS** (EI): Calculated for $C_{12}H_{16}INO_2$ [M]$^+$: 333.0226, Found: 333.0227.

4-(*N*-Phenylformamido)butyl acetate (2a-OAc)

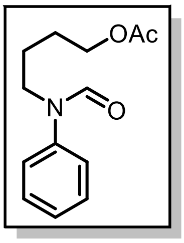

The reaction was performed using $TMSCF_2Cl$ (126 mg, 0.8 mmol) NH_4OAc (185 mg, 2.4 mmol); Colourless liquid (33 mg, 70%); *A 11:1 mixture of rotamers, Spectral data of major rotamer is reported*; **^{1}H NMR** (400 MHz, $CDCl_3$) δ 8.37 (s, 1H), 7.42 (t, *J* = 7.5 Hz, 2H), 7.30 (t, *J* = 7.5 Hz, 1H), 7.16 (d, *J* = 7.5 Hz, 2H), 4.02 (t, *J* = 6.0 Hz, 2H), 3.84 (t, *J* = 7.0 Hz, 2H), 2.00 (s, 3H), 1.66 – 1.56 (m, 4H); **^{13}C NMR** (100 MHz, $CDCl_3$) δ 171.0, 162.4, 140.7, 129.7, 127.0, 124.2, 63.8, 44.4, 25.9, 24.2, 20.9; **IR** (cm^{-1}) 2941, 2870, 1733, 1672, 1595, 1496, 1364, 1239, 1036, 733, 699; **High Resolution MS** (EI): Calculated for $C_{13}H_{17}NO_3$ $[M]^+$: 235.1208, Found: 235.1207.

***N*-(4-Bromobutyl)-*N*-(3,4-dimethoxyphenethyl)formamide (2s)**

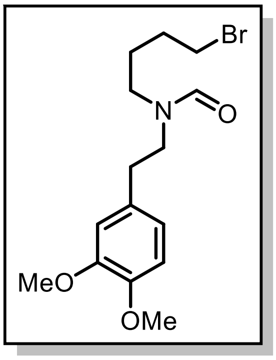

The reaction was performed on a 4.5 mmol scale, using ***1s*** *(1.06 g, 4.5 mmol), $TMSCF_2Br$ (0.77 mL, 4.95 mmol, 1.1 equiv.), NH_4OAc (382 mg, 4.95 mmol, 1.1 equiv.) and 1,2-DCE (10 mL)*; Colourless liquid (1.48 g, 96%); *Data recorded on a 1.1:1 mixture of rotamers;* **^{1}H NMR** (400 MHz, $CDCl_3$) δ 8.03 (s, 1H minor), 7.82 (s, 1H major), 6.79 – 6.61 (m, 3H major + 3H minor), 3.85 (s, 3H minor), 3.84 (s, 3H major), 3.83 (s, 3H major + 3H minor), 3.51 – 3.47 (m, 2H minor), 3.41 (app. t, *J* = 6.5 Hz, 4H major), 3.36 (t, *J* = 6.5 Hz, 2H minor), 3.34 (t, *J* = 7.0 Hz, 2H major), 3.12 (t, *J* = 7.0 Hz, 2H minor), 2.81 – 2.74 (m, 2H major + 2H minor), 1.87 – 1.61 (m, 4H major + 4H minor); **^{13}C NMR** (100 MHz, $CDCl_3$) δ 162.8 (major), 162.5 (minor), 149.0 (major), 148.9 (minor), 147.8 (major), 147.6 (minor), 131.1(minor), 130.0 (major), 120.7 (major), 120.6 (minor), 111.82 (minor), 111.79 (major), 111.4 (major), 111.2 (minor), 55.83 (minor), 55.81 (minor), 55.80 (major), 55.79 (major), 48.9 (major), 47.0 (minor), 44.2 (minor), 41.1 (major), 34.9 (major), 33.2 (major), 33.1 (minor), 32.7 (minor), 29.6 (major), 29.3 (minor), 27.1 (minor), 25.8 (major); **IR** (cm^{-1}) 2934, 2863, 1664, 1514, 1440, 1261, 1236, 1155, 1141, 1208, 732; **High Resolution MS** (EI): Calculated for $C_{15}H_{22}BrNO_3$ $[EI]^+$: 343.0783, Found: 343.0786.

***N*-(5-Bromopentyl)-*N*-phenylformamide (4a)**

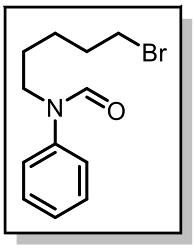

The reaction was performed at 60°C; Colourless liquid (43 mg, 80%); *A 10:1 mixture of rotamers, Spectral data of major rotamer is reported*; **^{1}H NMR** (600 MHz, $CDCl_3$) δ 8.36 (s, 1H), 7.41 (t, *J* = 7.5 Hz, 2H), 7.30 (t, *J* = 7.5 Hz, 1H), 7.16 (d, *J* = 7.5 Hz, 2H), 3.82 (t, *J* = 7.5 Hz, 2H), 3.35 (t, *J* = 6.5 Hz, 2H), 1.83 (app. quint., *J* = 7.0 Hz, 2H), 1.56 (app. quint., *J* = 7.5 Hz, 2H), 1.44 (app. quint., *J* = 7.5 Hz, 2H); **^{13}C NMR** (150 MHz, $CDCl_3$) δ 162.3, 140.7, 129.7, 126.9, 124.2, 44.6, 33.4, 32.1, 26.7, 25.2; **IR** (cm^{-1}) 2934, 2861, 1671, 1594, 1495, 1357, 1267, 911, 765, 731, 696; **High Resolution MS** (EI): Calculated for $C_{12}H_{16}BrNO$ $[M]^+$: 269.0415, Found: 269.0415.

***N*-(5-Bromopentyl)-*N*-ethylformamide (4b)**

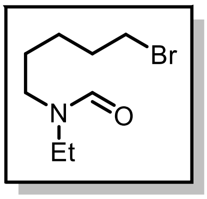

The reaction was performed at 80 ℃; Colourless liquid (7.8 mg, 18%); *Data recorded on a 1.1:1 mixture of rotamers*; **^{1}H NMR** (600 MHz, $CDCl_3$) δ 8.08 (s, 1H major), 8.03 (s, 1H minor), 3.41 (t, *J* = 6.7 Hz, 2H major + 2H minor), 3.36 (q, *J* = 7.2 Hz, 2H minor), 3.33 – 3.30 (m, 2H major), 3.28 (q, *J* = 7.2 Hz, 2H major), 3.22 (t, *J* = 7.1 Hz, 2H minor), 1.93 – 1.85 (m, 2H major + 2H minor), 1.63 – 1.53 (m, 2H major + 2H minor), 1.49 – 1.39 (m, 2H major + 2H minor), 1.19 (t, *J* = 7.2 Hz, 3H major), 1.14 (t, *J* = 7.2 Hz, 3H minor); **^{13}C NMR** (100 MHz, $CDCl_3$) δ 162.8 (major), 162.7 (minor), 47.2 (minor), 42.4 (major), 41.8 (major), 37.2 (minor), 33.8 (major), 33.4 (minor), 32.5 (major), 32.4 (minor), 28.2 (major), 26.8 (minor), 25.7 (major), 25.3 (minor), 15.1 (major), 12.9 (minor); **IR** (cm^{-1}) 2956, 2868, 1667, 1429, 1399, 1264, 733, 704; **High Resolution MS** (ESI): Calculated for $C_8H_{16}BrNONa$ $[M+Na]^+$: 244.0307, Found: 244.0315.

***N*-(5-Bromopentyl)-*N*-isobutylformamide (4c)**

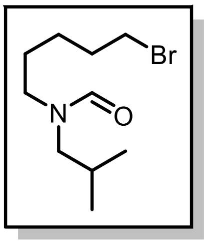

The reaction was performed at 80 ℃; Colourless liquid (7.5 mg, 15%); *Data recorded on a 1.7:1 mixture of rotamers*; **^{1}H NMR** (400 MHz, $CDCl_3$) δ 8.11 (s, 1H minor), 8.01 (s, 1H major), 3.53 (t, *J* = 6.7 Hz, 2H minor), 3.40 (t, *J* = 6.7 Hz, 2H major), 3.33 – 3.25 (m, 2H major), 3.22 (t, *J* = 7.1 Hz, 2H minor), 3.14 (d, *J* = 7.6 Hz, 2H minor), 2.98 (d, *J* = 7.4 Hz, 2H major), 2.00 – 1.75 (m, 3H major + 3H minor), 1.63 – 1.52 (m, 2H major + 2H minor), 1.50 – 1.38 (m, 2H major + 2H minor), 0.93 – 0.85 (m, 6H major + 6H minor); **^{13}C NMR** (100 MHz, $CDCl_3$) δ 163.3 (major), 163.2 (minor), 55.5 (major), 49.3 (minor), 47.7 (minor), 45.1 (minor), 42.6 (major), 33.8 (major), 32.5 (major), 32.4 (minor), 26.9 (major), 26.7 (minor), 26.6 (major), 26.5 (minor), 25.7 (major), 25.4 (minor), 20.3 (2C minor), 20.0 (2C major); **IR** (cm^{-1}) 2959, 2934, 2869, 1666, 1428, 1265, 1119, 732, 701; **High Resolution MS** (ESI): Calculated for $C_{10}H_{20}BrNONa$ $[M+Na]^+$: 272.0620, Found: 272.0651.

N-(5-Bromopentyl)-*N*-butylformamide (4d)

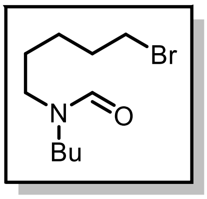

The reaction was performed at 80 ℃ for 36 h; Colourless liquid (26 mg, 30%); *Data recorded on a 1.2:1 mixture of rotamers*; **^{1}H NMR** (600 MHz, $CDCl_3$) δ 8.05 (s, 1H minor), 8.05 (s, 1H major), 3.41 (t, *J* = 6.7 Hz, 2H major + 2H minor), 3.32 – 3.26 (m, 2H major + 2H minor), 3.24 – 3.17 (m, 2H major + 2H minor), 1.92 – 1.84 (m, 2H major + 2H minor), 1.59 – 1.54 (m, 2H major + 2H minor), 1.54 – 1.49 (m, 2H major + 2H minor), 1.48 – 1.40 (m, 2H major + 2H minor), 1.36 – 1.27 (m, 2H major + 2H minor), 0.97 – 0.88 (m, 3H major + 3H minor); **^{13}C NMR** (100 MHz, $CDCl_3$) δ 163.0 (major), 162.9 (minor), 47.5 (major), 47.5 (minor), 42.1 (minor), 42.1 (major), 33.8 (major), 33.4 (minor), 32.5 (major), 32.4 (minor), 31.0 (major), 29.6 (minor), 28.1 (minor), 26.7 (major), 25.7 (major), 25.3 (minor), 20.4 (minor), 19.9 (major), 14.0 (minor), 13.9 (major); **IR** (cm^{-1}) 2956, 2929, 2861, 1666, 1427, 1283, 800, 758; **High Resolution MS** (ESI): Calculated for $C_{10}H_{20}BrNONa$ $[M+Na]^+$: 272.0620, Found: 272.0624.

N-(5-Bromopentyl)-*N*-isopentylformamide (4e)

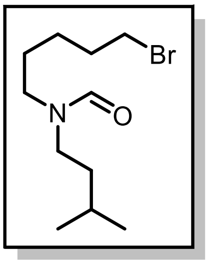

The reaction was performed at 80 ℃ for 36 h; Colourless liquid (26 mg, 50%); *Data recorded on a 1.2:1 mixture of rotamers*; **^{1}H NMR** (600 MHz, $CDCl_3$) δ 8.05 (s, 1H major), 8.03 (s, 1H minor), 3.40 (t, *J* = 6.6 Hz, 2H major + 2H minor), 3.34 – 3.26 (m, 2H major + 2H minor), 3.21 (t, *J* = 7.3 Hz, 2H major + 2H minor), 1.88 (m, 2H major + 2H minor), 1.56 (m, 3H major + 3H minor), 1.43 (m, 4H major + 4H minor), 0.92 (d, *J* = 6.6 Hz, 6H major + 6H minor); **^{13}C NMR** (150 MHz, $CDCl_3$) 162.9 (major), 162.8 (minor), 47.3 (minor), 45.9 (major), 42.0 (major), 40.7 (minor), 37.9 (major), 36.2 (minor), 33.8 (major), 33.4 (minor), 32.5 (major), 32.4 (minor), 28.0 (minor), 26.6 (minor), 26.3 (major), 25.6 (minor), 25.5 (major), 25.3 (major), 22.7 (2C minor), 22.5 (2C major); **IR** (cm^{-1}) 2958, 2869, 1666, 1458, 1264, 736, 705; **High Resolution MS** (ESI): Calculated for $C_{11}H_{22}BrNONa$ $[M+Na]^+$: 286.0777, Found: 286.0783.

N-(5-Bromopentyl)-N-(3,3-dimethylbutyl)formamide (4f)

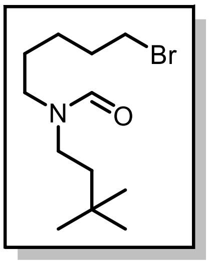

The reaction was performed at 80 ℃ for 36 h; Colourless liquid (44 mg, 79%); *Data recorded on a 1:1 mixture of rotamers*; **^{1}H NMR** (600 MHz, $CDCl_3$) δ 8.05 (s, 1H), 8.01 (s, 1H), 3.53 (td, *J* = 6.5, 2.8 Hz, 2H + 1H), 3.40 (t, *J* = 6.6 Hz, 1H), 3.31 – 3.25 (m, 2H + 2H), 3.22 – 3.16 (m, 2H + 2H), 1.84 – 1.73 (m, 2H + 2H), 1.60 – 1.51 (m, 2H + 2H), 1.47 – 1.36 (m, 4H + 4H), 0.93 (s, 9H + 9H); **^{13}C NMR** (150 MHz, $CDCl_3$) δ 162.9, 162.6, 47.4, 45.1, 44.8, 44.2, 43.2, 42.1, 40.7, 39.0, 32.3, 32.2, 30.0, 29.9, 29.45 (3C), 29.43 (3C), 28.2, 26.8, 24.3, 24.0; **IR** (cm^{-1}) 2956, 2866, 1669, 1463, 1365, 1141, 1125, 950, 869, 785, 642; **High Resolution MS** (ESI): Calculated for $C_{12}H_{24}BrNONa$ $[M+Na]^+$: 300.0933, Found: 300.0929.

Ethyl 4-bromo-2-{2-[N-(4-methoxyphenyl)formamido]ethyl}butanoate (4g)

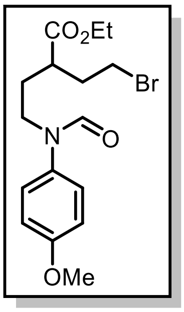

The reaction was performed at 60°C; Yellow liquid (63 mg, 85%); *A 9:1 mixture of rotamers, Spectral data of major rotamer is reported*; **^{1}H NMR** (600 MHz, $CDCl_3$) δ 8.25 (s, 1H), 7.08 (d, *J* = 8.0 Hz, 2H), 6.92 (d, *J* = 8.0 Hz, 2H), 4.13 (q, *J* = 7.0 Hz, 2H), 3.86 – 3.77 (m, 1H), 3.81 (s, 3H), 3.74 – 3.69 (m, 1H), 3.43 – 3.36 (m, 1H), 3.36 – 3.29 (m, 1H), 2.62 – 2.58 (m, 1H), 2.24 – 2.16 (m, 1H), 2.03 – 1.93 (m, 1H), 1.91 – 1.80 (m, 1H), 1.76 – 1.67 (m, 1H), 1.23 (t, *J* = 7.0 Hz, 3H); **^{13}C NMR** (150 MHz, $CDCl_3$) δ 174.4, 162.6, 158.8, 133.6, 126.4, 115.0, 61.0, 55.7, 43.3, 41.5, 34.9, 30.7, 29.5, 14.3; **IR** (cm^{-1}) 2947, 1730, 1670, 1595, 1436, 1358, 1265, 764, 699; **High Resolution MS** (EI): Calculated for $C_{16}H_{22}BrNO_4$ $[M]^+$: 371.0732, Found: 371.0730.

Methyl 5-bromo-3-[2-(N-phenylformamido)ethyl]pentanoate (4h)

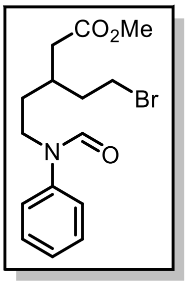

The reaction was performed at 60°C; Yellow liquid (68 mg, 80%); **^{1}H NMR** (400 MHz, $CDCl_3$) δ 8.36 (s, 1H), 7.42 (t, *J* = 7.5 Hz, 2H), 7.30 (t, *J* = 7.5 Hz, 1H), 7.17 (d, *J* = 7.5 Hz, 2H), 3.97 – 3.87 (m, 1H), 3.87 – 3.77 (m, 1H), 3.62 (s, 3H), 3.37 (t, *J* = 7.0 Hz, 2H), 2.35 (d, *J* = 6.5 Hz, 2H), 2.07 (app. quint., *J* = 6.5 Hz, 1H), 1.97 – 1.80 (m, 2H), 1.66 – 1.48 (m, 2H); **^{13}C NMR** (150 MHz, $CDCl_3$) δ 172.7, 162.5, 140.7, 129.9, 127.2, 124.3, 51.7, 42.4, 37.6, 36.8, 31.7, 31.1, 30.9; **IR** (cm^{-1}) 2947, 2930, 2872, 1731, 1671, 1595, 1496, 1358, 1206, 699; **High Resolution MS** (EI): Calculated for $C_{15}H_{20}BrNO_3$ $[M]^+$: 341.0627, Found: 341.0627.

***N*-{2-[1-(2-Bromoethyl)cyclopentyl]ethyl}-*N*-phenylformamide (4i)**

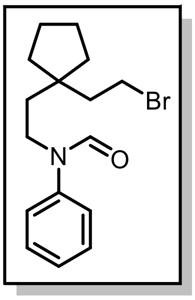

The reaction was performed at 60°C; Colourless liquid (64 mg, 99%); **^{1}H NMR** (400 MHz, $CDCl_3$) δ 8.35 (s, 1H), 7.45 – 7.40 (m, 2H), 7.33 – 7.29 (m, 1H), 7.17 – 7.14 (m, 2H), 3.81 – 3.77 (m, 2H), 3.37 – 3.33 (m, 2H), 1.96 – 1.92 (m, 2H), 1.61 – 1.57 (m, 4H), 1.55 – 1.51 (m, 2H), 1.46 – 1.40 (m, 4H); **^{13}C NMR** (150 MHz, $CDCl_3$) δ 162.1, 141.0, 129.8, 126.9, 123.9, 45.1, 42.2, 42.0, 37.5, 35.4, 29.2, 24.5; **IR** (cm^{-1}) 2943, 2863, 1667, 1594, 1495, 1357, 1264, 732, 698; **High Resolution MS** (EI): Calculated for $C_{16}H_{22}BrNO$ $[M]^+$: 323.0885, Found: 323.0887.

***N*-[2-(2-Bromoethoxy)ethyl]-*N*-phenylformamide (4j)**

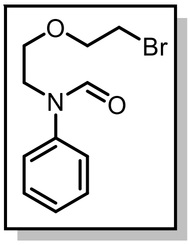

The reaction was performed at 40°C; Colourless liquid (49 mg, 90%); *A 10:1 mixture of rotamers, Spectral data of major rotamer is reported*; **^{1}H NMR** (400 MHz, $CDCl_3$) δ 8.35 (s, 1H), 7.34 (t, *J* = 7.5 Hz, 2H), 7.25 – 7.20 (m, 3H), 3.92 (t, *J* = 5.5 Hz, 2H), 3.67 (t, *J* = 6.0 Hz, 2H), 3.63 (t, *J* = 5.5 Hz, 2H), 3.32 (t, *J* = 6.0 Hz, 2H); **^{13}C NMR** (100 MHz, $CDCl_3$) δ 162.6, 141.3, 129.5, 126.9, 124.5, 70.7, 67.7, 45.6, 30.3; **IR** (cm^{-1}) 2922, 2869, 1670, 1595, 1495, 1342, 1155, 1091, 761, 732, 698; **High Resolution MS** (EI): Calculated for $C_{11}H_{14}BrNO_2$ $[M]^+$: 271.0208, Found: 271.0207.

***N*-(2-(2-Bromoethoxy)ethyl)-*N*-(3,3-dimethylbutyl)formamide (4k)**

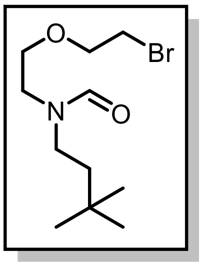

The reaction was performed at 80°C for 36 h; Colourless liquid (50 mg, 90%); *Data recorded on a 1.2:1 mixture of rotamers*; **^{1}H NMR** (400 MHz, $CDCl_3$) δ 8.06 (s, 1H major), 8.03 (s, 1H minor), 3.79 – 3.72 (m, 2H major + 1H minor), 3.72 – 3.66 (m, 1H minor), 3.65 – 3.54 (m, 2H major + 2H minor), 3.51 – 3.47 (m, 2H major), 3.46 – 3.41 (m, 2H major + 2H minor), 3.40 – 3.30 (m, 4H major + 2H minor), 1.51 – 1.36 (m, 2H major + 2H minor), 0.93 (s, 9H major + 9H minor); **^{13}C NMR** (100 MHz, $CDCl_3$) δ 163.3 (minor), 163.1 (major), 71.3 (minor), 71.0 (major), 69.32 (major), 69.28 (minor), 47.4 (major), 45.5 (minor), 43.1 (major), 42.4 (major), 40.8 (minor), 39.8 (minor), 30.7 (major), 30.2 (minor), 30.0 (minor), 29.9 (major), 29.48 (3C, major), 29.45 (3C, minor); **IR** (cm^{-1}) 2956, 2866, 1665, 1429, 1397, 1366, 1120, 910, 733; **High Resolution MS** (ESI): Calculated for $C_{11}H_{22}BrNO_2Na$ $[M+Na]^+$: 302.0726, Found: 302.0728.

***N*-(2-{[*N*-(2-Bromoethyl)-4-methylphenyl]sulfonamide}ethyl)-*N*-phenylformamide (4l)**

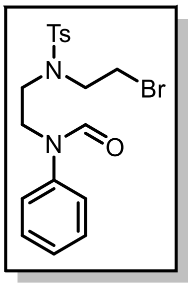

The reaction was performed at 60°C; Yellow liquid (65 mg, 76%); **^{1}H NMR** (600 MHz, $CDCl_3$) δ 8.40 (s, 1H), 7.64 (d, *J* = 8.0 Hz, 2H), 7.44 (t, *J* = 7.5 Hz, 2H), 7.32 (t, *J* = 7.5 Hz, 1H), 7.29 (d, *J* = 8.0 Hz, 2H), 7.20 (d, *J* = 7.5 Hz, 2H), 4.01 – 3.98 (m, 2H), 3.49 – 3.46 (m, 2H), 3.44 – 3.42 (m, 2H), 3.36 – 3.34 (m, 2H), 2.42 (s, 3H); **^{13}C NMR** (150 MHz, $CDCl_3$) δ 162.5, 143.9, 140.7, 135.6, 129.9, 129.85, 127.2, 127.1, 123.6, 51.0, 46.8, 45.2, 29.0, 21.5; **IR** (cm^{-1}) 2891, 1664, 1595, 1495, 1342, 1157, 1091, 951, 815, 734; **High Resolution MS** (EI): Calculated for $C_{18}H_{21}BrN_2O_3S$ $[M]^+$: 424.0456, Found: 424.0460.

***N*–[2-(Bromomethyl)phenethyl]-*N*-phenylformamide (4m)**

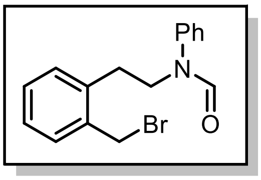

Colourless liquid (59 mg, 93%, r.r. = 12:1); *Spectral data of major regioisomer is reported*; **^{1}H NMR** (600 MHz, $CDCl_3$) δ 8.42 (s, 1H), 7.42 (t, *J* = 7.5 Hz, 2H), 7.35 – 7.30 (m, 2H), 7.27 – 7.19 (m, 3H), 7.16 (d, *J* = 8.0 Hz, 2H), 4.54 (s, 2H), 4.08 – 4.05 (m, 2H), 3.04 – 3.01 (m, 2H); **^{13}C NMR** (150 MHz, $CDCl_3$) δ 162.3, 140.8, 137.1, 136.1, 130.8, 130.3, 129.7, 129.1, 127.3, 127.0, 124.1, 46.2, 31.3, 30.5; **IR** (cm^{-1}) 2914, 2873, 1719, 1594, 1495, 1355, 1176, 732; **High Resolution MS** (EI): Calculated for $C_{16}H_{16}BrNO$ $[M]^+$: 317.0415, Found: 317.0413.

The relative regiochemistry of the title compound was corroborated by ^{1}H NMR, ^{13}C NMR and HSQC experiments, in which the singlet CH_2 proton peak correlates with the carbon signal at 31.3 ppm. This chemical shift value falls in a range expected for benzyl bromides (see Spectral Data).

***trans*-*N*-{[2-(2-Bromoethyl)cyclohexyl]methyl}-*N*-(4-methoxyphenyl)formamide (4n)**

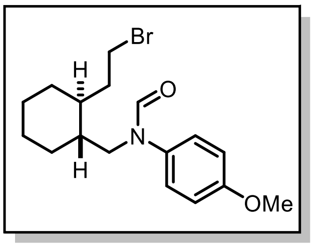

*The reaction was performed on a 2.0 mmol scale, using **3n** (491 mg, 2.0 mmol), $TMSCF_2Br$ (1.24 mL, 8.0 mmol, 4.0 equiv.), NH_4OAc (671 mg, 8.0 mmol, 4.0 equiv.) and 1,2-DCE (5 mL)*; Yellow liquid (624 mg, 88%); *A 9:1 mixture of rotamers, Spectral data of the major rotamer is reported:* **^{1}H NMR** (400 MHz, $CDCl_3$) δ 8.30 (s, 1H), 7.09 (d, *J* = 9.0 Hz, 2H), 6.92 (d, *J* = 9.0 Hz, 2H), 3.82 (s, 3H), 3.81 – 3.76 (m, 2H), 3.44 – 3.37 (m, 1H), 3.32 – 3.25 (m, 1H), 2.12 – 2.03 (m, 1H), 1.80 – 1.56 (m, 5H), 1.39 – 1.30 (m, 2H), 1.25 – 0.89 (m, 4H); **^{13}C NMR** (100 MHz, $CDCl_3$) δ 163.2, 158.5, 134.2, 126.0, 115.0, 55.6, 48.3, 39.2, 38.4, 36.6, 31.6, 30.2, 29.4, 25.1, 25.0; **IR** (cm^{-1}) 2932, 2856, 1665, 1512, 1248, 907, 730; **High Resolution MS** (ESI): Calculated for $C_{17}H_{24}BrNO_2Na$ $[M+Na]^+$: 376.0883, Found: 376.0884.

The relative regiochemistry was corroborated by ^{1}H NMR, ^{13}C NMR, HMBC, HSQC and NOESY experiments. In HMBC, the formyl CH proton peak correlates with a carbon signal at 48.3 ppm. The

proton peaks corresponding to this carbon was assigned by HSQC, which was then found to show a correlation with a C–H at tertiary carbon in NOESY.

8-(3-Bromopropyl)-3,4-dihydroquinoline-1(2*H*)-carbaldehyde (4o)

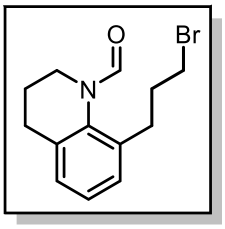

The reaction was performed at 40°C; Yellow liquid (40 mg, 71%); *Data recorded on a 5:1 mixture of rotamers*; **^{1}H NMR** (600 MHz, $CDCl_3$) δ 8.29 (s, 1H major), 8.28 (s, 1H minor), 7.17 – 7.12 (m, 2H major + 2H minor), 7.05 (d, *J* = 7.5 Hz, 1H major + 1H minor), 3.79 (t, *J* = 7.0 Hz, 2H major + 2H minor), 3.34 (t, *J* = 6.5 Hz, 2H major + 2H minor), 2.82 (t, *J* = 7.5 Hz, 2H major + 2H minor), 2.67 (t, *J* = 6.5 Hz, 2H major + 2H minor), 2.15 – 2.10 (m, 2H major + 2H minor), 1.96 (app. quint., *J* = 6.5 Hz, 2H major + 2H minor); **^{13}C NMR** (150 MHz, $CDCl_3$) δ 163.3 (major), 161.3 (minor), 136.5 (major), 136.4 (minor), 135.7 (major), 135.4 (minor), 134.3 (minor), 133.2 (major), 128.4 (major), 127.4 (minor), 126.9 (minor), 126.4 (major), 126.2 (minor), 126.1 (major), 44.6 (minor), 40.0 (major), 33.5 (minor), 33.4 (major), 32.8 (minor), 32.5 (major), 30.2 (minor), 29.3 (major), 27.0 (major), 26.0 (minor), 24.8 (minor), 23.6 (major); **IR** (cm^{-1}) 2930, 2871, 1669, 1594, 1495, 1356, 1267, 1175, 763; **High Resolution MS** (EI): Calculated for $C_{13}H_{16}BrNO$ $[M]^+$: 281.0415, Found: 281.0412.

4-(2-Bromoethyl)piperidine-1-carbaldehyde (4p)

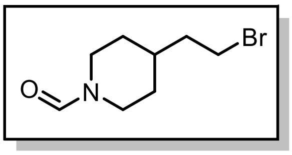

The reaction was performed at 80°C using $TMSCF_2Br$ (34 µL, 0.22 mmol, 1.1 equiv.) and NH_4OAc (17 mg, 0.22 mmol, 1.1 equiv.); Yellow liquid (33 mg, 75%); **^{1}H NMR** (400 MHz, $CDCl_3$) δ 8.01 (s, 1H), 4.44 – 4.39 (m, 1H), 3.65 – 3.60 (m, 1H), 3.59 (t, *J* = 6.5 Hz, 2H), 3.08 (td, *J* = 13.0, 3.0 Hz, 1H), 2.63 (td, *J* = 13.0, 3.0 Hz, 1H), 1.88 – 1.72 (m, 5H), 1.18 – 1.04 (m, 2H); **^{13}C NMR** (100 MHz, $CDCl_3$) δ 160.9, 46.1, 42.3, 39.9, 38.8, 33.6, 32.4, 31.0; **IR** (cm^{-1}) 2930, 2871, 1669, 1594, 1495, 1356, 1267, 1175, 763, 698; **High Resolution MS** (EI): Calculated for $C_8H_{14}BrNO$ $[M]^+$: 219.0259, Found: 219.0257.

Methyl (*Z*)-2-methylene-5-(*N*-methylformamido)pent-3-enoate (4q)

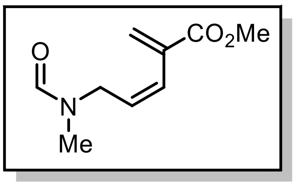

The reaction was performed at 25°C using TMSCF$_2$Br (34 µL, 0.22 mmol, 1.1 equiv.) and NH$_4$OAc (17 mg, 0.22 mmol, 1.1 equiv.); Colourless liquid (32 mg, 87%); *Data recorded on a 1.3:1 mixture of rotamers;* **^{1}H NMR** (400 MHz, DMSO-*d$_6$*) δ 8.03 (s, 1H major), 8.00 (s, 1H minor), 6.35 (s, 1H major + 1H minor), 6.30 (d, *J* = 12.0 Hz, 1H major), 6.29 (d, *J* = 12.0 Hz, 1H minor), 5.79 (s, 1H minor), 5.73 (s, 1H major), 5.70 (dt, *J* = 12.0, 6.0 Hz, 1H major), 5.60 (dt, *J* = 12.0, 6.0 Hz, 1H minor), 4.03 (d, *J* = 6.5 Hz, 2H major), 4.00 (d, *J* = 6.5 Hz, 2H minor), 3.72 (s, 3H major + 3H minor), 2.85 (s, 3H minor), 2.67 (s, 3H major); **^{13}C NMR** (100 MHz, DMSO-*d$_6$*) δ 166.1 (minor), 166.0 (major), 162.4 (major), 162.3 (minor), 134.7 (major + minor), 131.0 (major), 130.0 (minor), 128.7 (major), 128.6 (minor), 126.9 (major), 126.7 (minor), 52.14 (major), 52.11 (minor), 46.5 (major), 41.3 (minor), 33.7 (minor), 28.8 (major); **IR** (cm^{-1}) 2928, 1718, 1669, 1437, 1265, 907, 728, 703; **High Resolution MS** (EI): Calculated for $C_9H_{13}NO_3$ [M]$^+$: 183.0895, Found: 183.0897.

The relative regiochemistry and stereochemistry of the title compound was corroborated by ^{1}H NMR, NOESY and HMBC experiments (see Spectral Data).

***N*-(5-Bromo-5-phenylpentyl)-*N*-butylformamide (4r)**

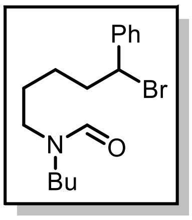

The reaction was performed at RT using TMSCF$_2$Br (34 µL, 0.22 mmol, 1.1 equiv.) and NH$_4$OAc (17 mg, 0.22 mmol, 1.1 equiv.); *Data recorded on a 1.1:1 mixture of rotamers;* Colourless liquid (57 mg, 87%); **^{1}H NMR** (400 MHz, CD_2Cl_2) δ 7.99 (s, 1H major), 7.97 (s, 1H minor), 7.43 – 7.26 (m, 5H major + 5H minor), 5.00 – 4.95 (m, 1H major + 1H minor), 3.26 – 3.21 (m, 2H major + 2H minor), 3.18 – 3.13 (m, 2H major + 2H minor), 2.36 – 2.26 (m, 2H minor), 2.22 – 2.11 (m, 2H major), 1.60 – 1.41 (m, 6H major + 4H minor), 1.34 – 1.24 (m, 2H major + 4H minor), 0.94 – 0.90 (m, 3H major + 3H minor); **^{13}C NMR** (100 MHz, CD_2Cl_2) δ 163.1 (major), 162.9 (minor), 142.8 (major), 142.6 (minor), 129.3 (minor), 129.2 (major), 129.0 (minor), 128.9 (major), 127.80 (major), 127.76 (minor), 56.2 (major), 55.9 (minor), 47.61 (major), 47.57 (minor), 42.2 (minor), 42.1 (major), 40.1 (minor), 40.0 (major), 31.3 (major), 29.9 (minor), 28.6 (minor), 27.1 (major), 26.1 (major), 25.8 (minor), 20.7 (minor), 20.2 (major), 14.1 (minor), 14.0 (major); **IR** (cm^{-1}) 2954, 2930, 2861, 1667, 1426, 1397, 698; **High Resolution MS** (ESI): Calculated for $C_{16}H_{24}BrNONa$ [M+Na]$^+$: 348.0933, Found: 348.0934.

The relative regiochemistry of the title compound was corroborated by ^{1}H NMR, HSQC and HMBC experiments (see Spectral Data).

***N*-(6-Bromohexyl)-*N*-phenylformamide (7a)**

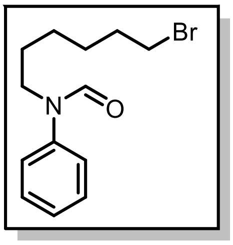

Colourless liquid (54 mg, 95%); *A 10:1 mixture of rotamers, Spectral data of major rotamer is reported*; **^{1}H NMR** (600 MHz, $CDCl_3$) δ 8.36 (s, 1H), 7.41 (t, *J* = 7.5 Hz, 2H), 7.29 (t, *J* = 7.5 Hz, 1H), 7.16 (d, *J* = 7.5 Hz, 2H), 3.81 (t, *J* = 7.5 Hz, 2H), 3.35 (t, *J* = 7.0 Hz, 2H), 1.80 (app. quint., *J* = 7.0 Hz, 2H), 1.54 (app. quint., *J* = 7.5 Hz, 2H), 1.41 (app. quint., *J* = 7.5 Hz, 2H), 1.31 (app. quint., *J* = 7.5 Hz, 2H); **^{13}C NMR** (150 MHz, $CDCl_3$) δ 162.3, 140.9, 129.6, 126.8, 124.2, 44.7, 33.6, 32.5, 27.6, 27.3, 25.8; **IR** (cm^{-1}) 2931, 2857, 1671, 1595, 1495, 1358, 1261, 910, 732; **High Resolution MS** (EI): Calculated for $C_{13}H_{18}BrNO$ [M]$^+$: 283.0572, Found: 283.0573.

***N*-(6-Bromohexyl)-*N*-butylformamide (7b)**

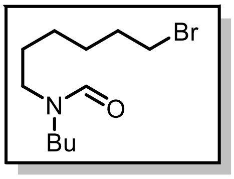

The reaction was performed at 80 °C; Yellow liquid (36 mg, 68%); *Data recorded on a 1.1:1 mixture of rotamers*; **^{1}H NMR** (400 MHz, CD_2Cl_2) δ 8.00 (s, 1H major + 1H minor), 3.42 (2 × t, *J* = 7.0 Hz, 2H major + 2H minor), 3.27 – 3.23 (m, 2H major + 2H minor), 3.17 (2 × t, *J* = 7.0 Hz, 2H major + 2H minor), 1.89 – 1.82 (m, 2H major + 2H minor), 1.56 – 1.41 (m, 6H major + 6H minor), 1.35 – 1.24 (m, 4H major + 4H minor), 0.92 (2 × t, *J* = 7.0 Hz, 3H major + 3H minor); **^{13}C NMR** (100 MHz, CD_2Cl_2) δ 163.0 (major), 162.9 (minor), 47.7 (minor), 47.6 (major), 42.3 (major), 42.1 (minor), 34.6 (major), 34.5 (minor), 33.3 (major), 33.2 (minor), 31.3 (major), 29.9 (minor), 29.0 (minor), 28.4 (major), 28.3 (minor), 27.7 (major), 26.6 (major), 26.2 (minor), 20.7 (minor), 20.2 (major), 14.1 (minor), 14.0 (major); **IR** (cm^{-1}) 2460, 2242, 2212, 2069, 1650, 1122, 974, 823; **High Resolution MS** (ESI): Calculated for $C_{11}H_{22}BrNONa$ [M+Na]$^+$: 286.0777, Found: 286.0775.

***N*-(7-Bromoheptyl)-*N*-phenylformamide (9a)**

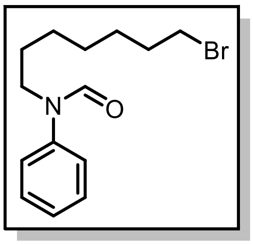

Brown liquid (48 mg, 80%); *A 10:1 mixture of rotamers, Spectral data of major rotamer is reported*; **^{1}H NMR** (400 MHz, $CDCl_3$) δ 8.36 (s, 1H), 7.41 (t, *J* = 7.5 Hz, 2H), 7.30 (t, *J* = 7.5 Hz, 1H), 7.16 (d, *J* = 7.5 Hz, 2H), 3.80 (t, *J* = 7.5 Hz, 2H), 3.37 (t, *J* = 7.0 Hz, 2H), 1.81 (app. quint., *J* = 7.0 Hz, 2H), 1.57 – 1.50 (m, 2H), 1.42 – 1.35 (m, 2H), 1.33 – 1.26 (m, 4H); **^{13}C NMR** (100 MHz, $CDCl_3$) δ 162.3, 140.9, 129.6, 126.8, 124.2, 44.8, 33.8, 32.6, 28.3, 27.9, 27.4, 26.5; **IR** (cm^{-1}) 2929, 2855, 1673, 1595, 1496, 1357, 1266, 911, 732, 698; **High Resolution MS** (EI): Calculated for $C_{14}H_{20}BrNO$ [M]$^+$: 297.0728, Found: 297.0728.

***N*-(7- Bromoheptyl)-*N*-butylformamide (9b)**

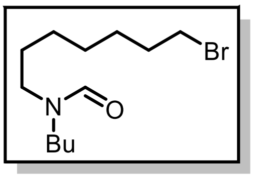

The reaction was performed at 60 °C; Colourless liquid (51 mg, 92%); *Data recorded on a 1.1:1 mixture of rotamers*; **^{1}H NMR** (400 MHz, $CDCl_3$) δ 8.03 (s, 1H major + 1H minor), 3.39 (2 × t, *J* = 7.0 Hz, 2H major + 2H minor), 3.27 (2 × t, *J* = 7.5 Hz, 2H major + 2H minor), 3.18 (app. t, *J* = 7.0 Hz, 2H major + 2H minor), 1.87 – 1.80 (m, 2H major + 2H minor), 1.56 – 1.46 (m, 4H major + 4H minor), 1.45 – 1.38 (m, 2H major + 2H minor), 1.37 – 1.24 (m, 6H major + 6H minor), 0.92 (2 × t, *J* = 7.0 Hz, 3H major + 3H minor); **^{13}C NMR** (100 MHz, $CDCl_3$) δ 162.9 (major), 162.8 (minor), 47.5 (minor), 47.3 (major), 42.1 (major), 42.0 (minor), 34.0 (major), 33.9 (minor), 32.8 (major), 32.7 (minor), 30.9 (major), 29.5 (minor), 28.7 (major), 28.5 (major), 28.4 (minor), 28.13 (major), 28.08 (minor), 27.3 (minor), 26.8 (major), 26.4 (minor), 20.3 (minor), 19.8 (major), 13.9 (minor), 13.8 (major); **IR** (cm^{-1}) 2927, 2856, 1669, 1426, 1379, 1261, 728; **High Resolution MS** (ESI): Calculated for $C_{12}H_{24}BrNONa$ $[M+Na]^+$: 300.0933, Found: 300.0929.

Procedure for late-stage ring-opening functionalization

***N*-{2-[(*N*-(2-bromoethyl)-4-ethoxy-3-(1-methyl-7-oxo-3-propyl-6,7-dihydro-1*H*-pyrazolo[4,3-*d*]pyrimidin-5-yl)phenyl)sulfonamide]ethyl}-*N*-phenylformamide (11)**

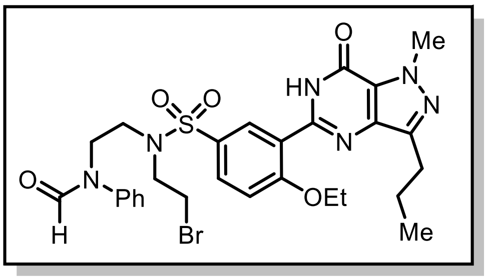

The reaction was performed at 60°C using 1,1,2,2-tetrachloroethane (0.5 mL) as an alternative solvent; Blue Solid (55 mg, 43%); **m.p.** 163 – 165 °C **^{1}H NMR** (400 MHz, $CDCl_3$) δ 10.81 (s, 1H), 8.83 (d, *J* = 2.5 Hz, 1H), 8.40 (s, 1H), 7.86 (dd, *J* = 9.0, 2.5 Hz, 1H), 7.42 (t, *J* = 7.5 Hz, 2H), 7.29 (t, *J* = 7.5 Hz, 1H), 7.21 (m, 2H), 7.11 (d, *J* = 9.0 Hz, 1H), 4.36 (q, *J* = 7.0 Hz, 2H), 4.26 (s, 3H), 4.07 – 3.99 (m, 2H), 3.60 – 3.54 (m, 2H), 3.53 – 3.46 (m, 2H), 3.43 (t, *J* = 7.0 Hz, 2H), 2.89 (t, *J* = 7.5 Hz, 2H), 1.82 (h, *J* = 7.5 Hz, 2H), 1.63 (t, *J* = 7.0 Hz, 3H), 0.98 (t, *J* = 7.0 Hz, 3H); **^{13}C NMR** (100 MHz, $CDCl_3$) δ 162.7, 159.5, 153.7, 147.1, 146.4, 140.9, 138.4, 132.2, 131.4, 130.6, 130.0, 127.2, 124.6, 123.5, 121.5, 113.3, 66.2, 51.0, 46.8, 45.4, 38.4, 29.0, 27.8, 22.4, 14.6, 14.1; **IR** (cm^{-1}) 3296, 2920, 2850, 1708, 1670, 1494, 1459, 1344, 1158, 728; **High Resolution MS** (EI): Calculated for $C_{28}H_{33}BrN_6O_5S$ $[M]^+$: 644.1417, Found: 644.1414.

Methyl (1*S*,2*R*,3*R*,4a*S*,14*S*,15a*S*)-14-acetoxy-6-formyl-2,11-dimethoxy-3-((3,4,5-trimethoxy-benzoyl)oxy)-2,3,4,4a,5,6,7,8,13,14,15,15a-dodecahydro-1*H*-benzo[8,9]azecino[5,4-*b*]indole-1-carboxylate (12)

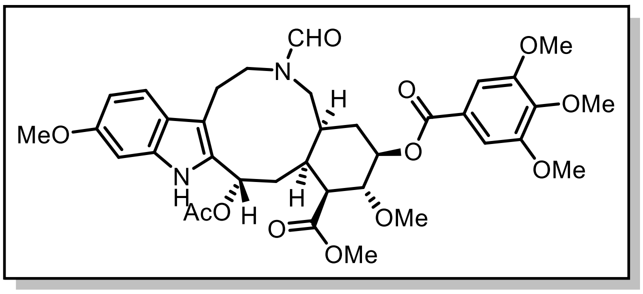

The reaction was performed at RT for 1 h, using TMSCF$_2$Cl (63 mg, 0.4 mmol, 2.0 equiv.), NH$_4$OAc (61.7 mg, 0.8 mmol, 4.0 equiv.) and 1,2-DCE (5.0 mL); Off-white solid (78 mg, 56%); **m.p.** 132 - 134 °C; **^{1}H NMR** (400 MHz, CD$_2$Cl$_2$) δ 8.16 (s, 1H), 7.40 (s, 1H), 7.39 (d, *J* = 8.5 Hz, 1H), 7.33 (s, 2H), 6.85 (d, *J* = 2.0 Hz, 1H), 6.75 (dd, *J* = 8.5, 2.0 Hz, 1H), 5.52 (dd, *J* = 13.0, 5.5 Hz, 1H), 5.00 – 4.94 (m, 1H), 4.00 – 3.95 (m, 1H), 3.92 (s, 3H), 3.91 – 3.89 (m, 1H – overlapping with other peaks), 3.90 (s, 6H), 3.85 (s, 3H), 3.82 (s, 3H), 3.51 (s, 3H), 3.44 (dd, *J* = 13.5, 5.0 Hz, 1H), 3.36 – 3.24 (m, 2H), 3.10 – 2.99 (m, 1H), 2.95 – 2.90 (m, 1H), 2.72 (t, *J* = 13.5 Hz, 1H), 2.52 (dd, *J* = 11.0, 4.0 Hz, 1H), 2.28 (t, *J* = 13.5 Hz, 1H), 2.13 (ddd, *J* = 13.5, 9.5, 5.5 Hz, 1H), 1.99 (s, 3H), 1.91 – 1.87 (m, 2H), 1.31 – 1.22 (m, 1H); **^{13}C NMR** (100 MHz, CD$_2$Cl$_2$) δ 174.0, 171.1, 165.7, 165.1, 157.6, 153.7, 142.9, 137.0, 131.7, 125.9, 122.7, 119.6, 110.8, 110.5, 107.3, 95.1, 79.6, 77.8, 70.4, 61.3, 61.1, 56.7, 56.0, 52.7, 51.9, 49.2, 48.5, 35.5, 33.2, 30.4, 27.8, 24.0, 21.5; **IR** (cm^{-1}) 2939, 2833, 1714, 1666, 1587, 1457, 1414, 1331, 1220, 1124, 984, 761; **High Resolution MS** (EI): Calculated for $C_{36}H_{44}N_2O_{12}$ [M]$^+$: 696.2894, Found: 696.2897; **Specific Rotation** $[\alpha]_D^{20}$ –120.0±0.8 (*c* 0.5, CH$_3$Cl).

The relative regiochemistry and stereochemistry of the title compound was corroborated by ^{1}H NMR, NOESY and HMBC experiments (see Spectral Data).

***N*-(5-Bromopentyl)-*N*-(3,3-diphenylpropyl)formamide (13)**

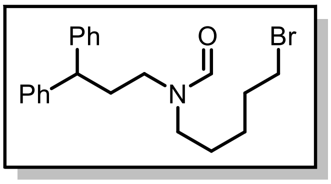

The reaction was performed at 80 ℃ for 36 h; Colourless liquid (47 mg, 60%); *Data recorded on a 1.3:1 mixture of rotamers*; **^{1}H NMR** (600 MHz, $CDCl_3$) δ 8.01 (s, 1H minor), 7.83 (s, 1H major), 7.32 – 7.26 (m, 4H major + 4H minor), 7.26 – 7.21 (m, 4H major + 4H minor), 7.21 – 7.15 (m, 2H major + 2H minor), 3.92 (t, *J* = 7.8 Hz, 1H minor), 3.87 (t, *J* = 7.9 Hz, 1H major), 3.53 – 3.44 (m, 2H major + 1H minor), 3.40 – 3.28 (m, 2H major + 1H minor), 3.25 – 3.21 (m, 2H minor), 3.18 – 3.12 (m, 2H major + 2H minor), 2.34 – 2.20 (m, 2H major + 2H minor), 1.87 – 1.77 (m, 2H minor), 1.77 – 1.70 (m, 2H major), 1.56 – 1.47 (m, 2H major), 1.46 – 1.38 (m, 2H major + 2H minor), 1.38 – 1.31 (m, 2H minor); **^{13}C NMR** (100 MHz, $CDCl_3$) δ 163.1 (major), 162.8 (minor), 144.2 (2C, minor), 143.6 (2C, major), 129.0 (4C, major), 128.8 (4C, minor), 127.8 (4C, minor), 127.8 (4C, major), 126.9 (2C, major), 126.6 (2C, minor), 49.3 (minor), 48.2 (major), 47.7 (minor), 45.7 (major), 45.0 (major), 44.7 (minor), 41.8 (major), 41.7 (minor), 34.2 (major), 33.1 (minor), 32.2 (major), 32.1 (minor), 28.1 (minor), 26.6 (major), 24.3 (major), 23.9 (minor); **IR** (cm^{-1}) 2934, 2862, 1667, 1493, 1450, 1428, 732, 704; **High Resolution MS** (ESI): Calculated for $C_{21}H_{26}BrNONa$ $[M+Na]^+$: 410.1090, Found: 410.1093.

***N*-(6-Bromohexyl)-*N*-(3,3-diphenylpropyl)formamide (14)**

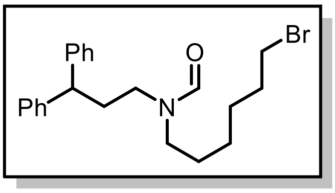

The reaction was performed at 80 ℃ for 36 h; Colourless liquid (65 mg, 81%); *Data recorded on a 1.2:1 mixture of rotamers*; **^{1}H NMR** (600 MHz, $CDCl_3$) δ 8.00 (s, 1H minor), 7.82 (s, 1H major), 7.32 – 7.26 (m, 4H major + 4H minor), 7.26 – 7.22 (m, 4H major + 4H minor), 7.21 – 7.15 (m, 2H major + 2H minor), 3.93 (t, *J* = 7.8 Hz, 1H minor), 3.87 (t, *J* = 7.9 Hz, 1H major), 3.50 (t, *J* = 6.6 Hz, 1H minor), 3.37 (t, *J* = 6.7 Hz, 2H major + 1H minor), 3.30 (t, *J* = 7.6 Hz, 2H major), 3.26 – 3.19 (m, 2H minor), 3.18 – 3.09 (m, 2H major + 2H minor), 2.36 – 2.24 (m, 2H major + 2H minor), 1.86 – 1.75 (m, 2H major + 2H minor), 1.53 – 1.46 (m, 2H major), 1.46 – 1.35 (m, 2H major + 4H minor), 1.32 – 1.16 (m, 2H major + 2H minor); **^{13}C NMR** (100 MHz, $CDCl_3$) δ 163.1 (major), 162.8 (minor), 144.2 (2C minor), 143.6 (2C major), 129.0 (4C major), 128.8 (4C minor), 127.8 (4C minor), 127.8 (4C major), 126.8 (2C major), 126.6 (2C minor), 49.3 (minor), 48.2 (major), 47.7 (minor), 45.7 (major), 41.9 (major), 41.7 (minor), 34.2 (minor), 33.9 (major), 33.8 (minor), 33.1 (major), 32.7 (major), 32.6 (minor), 28.6 (minor), 27.9 (minor), 27.7 (major), 27.2 (minor)z, 26.2 (major), 25.7 (minor); **IR** (cm^{-1}) 2932, 2858, 1666, 1493, 1450, 1427, 729, 699; **High Resolution MS** (ESI): Calculated for $C_{21}H_{26}BrNONa$ $[M+Na]^+$: 424.1246, Found: 424.1250.

(*S*)-*N*-[2-(2-Bromoethoxy)ethyl]-*N*-(3,7-dimethyloct-6-en-1-yl)formamide (15)

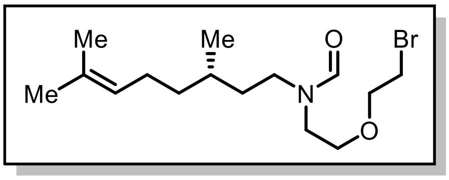

The reaction was performed at 80 °C using TMSCF$_2$Br (34 μL, 0.22 mmol, 1.1 equiv.) and NH$_4$OAc (17 mg, 0.22 mmol, 1.1 equiv.); Colourless liquid (42 mg, 63%); *Data recorded on a 1.3:1 mixture of rotamers;* **^{1}H NMR** (400 MHz, CDCl$_3$) δ 8.00 (s, 1H major), 7.99 (s, 1H minor), 5.01 (app. t, *J* = 7.0 Hz, 1H major + 1H minor), 3.71 – 3.24 (m, 10H major + 10H minor), 1.97 – 1.82 (m, 2H major + 2H minor), 1.62 (s, 3H major + 3H minor), 1.53 (s, 3H major + 3H minor), 1.50 – 1.06 (m, 5H major + 5H minor), 0.87 – 0.84 (m, 3H major + 3H minor); **^{13}C NMR** (100 MHz, CDCl$_3$) δ 163.3 (minor), 163.1 (major), 131.8 (major), 131.5 (minor), 124.7 (minor), 124.5 (major), 71.3 (minor), 71.0 (major), 69.21 (major), 69.19 (minor), 47.4 (minor), 47.0 (major), 42.3 (major), 41.3 (minor), 37.1 (minor), 37.0 (major), 36.1 (major), 34.5 (minor), 30.6 (major + minor), 30.2 (minor), 29.9 (major), 25.9 (major + minor), 25.6 (minor), 25.5 (major), 19.53 (minor), 19.49 (major), 17.82 (major), 17.81 (minor); **IR** (cm^{-1}) 2958, 2915, 2857, 1668, 1429, 1119, 910, 734; **High Resolution MS** (ESI): Calculated for $C_{15}H_{28}BrNO_2Na$ [M+Na]$^+$: 356.1196, Found: 356.1197; **Specific Rotation** $[\alpha]_D^{20}$ –39.5±0.5 (*c* 1.0, CH_3Cl).

Procedures for skeletal diversification

Procedure for intramolecular organolithium addition to formyl group

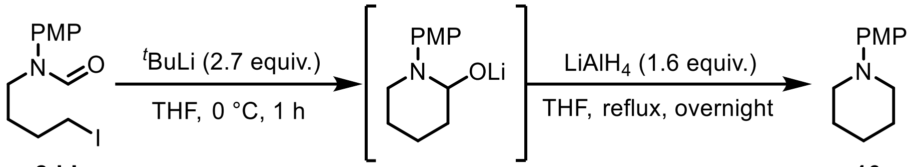

2d-I **16**

1-(4-Methoxyphenyl)piperidine (16)

To a solution of **2d-I** (67 mg, 0.2 mmol) in THF (10 ml) was added *tert*-butyllithium (1.7 M, 0.31 ml, 0.53 mmol) at 0 °C, and the reaction was further stirred at 0 °C for 1 h. $LiAlH_4$ (1 M solution in ether, 0.32 ml, 0.32 mmol) was then added and the reaction was heated overnight under reflux. After cooling to room temperature, the reaction was cautiously quenched with 2M $NaOH_{(aq)}$ solution (2 ml), followed by a saturated solution of Rochelle's salt (10 ml), and the mixture was vigorously stirred for 1 h. The organic phase was separated and the aqueous phase was extracted with ether (3 × 10 ml). The combined organic extracts were dried over Na_2SO_4, concentrated under reduced pressure, and purified by column chromatography on silica gel (5% EtOAc/Hexane) to give 1-(4-methoxyphenyl)piperidine as a grass green liquid (19 mg, 51%);

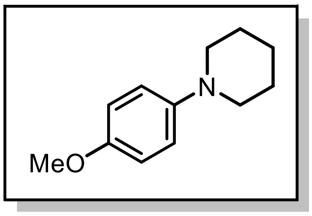

^{1}H NMR (400 MHz, $CDCl_3$) δ 6.93 (d, J = 9.0 Hz, 2H), 6.83 (d, J = 9.0 Hz, 2H), 3.77 (s, 2H), 3.12 – 2.94 (m, 4H), 1.73 (app. quint., J = 6.0 Hz, 4H), 1.60 – 1.49 (m, 2H); **^{13}C NMR** (100 MHz, $CDCl_3$) δ 153.8, 147.0, 119.0, 114.5, 55.8, 52.6, 26.3, 24.4; **IR** (cm^{-1}) 2930, 2851, 2789, 1509, 1241, 1040, 823; **High Resolution MS** (EI): Calculated for $C_{12}H_{17}NO$ $[M]^+$: 191.1310, Found: 191.1307. Data consistent with those reported[15].

Procedure for Bishler-Napieralski reaction

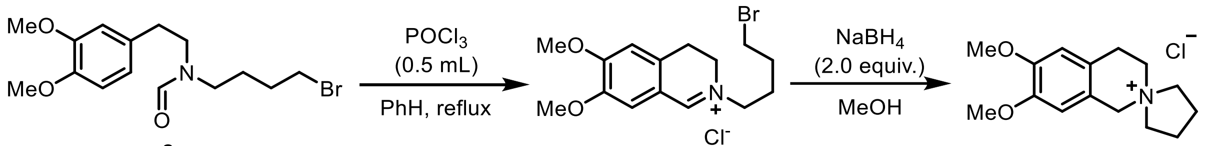

2s **17**

To a solution of **2s** (68 mg, 0.2 mmol) in benzene (1.5 mL) was added $POCl_3$ (0.5 mL). The reaction mixture heated under reflux for 12 h. After cooling to room temperature, the crude was concentrated *in vacuo* and diluted in MeOH (2.0 mL). To this was slowly added $NaBH_4$ (15 mg, 0.4 mmol) at 25 °C, and the reaction was stirred at room temperature for 3 h. The reaction was quenched by an addition of H_2O (10 mL) and extracted with DCM (3 × 15 mL). The combined organic extracts were dried over Na_2SO_4, concentrated under reduced pressure, and purified by column chromatography on silica gel using Hexane/EtOAc as eluent.

2-(4-Bromobutyl)-6,7-dimethoxy-3,4-dihydroisoquinolin-2-ium chloride

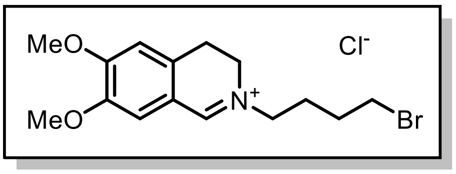

The title compound was obtained in a separate reaction to confirm the intermediate, by quenching the reaction before the addition of $NaBH_4$,; Pale yellow resin (61 mg, 91%); **^{1}H NMR** (400 MHz, methanol-*d4*) δ 8.97 (s, 1H), 7.41 (s, 1H), 7.13 (s, 1H), 4.07 – 4.01 (m, 4H), 4.00 (s, 3H), 3.90 (s, 3H), 3.68 (t, *J* = 6.5 Hz, 2H), 3.25 (t, *J* = 8.0 Hz, 2H), 2.12 – 2.05 (m, 2H), 1.94 – 1.87 (m, 2H); **^{13}C NMR** (100 MHz, methanol-*d4*) δ 166.0, 159.4, 150.3, 134.5, 118.4, 116.4, 112.3, 60.5, 57.3, 56.9, 48.9, 45.0, 30.3, 26.2, 26.1; **IR** (cm^{-1}) 2943, 2834, 1651, 1566, 1523, 1344, 1298, 1138, 1021, 980; **High Resolution MS** (EI): Calculated for $C_{15}H_{21}BrNO_2$ $[M-Cl]^+$: 326.0750, Found: 326.0754

6,7-Dimethoxy-3,4-dihydro-1*H*-spiro[isoquinoline-2,1'-pyrrolidin]-2-ium chloride (17)

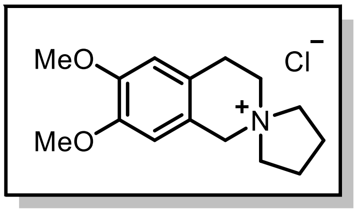

Pale yellow resin (61 mg, 93%); **^{1}H NMR** (600 MHz, $CDCl_3$) δ 6.67 (s, 1H), 6.66 (s, 1H), 4.75 (s, 2H), 4.11 (t, *J* = 6.5 Hz, 2H), 4.01 – 3.92 (m, 2H), 3.91 – 3.86 (m, 2H), 3.85 (s, 3H), 3.82 (s, 3H), 3.16 (t, *J* = 6.5 Hz, 2H), 2.32 (m, 4H); **^{13}C NMR** (150 MHz, $CDCl_3$) δ 149.8, 148.8, 121.6, 118.6, 111.2, 110.0, 62.4, 61.1, 57.8, 56.4, 56.3, 25.0, 21.8; **IR** (cm^{-1}) 2958, 2921, 2846, 1637, 1519, 1447, 1361, 1262, 1122; **High Resolution MS** (ESI): Calculated for $C_{15}H_{22}NO_2$ $[M-Cl]^+$: 248.1645, Found: 248.1605. *The structure of the title compound was further confirmed by X-ray crystallographic analysis (see X-ray Crystallographic Data).*

Procedure for E2 elimination reaction

To a solution of **2d** or **2s** (1.0 mmol) in toluene (8.5 mL) was added KO^{t}Bu (1.5 mL, 1.5 mmol, 1.0 M solution in ^{t}BuOH). The reaction mixture was stirred at 100 °C for 16 h, cooled to room temperature, filtered through a short pad of silica gel, and concentrated *in vacuo*. The crude product was purified by column chromatography on silica gel using Hexane/EtOAc as eluent.

***N*-(But-3-en-1-yl)-*N*-(4-methoxyphenyl)formamide**

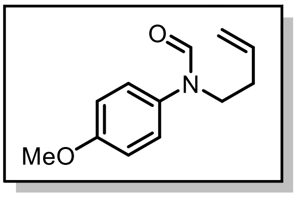

brown liquid (187 mg, 91%); **^{1}H NMR** (400 MHz, $CDCl_3$) δ 8.25 (s, 1H), 7.09 (d, *J* = 9.0 Hz, 2H), 6.92 (d, *J* = 9.0 Hz, 2H), 5.74 (ddt, *J* = 16.5, 11.5, 7.0 Hz, 1H), 5.06 – 5.03 (m, 1H), 5.01 (app. t, *J* = 1.5 Hz, 1H), 3.84 – 3.80 (m, 2H), 3.82 (s, 3H), 2.27 (app. q, *J* = 7.0 Hz, 2H); **^{13}C NMR** (100 MHz, $CDCl_3$) δ 162.7, 158.8, 135.0, 133.8, 126.8, 117.2, 114.9, 55.7, 44.8, 32.1; **IR** (cm^{-1}) 3076, 2934, 2837, 1667, 1500, 1357, 1244, 1179, 1031, 832; **High Resolution MS** (ESI): Calculated for $C_{12}H_{15}NO_2Na$ $[M+Na]^+$: 228.0995, Found: 228.0988

***N*-(But-3-en-1-yl)-*N*-(3,4-dimethoxyphenethyl)formamide**

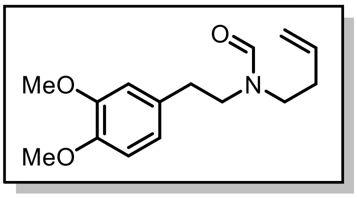

Colourless liquid (251 mg, 95%); *Data recorded on a 1.1:1 mixture of rotamers*; **^{1}H NMR** (400 MHz, $CDCl_3$) δ 8.03 (s, 1H major), 7.82 (s, 1H minor), 6.80 – 6.72 (m, 2H major + 2H minor), 6.67 – 6.60 (m, 1H major + 1H minor), 5.89 – 5.61 (m, 1H major + 1H minor), 5.11 – 5.02 (m, 2H major + 2H minor), 3.86 (s, 3H major), 3.85 (s, 3H minor), 3.84 (s, 3H major + 3H minor), 3.51 – 3.47 (m, 2H major), 3.41 (t, *J* = 7.0 Hz, 2H minor), 3.40 – 3.36 (m, 2H minor), 3.17 (t, *J* = 7.0 Hz, 2H major), 2.81 – 2.78 (m, 2H major), 2.75 (t, *J* = 7.0 Hz, 2H minor), 2.31 (app. q, *J* = 7.0 Hz, 2H minor), 2.24 (app. q, *J* = 7.0 Hz, 2H major); **^{13}C NMR** (100 MHz, $CDCl_3$) δ 162.92 (major), 162.87 (minor), 149.2 (minor), 149.0 (major), 148.0 (minor), 147.7 (major), 135.1 (minor), 134.0 (major), 131.4 (major), 130.3 (minor), 120.9 (minor), 120.7 (major), 118.2 (major), 117.1 (minor), 112.03 (major), 112.0 (minor), 111.5 (minor), 111.4 (major), 56.01 (minor), 55.99 (major), 55.98 (major), 55.97 (minor), 49.4 (minor), 47.6 (major), 44.5 (major), 42.0 (minor), 35.1 (minor), 33.4 (major), 33.2 (major), 32.0 (minor); **IR** (cm^{-1}) 2933, 2834, 1663, 1590, 1421, 1260, 1235, 1153, 1027, 764; **High Resolution MS** (EI): Calculated for $C_{15}H_{21}NO_3$ $[M]^+$: 263.1521, Found: 263.1523.

Procedure for Ni-catalysed intramolecular hydrocarbamoylation

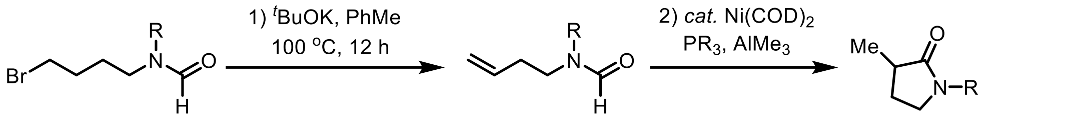

R = PMP, **2d**
R = (3,4-dimethoxyphenyl)ethyl, **2s**

R = PMP, **18**
R = (3,4-dimethoxyphenyl)ethyl, **19**

1-(4-Methoxyphenyl)-3-methylpyrrolidin-2-one (18)

A solution of *N*-(but-3-en-1-yl)-*N*-(4-methoxyphenyl)formamide (42 mg, 0.2 mmol) and $AlMe_3$ (40 µL, 0.08 mmol, 2.0 M solution in toluene) in degassed toluene (0.3 mL) was added to a solution of Ni(cod)$_2$ (5.5 mg, 0.02 mmol), PPh_3 (5.2 mg, 0.02 mmol) and H(O)P(^{t}Bu)$_2$ (3.2 mg, 0.02 mmol) in degassed toluene (0.1 mL) under argon atmosphere. The mixture was stirred at 40 °C for 24 h, filtered through a short pad of silica gel, and concentrated *in vacuo*. The title compound was isolated as a white solid after column chromatography purification (20% EtOAc/Hexane);

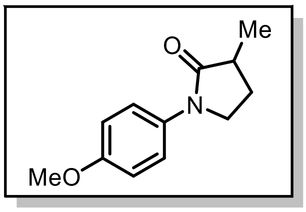

White solid (23 mg, 56%); **m.p.** = 83 - 85 °C; **^{1}H NMR** (400 MHz, $CDCl_3$) δ 7.52 (d, *J* = 9.0 Hz, 2H), 6.90 (d, *J* = 9.0 Hz, 2H), 3.80 (s, 3H), 3.77 – 3.69 (m, 2H), 2.70 – 2.60 (m, 1H), 2.36 (app. dddd, *J* = 12.5, 8.5, 7.0, 3.5 Hz, 1H), 1.76 (app. dq, *J* = 12.5, 8.5 Hz, 1H), 1.3 (d, *J* = 7.0 Hz, 3H); **^{13}C NMR** (100 MHz, $CDCl_3$) δ 176.5, 156.5, 133.1, 121.6, 114.1, 55.6, 47.1, 38.2, 27.2, 16.4; **IR** (cm^{-1}) 2928, 2856, 1673, 1594, 1511, 1496, 1246, 1029, 829, 698; **High Resolution MS** (EI): Calculated for $C_{12}H_{15}NO_2$ $[M]^+$: 205.1103, Found: 205.1099. Data consistent with those reported[16].

1-(3,4-Dimethoxyphenethyl)-3-methylpyrrolidin-2-one (19)

A solution of *N*-(but-3-en-1-yl)-*N*-(3,4-dimethoxyphenethyl)formamide (53 mg, 0.2 mmol) and $AlMe_3$ (40 µL, 0.08 mmol, 2.0 M solution in toluene) in degassed toluene (0.3 mL) was added to a solution of $Ni(cod)_2$ (5.5 mg, 0.02 mmol) and $P(^tBu)_3$ (16.2 mg, 0.08 mmol) in degassed toluene (0.1 mL) under argon atmosphere. The mixture was stirred at 40 °C for 24 h, filtered through a short pad of silica gel, and concentrated *in vacuo*. The title compound was isolated as a colourless liquid after column chromatography purification (80% EtOAc/Hexane);

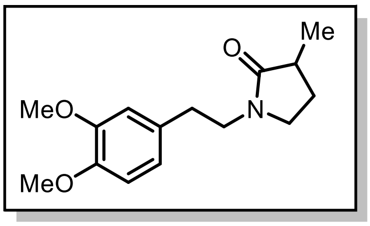

Colourless liquid (41 mg, 78%) **^{1}H NMR** (400 MHz, $CDCl_3$) δ 6.78 – 6.70 (m, 3H), 3.85 (s, 3H), 3.83 (s, 3H), 3.55 – 3.42 (m, 2H), 3.16 – 3.11 (m, 2H), 2.77 (t, *J* = 7.5 Hz, 2H), 2.39 (app. sextet, *J* = 7.0 Hz, 1H), 2.18 – 2.10 (m, 1H), 1.56 – 1.46 (m, 1H), 1.14 (d, *J* = 7.0 Hz, 3H); **^{13}C NMR** (100 MHz, $CDCl_3$) δ 177.2, 148.8, 147.5, 131.3, 120.5, 111.8, 111.2, 55.8 (2C), 45.6, 44.1, 36.7, 33.3, 27.2, 16.3; **IR** (cm^{-1}) 2932, 2834, 1667, 1514, 1459, 1423, 1261, 1235, 1155, 1027, 731; **High Resolution MS** (EI): Calculated for $C_{15}H_{21}NO_3$ $[M]^+$: 263.1521, Found: 263.1523.

Procedure for intramolecular Kulinkovich-de Meijere reaction

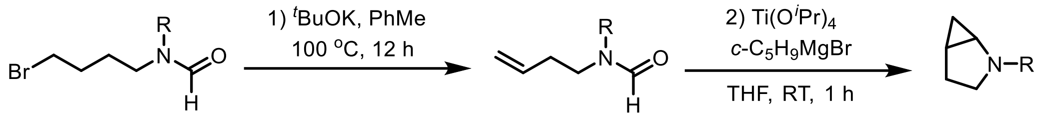

R = PMP, **2d**
R = (3,4-dimethoxyphenyl)ethyl, **2s**

R = PMP, **20**
R = (3,4-dimethoxyphenyl)ethyl, **21**

2-(4-Methoxyphenyl)-2-azabicyclo[3.1.0]hexane (20)

To a solution of *N*-(but-3-en-1-yl)-*N*-(4-methoxyphenyl)formamide (42 mg, 0.2 mmol) and Ti(O^{i}Pr)$_4$ (177 μL, 0.6 mmol) in Et_2O (4 mL) was slowly added cyclopentylmagnesium chloride (0.5 mL, 1.0 mmol, 2.0 M solution in Et_2O) over 20 min at room temperature. After stirring for 1 h, the mixture was quenched with H_2O (20 mL) and extracted with EtOAc (2 × 30 mL). The combined organic phases were dried over $MgSO_4$, filtered and concentrated *in vacuo*. The title compound was isolated as a colourless liquid after column chromatography purification on neutral alumina gel (5% EtOAc/Hexane);

Note: Unlike the N,N-dialkyl substrate, THF as a solvent is less efficient for N-aryl substrates. The use of a large excess amount of Ti(O^{i}Pr)$_4$ is also crucial for obtaining good yields.

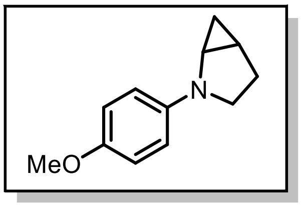

Colourless liquid (31 mg, 82%); **^{1}H NMR** (400 MHz, CD_2Cl_2) δ 6.82 – 6.76 (m, 4H), 3.73 (s, 3H), 3.63 (td, *J* = 9.5, 1.5 Hz, 1H), 3.15 (ddd, *J* = 6.5, 5.5, 2.5 Hz, 1H), 2.56 (td, *J* = 9.5, 8.0 Hz, 1H), 2.30 – 2.21 (m, 1H), 2.07 (ddd, *J* = 12.5, 8.0, 1.5 Hz, 1H), 1.64 – 1.58 (m, 1H), 0.58 (dt, *J* = 8.5, 5.5 Hz, 1H), 0.40 (dt, *J* = 5.0, 2.5 Hz, 1H); **^{13}C NMR** (100 MHz, CD_2Cl_2) δ 152.7, 145.5, 116.1, 114.9, 56.2, 47.9, 38.4, 27.3, 16.6, 8.3; **IR** (cm^{-1}) 2928, 2848, 1510, 1240, 1038, 808, 764; **High Resolution MS** (EI): Calculated for $C_{12}H_{15}NO$ $[M]^+$: 189.1154, Found: 189.1155.

2-(3,4-Dimethoxyphenethyl)-2-azabicyclo[3.1.0]hexane (21)

To a solution of *N*-(but-3-en-1-yl)-*N*-(3,4-dimethoxyphenethyl)formamide (53 mg, 0.2 mmol) and $Ti(O^iPr)_4$ (88 µL, 0.3 mmol) in THF (4 mL) was slowly added cyclopentylmagnesium chloride (0.45 mL, 0.9 mmol, 2.0 M solution in Et_2O) over 20 min at room temperature. After stirring for 1 h, the mixture was quenched with H_2O (20 mL) and extracted with EtOAc (2 × 30 mL). The combined organic phases were dried over $MgSO_4$, filtered and concentrated *in vacuo*. The title compound was isolated as a colourless liquid after column chromatography purification on neutral alumina gel (50% EtOAc/Hexane);

Note: For all substrates, the slow addition, as well as concentration, is very crucial for achieving good yields in this reaction. Fast addition and reactions that are more concentrated lead to unknown side products, affording only a negligible amount of the desired product.

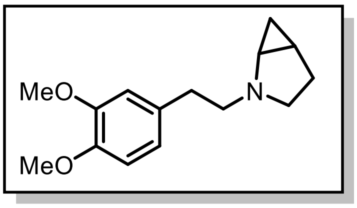

Colourless liquid (38 mg, 77%); **^{1}H NMR** (400 MHz, CD_2Cl_2) δ 6.84 – 6.75 (m, 3H), 3.82 (s, 3H), 3.79 (s, 3H), 2.93 (t, *J* = 7.5 Hz, 1H), 2.80 – 2.60 (m, 5H), 1.99 – 1.80 (m, 3H), 1.41 – 1.35 (m, 1H), 0.68 – 0.65 (m, 1H), 0.08 – 0.03 (m, 1H); **^{13}C NMR** (100 MHz, CD_2Cl_2) δ 149.6, 148.0, 134.1, 121.0, 112.9, 112.1, 56.9, 56.4, 56.3, 48.9, 41.2, 35.7, 27.4, 15.4, 1.8; **IR** (cm^{-1}) 2932, 2832, 1514, 1462, 1261, 1235, 1141, 1027, 805; **High Resolution MS** (EI): Calculated for $C_{15}H_{21}NO_2$ $[M]^+$: 247.1572, Found: 247.1574.

Procedure for skeletal remodeling of 6-membered cyclic amines

N-(4-Methoxyphenyl)-*N*-[2-(2-oxotetrahydrofuran-3-yl)ethyl]formamide (22)

To a 2.0 mL reaction vial containing a magnetic stir bar were added **3g** (79 mg, 0.3 mmol), NH_4OAc (91.5 mg, 1.2 mmol), and 1,2-DCE (0.75 mL), followed by $TMSCF_2Br$ (187.5 µL, 1.2 mmol). The reaction vial was sealed and stirred at 60 °C for 12 h. The reaction mixture was then cooled to room temperature and the solvent was removed under reduced pressure. The residue was diluted in 1,4-dioxane (15 mL) and H_2O (15 mL), and to this was added $AgNO_3$ (242 mg, 1.4 mmol). The reaction mixture was heated at 90 °C for 4 h, cooled to room temperature, and filtered. 1,4-Dioxane was removed *in vacuo* and the aqueous residue was extracted EtOAc (20 mL × 3). The organic layer was washed with brine, dried over $MgSO_4$, and concentrated under reduced pressure. The title compound was isolated as a colourless liquid (66 mg, 83%) after column chromatography purification on silica gel (20 % EtOAc/Hexane);

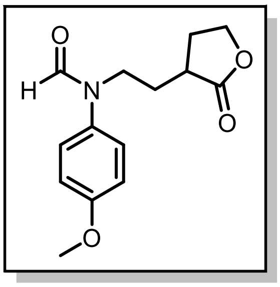

A 19:1 mixture of rotamers, Spectral data of major rotamer is reported; ^{1}**H NMR** (600 MHz, $CDCl_3$) δ 8.26 (s, 1H), 7.11 – 7.06 (m, 2H), 6.95 – 6.87 (m, 2H), 4.37 – 4.28 (m, 1H), 4.21 – 4.14 (m, 1H), 4.01 – 3.93 (m, 1H), 3.81 (s, 3H), 3.80 – 3.76 (m, 1H), 2.58 – 2.44 (m, 2H), 2.12 – 2.06 (m, 1H), 2.04 – 1.93 (m, 1H), 1.66 – 1.59 (m, 1H); ^{13}**C NMR** (150 MHz, $CDCl_3$) δ 179.0, 162.9, 159.0, 133.3, 126.6, 115.2, 66.8, 55.8, 43.4, 37.3, 29.1, 28.7; **IR** (cm^{-1}) 2935, 2912, 2838, 1763, 1666, 1511, 1372, 1247, 1158, 1026, 837; **High Resolution MS** (ESI): Calculated for $C_{14}H_{17}NO_4Na$ $[M+Na]^+$: 286.1050, Found: 286.1050.

N-[2-(2-Oxooxazolidin-3-yl)ethyl]-*N*-phenylformamide (23)

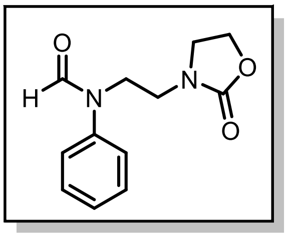

The title compound was prepared according to the general procedure for deconstructive C–N bromoformylation using ***3s****. The reaction was performed at 60°C*; White solid (37 mg, 79%); **m.p.** 103 - 105 °C; ^{1}**H NMR** (600 MHz, $CDCl_3$) δ 8.33 (s, 1H), 7.41 (t, J = 7.5 Hz, 2H), 7.31 (t, J = 7.5 Hz, 1H), 7.24 (d, J = 7.5 Hz, 2H), 4.20 (t, J = 8.0 Hz, 2H), 3.98 (t, J = 6.0 Hz, 2H), 3.63 (t, J = 8.0 Hz, 2H), 3.43 (t, J = 6.0 Hz, 2H); ^{13}**C NMR** (150 MHz, $CDCl_3$) δ 162.8, 158.4, 140.2, 129.8, 127.4, 124.9, 61.9, 44.4, 42.4, 42.3; **IR** (cm^{-1}) 2967, 2928, 2894, 1717, 1665, 1592, 1495, 1457, 1362, 1349, 1242, 1052, 756; **High Resolution MS** (EI): Calculated for $C_{12}H_{14}N_2O_3$ $[M]^+$: 234.1004, Found: 234.1006.

The structure of the title compound was further confirmed by X-ray crystallographic analysis (see X-ray Crystallographic Data).

(*E*)-*N*-[2-(3,4-Dimethoxystyryl)-4,5-dimethoxyphenethyl]-*N*-methylformamide (24)

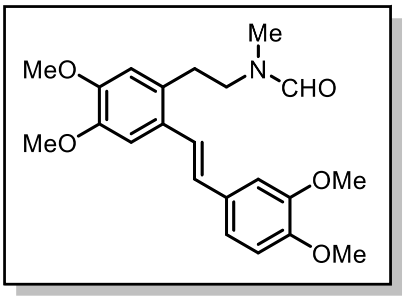

The reaction was performed on a 1.0 mmol scale at 25 °C for 12 h, using TMSCF$_2$Br (170 µL, 1.1 mmol, 1.1 equiv.), NH$_4$OAc (85 mg, 1.1 mmol, 1.1 equiv.) and 1,2-DCE (15 mL); White solid (355 mg, 92%); *Data recorded on a 1.1:1 mixture of rotamers*; **m.p.** 140 – 142 °C; **^{1}H NMR** (400 MHz, CDCl$_3$) δ 8.03 (s, 1H major), 7.83 (s, 1H minor), 7.37 (d, *J* = 16.0 Hz, 1H major), 7.22 (d, *J* = 2.0 Hz, 1H major), 7.13 (s, 1H major), 7.11 – 7.04 (m, 1H major + 3H minor), 7.01 (d, *J* = 2.0 Hz, 1H minor), 6.88 – 6.83 (m, 2H major + 2H minor), 6.69 (s, 1H major), 6.58 (s, 1H minor), 3.99 (s, 3H minor), 3,94 (s, 3H major), 3.933 (s, 3H major), 3.927 (s, 3H minor), 3.90 (s, 3H minor), 3.893 (s, 3H major), 3.890 (s, 3H major), 3.88 (s, 3H minor), 3.51 – 3.50 (m, 2H, major), 3.42 (t, *J* = 7.0 Hz, 2H minor), 2.98 – 2.92 (m, 2H major + 2H minor), 2.89 (s, 3H minor), 2.85 (s, 3H major); **^{13}C NMR** (100 MHz, CDCl$_3$) δ 162.6 (major + minor), 149.4 (major), 149.3 (minor), 149.1 (minor), 148.9 (major), 148.8 (minor), 148.7 (major), 148.3 (minor), 148.1 (major), 130.9 (major), 130.6 (minor), 129. (major), 129.1 (minor), 129.0 (major + minor) 128.5 (major), 128.0 (minor), 123.6 (major), 123.5 (minor), 119.8 (major), 119.3 (minor), 113.11 (minor), 113.07 (major), 111.5 (minor), 111.3 (major), 109.2 (major), 109.1 (minor), 108.9 (major), 108.3 (minor), 56.2 – 56.0 (4C major + 4C minor), 50.9 (minor), 46.5 (major), 35.5 (major), 32.6 (minor), 30.7 (major), 30.1 (minor); **IR** (cm^{-1}) 2969, 2848, 1672, 1599, 1510, 1360, 1259, 1199, 1158, 1097, 1023, 960, 865; **High Resolution MS** (EI): Calculated for $C_{22}H_{27}NO_5$ [M]$^+$: 385.1889, Found: 385.1890.

The relative regiochemistry and stereochemistry of the title compound was corroborated by ^{1}H NMR, NOESY and HMBC experiments (see Spectral Data), as well as by X-ray crystallographic analysis (see X-ray Crystallographic Data).

Procedure for Ring-Closing Reaction of (*E*)-*N*-[2-(3,4-Dimethoxystyryl)-4,5-dimethoxyphenethyl]-*N*-methylformamide (25)

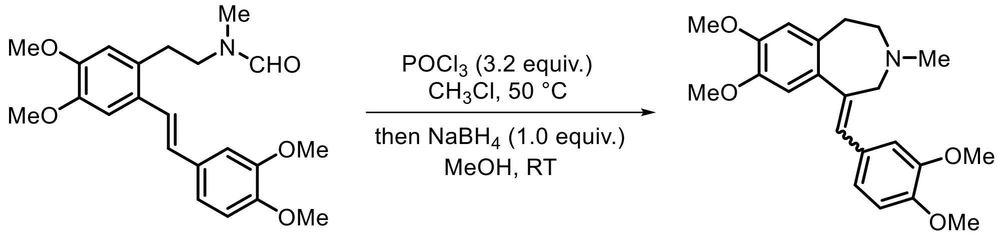

To a 2.0 mL reaction vial containing a solution of (*E*)-*N*-[2-(3,4-dimethoxystyryl)-4,5-dimethoxyphenethyl]-*N*-methylformamide (77 mg, 0.2 mmol) in $CHCl_3$ (1.0 mL) was added $POCl_3$ (60 µL, 0.64 mmol). The reaction mixture was then stirred at 50 °C for 12 h. After cooling to room temperature, the crude was concentrated *in vacuo* and diluted again in MeOH (2.0 mL). To this was slowly added $NaBH_4$ (8 mg, 0.21 mmol, 1.05 equiv.) at 0 °C, and the reaction was stirred at room temperature for 1h. The reaction was quenched by an addition of H_2O (10 mL) and extracted with DCM (3 × 15 mL). The combined organic phase was dried over $MgSO_4$, filtered and concentrated *in vacuo*. The major isomer was first isolated by preparative thin layer chromatography (10% MeOH/DCM), and the minor one was further purified by silica column chromatography (100% EtOAc).

(*E*)-1-(3,4-Dimethoxybenzylidene)-7,8-dimethoxy-3-methyl-2,3,4,5-tetrahydro-1H-benzo[*d*]-azepine

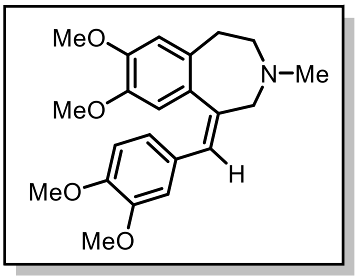

Off-white solid (34 mg, 46%); **m.p.** 132 - 134 °C; **^{1}H NMR** (400 MHz, $CDCl_3$) δ 6.69 (s, 1H), 6.63 (d, *J* = 8.5 Hz, 1H), 6.60 (d, *J* = 8.5, 2.0 Hz, 1H), 6.54 (s, 1H), 6.52 – 6.50 (m, 2H), 3.85 (s, 3H), 3.78 (s, 3H), 3.54, (s, 3H), 3.52 (s, 3H), 3.31 (s, 2H), 2.92 – 2.82 (m, 4H), 2.49 (s, 3H); **^{13}C NMR** (100 MHz, $CDCl_3$) δ 148.11, 148.05, 147.8, 147.3, 136.7, 133.1, 132.4, 130.6, 129.5, 122.7, 112.8 (2C), 112.3, 110.6, 64.8, 56.2, 56.00, 55.97, 55.8, 55.5, 44.5, 33.9; **IR** (cm^{-1}) 2915, 2848, 1672, 1511, 1460, 1259, 1240, 1138, 1027, 729; **High Resolution MS** (EI): Calculated for $C_{22}H_{27}NO_4$ $[M]^+$: 369.1940, Found: 369.1937.

The relative regiochemistry and stereochemistry of the title compound was corroborated by ^{1}H NMR, NOESY and HMBC experiments (see Spectral Data).

(*Z*)-1-(3,4-Dimethoxybenzylidene)-7,8-dimethoxy-3-methyl-2,3,4,5-tetrahydro-1H-benzo[*d*]-azepine

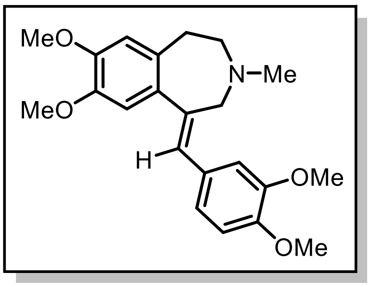

Yellow liquid (18 mg, 24%); **^{1}H NMR** (400 MHz, $CDCl_3$) δ 7.14 (d, *J* = 2.0 Hz, 1H), 7.06 (dd, *J* = 8.0, 2.0 Hz, 1H), 6.90 (d, *J* = 8.0 Hz, 1H), 6.89 (s, 1H), 6.66 (s, 1H), 6.61 (s, 1H), 3.94 (s, 3H), 3.93 (s, 3H), 3.91 (s, 3H), 3.89 (s, 3H), 3.32 (s, 2H), 2.90 – 2.85 (m, 2H), 2.79 – 2.74 (m, 2H), 2.39 (s, 3H); **^{13}C NMR** (100 MHz, $CDCl_3$) δ 148.7, 148.2, 147.9, 147.4, 140.4, 137.0, 131.4, 130.6, 130.0, 122.0, 112.8, 112.6, 111.1, 111.0, 58.6, 57.5, 56.3, 56.1, 56.03, 55.99, 46.7, 34.5; **IR** (cm^{-1}) 2916, 2847, 2835, 1510, 1464, 1263, 1159, 735; **High Resolution MS** (ESI): Calculated for $C_{22}H_{28}NO_4$ $[M+H]^+$: 370.2013, Found: 370.2016.

The relative regiochemistry and stereochemistry of the title compound was corroborated by ^{1}H NMR, NOESY and HMBC experiments (see Spectral Data).

Computational details

All calculations were carried out using density functional theory[17] with Gaussian 09 program.[18] Geometry optimizations to the stationary points as well as transition state optimizations to the 1st-order saddle points were performed with M06-2X functional[19] and the 6-31G+(d,p) basis set[20-23] for main group elements. The bromine atom was represented using Los Alomos LANL2DZ basis set.[24] For those structures having various conformations, the most stable conformer was searched and utilized. Vibrational frequencies were carried out at the same level of theory as the geometry optimizations. Thermodynamic parameters at the reaction temperatures were obtained by frequency calculations. Frequency analysis verified that the stationary points were minima or first-order saddle points. Electronic energies of the optimized structures were further reevaluated by single-point calculations with M06-2X functional and the high quality triple-ζ 6-311++G(d,p) basis set.[23,25] Solvation of dichloroethane (ε = 10.125) was considered by single-point calculations with integral equation formalism PCM model. Additionally, the computational details for other program are described in the corresponding section, and those calculations were conducted with the optimized geometries from Gaussian 09 program. Schemes of three-dimensional molecular structures were prepared by using Chemcraft.

The reaction energy profiles and structural information for nucleophilic substitution steps

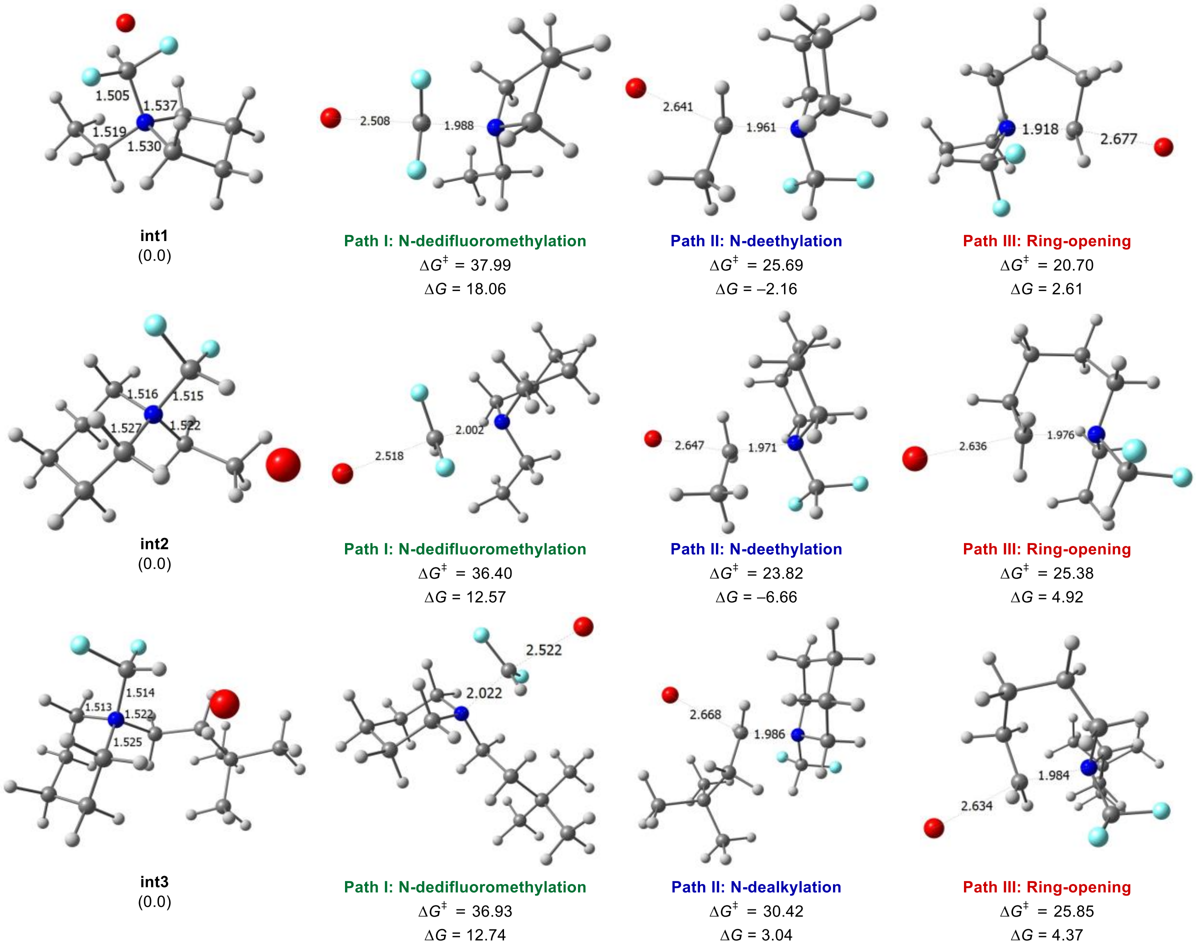

Supplementary Figure 2. The reaction energy profiles and bond lengths for three transition states of **int1–3**. The unit of Gibbs free energies is kcal/mol. The unit of bond length is angstrom (Å).

The Gibbs free energy profiles for subsequent hydrolysis process

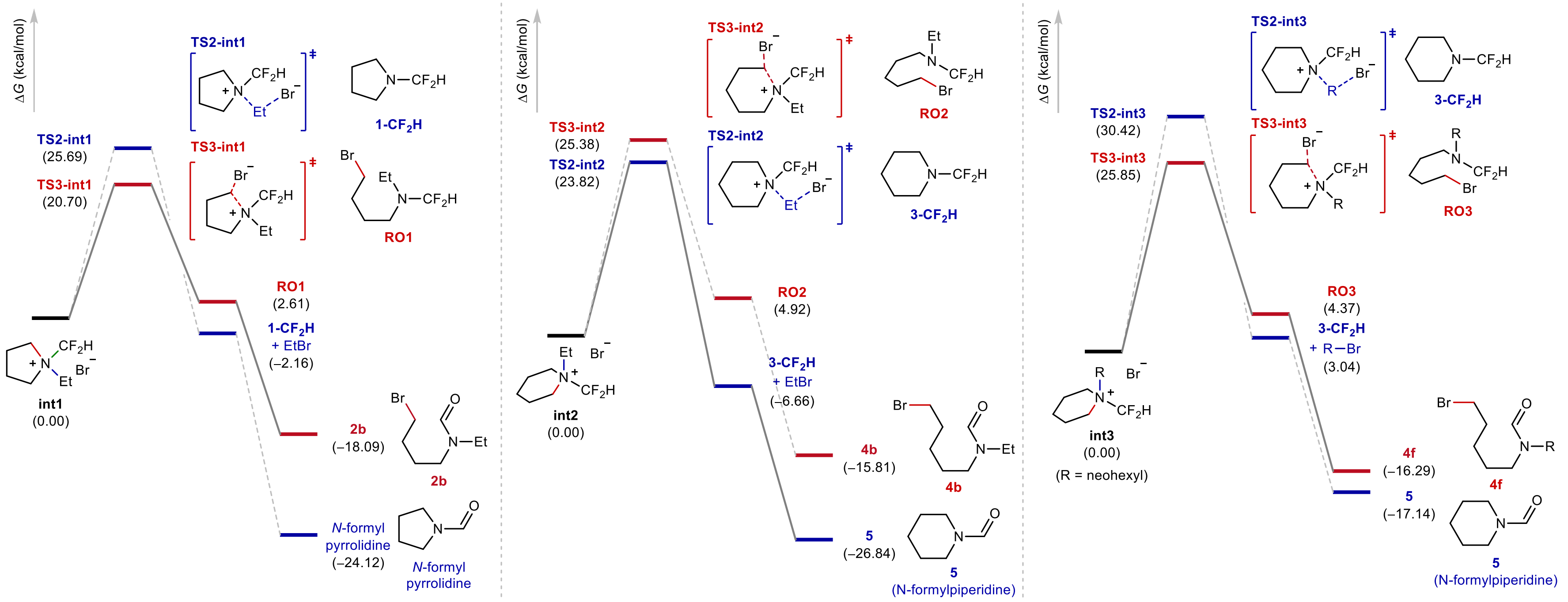

Supplementary Figure 3. The reaction energy profiles of subsequent hydrolysis process for **int1–3**. The unit of Gibbs free energies is kcal/mol.

Subsequent hydrolysis process of intermediates from N-dealkylation (blue) and Ring-opening (red) was examined to disclose the thermodynamics of overall reaction. The reaction energy profiles including the hydrolysis process for **int1–3** were illustrated in Supplementary Figure 2. Hydrolysis process of intermediates carrying the CF_2H group, which leads to the N-formyl product, was briefly calculated by adding one H_2O molecule along with the loss of two HF molecules. Followed by the nucleophilic attack step, the subsequent hydrolysis was calculated to be highly exergonic process by –21.96, –20.70, –20.18, –20.73, –20.18 and –20.66 kcal/mol, respectively {*N*-formylpyrrolidine and **2b** in **int1**, **5** (*N*-formylpiperidine) and **4b** in **int2**, and **5** (*N*-formylpiperidine) and **4f** in **int3**}. These calculation results indicate that the transformation of the CF_2H group into the formyl group make the overall reaction irreversible

Distortion-interaction analysis for N-deethylation vs ring-opening of int1

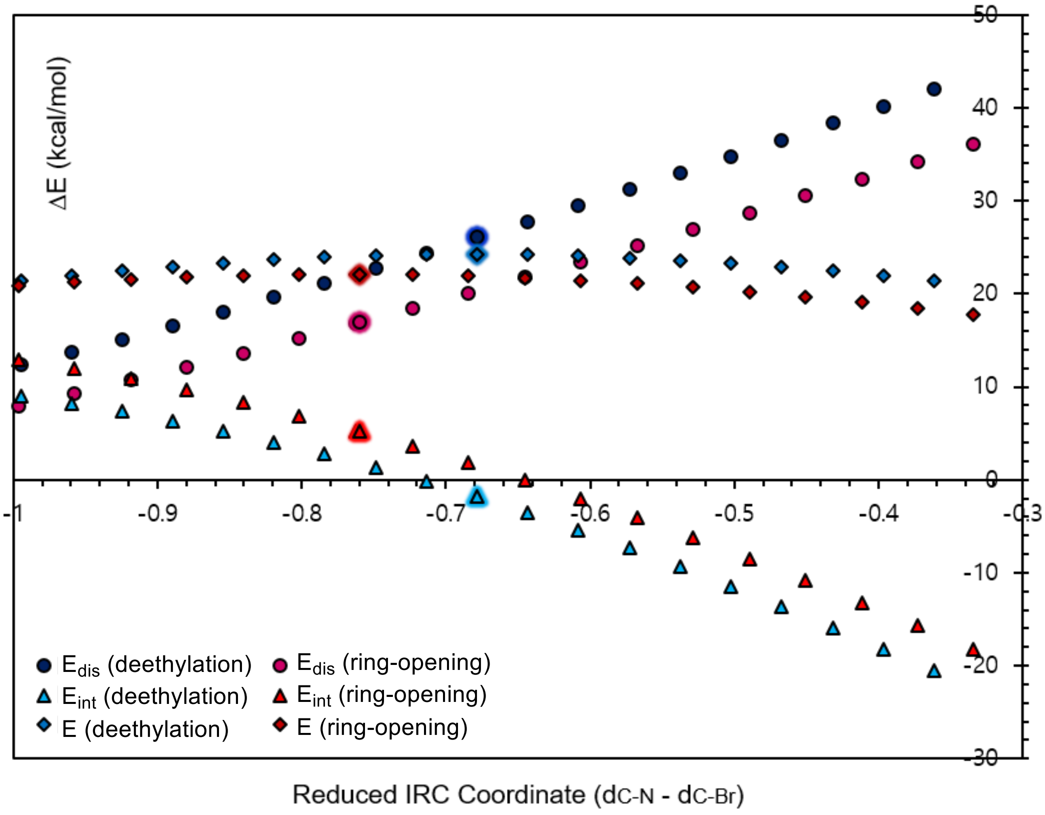

Supplementary Figure 4. Distortion-interaction analysis for N-deethylation vs ring opening of **int1**.

The *x*-axis is the reduced form of intrinsic reaction coordinate (IRC), calculated by the subtraction of broken bond ($d_{C\text{–}N}$) and forming bond ($d_{C\text{–}Br}$).[26] The *y*-axis is the relative electronic energy in kcal/mol. IRC calculations along with single-point calculations for each coordinate were performed with M06-2X/6-311++G(d,p) level of theory. The location of transition states is highlighted. The "blue curves" are plots related to N-deethylation process and "red curves" are plots related to ring-opening process. The blue and red "diamond" curves show that the electronic energy of N-deethylation is higher than ring-opening pathway along the IRC. While the blue and red "triangle" curves show relatively small differences in terms of the interaction energy, the blue and red "circle" curves show that the distortion energy terms in ring-opening process are significantly smaller than that of N-deethylation. Accordingly, the nucleophilic attack of bromide for ring-opening involves the least structural reorganization in the transition state, and this result is in good agreement with the lowest barrier in ring-opening process, as aforementioned in the reaction energy profile (Supplementary Figure 2).

Distortion-interaction analysis for N-dealkylation vs ring-opening of int2 and int3

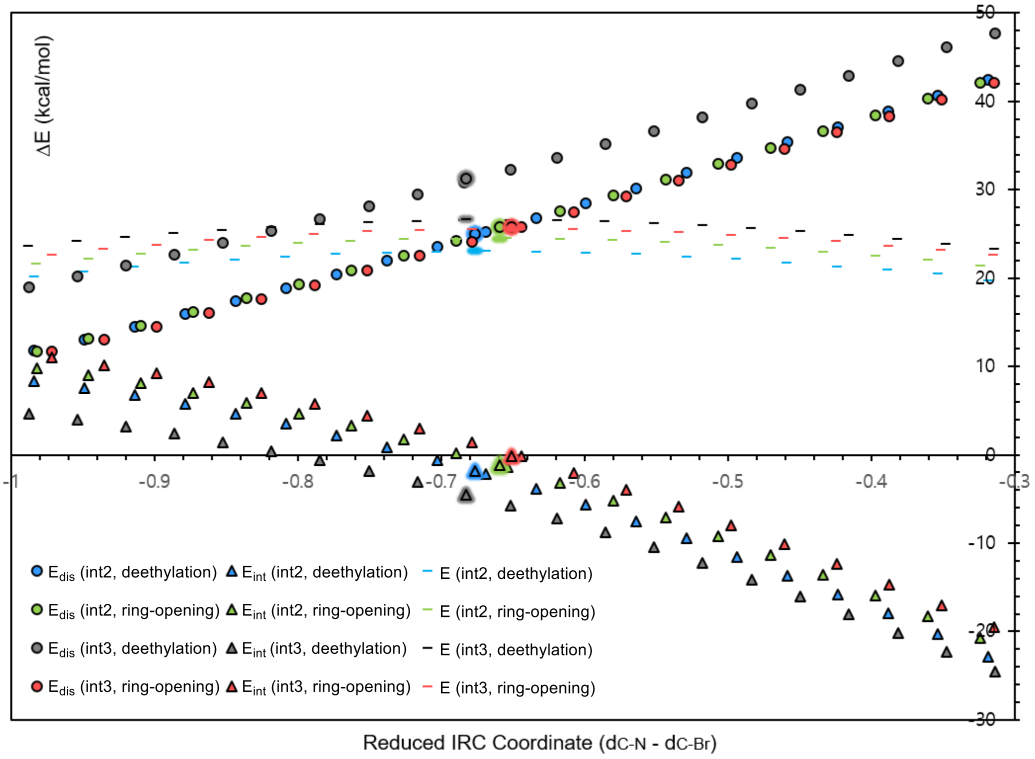

Supplementary Figure 5. Distortion-interaction analysis for N-dealkylation vs ring opening of **int2** and **int3**.

The *x*-axis is the reduced form of intrinsic reaction coordinate (IRC), calculated by the subtraction of broken bond ($d_{C\text{–}N}$) and forming bond ($d_{C\text{–}Br}$). The *y*-axis is the relative electronic energy in kcal/mol. IRC calculations along with single-point calculations for each coordinate were performed with M06-2X/6-311++G(d,p) level of theory. The location of transition states is highlighted. The "blue curves" are plots related to N-deethylation process (Path II) and "green curves" are plots related to ring-opening process (Path III) in case of int3. The "grey curves" are plots related to N-dealkylation process (Path II) and "red curves" are plots related to ring-opening process (Path III) in case of int4

Spectral Copies of ^{1}H, ^{13}C and ^{19}F NMR of Novel Compounds Obtained in this Study

5-Tosyl-1,2,3,3a,4,5-hexahydropyrrolo[1,2-a]quinoxaline (1o)

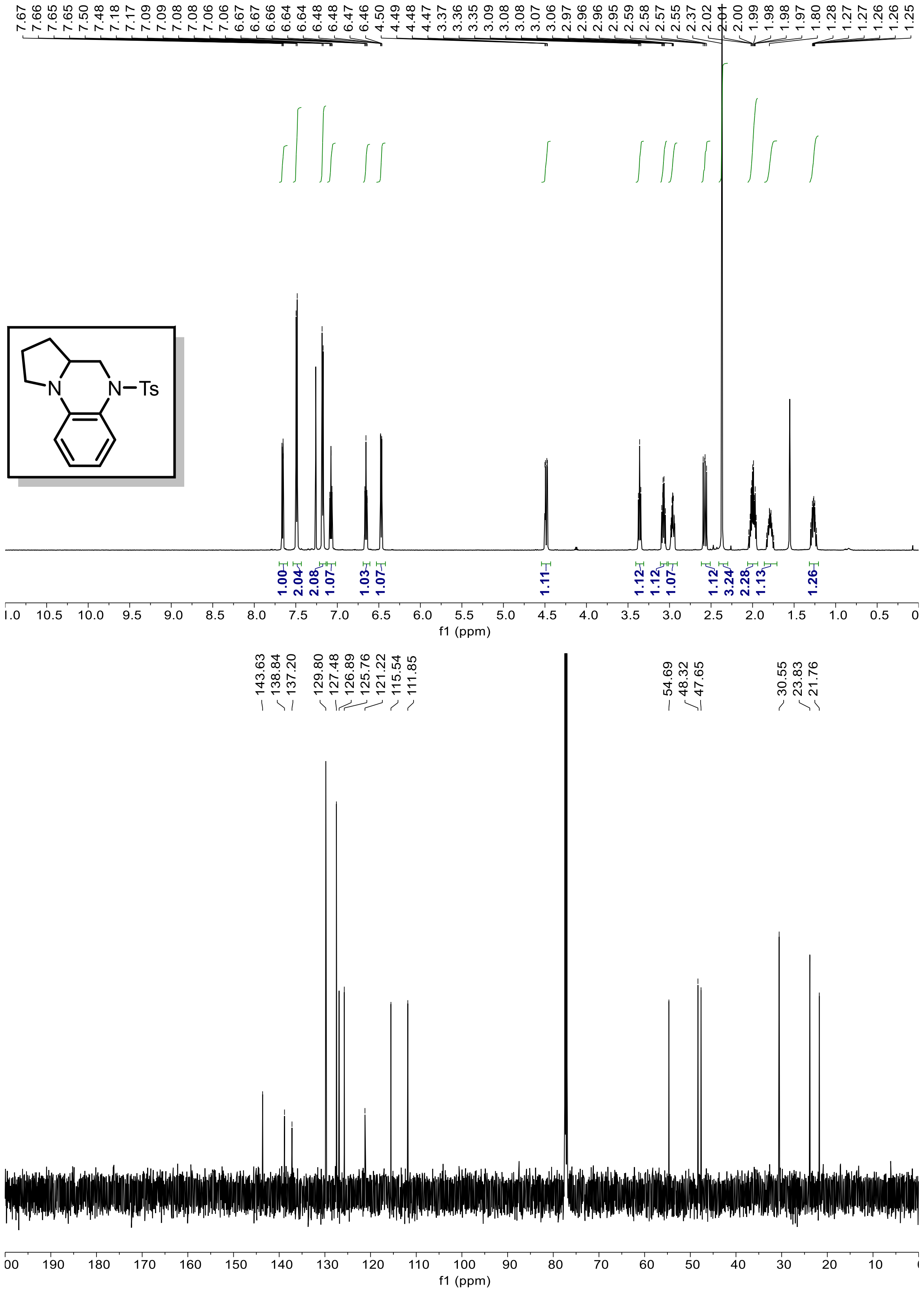

Supplementary Figure 6. ^{1}H and ^{13}C NMR spectra of **1o**.

1-(4-Methoxyphenyl)-3-phenylpyrrolidine (1p)

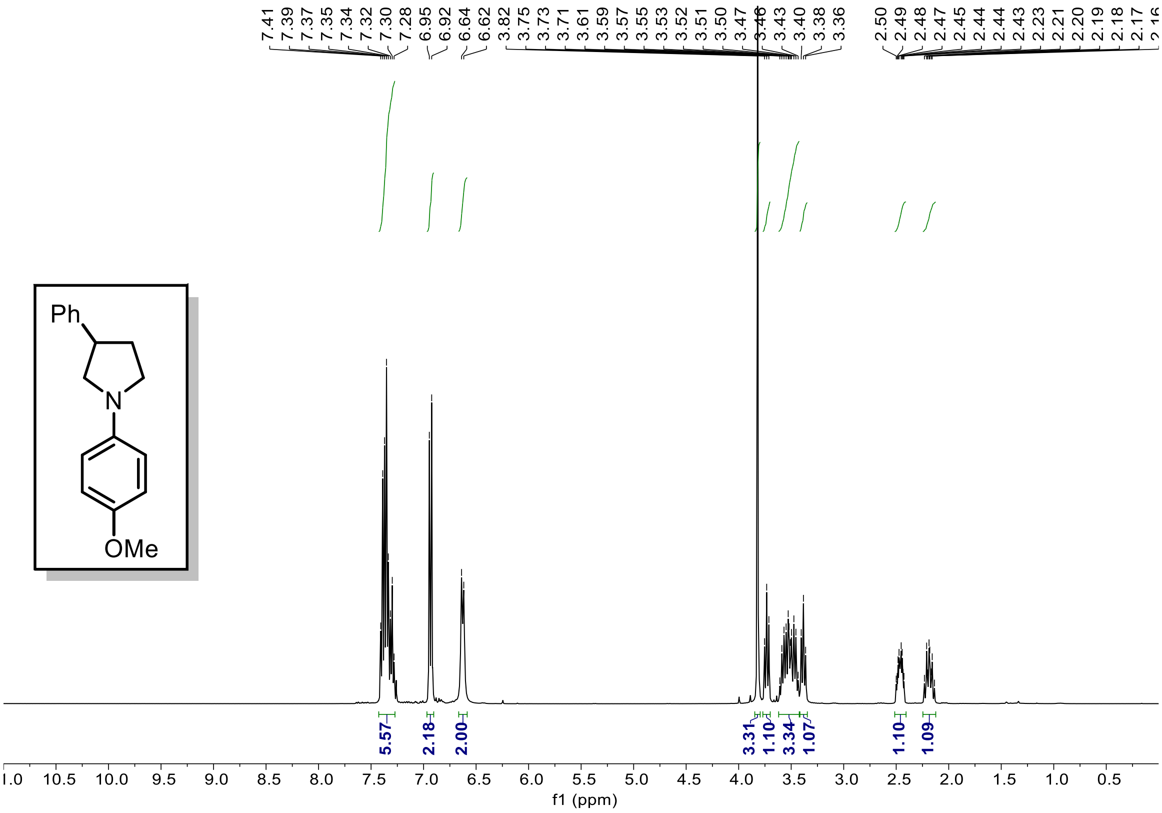

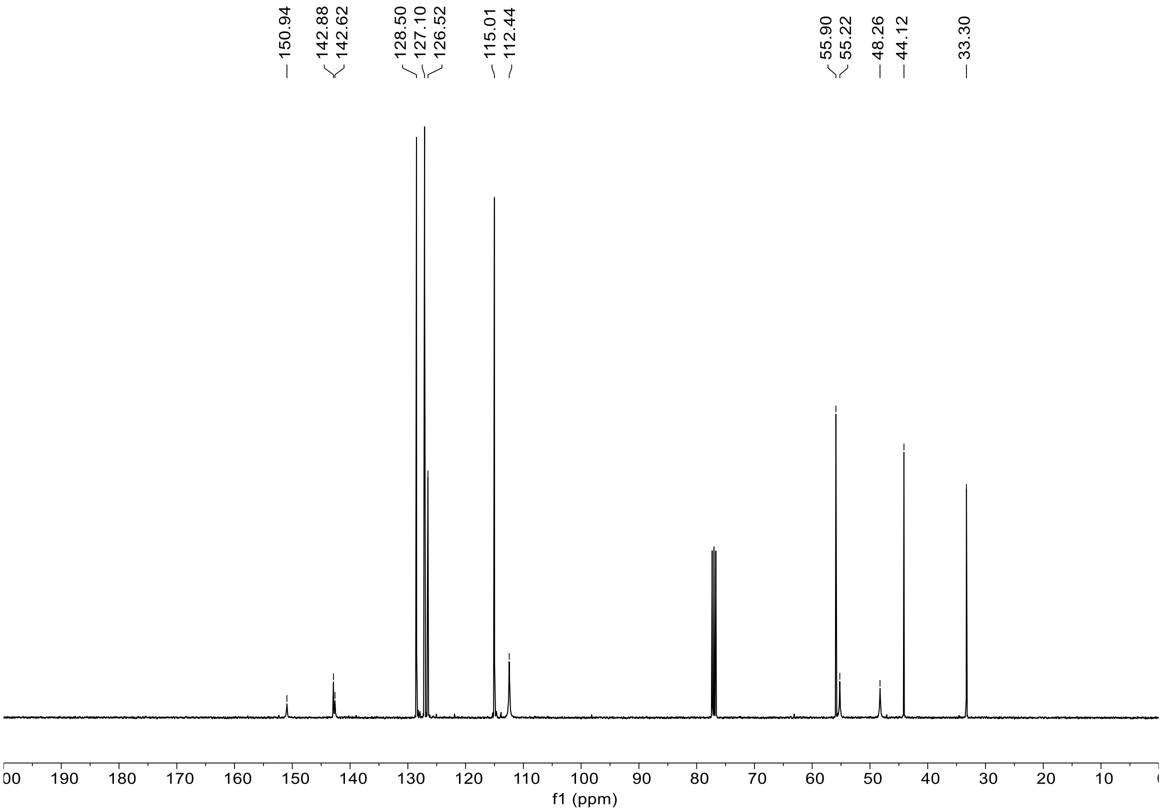

Supplementary Figure 7. ^{1}H and ^{13}C NMR spectra of **1p**.

2-Phenyl-2-azaspiro[4.5]decan-3-one

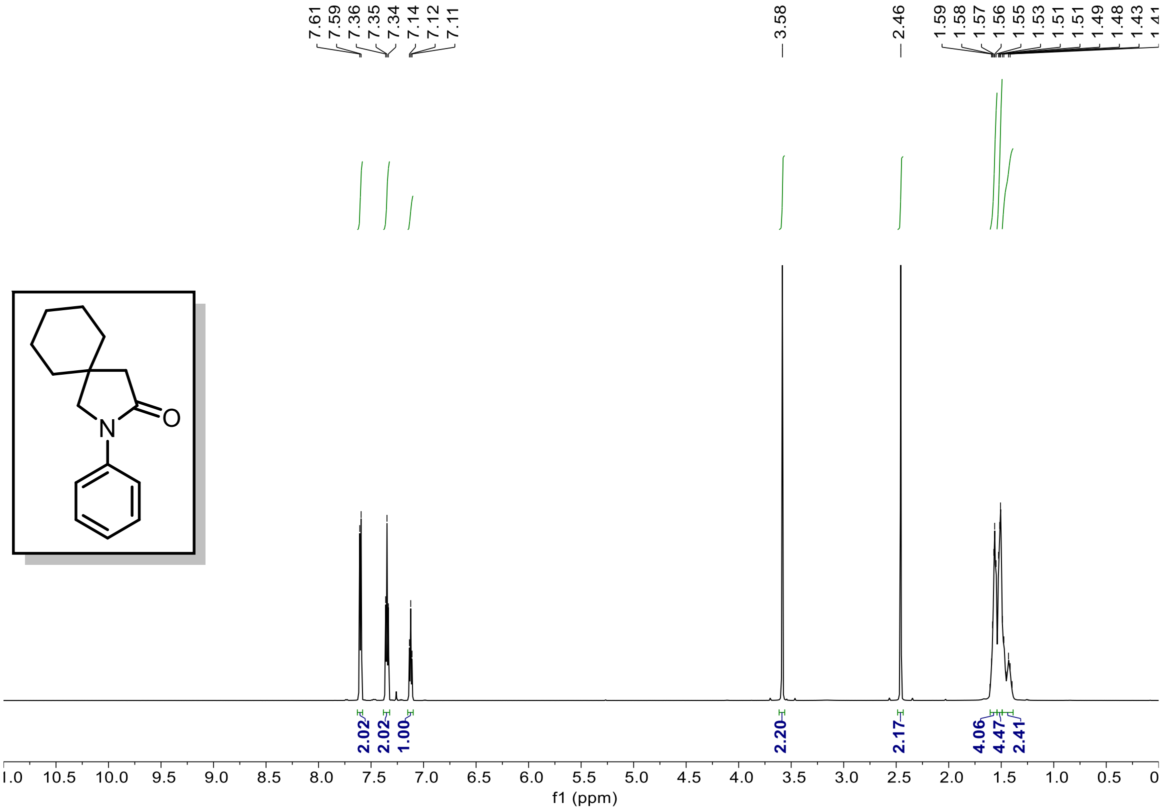

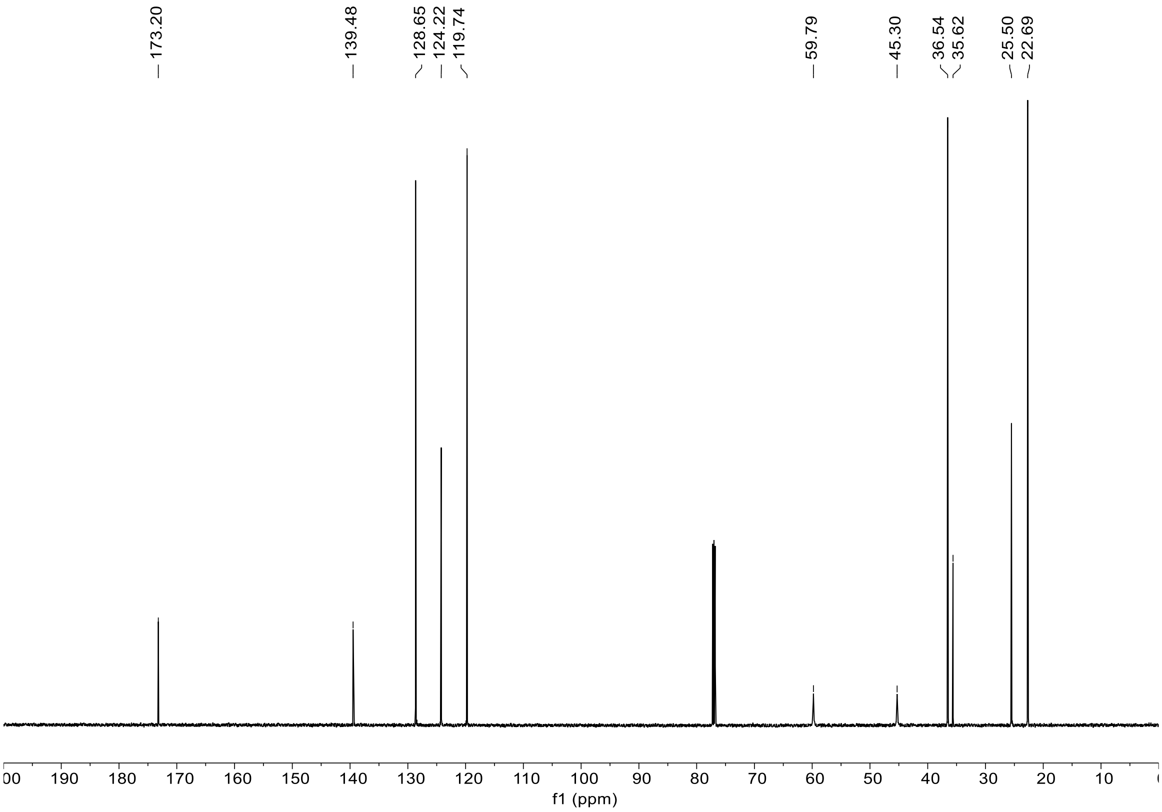

Supplementary Figure 8. ^{1}H and ^{13}C NMR spectra of 2-phenyl-2-azaspiro[4.5]decan-3-one.

2-Phenyl-2-azaspiro[4.5]decane (1q)

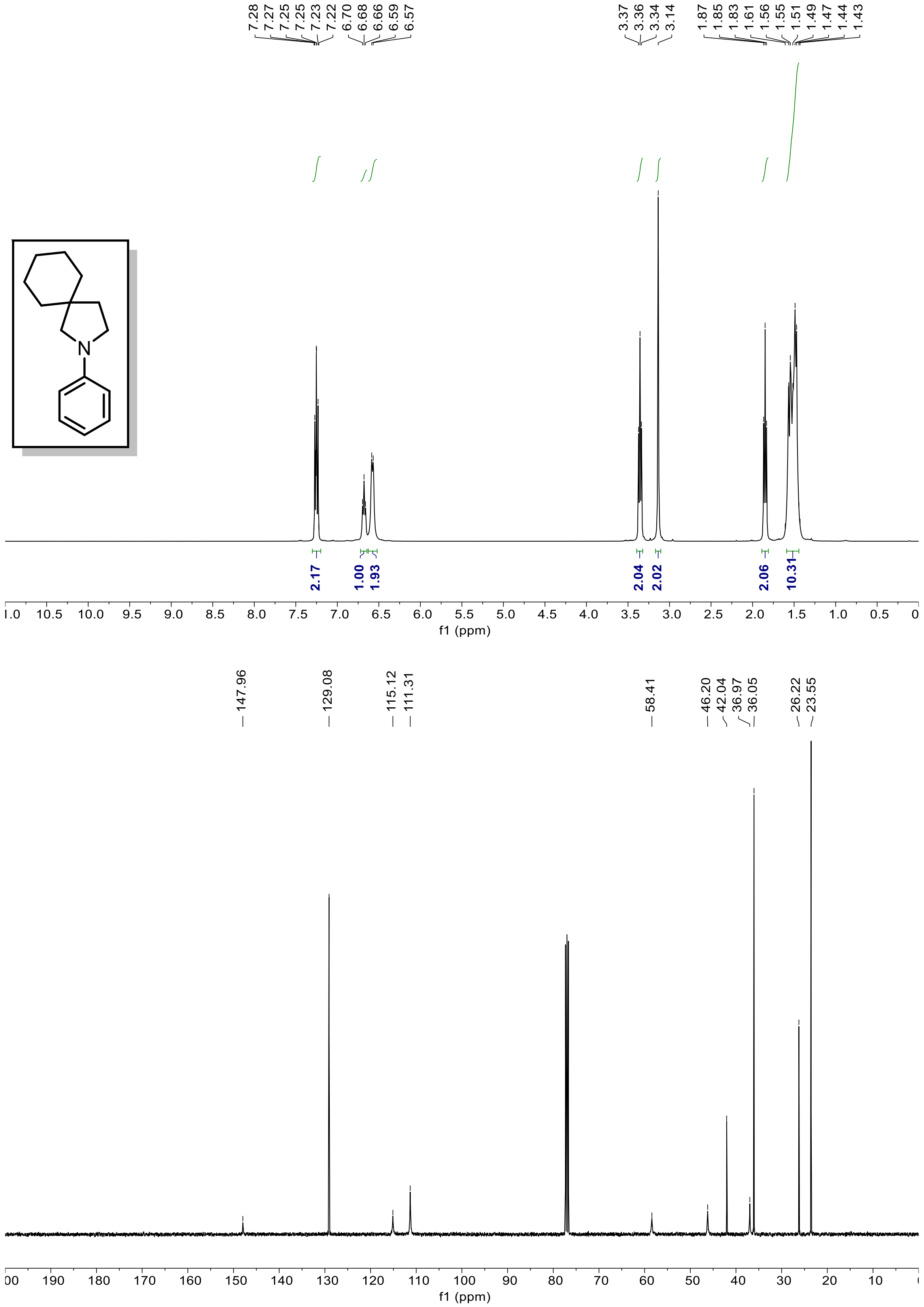

Supplementary Figure 9. ^{1}H and ^{13}C NMR spectra of **1q**.

(*S*)-(1-Methylpyrrolidin-2-yl)methyl acetate (1r)

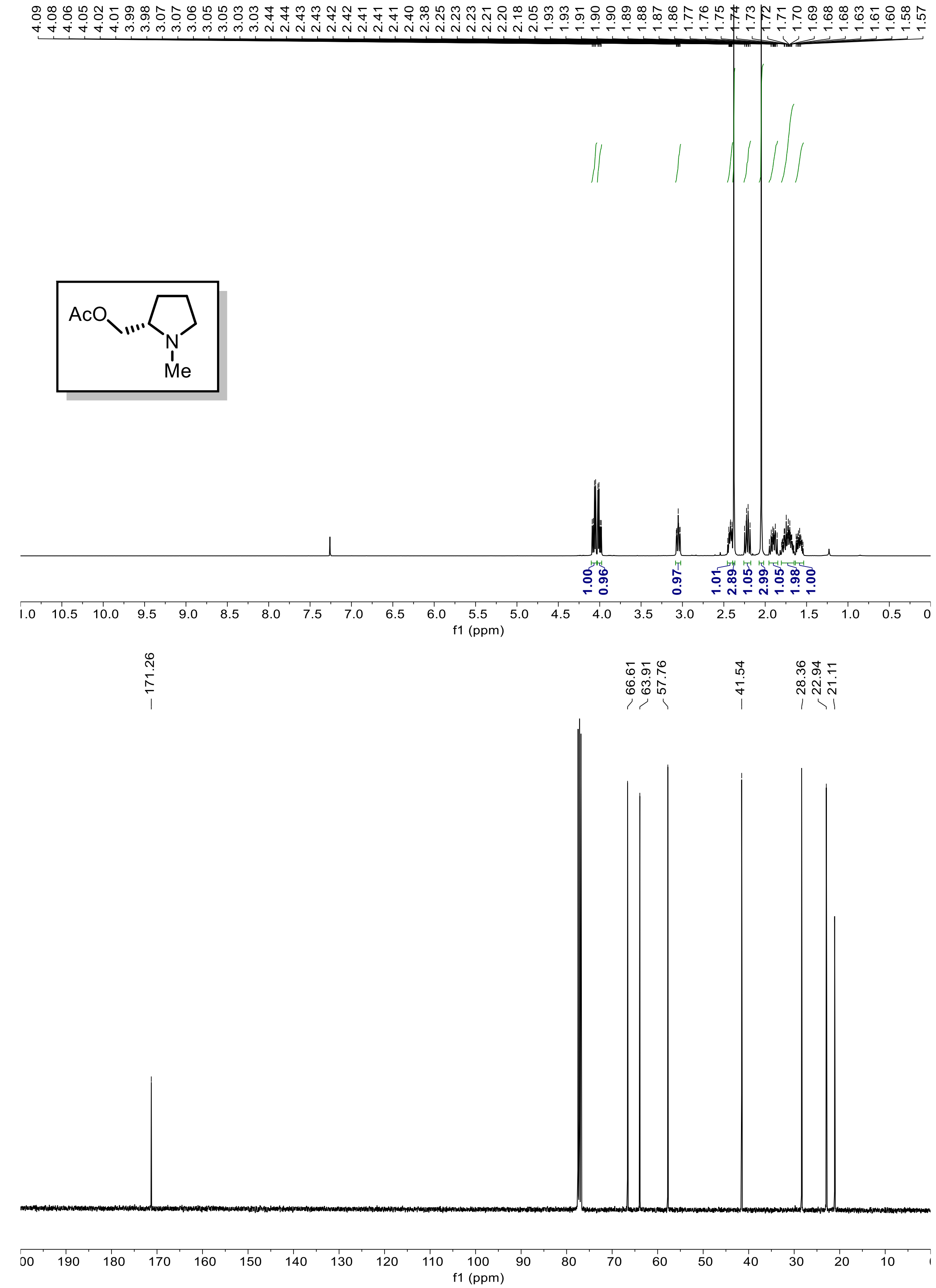

Supplementary Figure 10. ^{1}H and ^{13}C NMR spectra of **1r**.

8-Phenyl-8-azaspiro[4.5]decane-7,9-dione

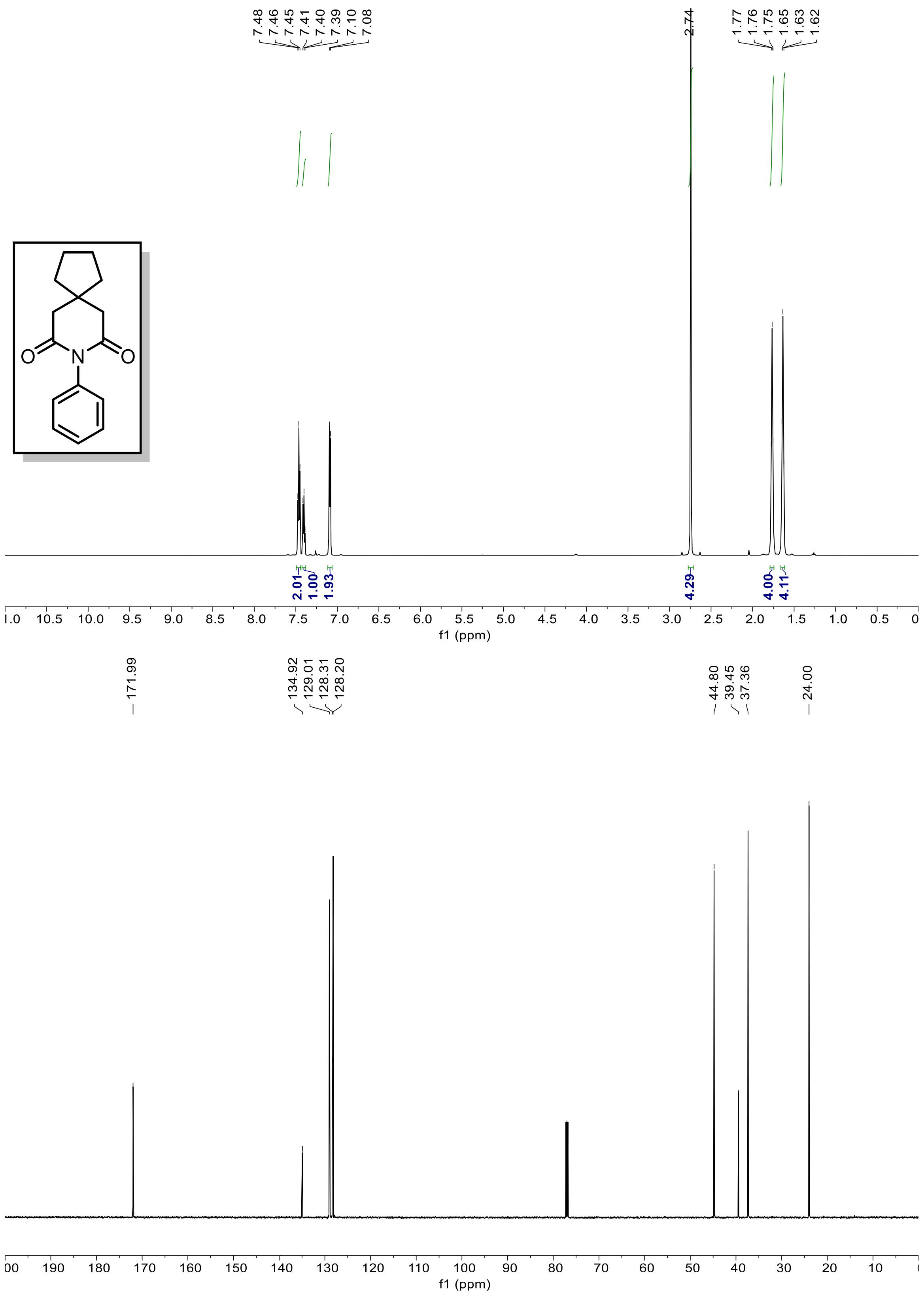

Supplementary Figure 11. ^{1}H and ^{13}C NMR spectra of 8-phenyl-8-azaspiro[4.5]decane-7,9-dione.

8-Phenyl-8-azaspiro[4.5]decane (3i)

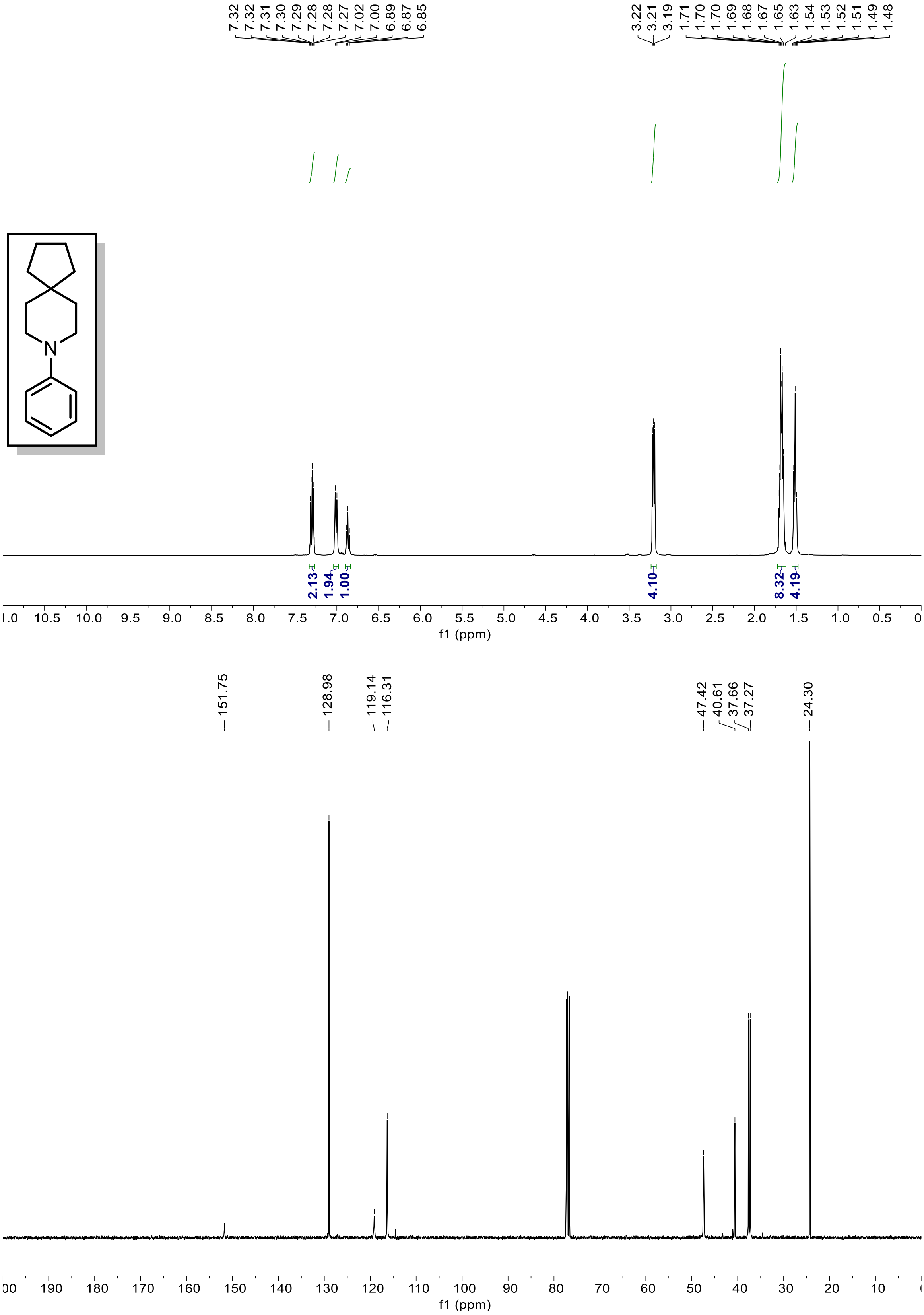

Supplementary Figure 12. ^{1}H and ^{13}C NMR spectra of **3i**.

4-(3,3-Dimethylbutyl)morpholine (3k)

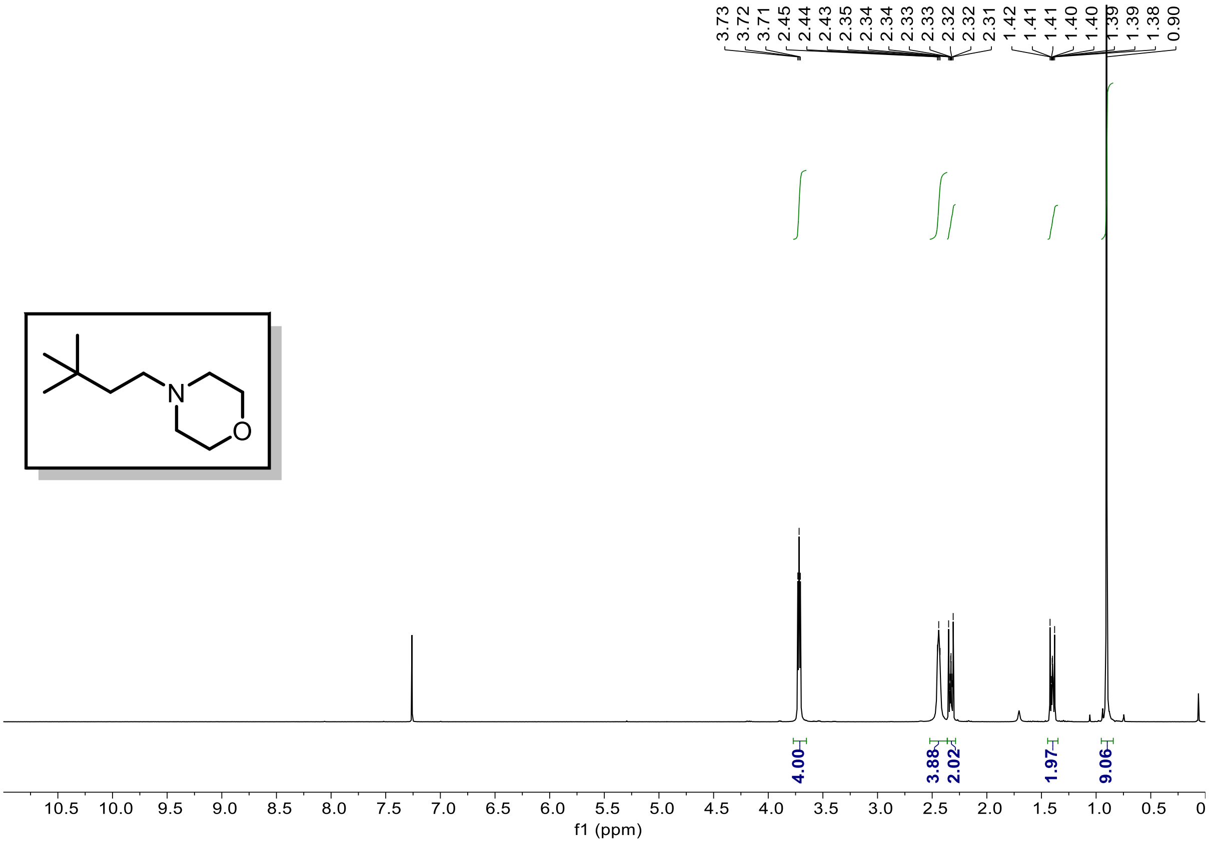

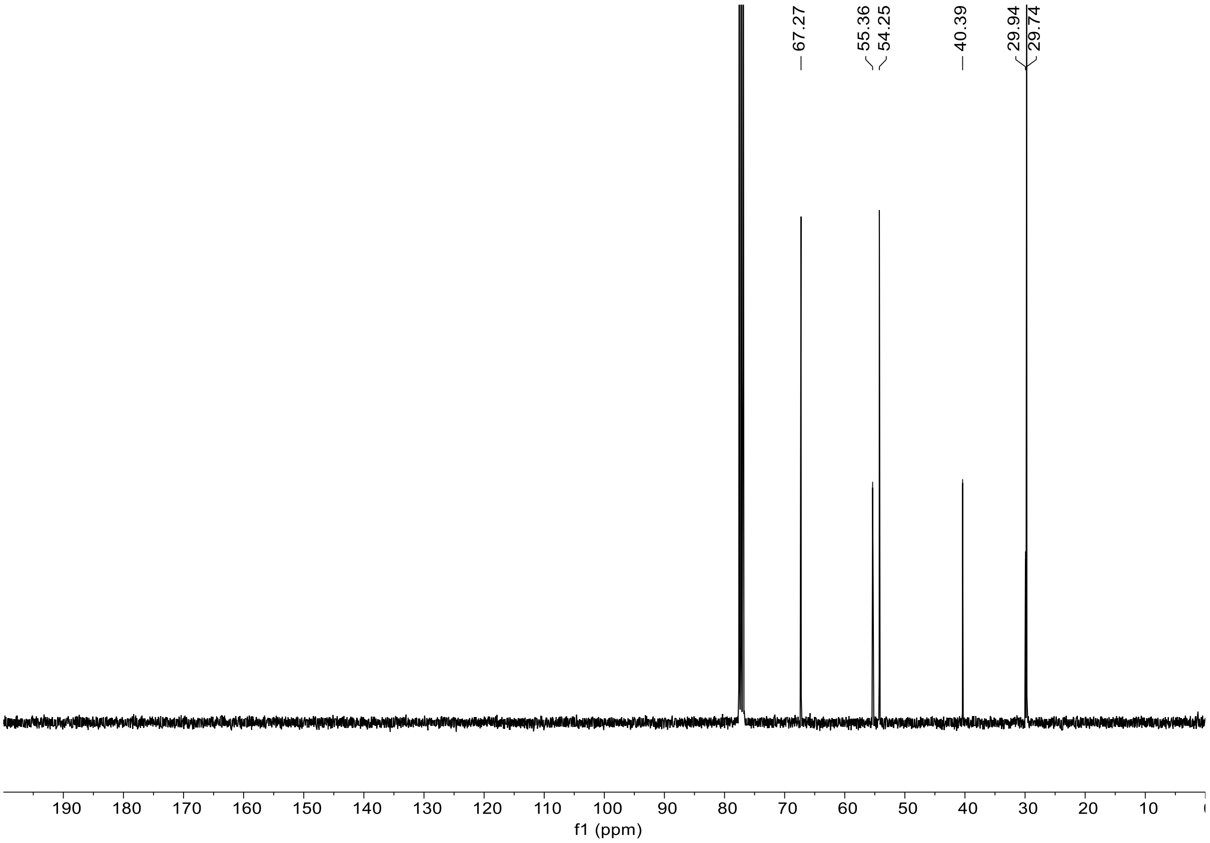

Supplementary Figure 13. ^{1}H and ^{13}C NMR spectra of **3k**.

1-Butyl-2-phenylpiperidine (3r)

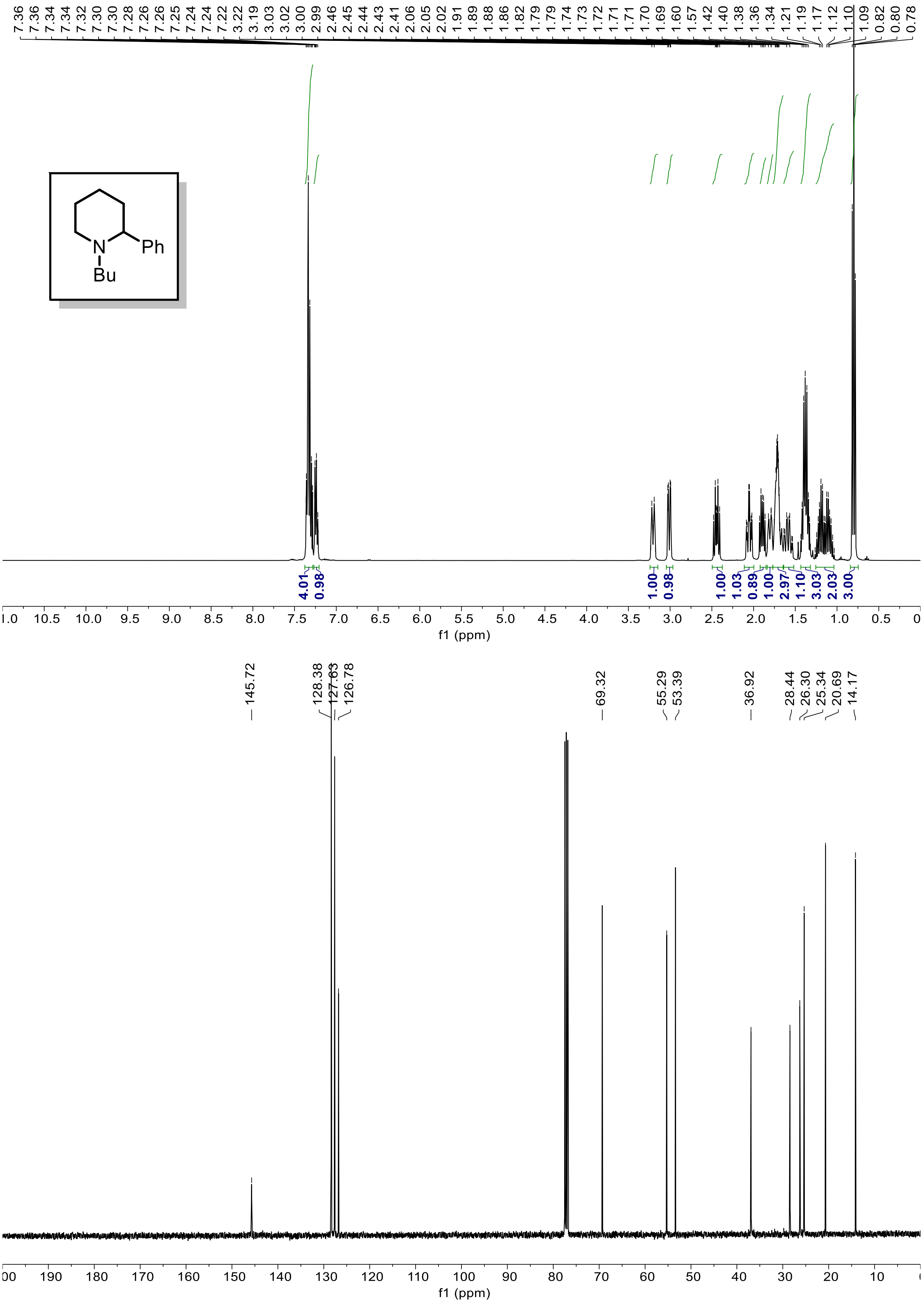

Supplementary Figure 14. ^{1}H and ^{13}C NMR spectra of **3r**.

1-Butylazocane (8b)

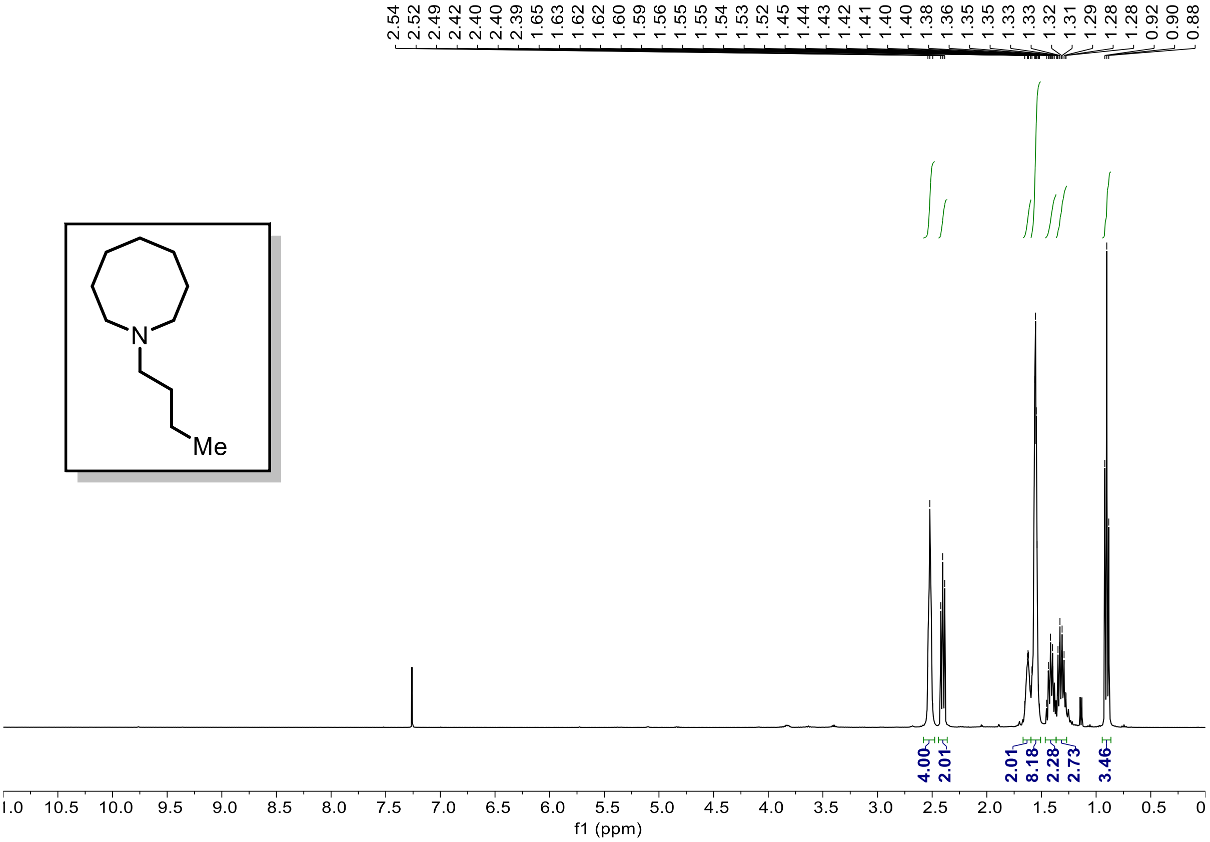

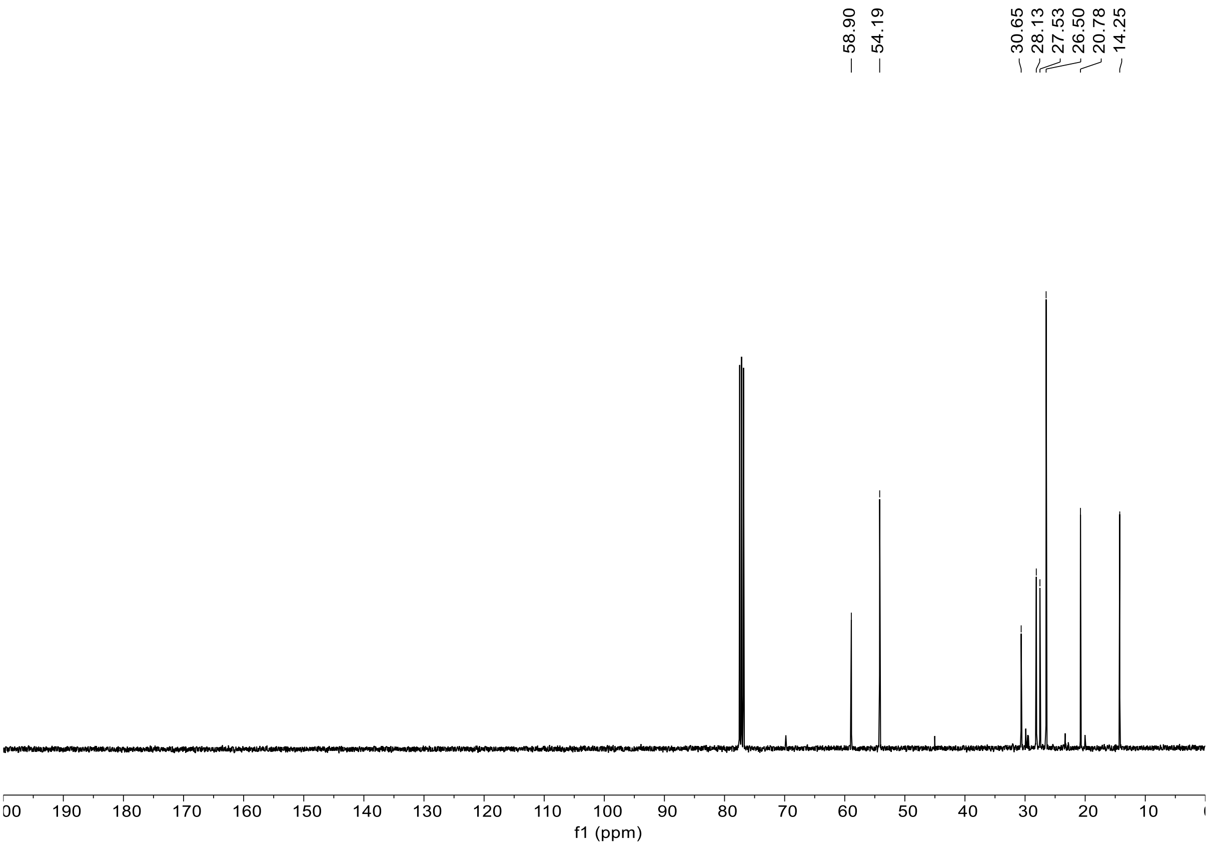

Supplementary Figure 15. ^{1}H and ^{13}C NMR spectra of **8b**.

(*S*)-4-(3,7-Dimethyloct-6-en-1-yl)morpholine

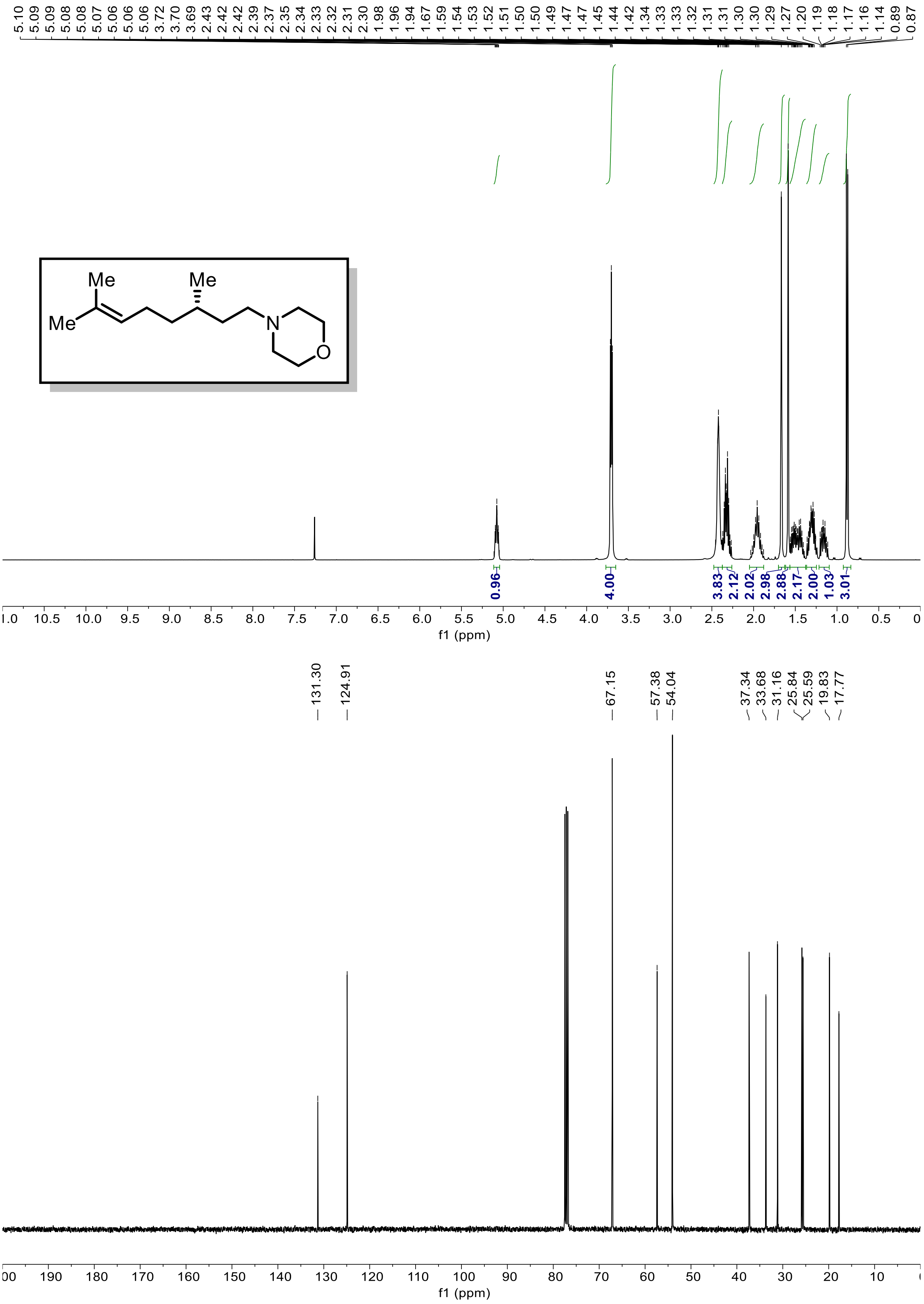

Supplementary Figure 16. ^{1}H and ^{13}C NMR spectra of (*S*)-4-(3,7-dimethyloct-6-en-1-yl)morpholine.

***N*-(Difluoromethyl)-*N*-ethylpyrrolidinium bromide (int1)**

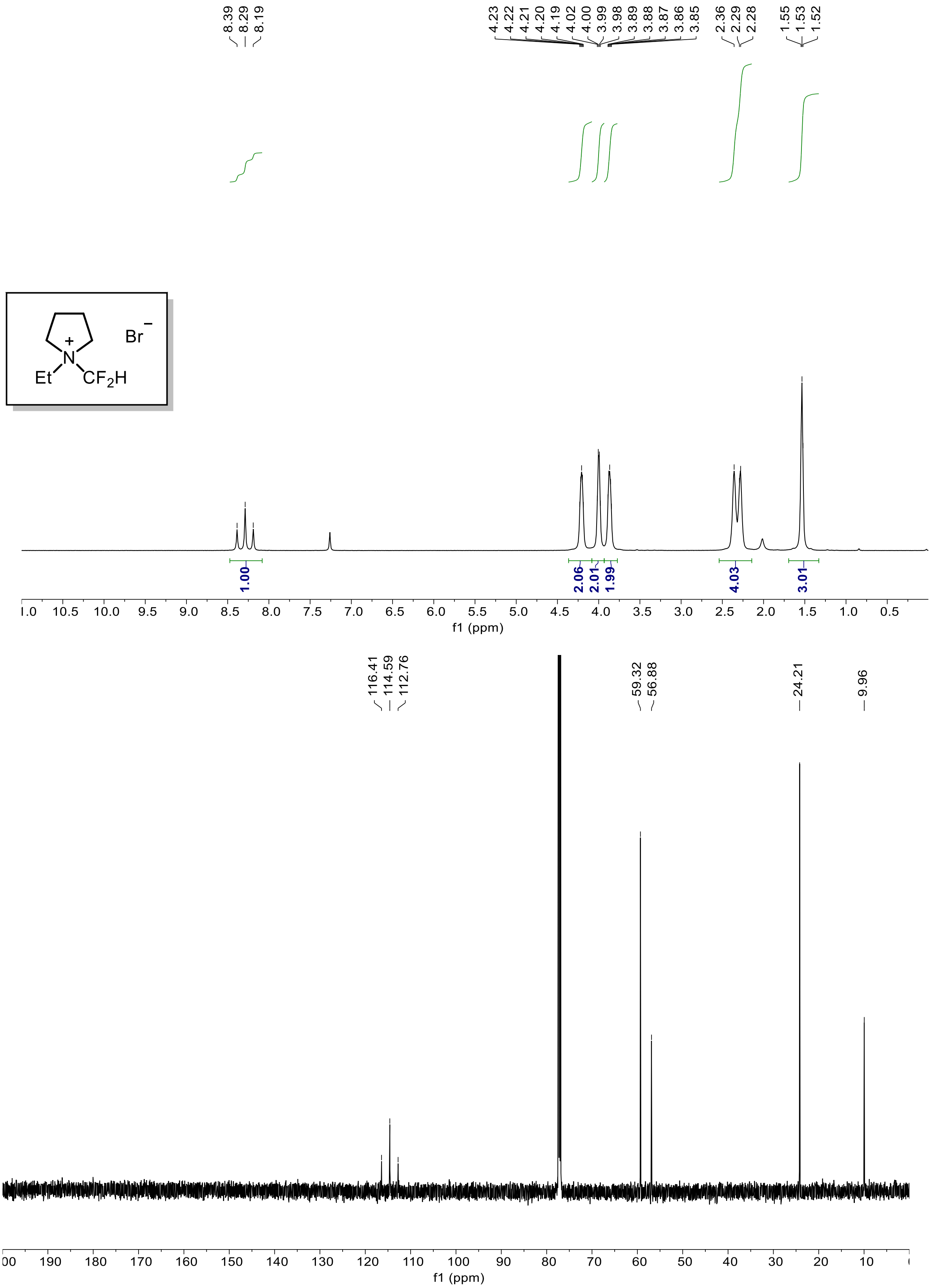

Supplementary Figure 17. ^{1}H and ^{13}C NMR spectra of **int1**.

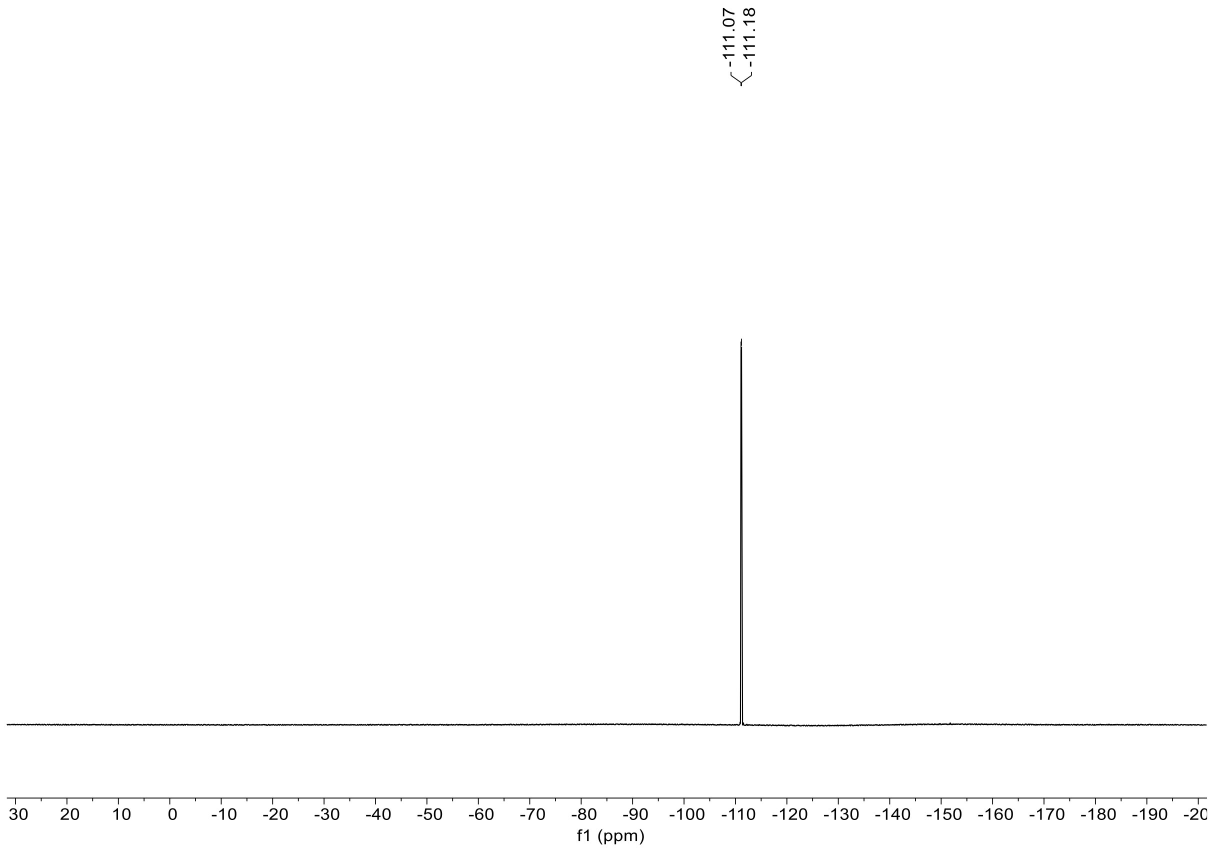

Supplementary Figure 18. ^{19}F NMR spectra of **int1**.

***N*-(Difluoromethyl)-*N*-ethylpiperidinium bromide (int2)**

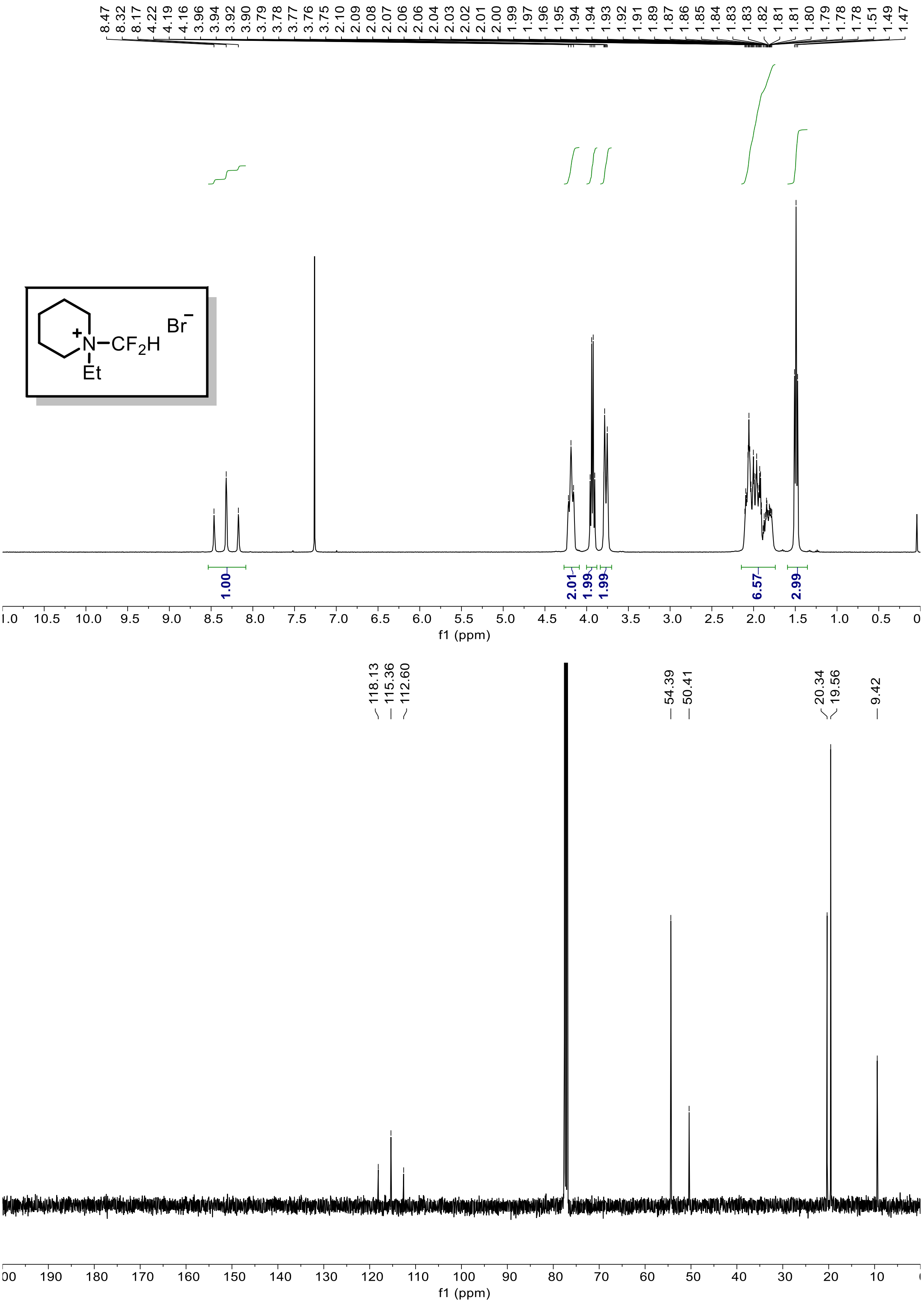

Supplementary Figure 19. ^{1}H and ^{13}C NMR spectra of **int2**.

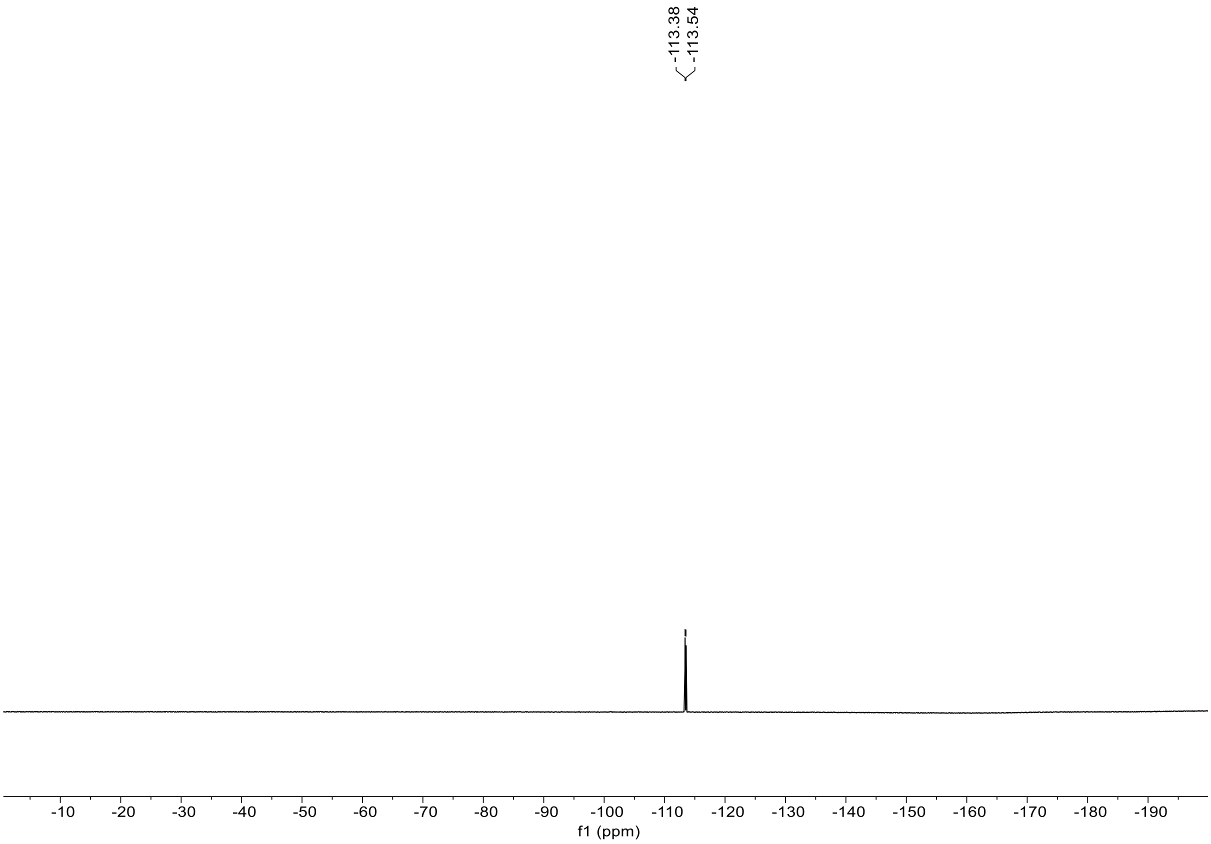

Supplementary Figure 20. ^{19}F NMR spectra of **int2**.

***N*-(Difluoromethyl)-*N*-(3,3-dimethylbutyl)piperidinium bromide (int3)**

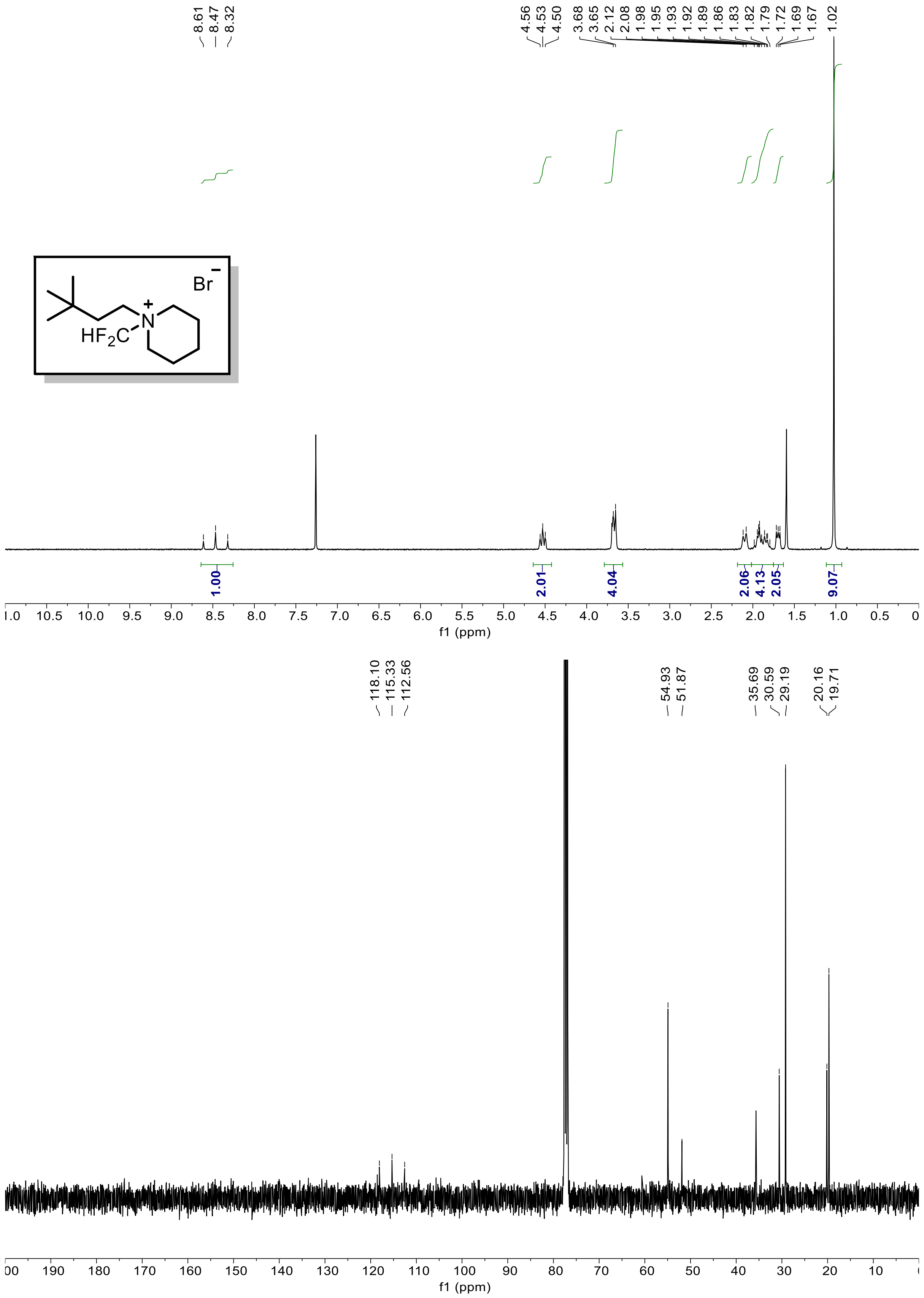

Supplementary Figure 21. ^{1}H and ^{13}C NMR spectra of **int3**.

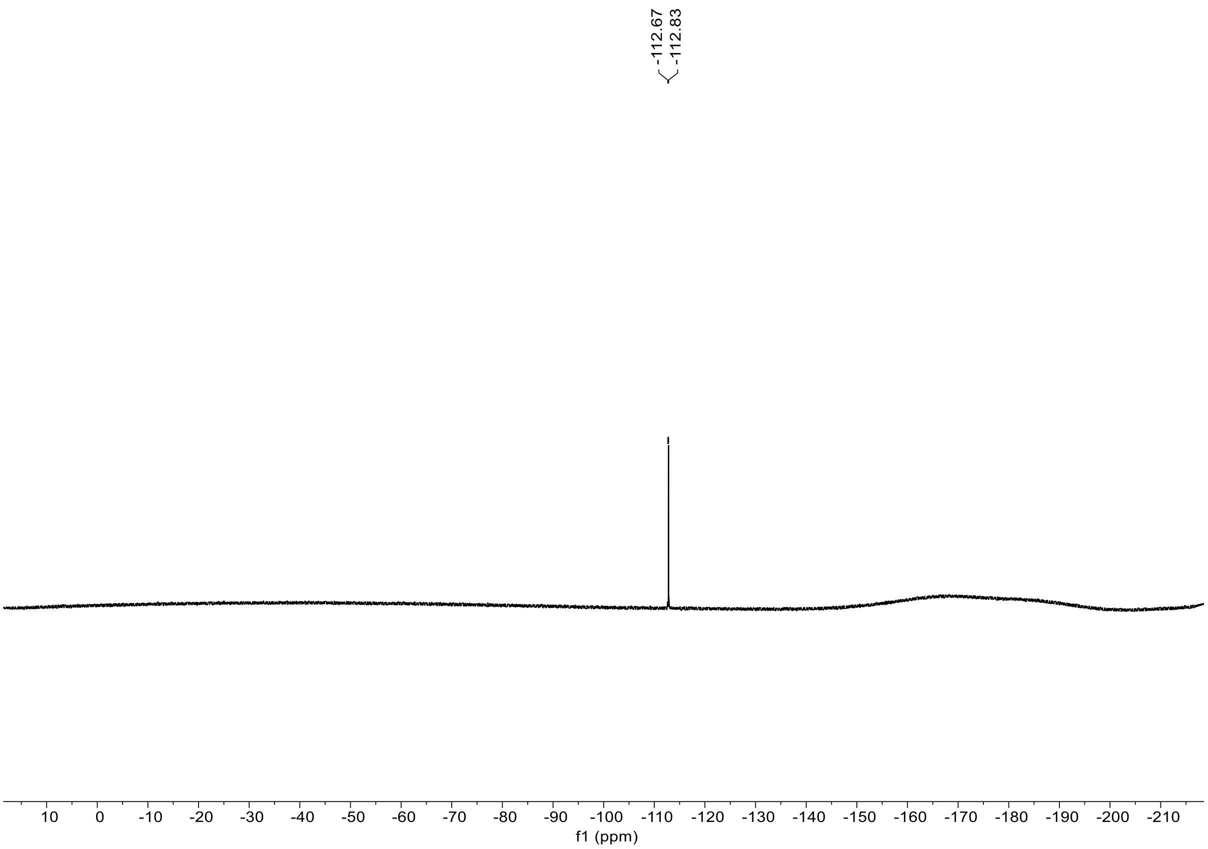

Supplementary Figure 22. ^{19}F NMR spectra of **int3**.

***N*-(4-Bromobutyl)-*N*-phenylformamide (2a)**

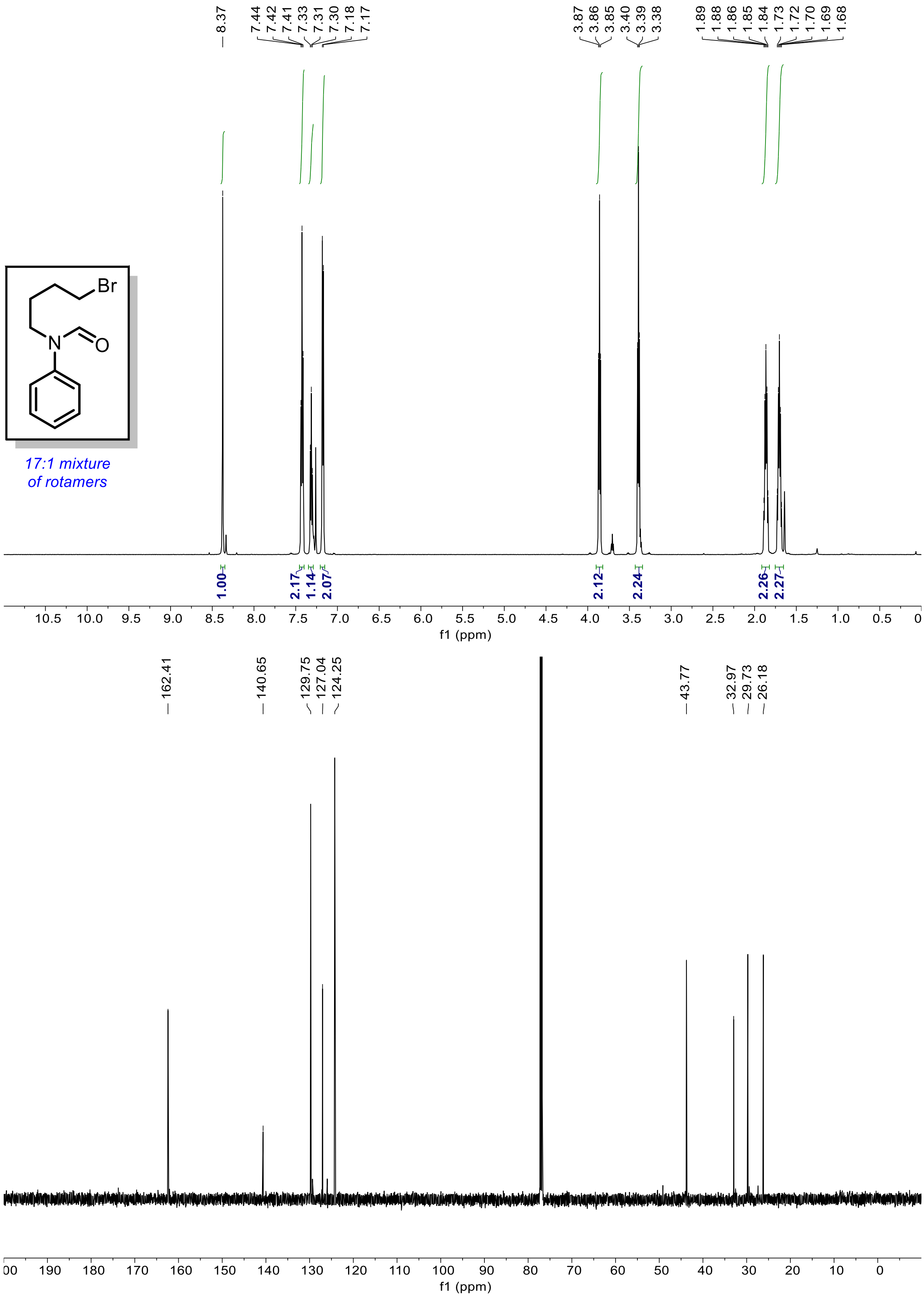

Supplementary Figure 23. ^{1}H and ^{13}C NMR spectra of **2a**.

***N*-(4-Bromobutyl)-*N*-ethylformamide (2b)**

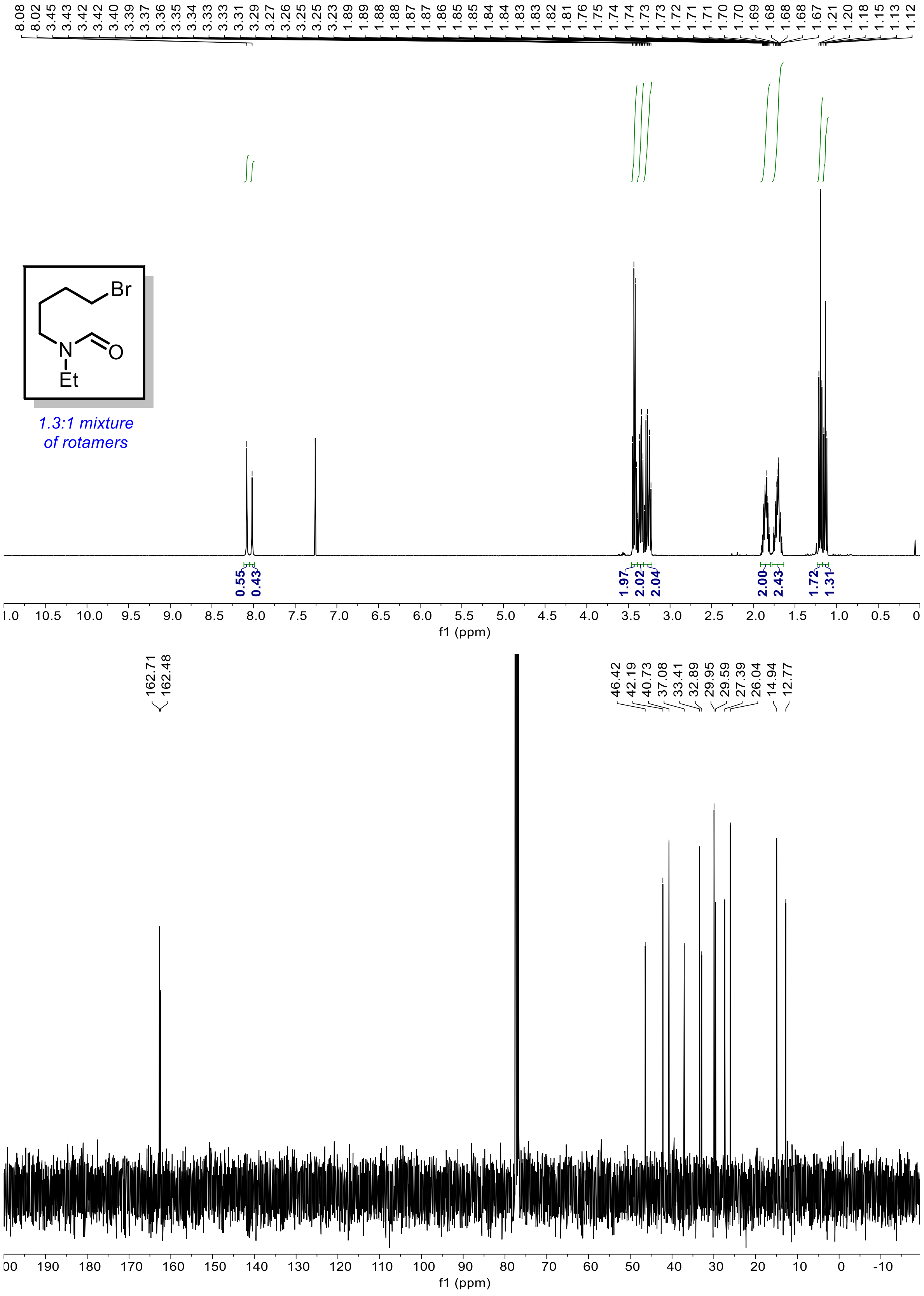

Supplementary Figure 24. ^{1}H and ^{13}C NMR spectra of **2b**.

***N*-(4-Bromobutyl)-*N*-methylformamide (2c)**

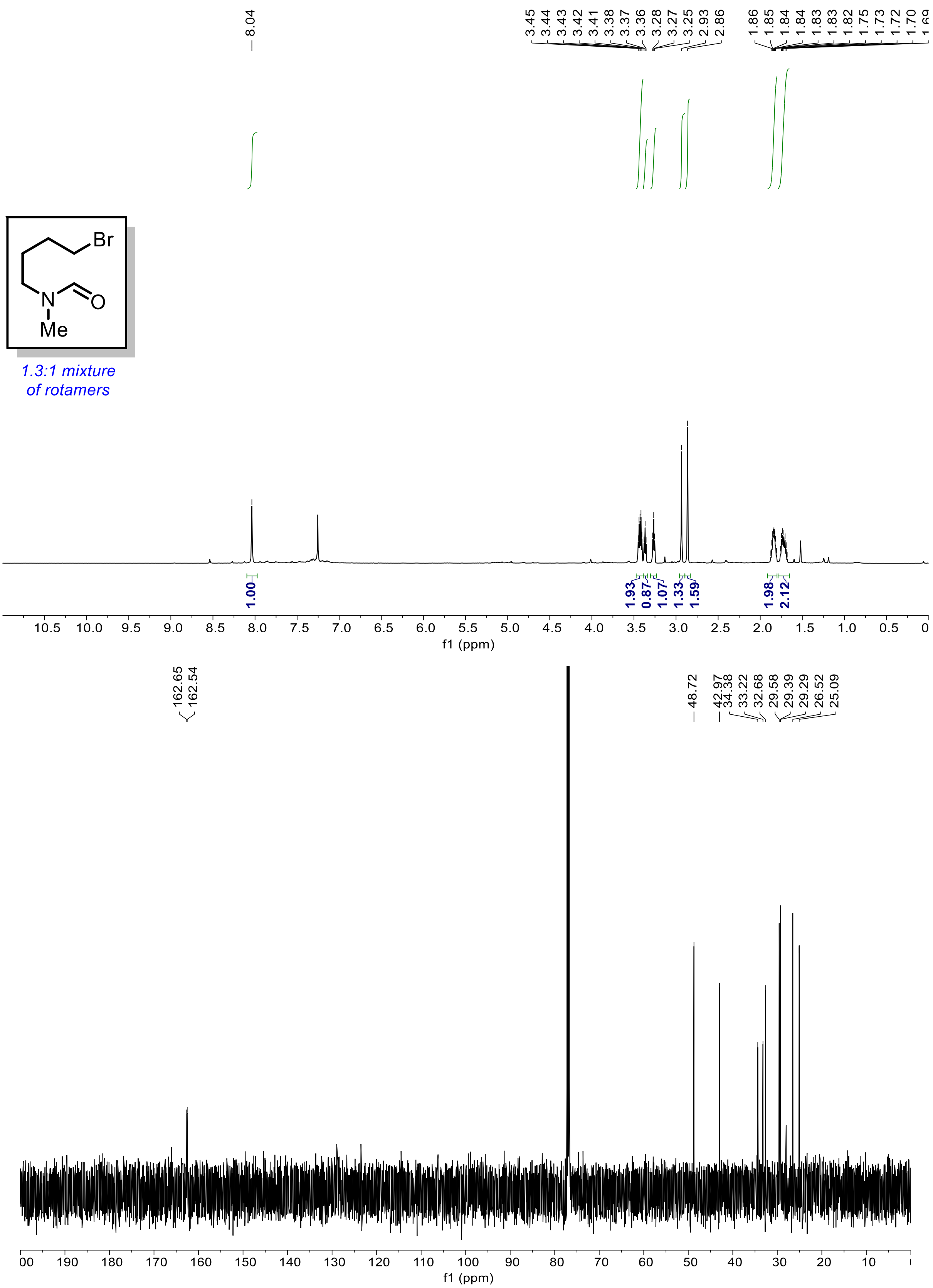

Supplementary Figure 25. ^{1}H and ^{13}C NMR spectra of **2c**.

N-(4-Bromobutyl)-*N*-(4-methoxyphenyl)formamide (2d)

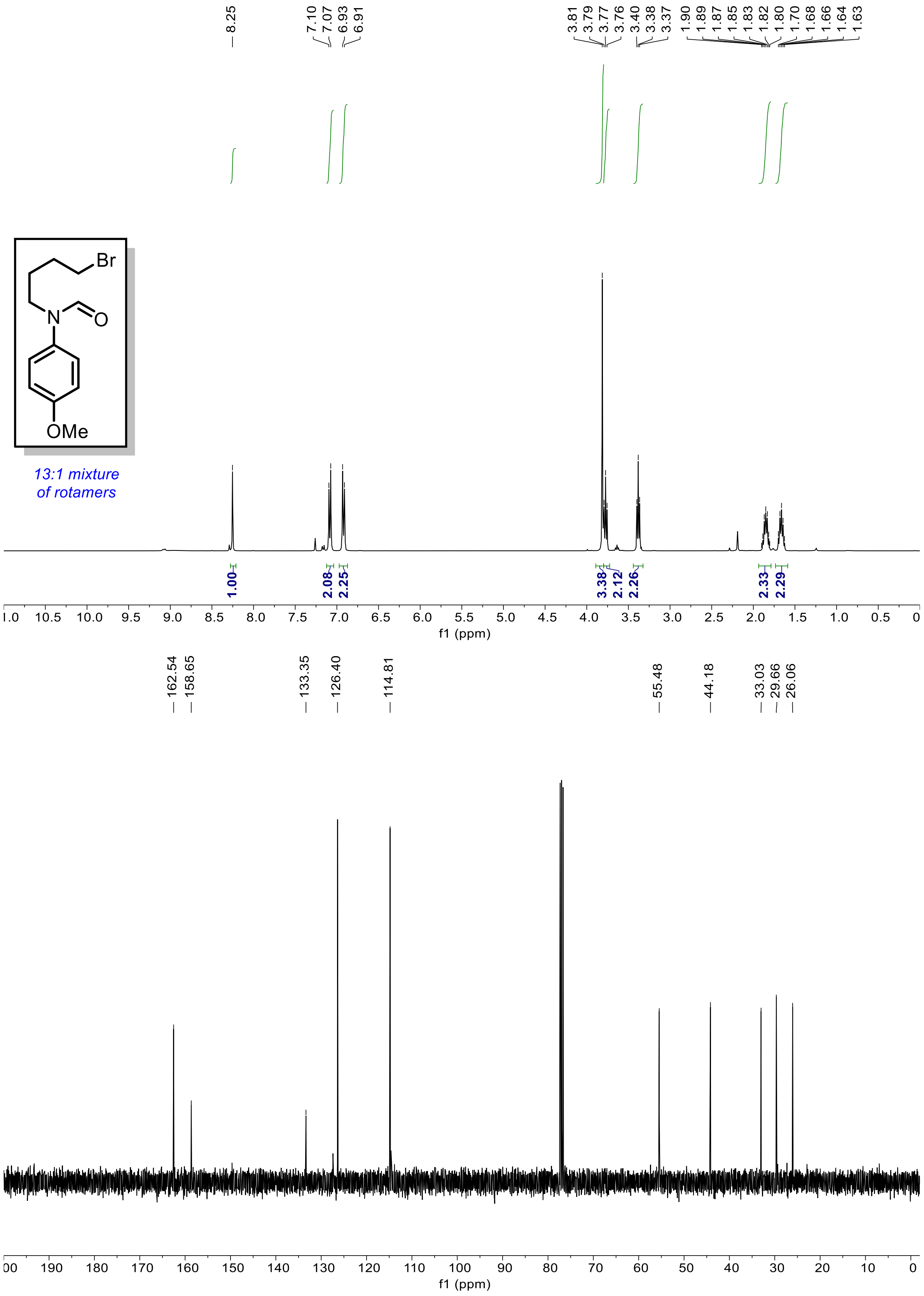

Supplementary Figure 26. ^{1}H and ^{13}C NMR spectra of **2d**.

***N*-(4-Bromobutyl)-*N*-[4-(trifluoromethyl)phenyl]formamide (2e)**

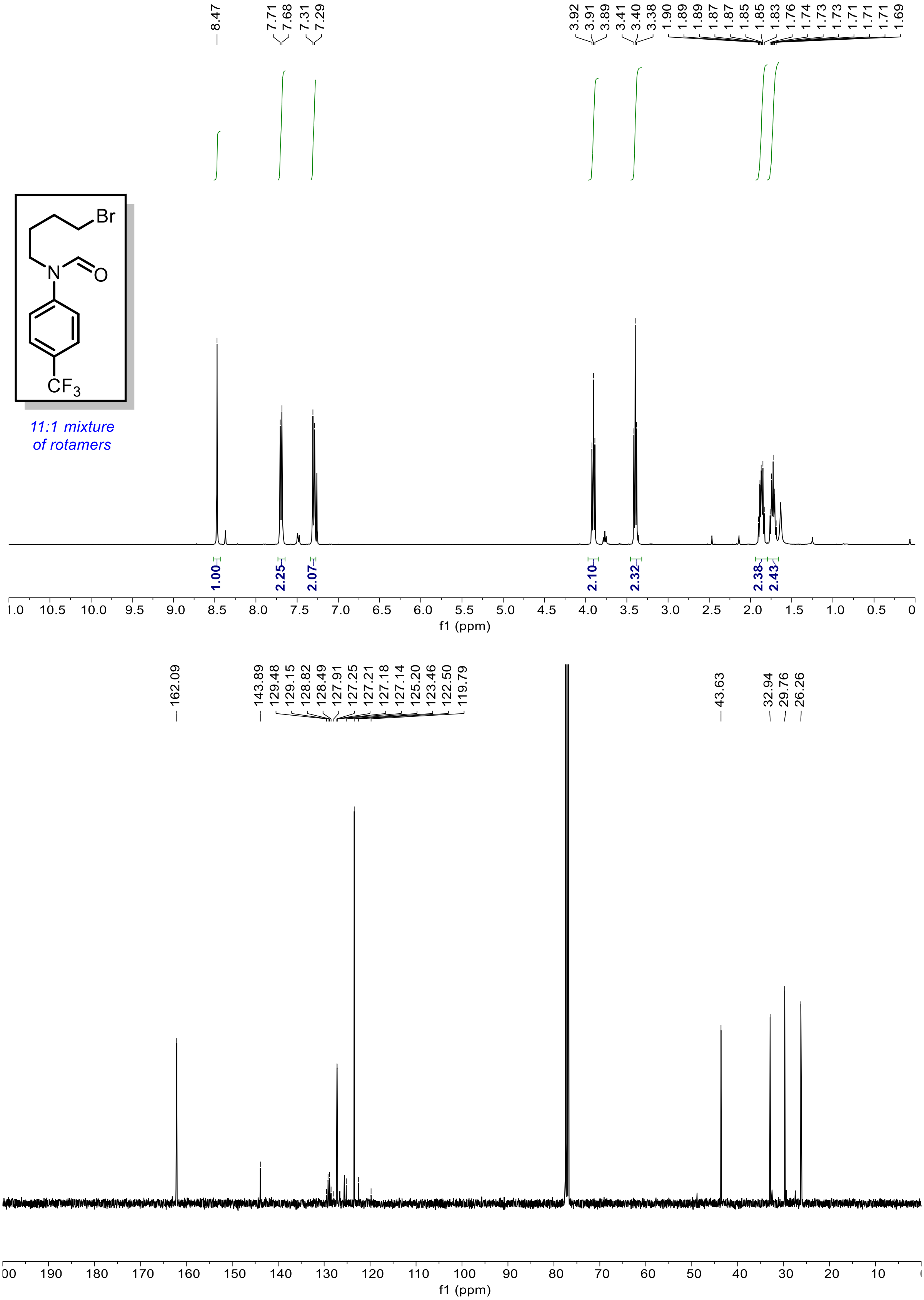

Supplementary Figure 27. ^{1}H and ^{13}C NMR spectra of **2e**.

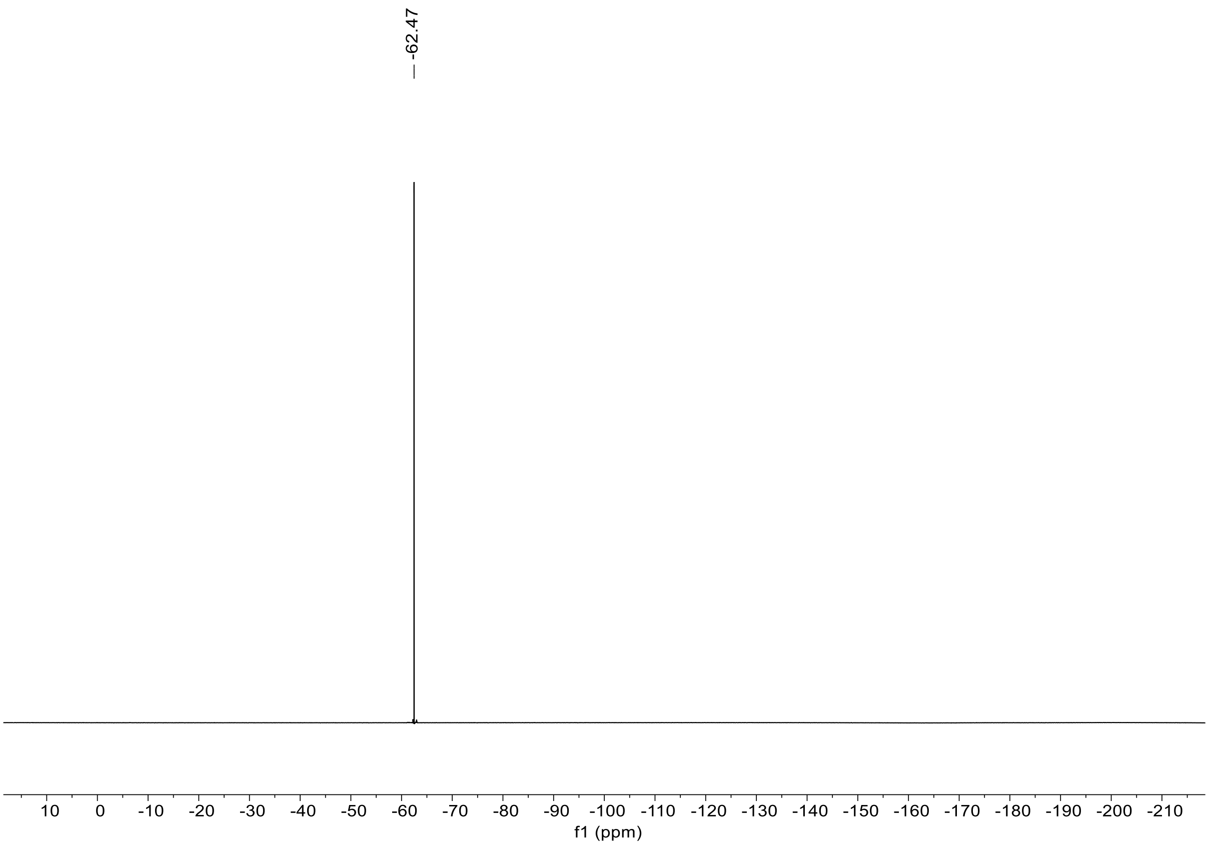

Supplementary Figure 28. ^{19}F NMR spectra of **2e**.

***N*-(4-Acetylphenyl)-*N*-(4-bromobutyl)formamide (2f)**

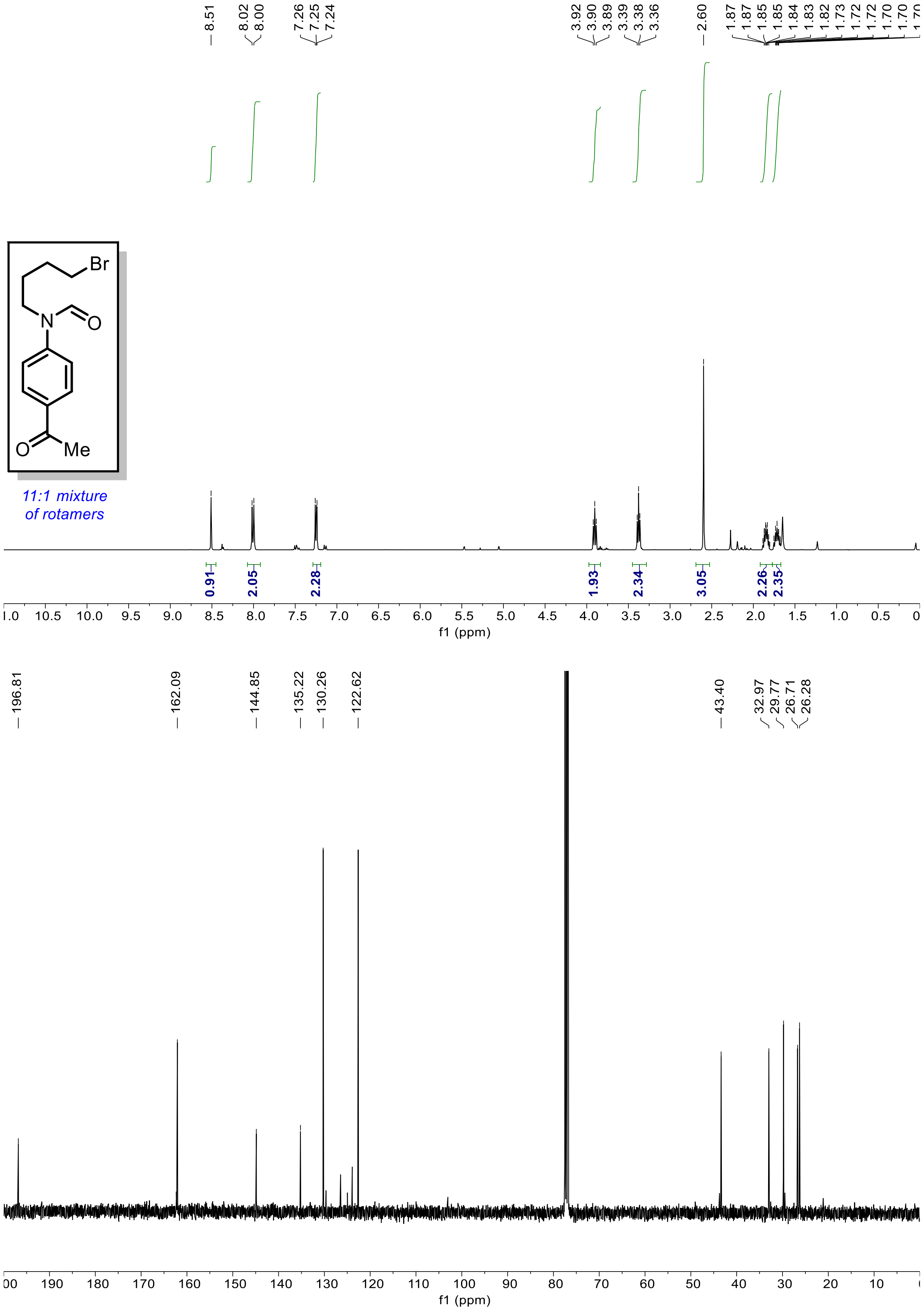

Supplementary Figure 29. ^{1}H and ^{13}C NMR spectra of **2f**.

4-[*N*-(4-Bromobutyl)formamido]-*N*,*N*-dimethylbenzamide (2g)

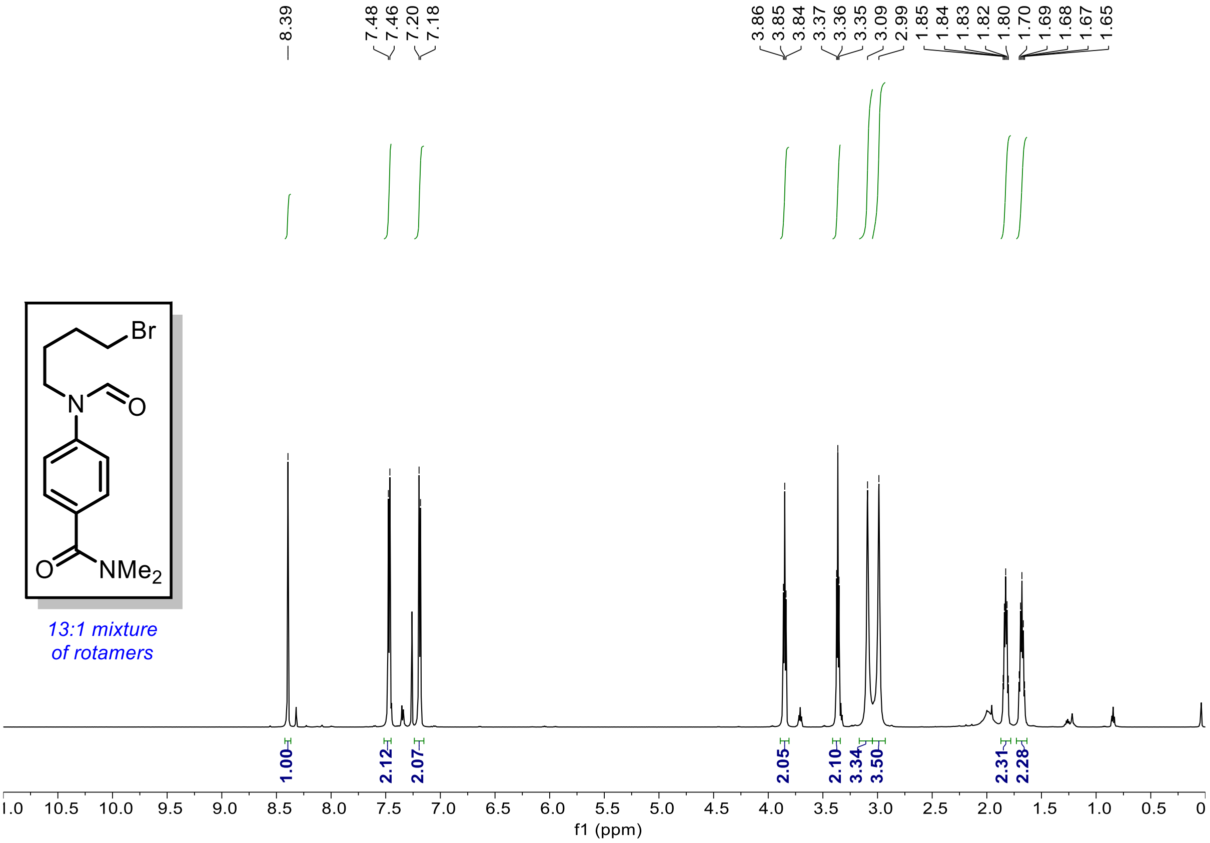

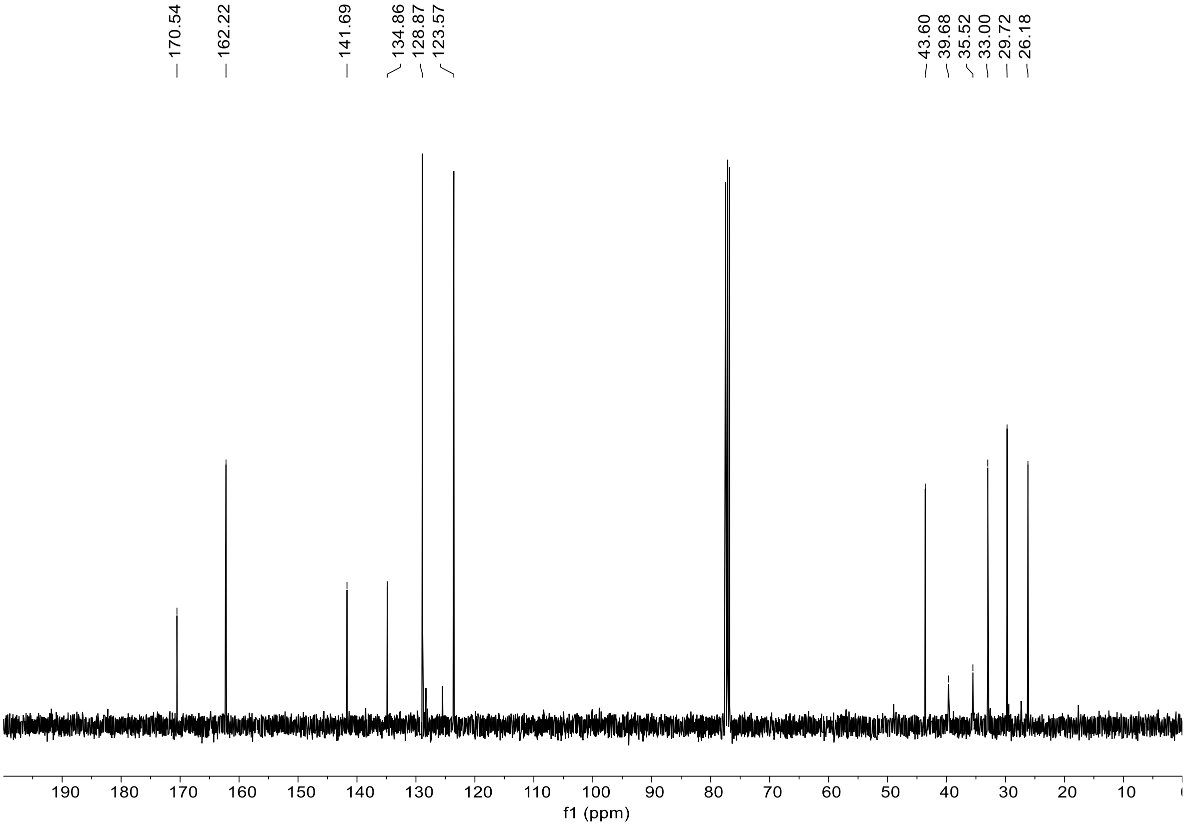

Supplementary Figure 30. ^{1}H and ^{13}C NMR spectra of **2g**.

N-[2-(Bromomethyl)benzyl]-N-phenylformamide (2h)

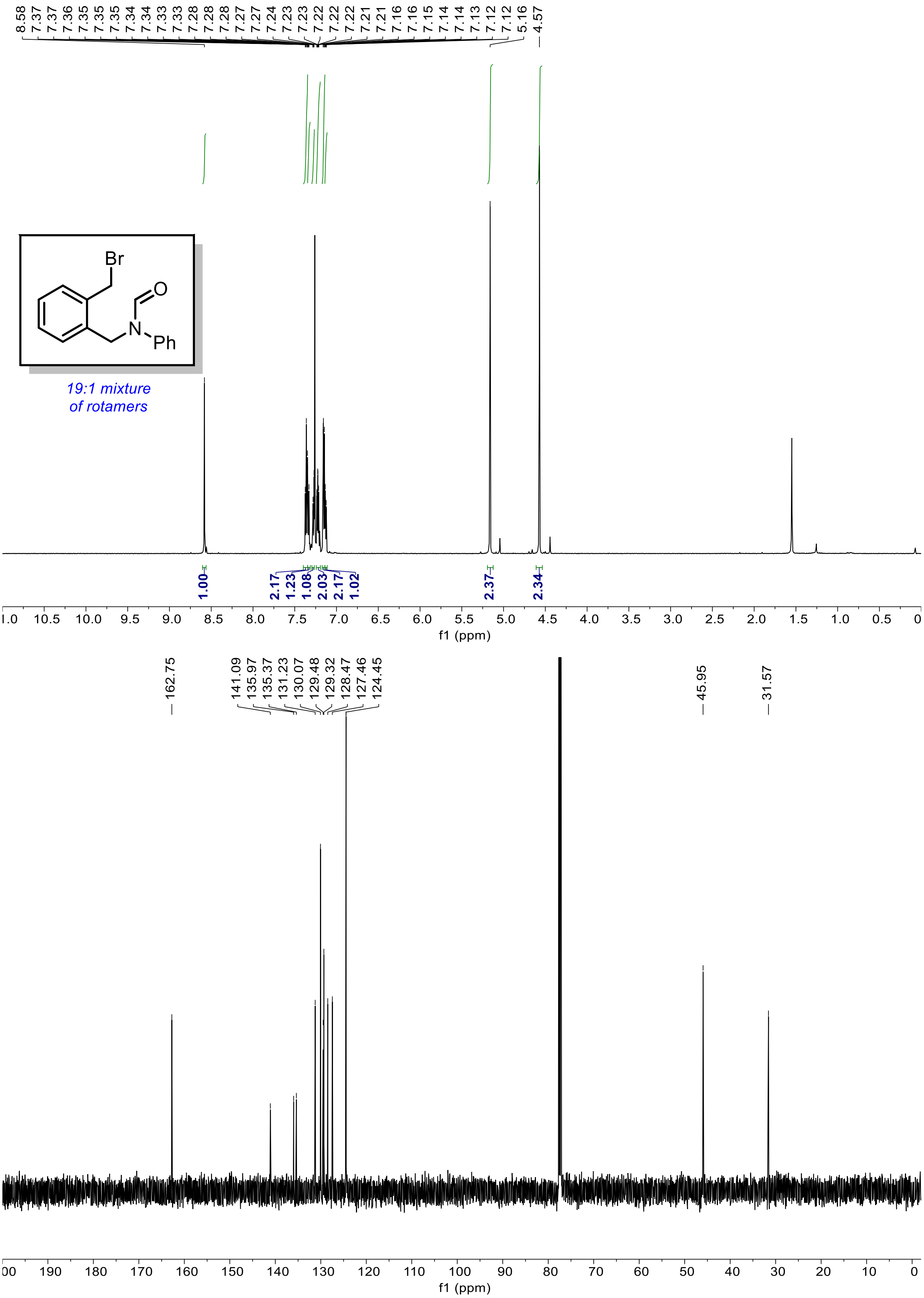

Supplementary Figure 31. ^{1}H and ^{13}C NMR spectra of **2h**.

***N*-[2-(2-Bromoethyl)phenyl]-*N*-phenylformamide (2i)**

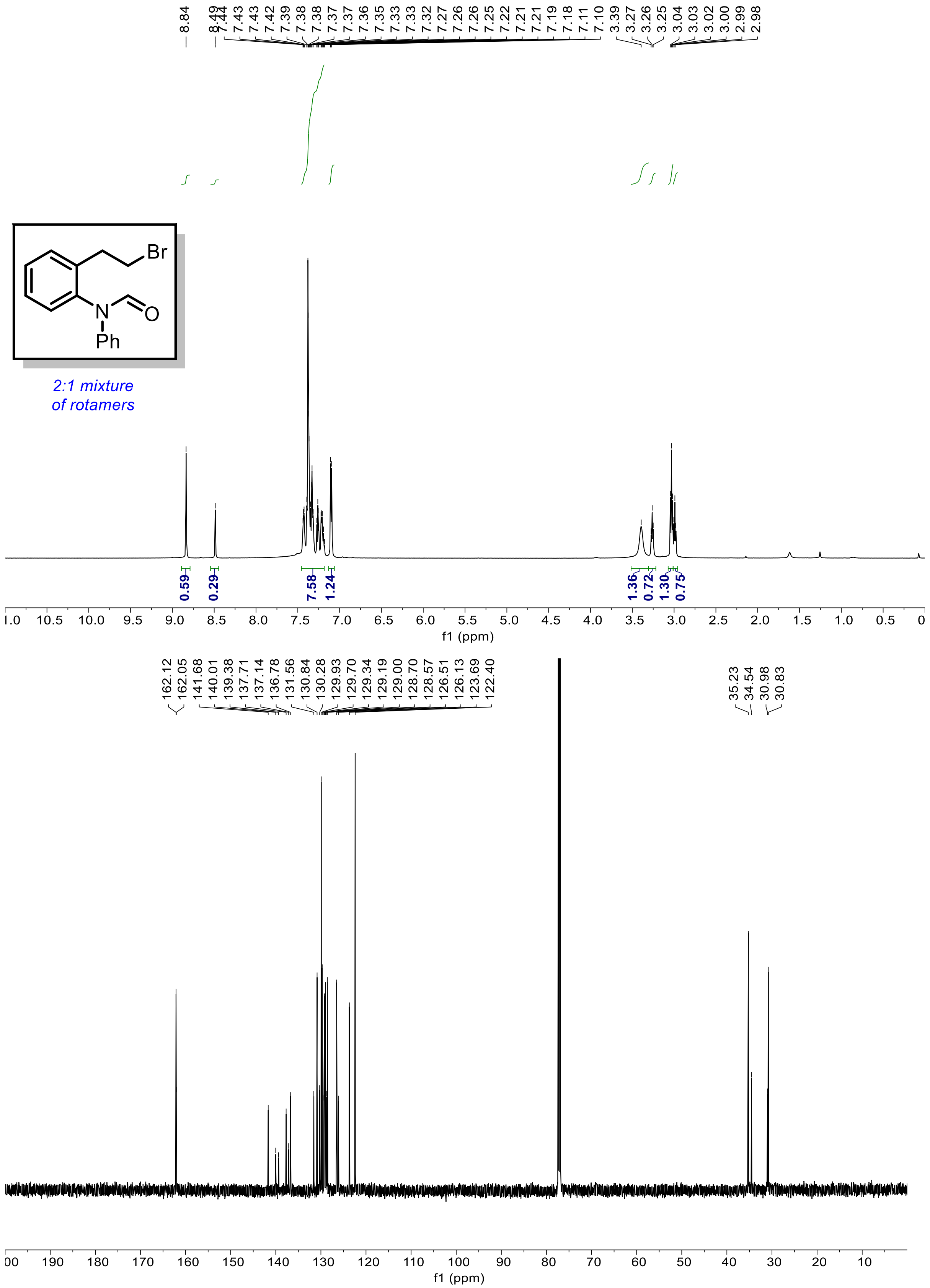

Supplementary Figure 32. ^{1}H and ^{13}C NMR spectra of **2i**.

2-(3-Bromopropyl)pyrrolidine-1-carbaldehyde (2j)

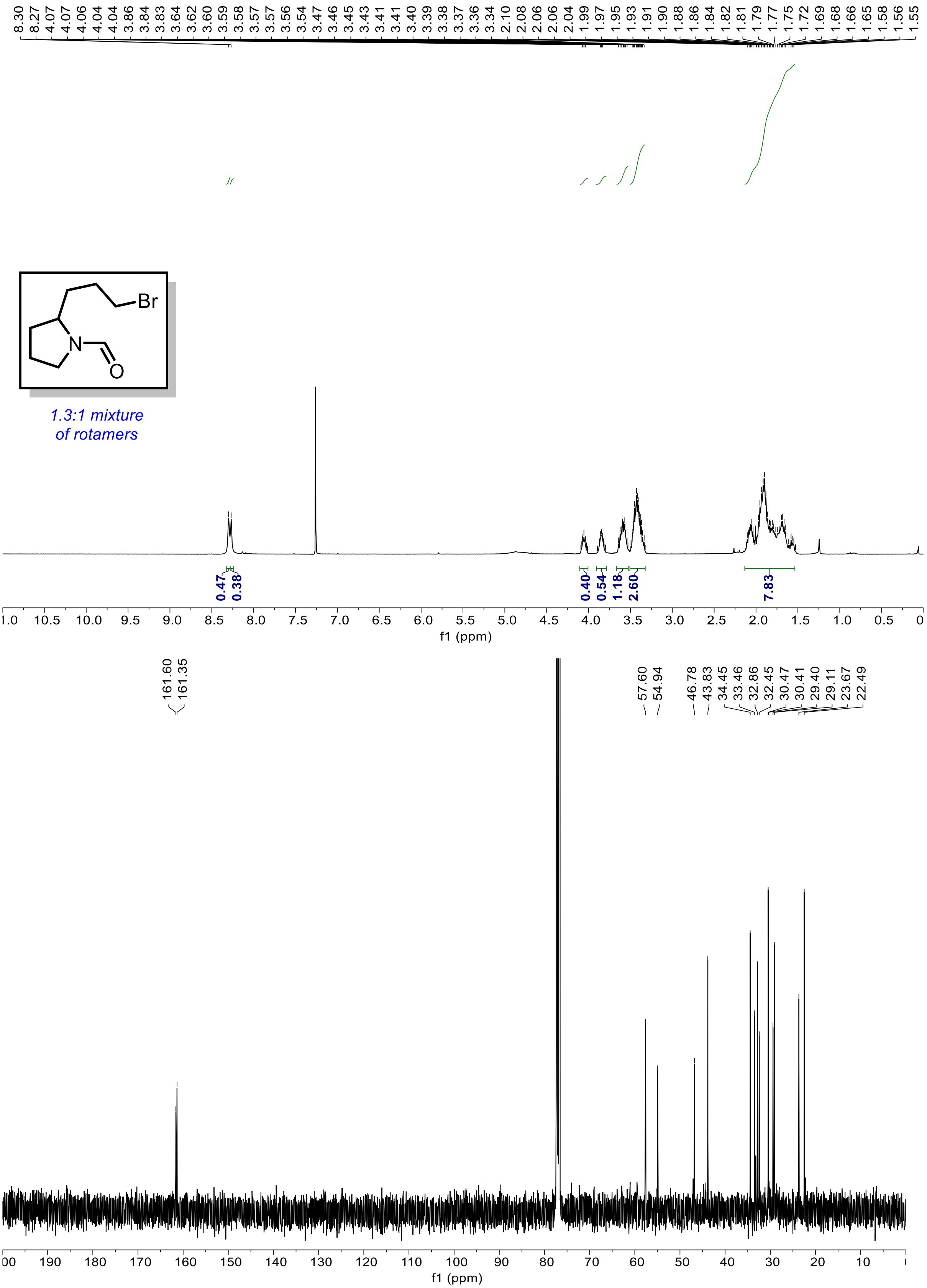

Supplementary Figure 33. ^{1}H and ^{13}C NMR spectra of **2j**.

N-[2-(2-Bromoethoxy)ethyl]-*N*-isopentylformamide (2k)

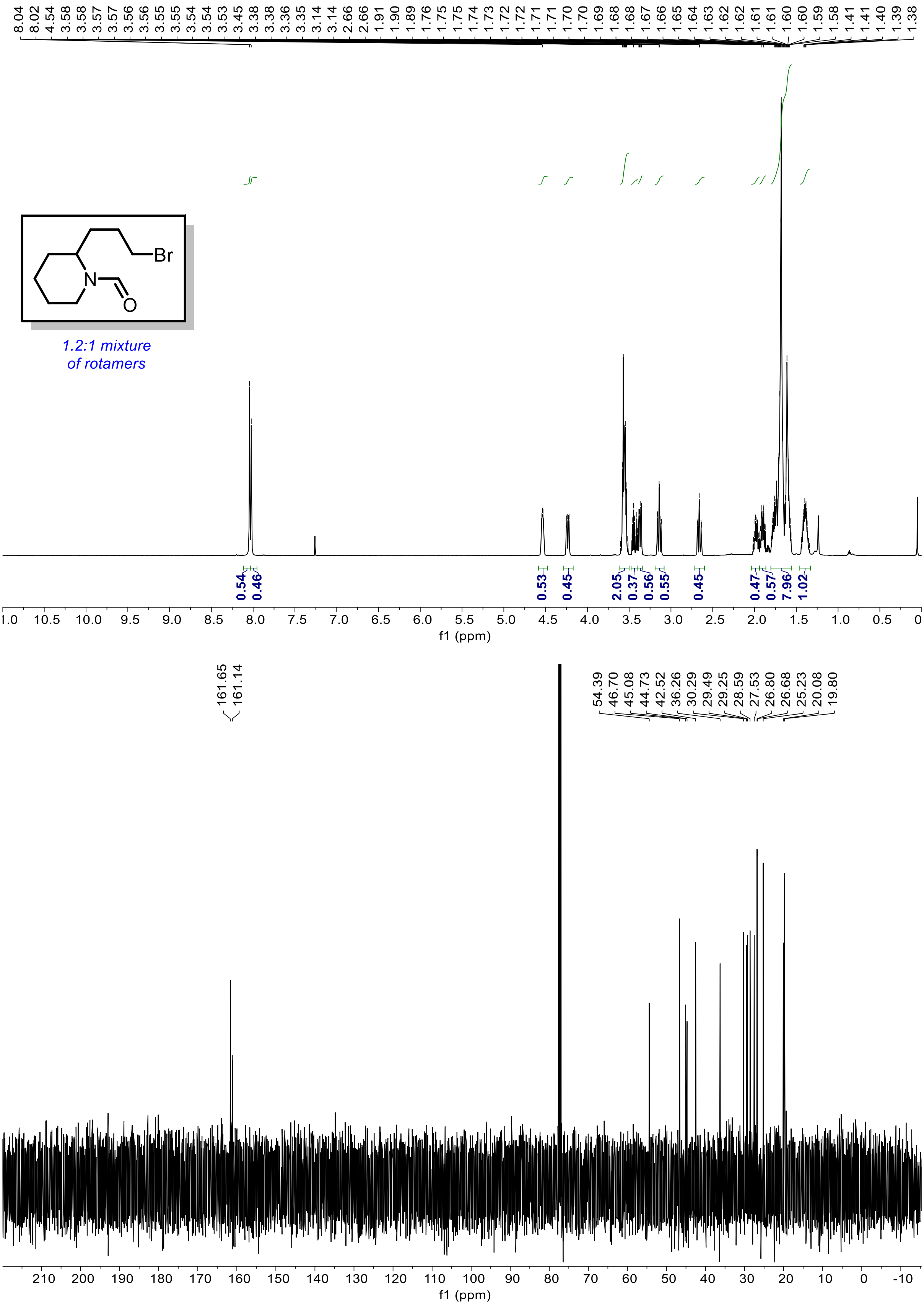

Supplementary Figure 34. ^{1}H and ^{13}C NMR spectra of **2k**.

HMBC (600MHz)

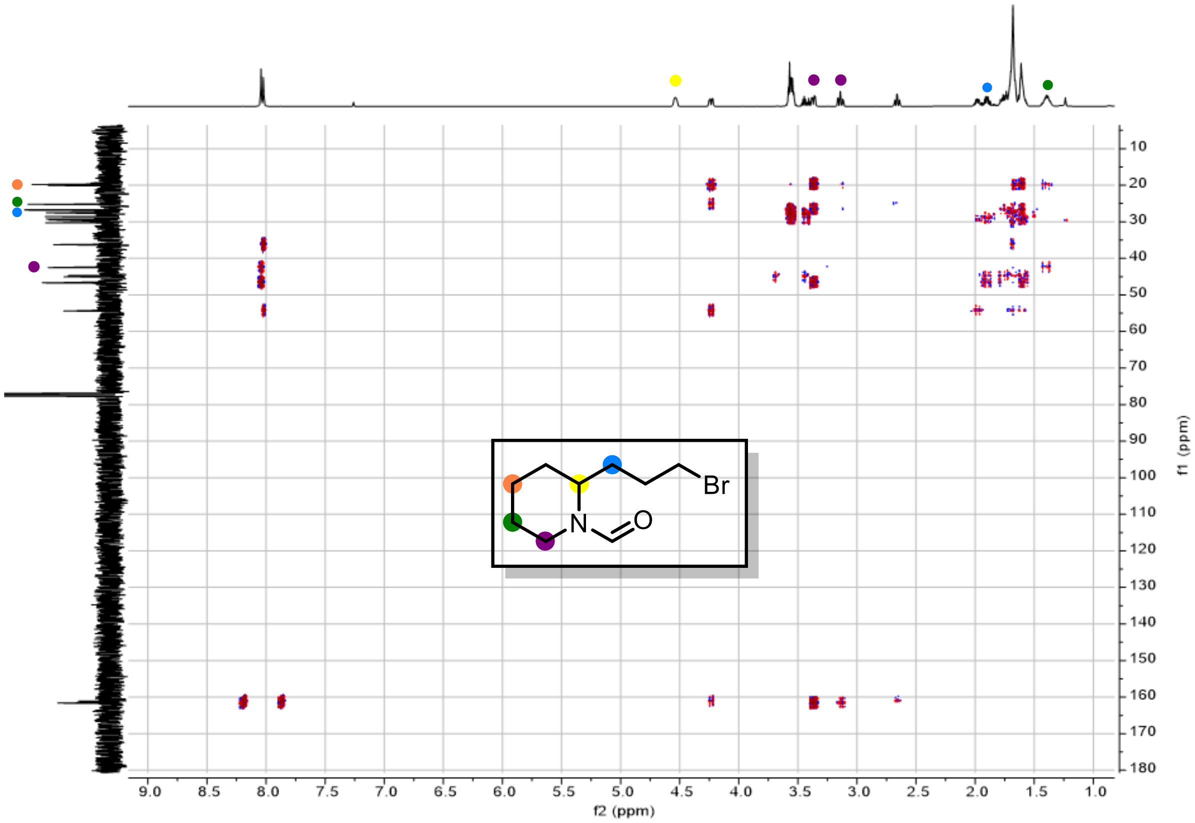

Supplementary Figure 35. HMBC of **2k**.

N-{[(1*S*,2*R*)-2-(Bromomethyl)cyclohexyl]methyl}-*N*-(4-methoxyphenyl)formamide (2l)

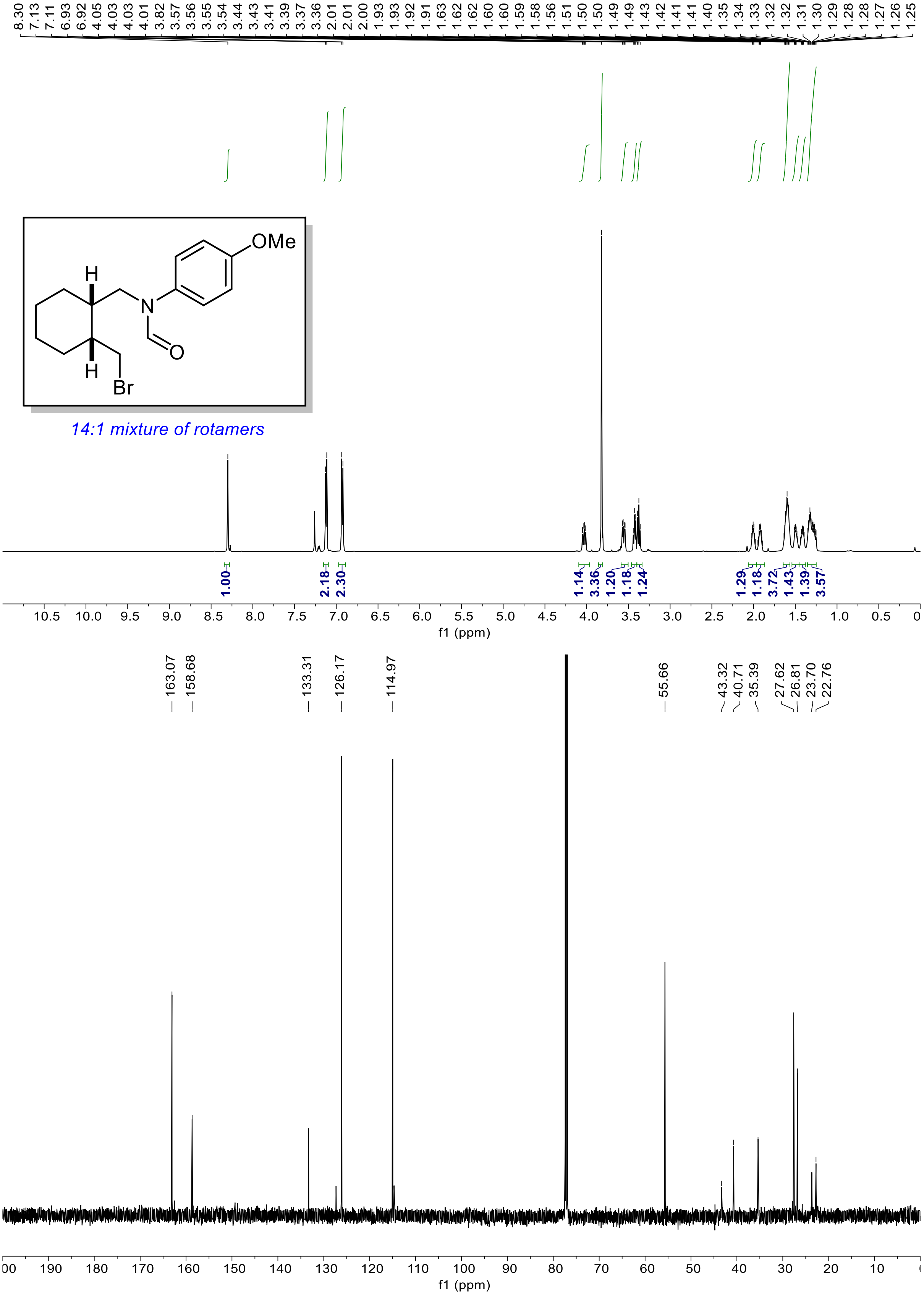

Supplementary Figure 36. ^{1}H and ^{13}C NMR spectra of **2l**.

(*Z*)-*N*-(4-Bromobut-2-en-1-yl)-*N*-(4-methoxyphenyl)formamide (2m)

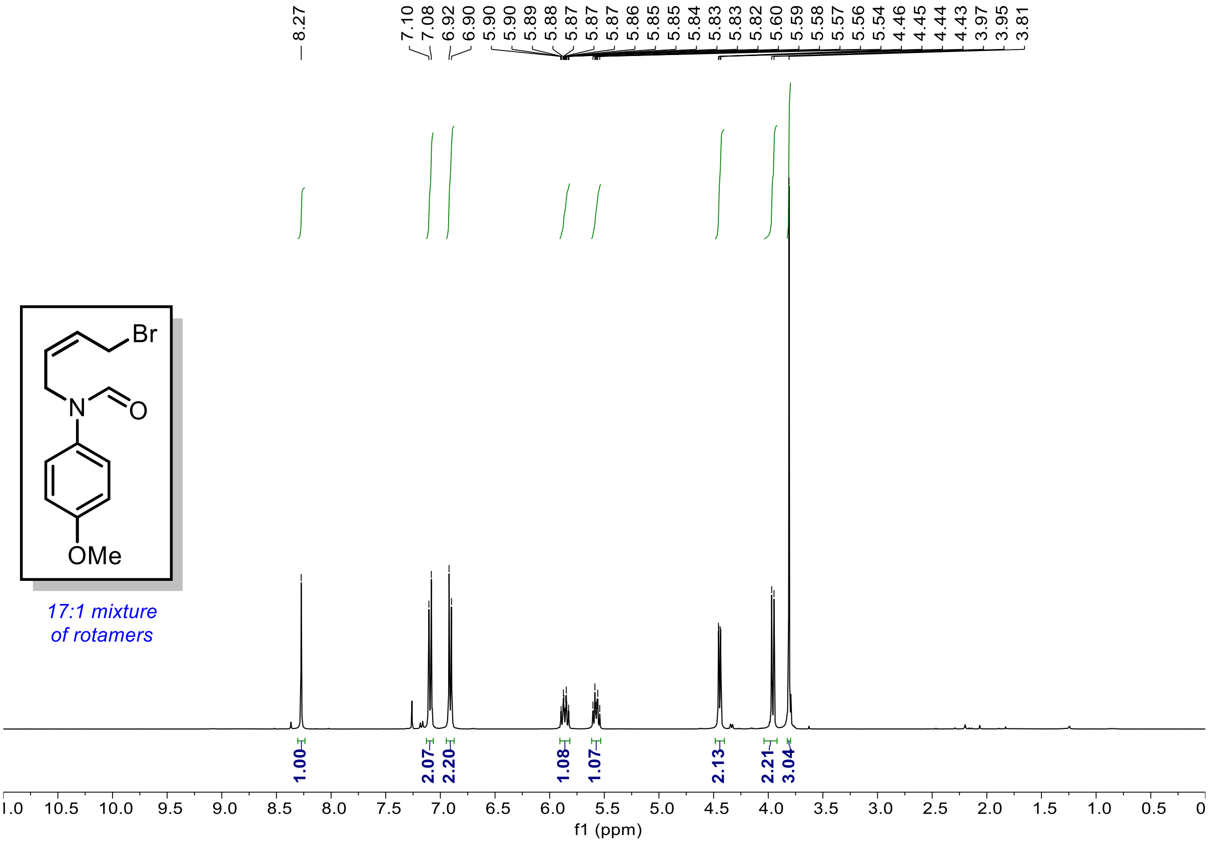

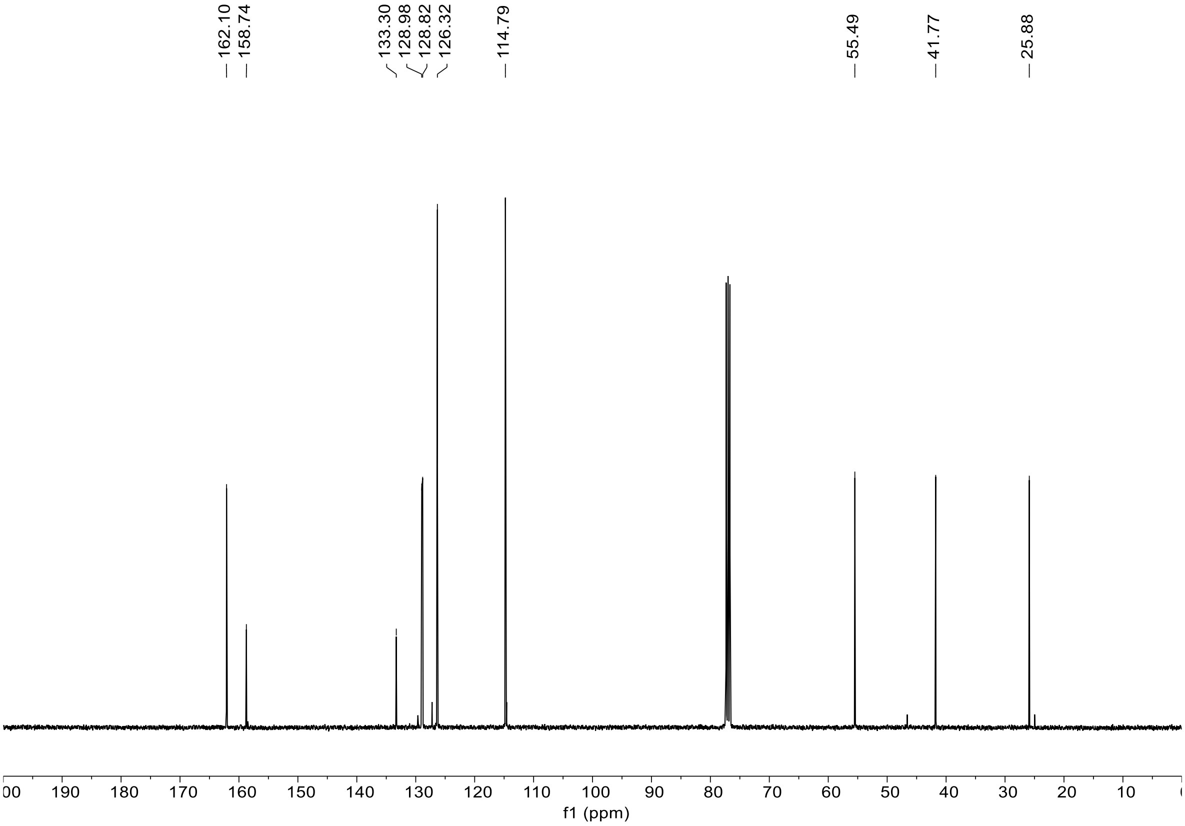

Supplementary Figure 37. ^{1}H and ^{13}C NMR spectra of **2m**.

***tert*-Butyl 4-[*N*-(4-bromobutyl)formamido]piperidine-1-carboxylate (2n)**

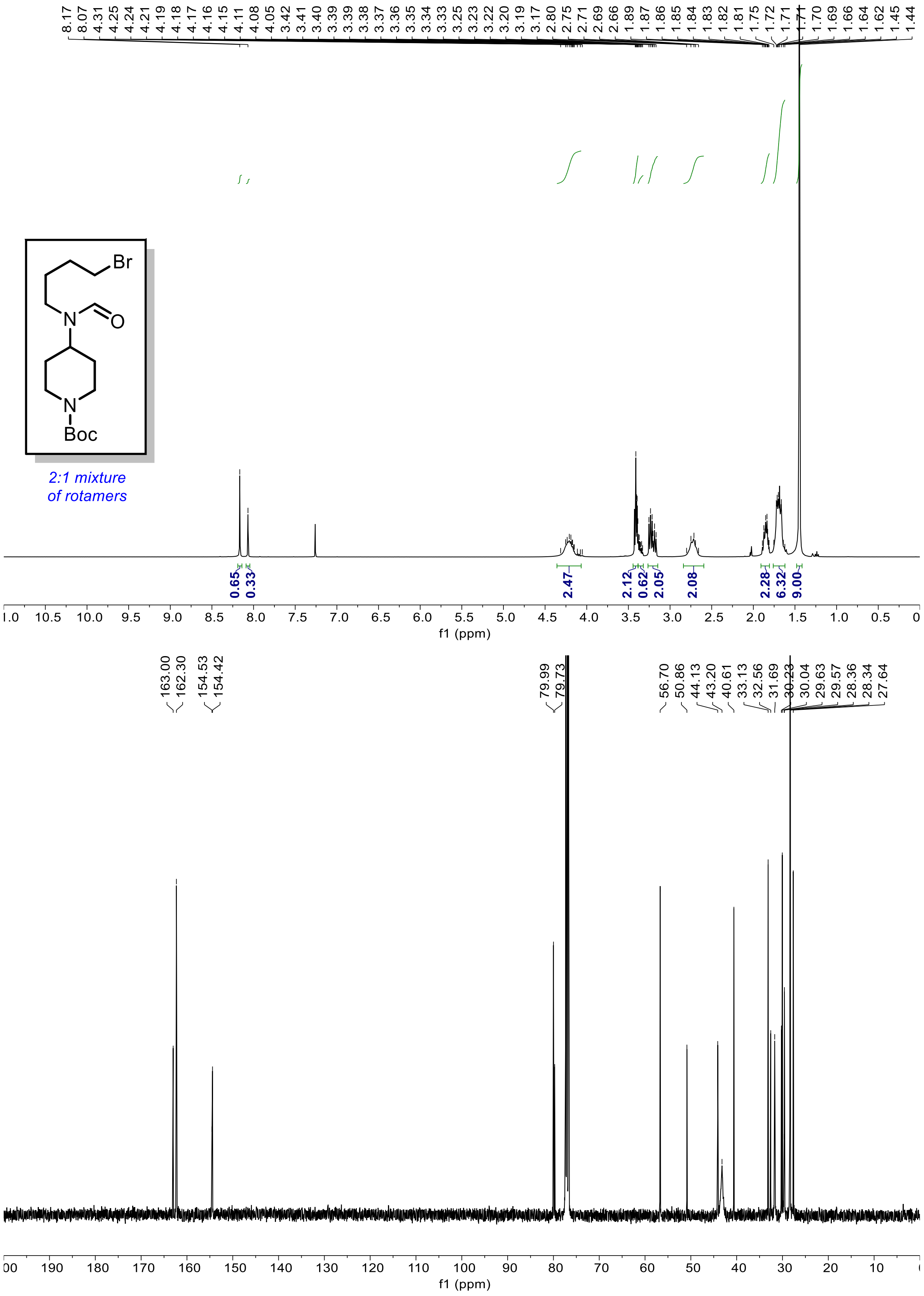

Supplementary Figure 38. ^{1}H and ^{13}C NMR spectra of **2n**.

2-(3-Bromopropyl)-4-tosyl-3,4-dihydroquinoxaline-1(2*H*)-carbaldehyde (2o)

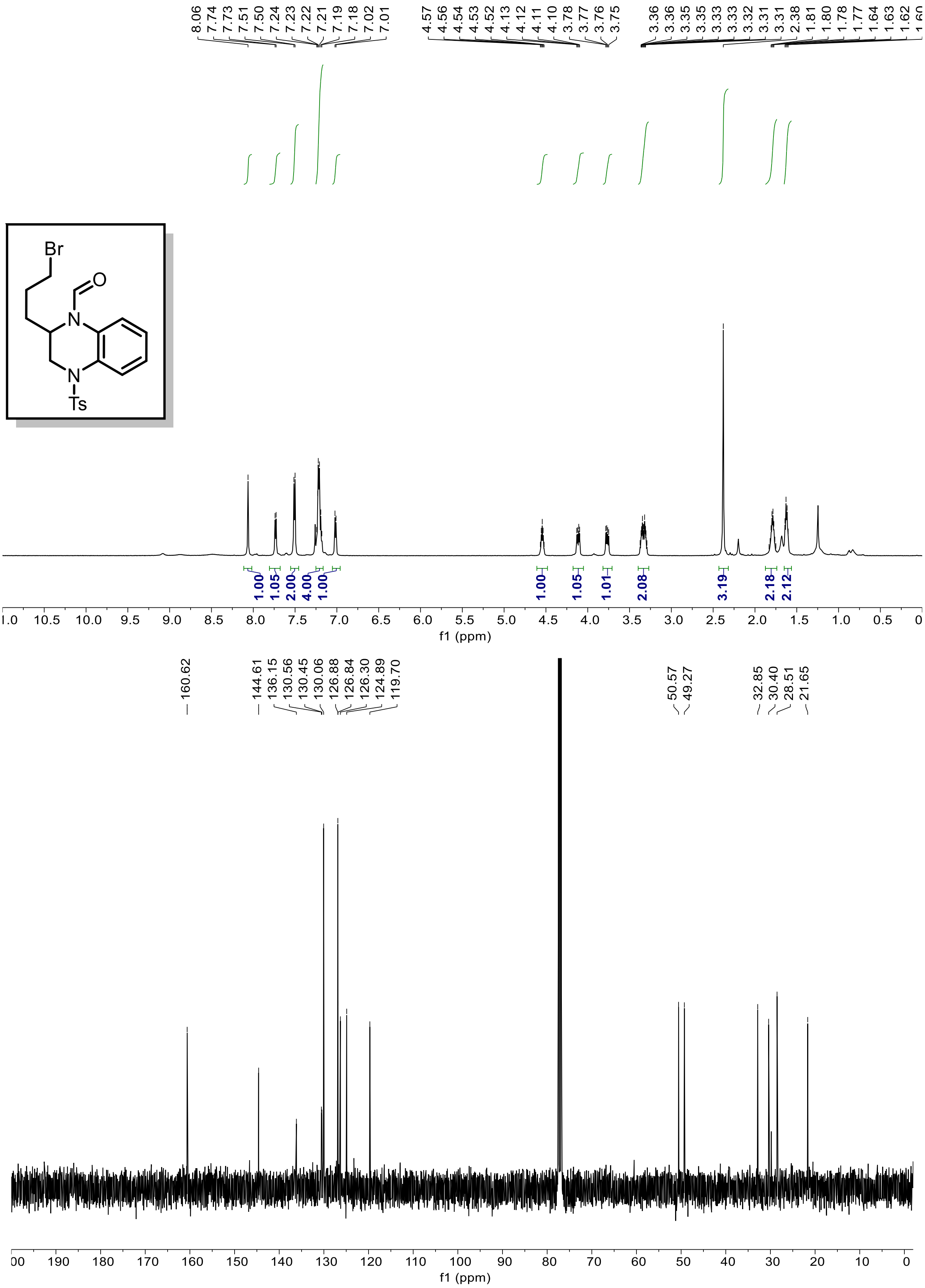

Supplementary Figure 39. ^{1}H and ^{13}C NMR spectra of **2o**.

N-(4-Bromo-2-phenylbutyl)-*N*-(4-methoxyphenyl)formamide (2p)

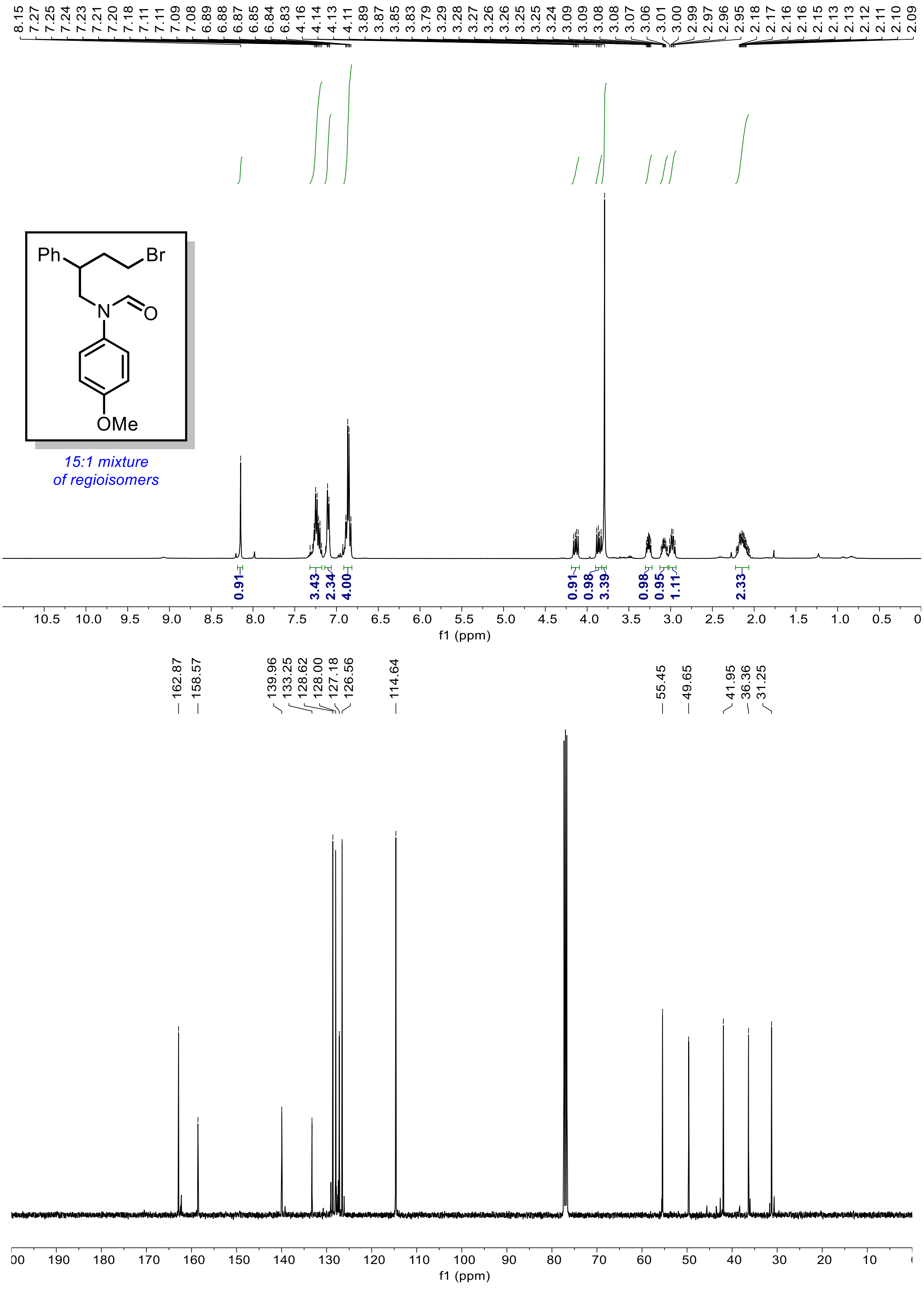

Supplementary Figure 40. ^{1}H and ^{13}C NMR spectra of **2p**.

NOESY (400MHz)

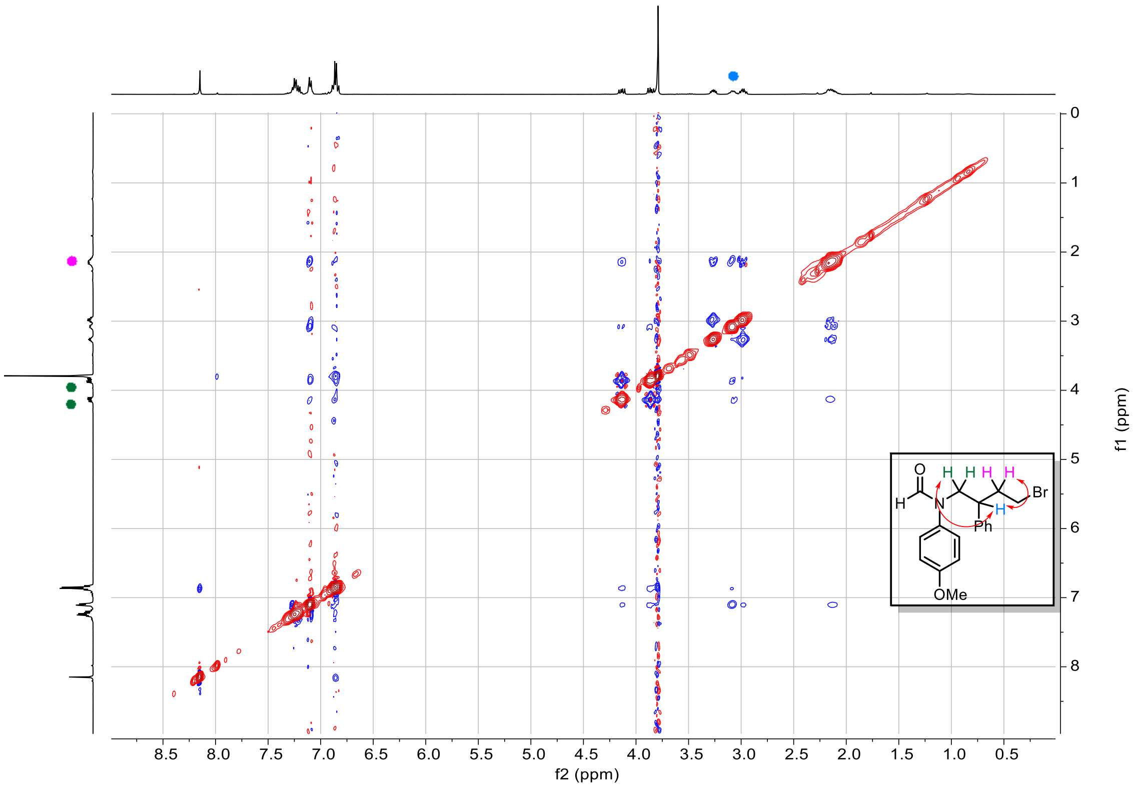

HMBC (400MHz)

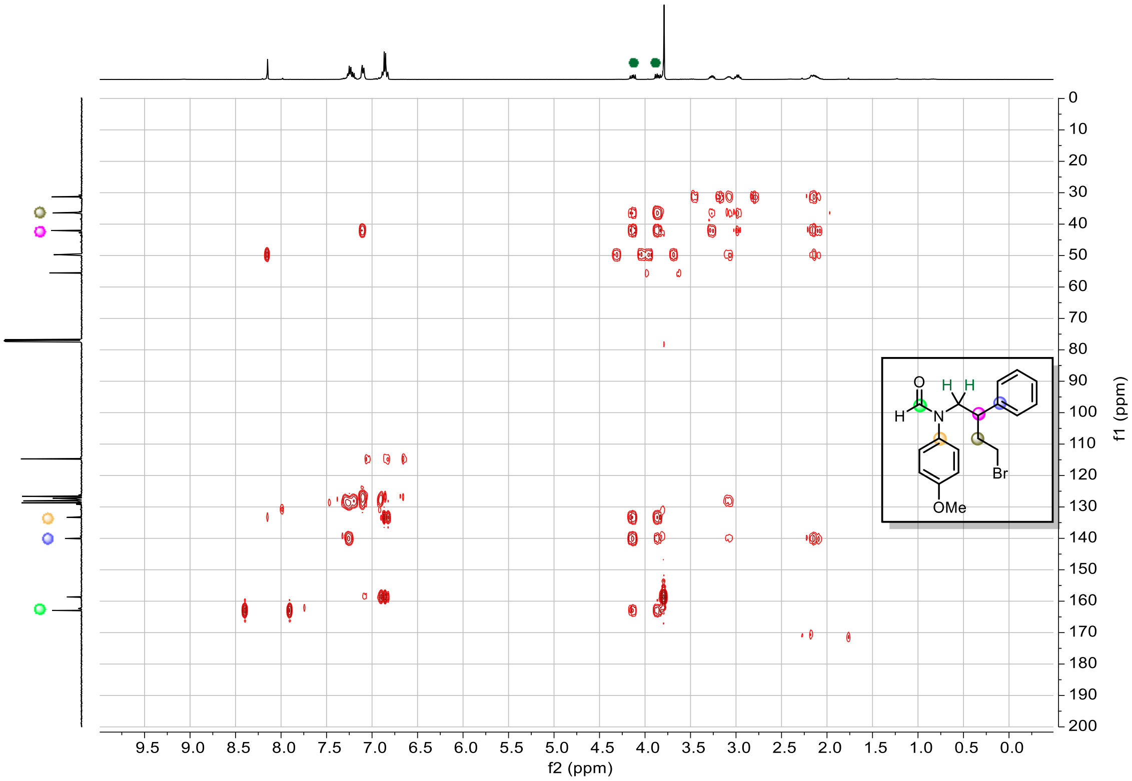

Supplementary Figure 41. NOESY and HMBC of **2p**.

N-{[1-(2-Bromoethyl)cyclohexyl]methyl}-*N*-phenylformamide (2q)

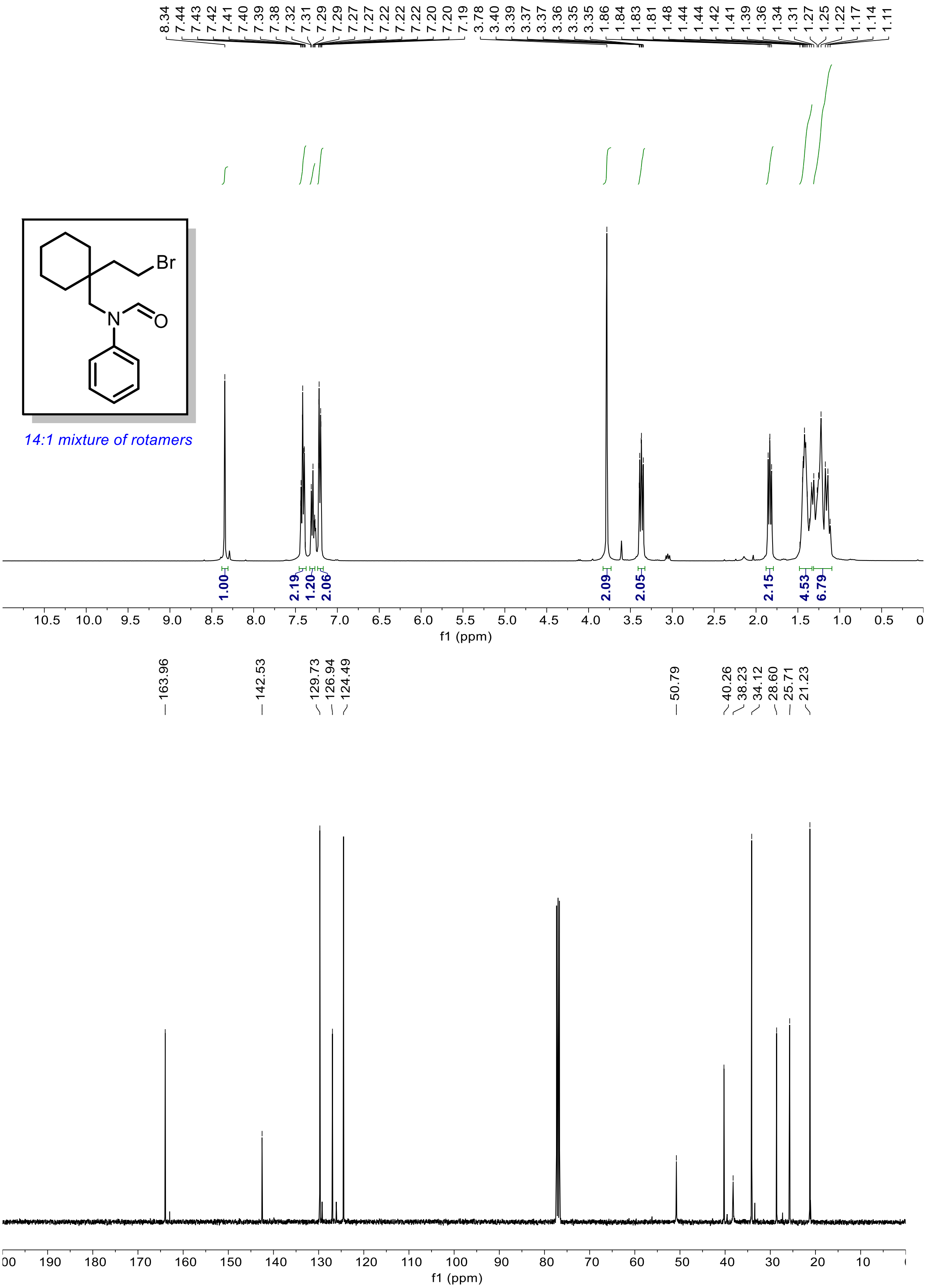

Supplementary Figure 42. ^{1}H and ^{13}C NMR spectra of **2q**.

HSQC (400MHz)

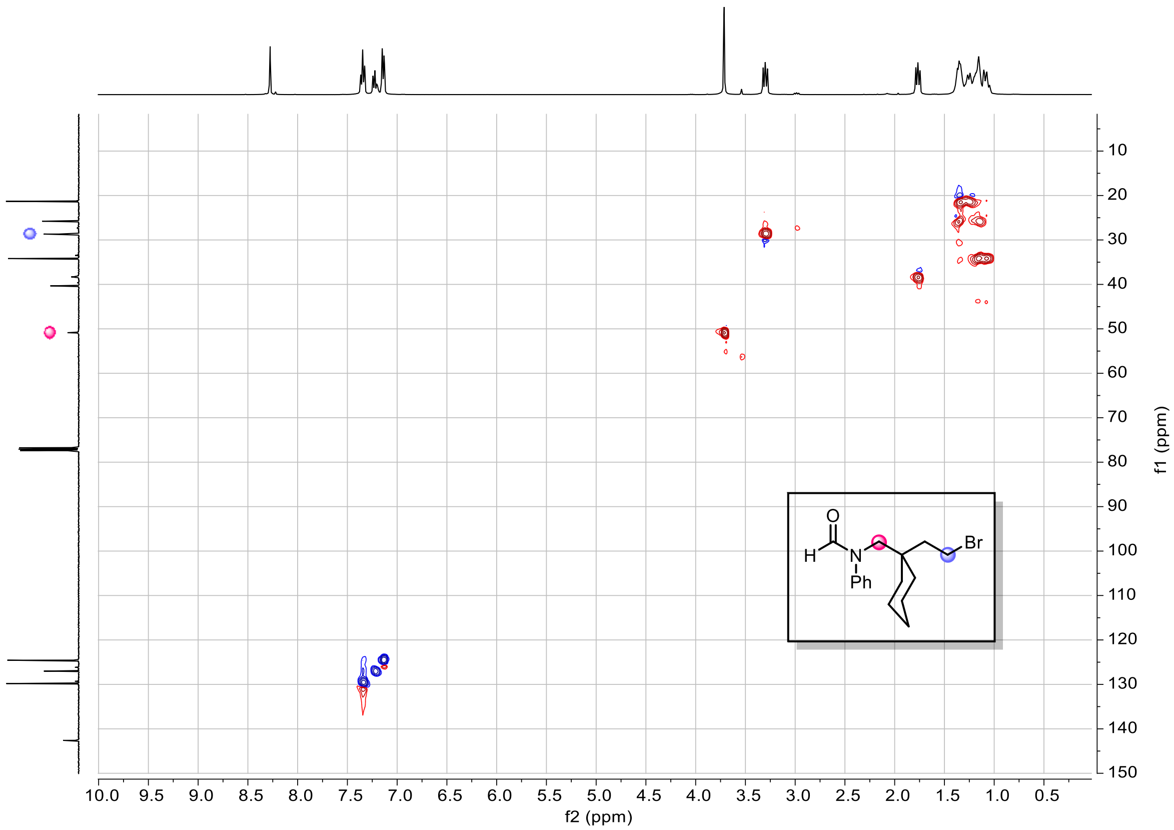

Supplementary Figure 43. HSQC of **2q**.

(*S*)-5-Bromo-2-(*N*-methylformamido)pentyl acetate (2r)

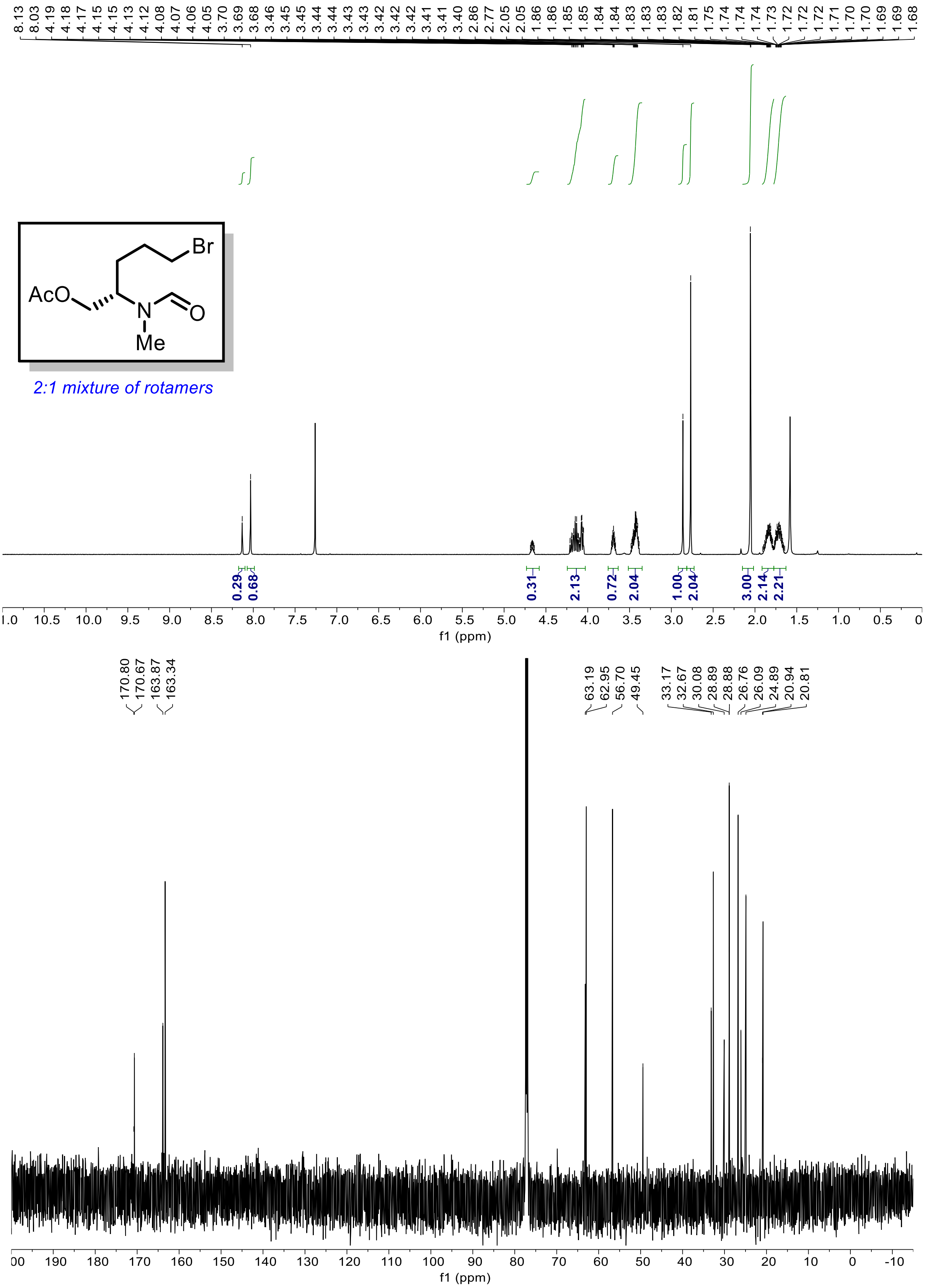

Supplementary Figure 44. ^{1}H and ^{13}C NMR spectra of **2r**.

NOESY (400MHz)

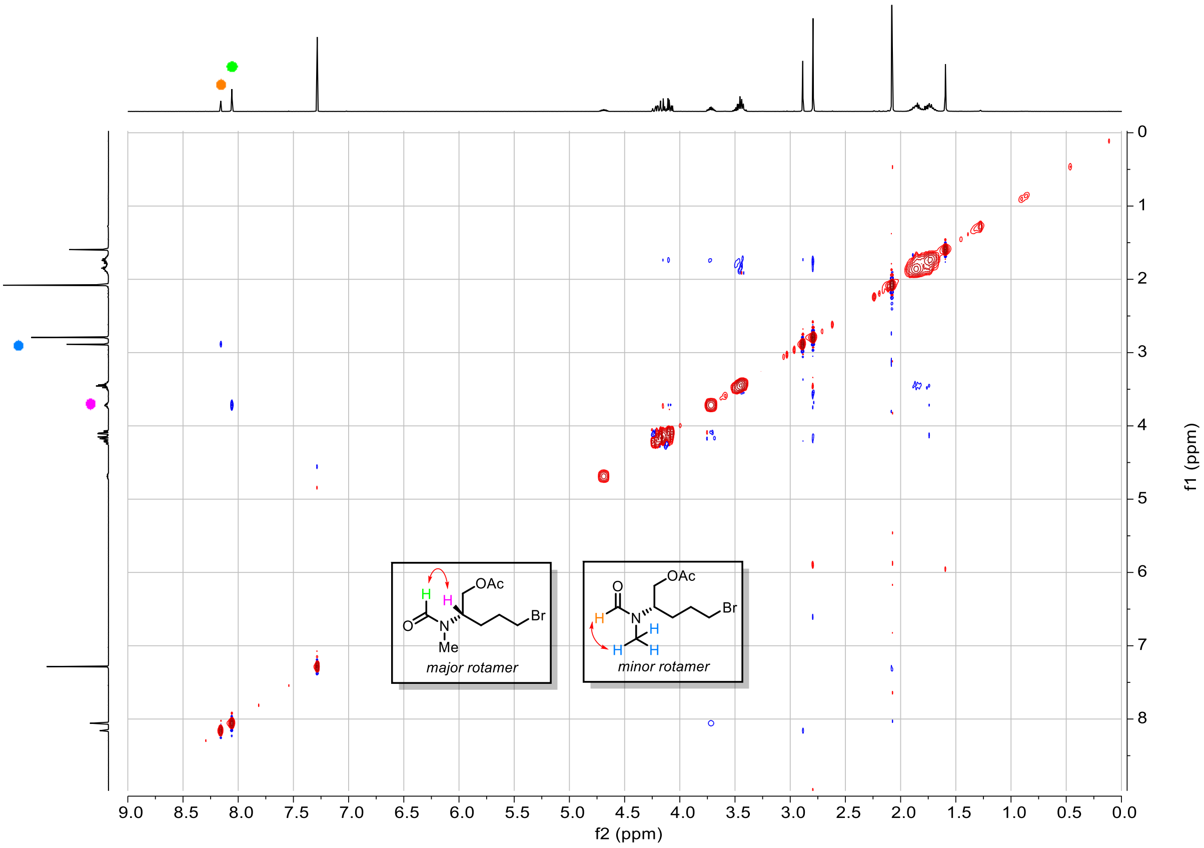

HMBC (400MHz)

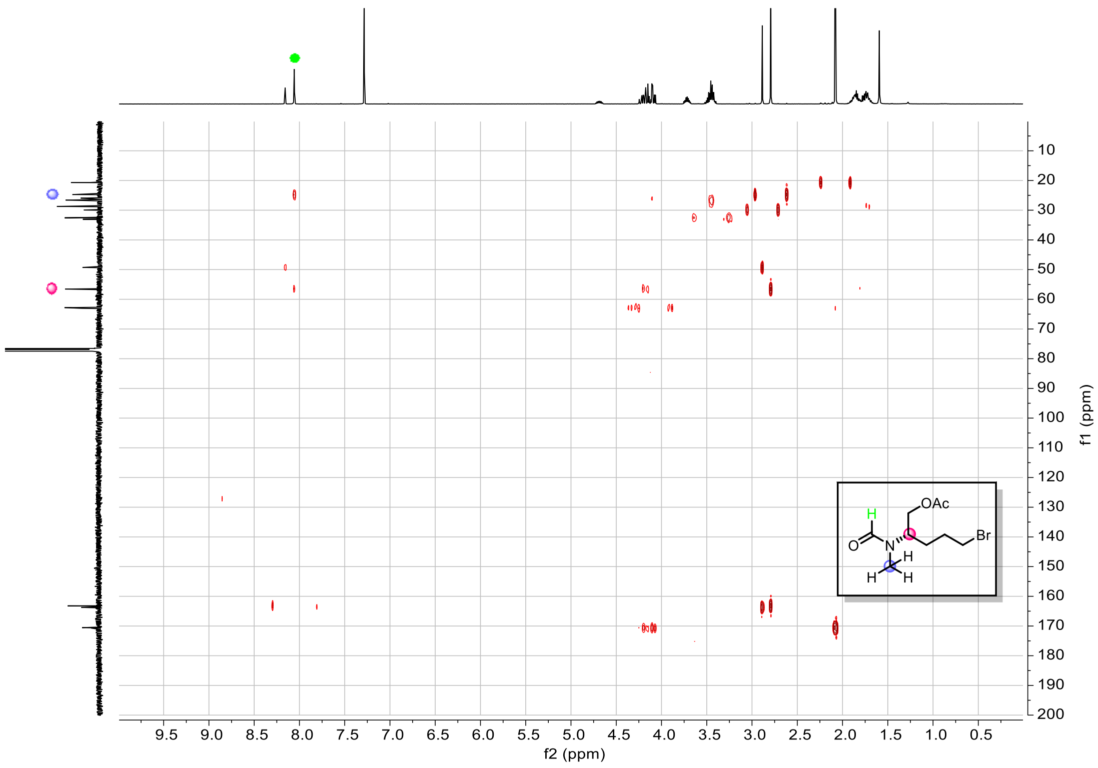

Supplementary Figure 45. NOESY and HMBC of **2r**.

N-(4-Chlorobutyl)-*N*-phenylformamide (2a-Cl)

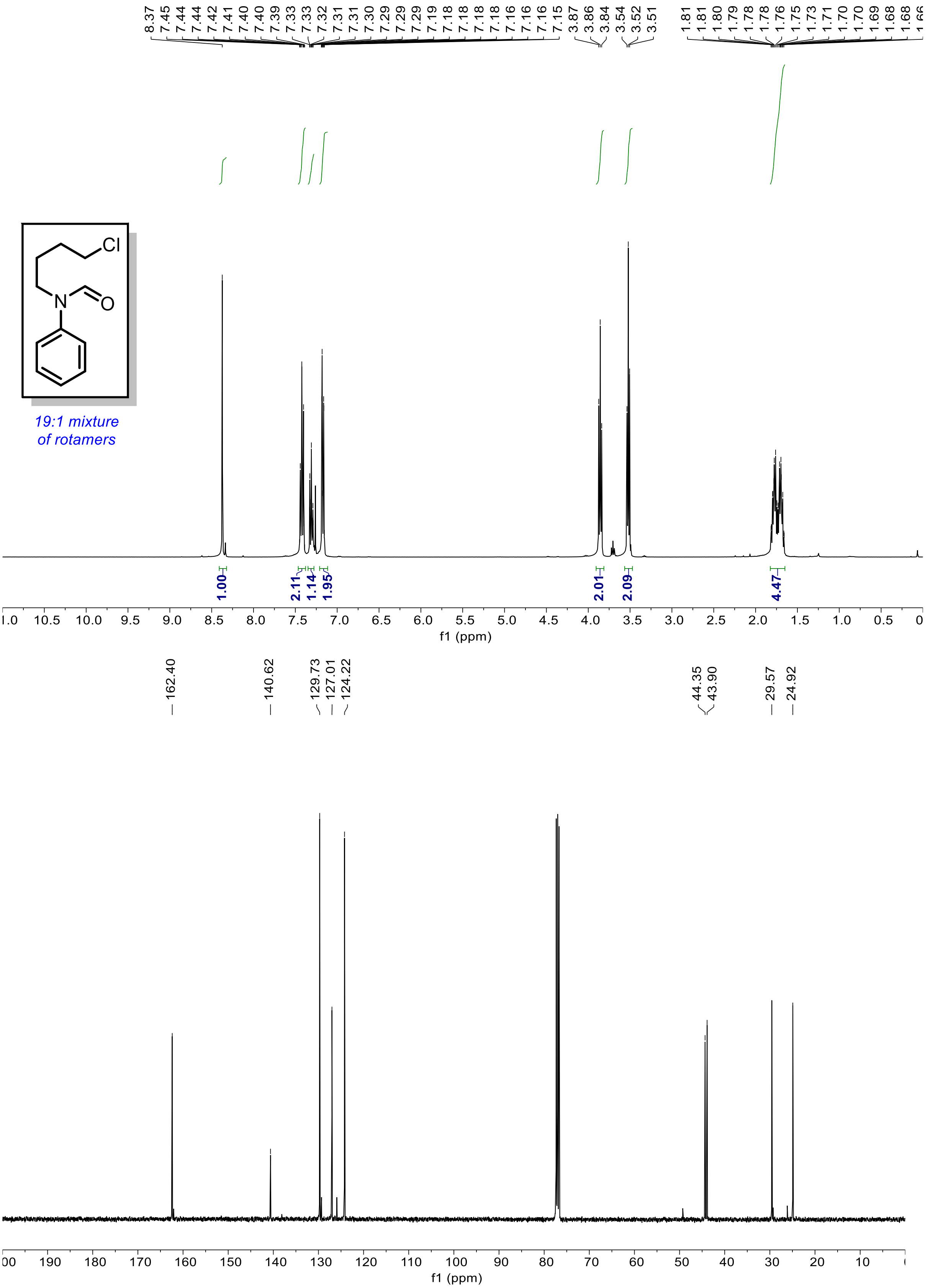

Supplementary Figure 46. ^{1}H and ^{13}C NMR spectra of **2a-Cl**.

N-(4-Chlorobutyl)-*N*-ethylformamide (2b-Cl)

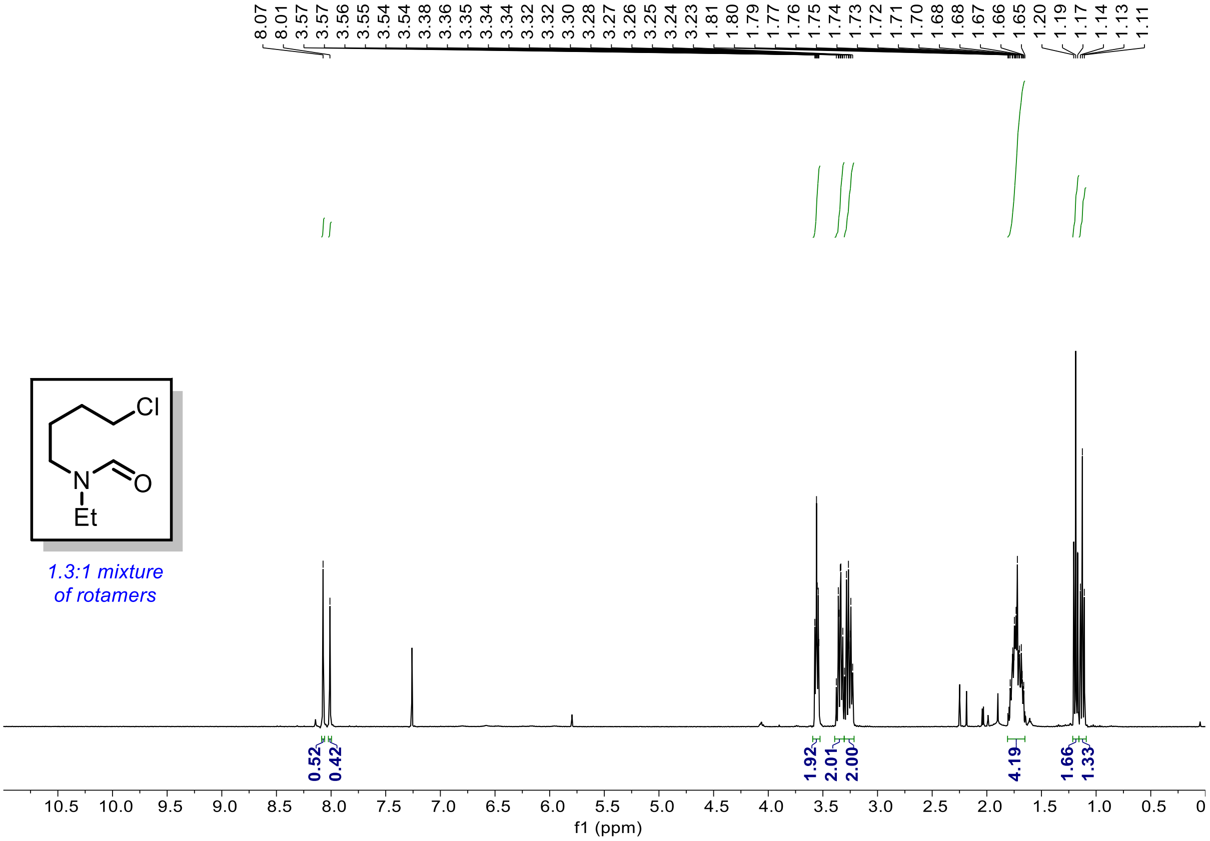

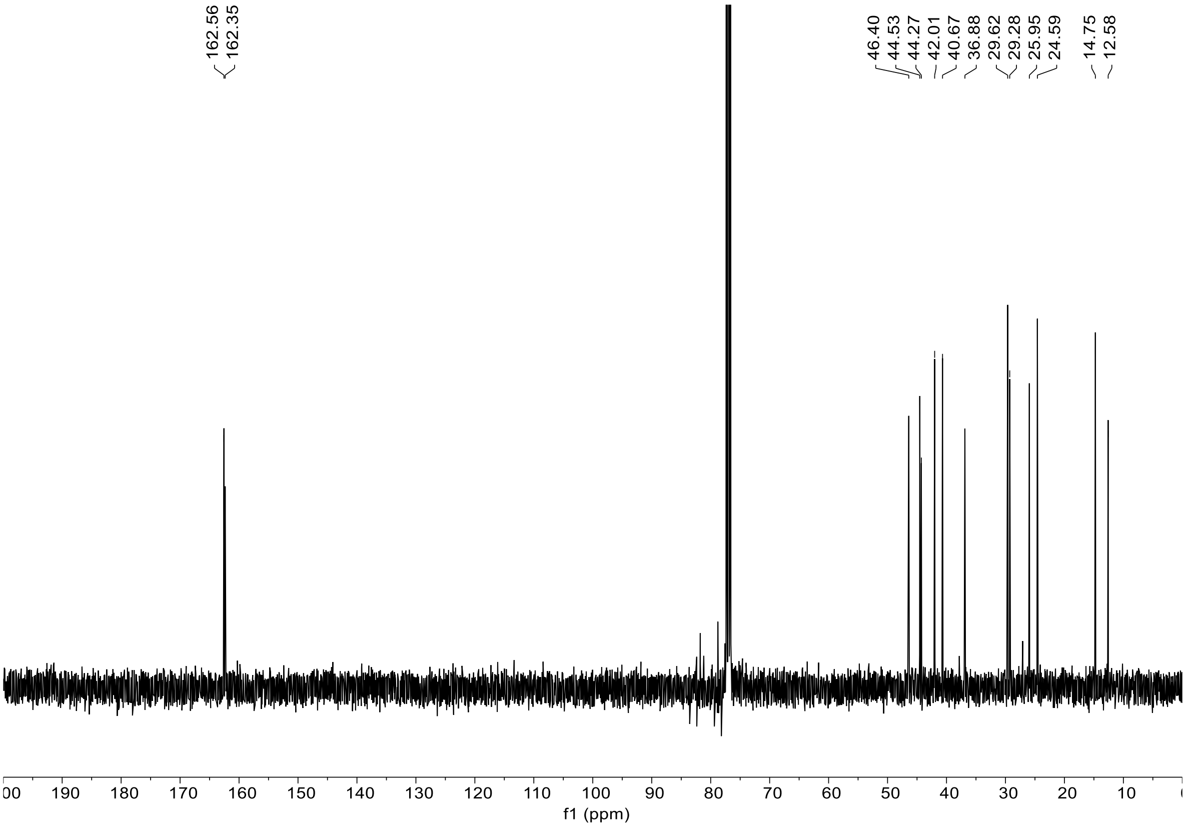

Supplementary Figure 47. ^{1}H and ^{13}C NMR spectra of **2b-Cl**.

***N*-(4-Iodobutyl)-*N*-(4-methoxyphenyl)formamide (2d-I)**

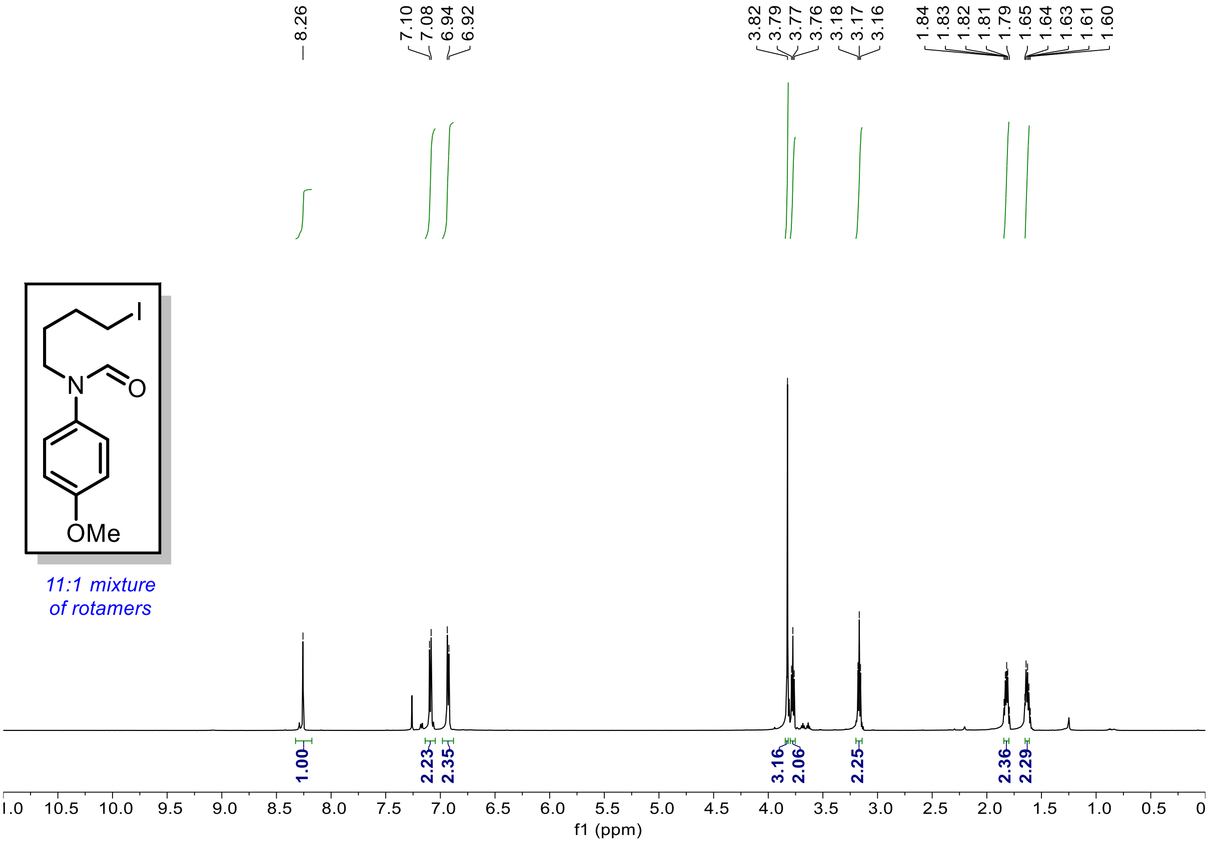

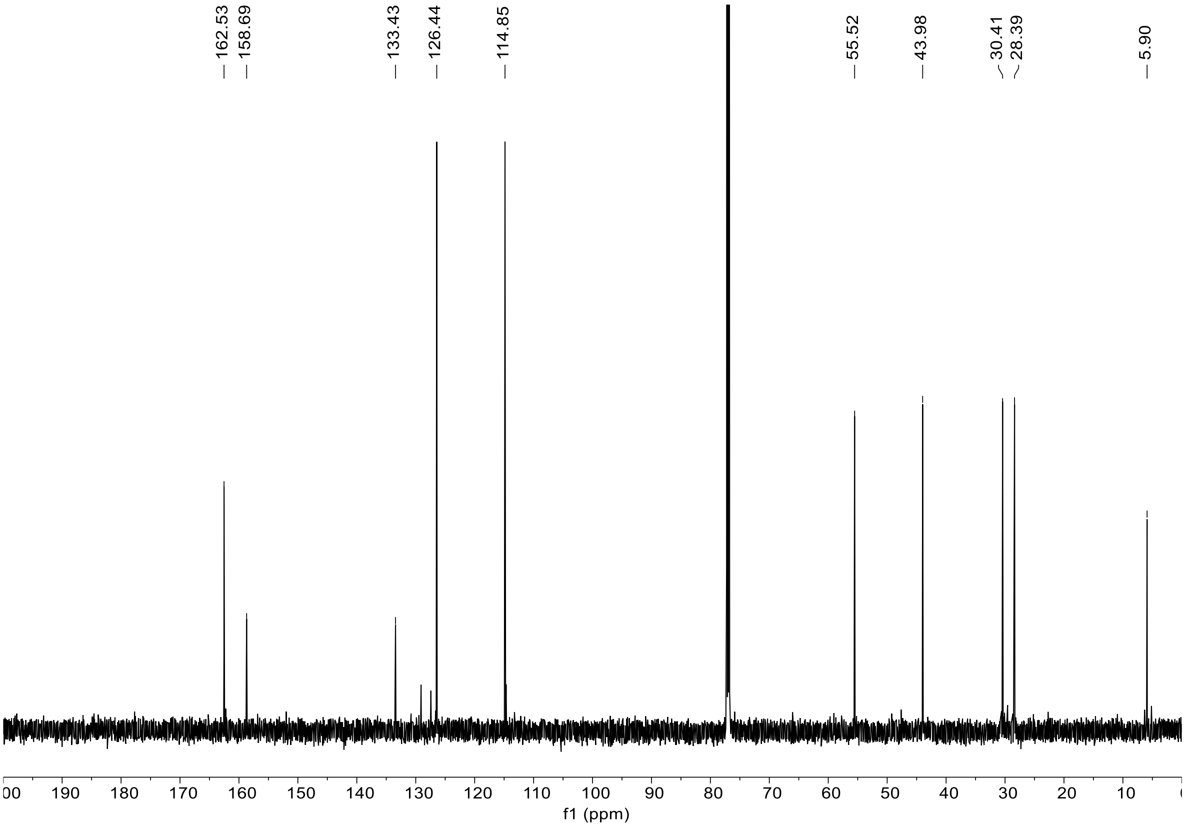

Supplementary Figure 48. ^{1}H and ^{13}C NMR spectra of **2d-I**.

4-(*N*-Phenylformamido)butyl acetate (2a-OAc)

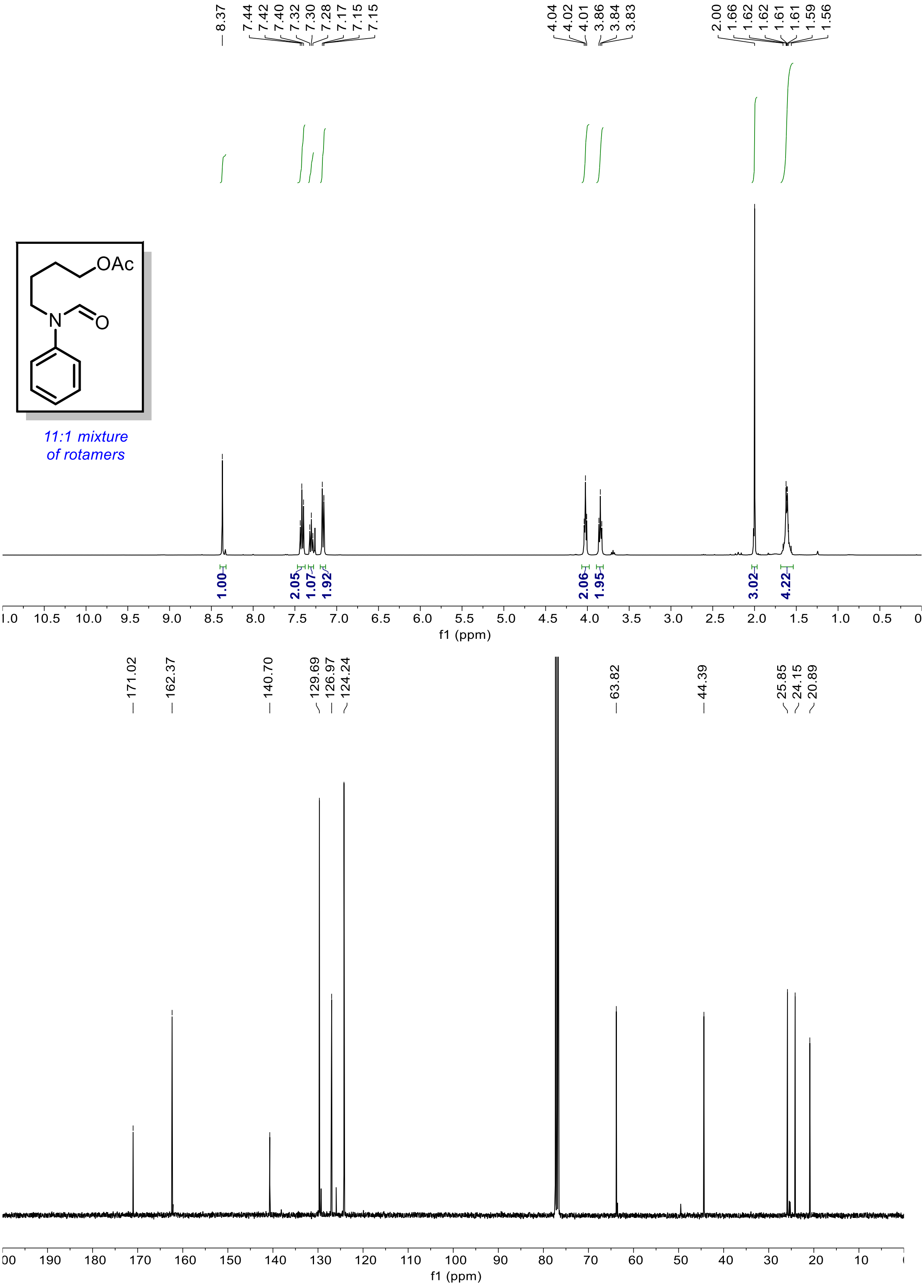

Supplementary Figure 49. ^{1}H and ^{13}C NMR spectra of **2a-OAc**.

***N*-(4-Bromobutyl)-*N*-(3,4-dimethoxyphenethyl)formamide (2s)**

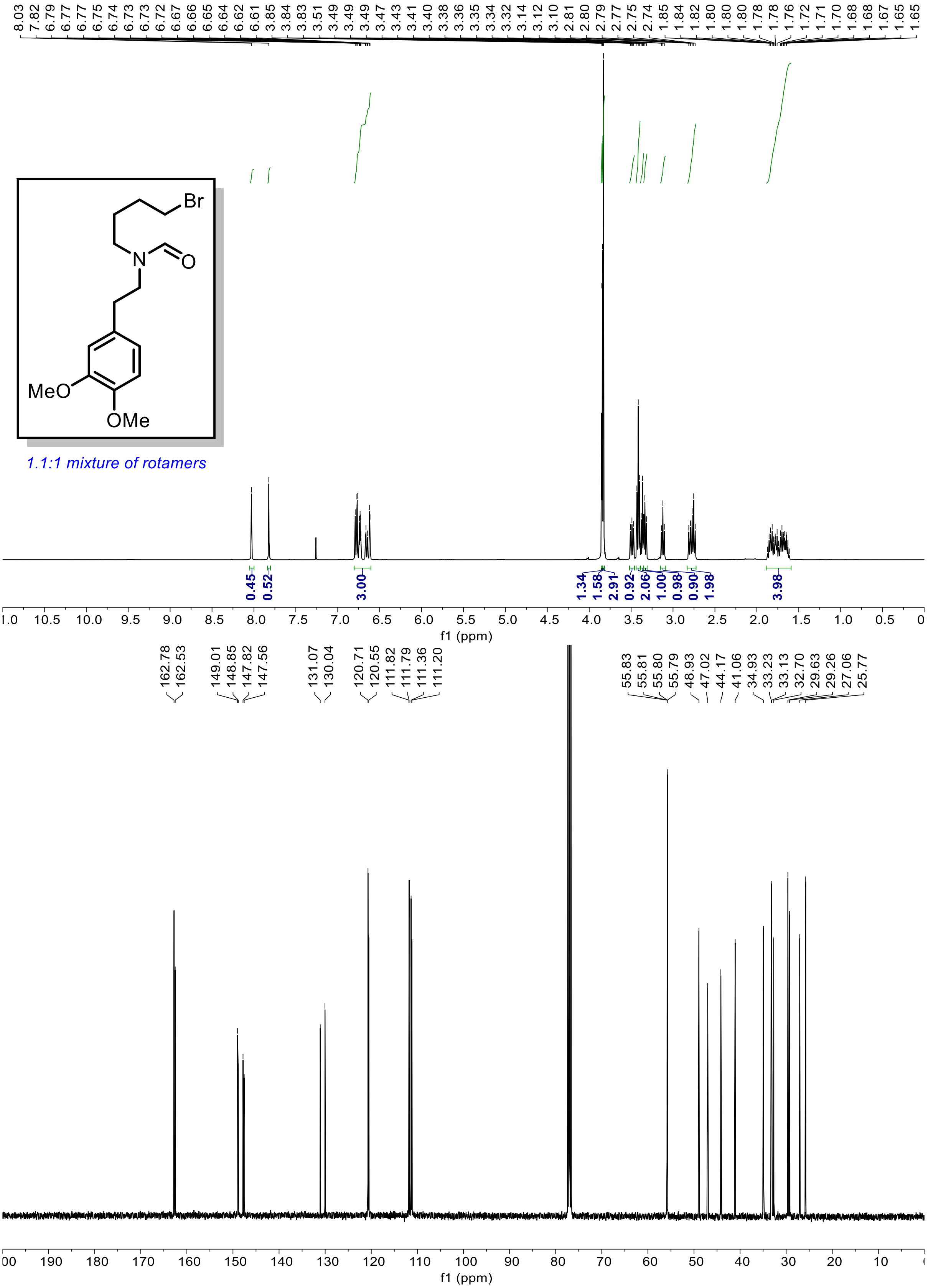

Supplementary Figure 50. ^{1}H and ^{13}C NMR spectra of **2s**.

***N*-(5-Bromopentyl)-*N*-phenylformamide (4a)**

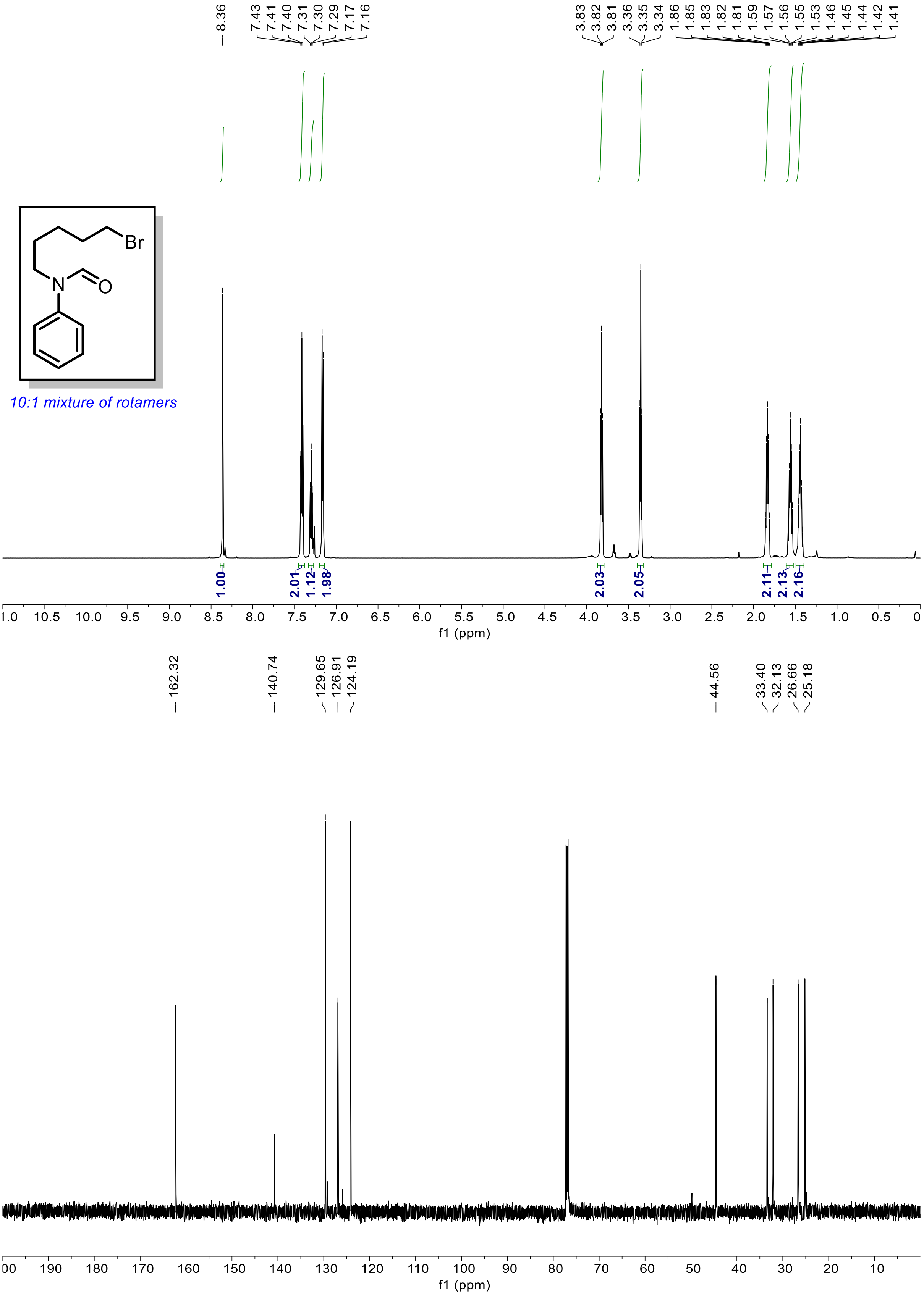

Supplementary Figure 51. ^{1}H and ^{13}C NMR spectra of **4a**.

***N*-(5-Bromopentyl)-*N*-ethylformamide (4b)**

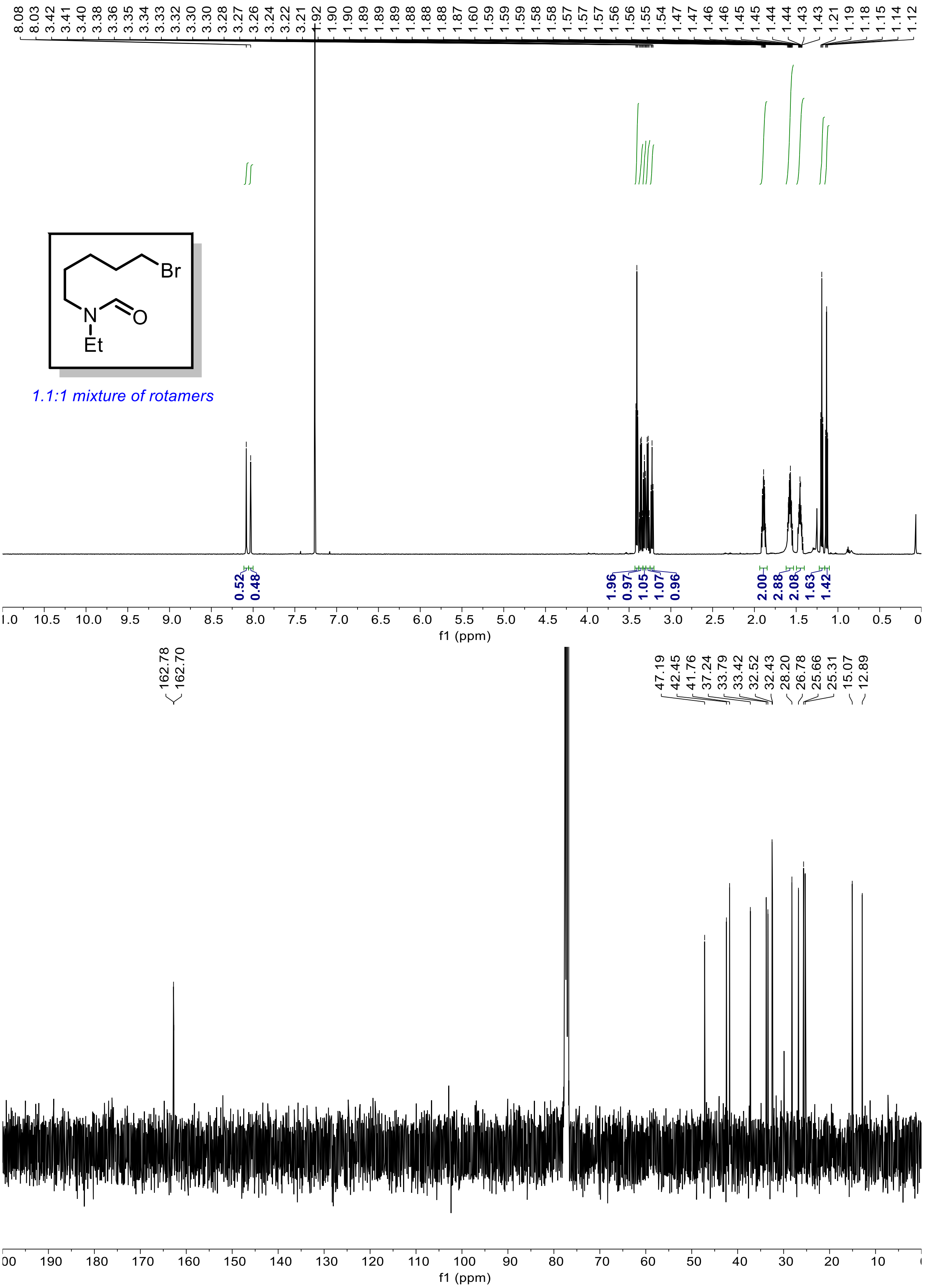

Supplementary Figure 52. ^{1}H and ^{13}C NMR spectra of **4b**.

***N*-(5-Bromopentyl)-*N*-isobutylformamide (4c)**

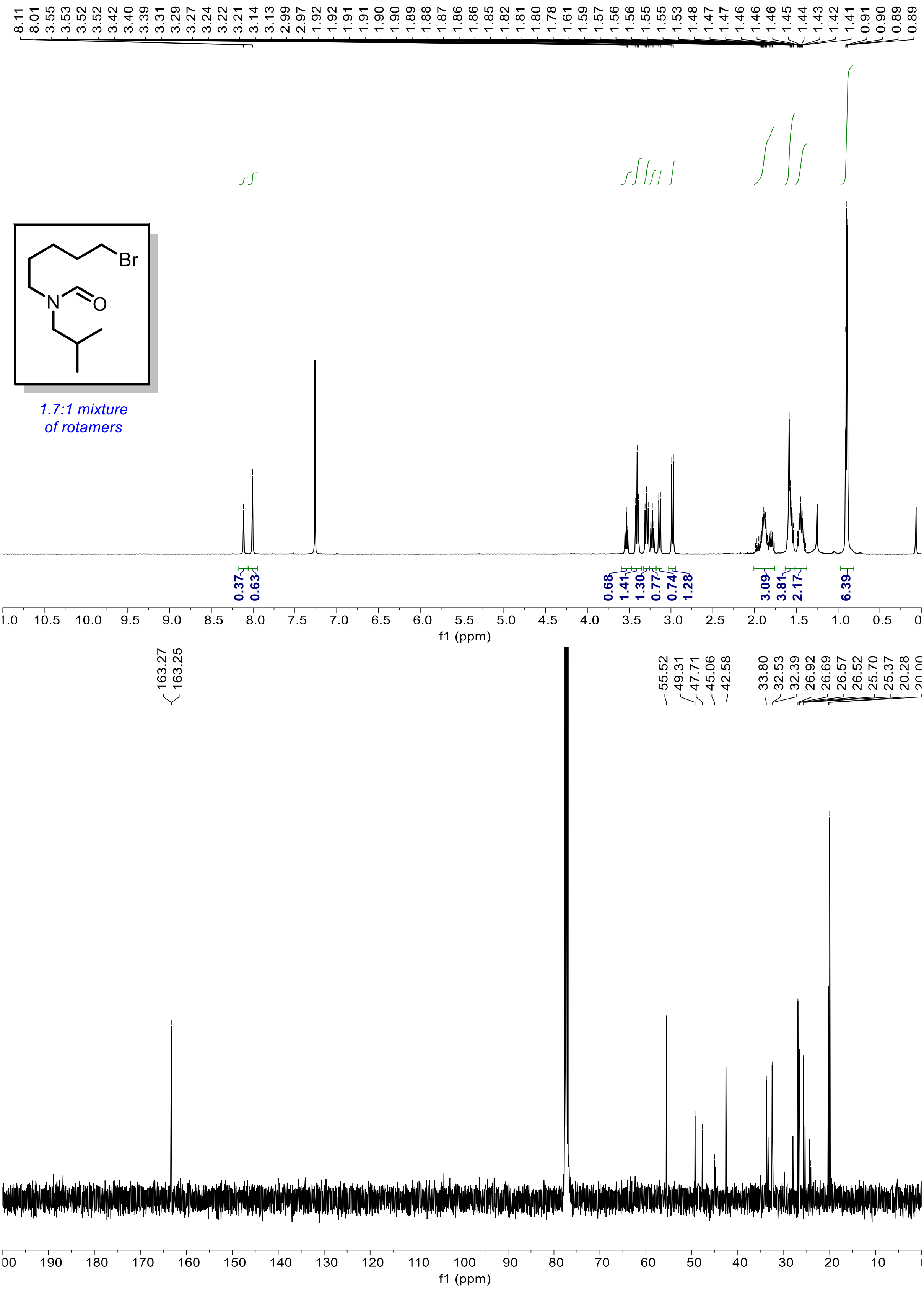

Supplementary Figure 53. ^{1}H and ^{13}C NMR spectra of **4c**.

N-(5-Bromopentyl)-*N*-butylformamide (4d)

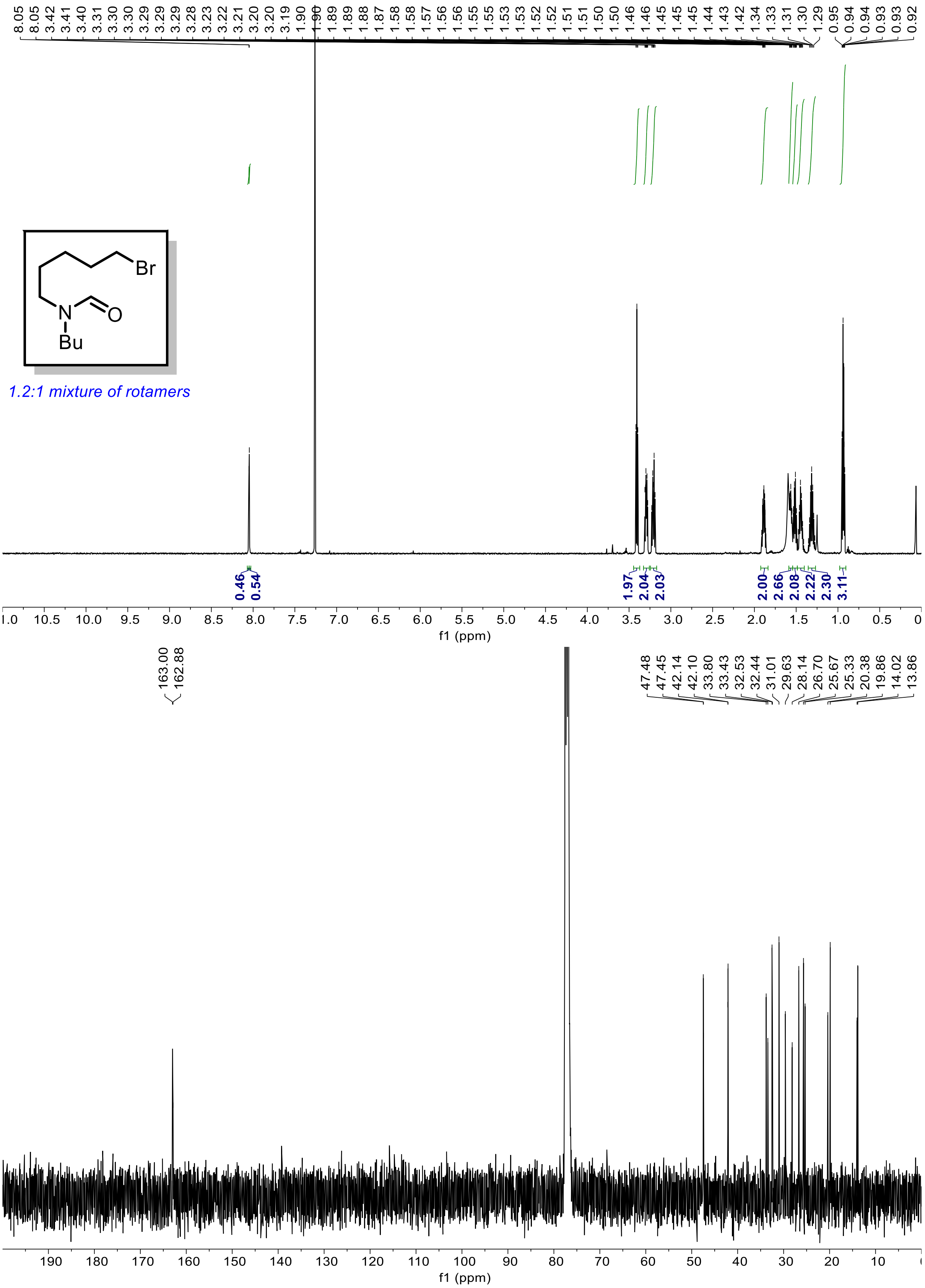

Supplementary Figure 54. ^{1}H and ^{13}C NMR spectra of **4d**.

N-(5-Bromopentyl)-*N*-isopentylformamide (4e)

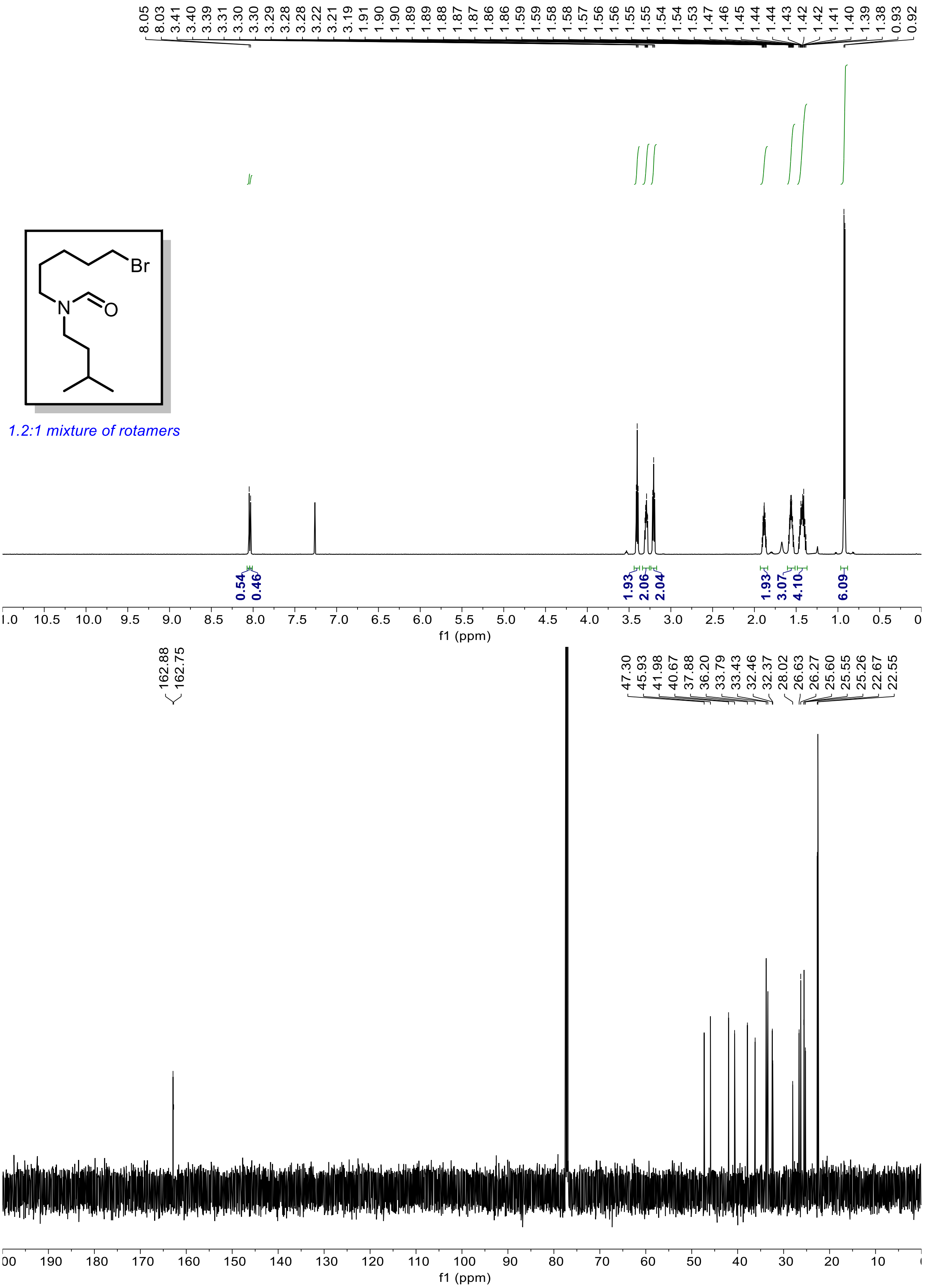

Supplementary Figure 55. ^{1}H and ^{13}C NMR spectra of **4e**.

N-(5-Bromopentyl)-N-(3,3-dimethylbutyl)formamide (4f)

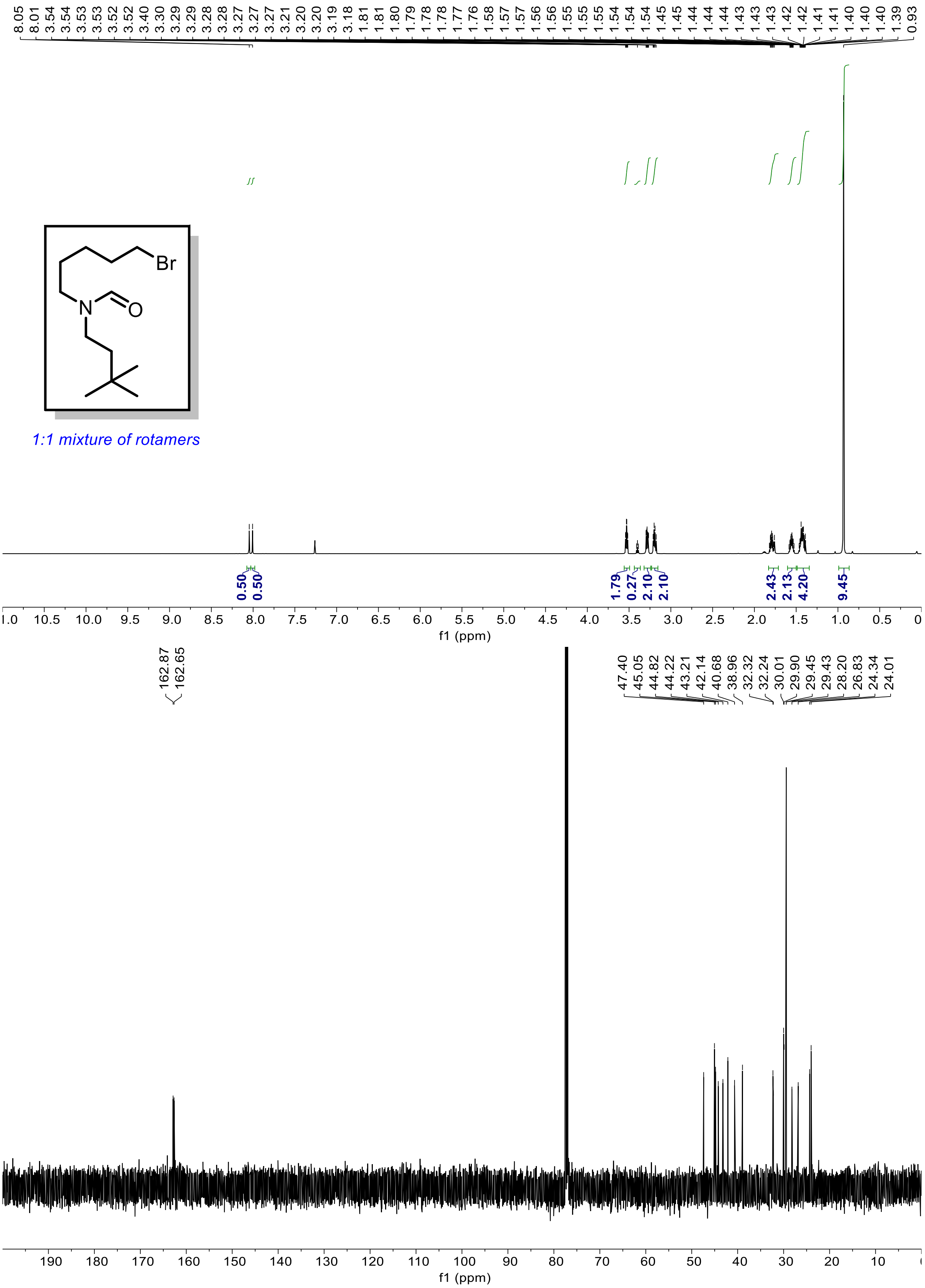

Supplementary Figure 56. ^{1}H and ^{13}C NMR spectra of **4f**.

Ethyl 4-bromo-2-{2-[*N*-(4-methoxyphenyl)formamido]ethyl}butanoate (4g)

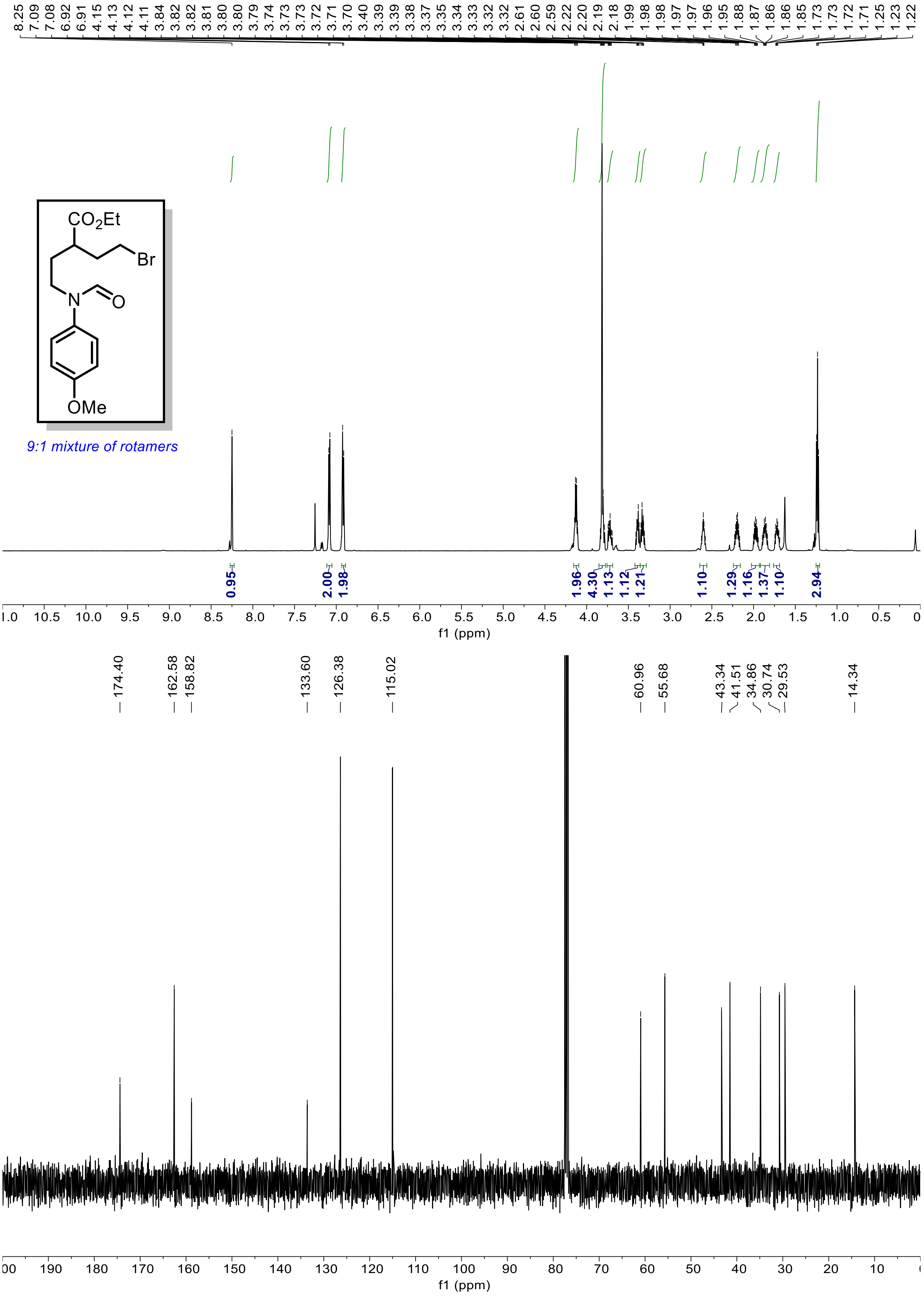

Supplementary Figure 57. ^{1}H and ^{13}C NMR spectra of **4g**.

Methyl 5-bromo-3-{2-(*N*-phenylformamido)ethyl}pentanoate (4h)

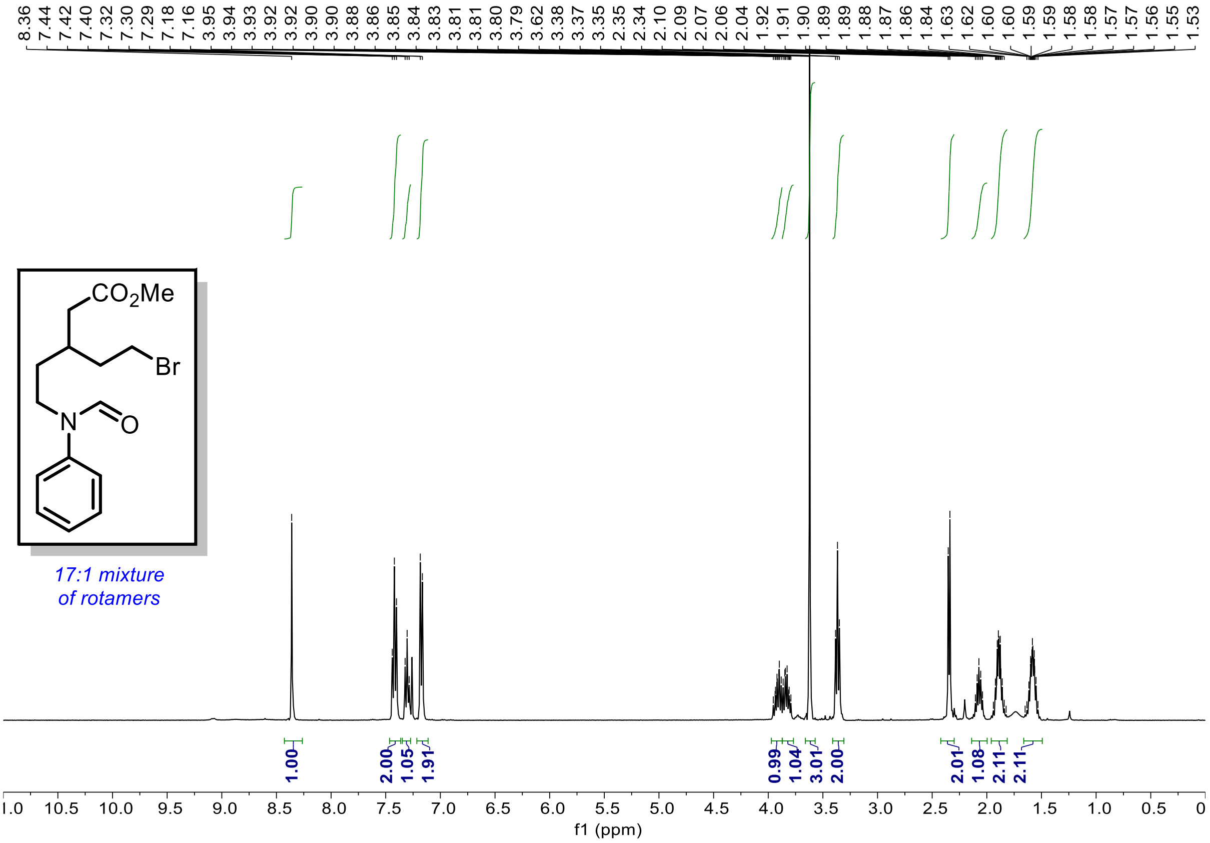

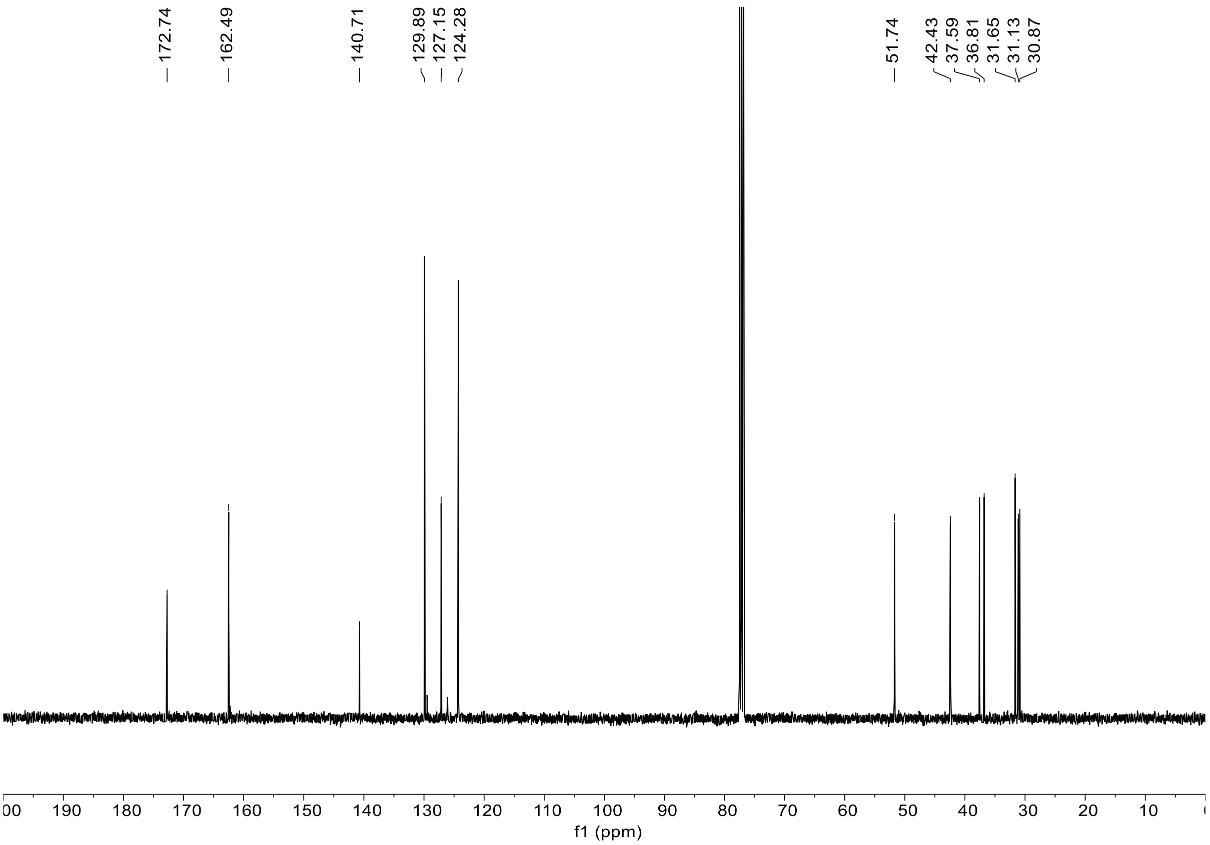

Supplementary Figure 58. ^{1}H and ^{13}C NMR spectra of **4h**.

_N_-{2-[1-(2-Bromoethyl)cyclopentyl]ethyl}-_N_-phenylformamide (4i)

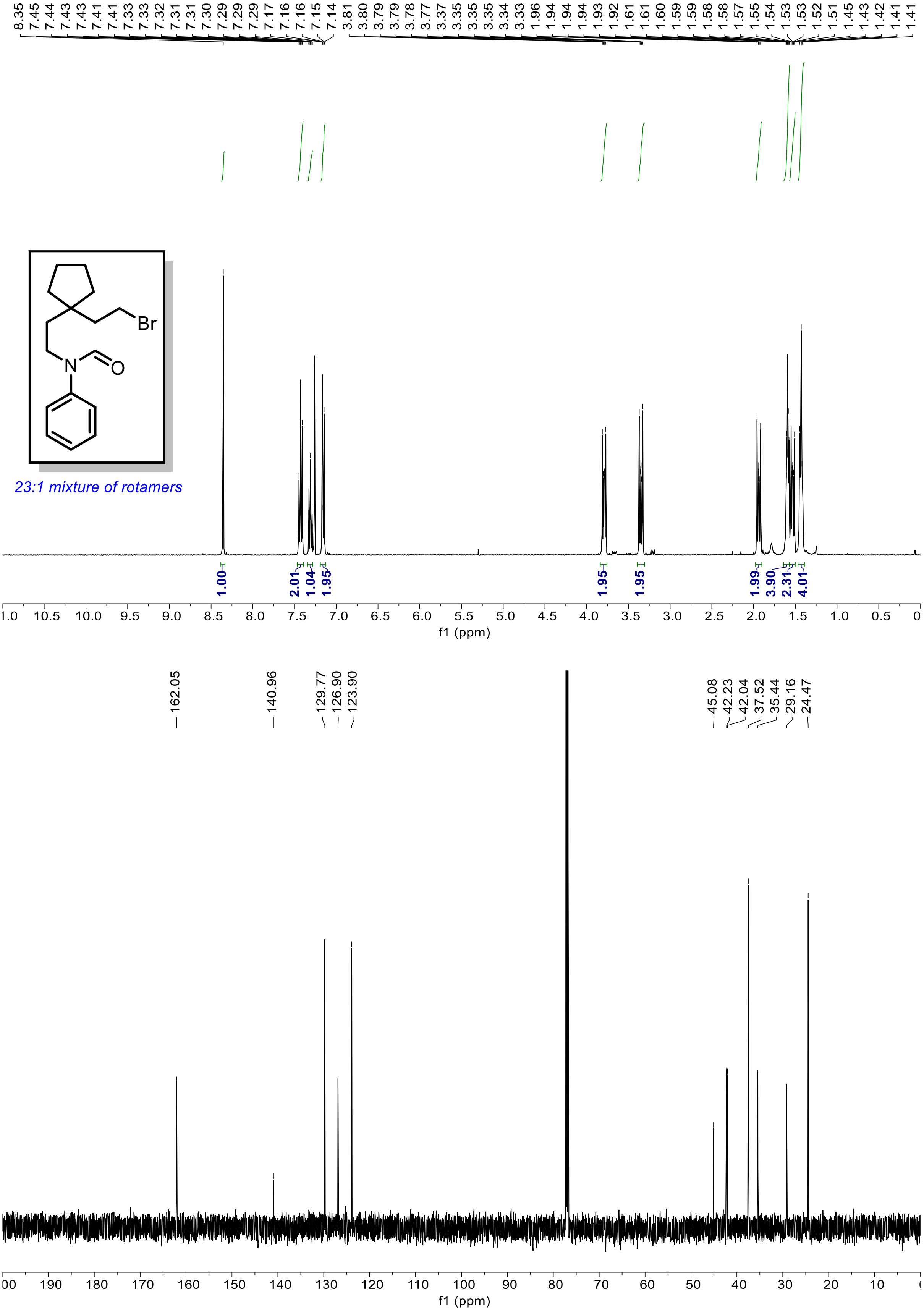

Supplementary Figure 59. ^{1}H and ^{13}C NMR spectra of **4i**.

***N*-[2-(2-Bromoethoxy)ethyl]-*N*-phenylformamide (4j)**

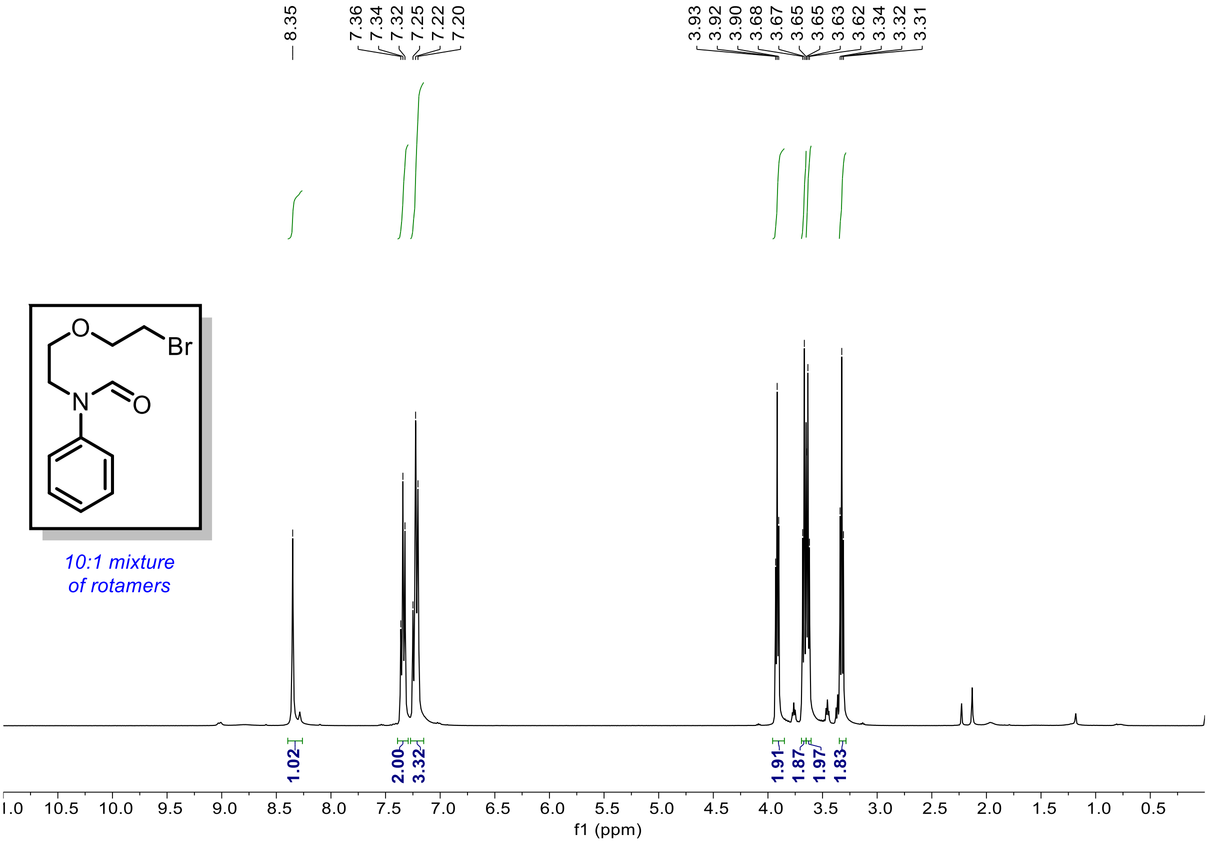

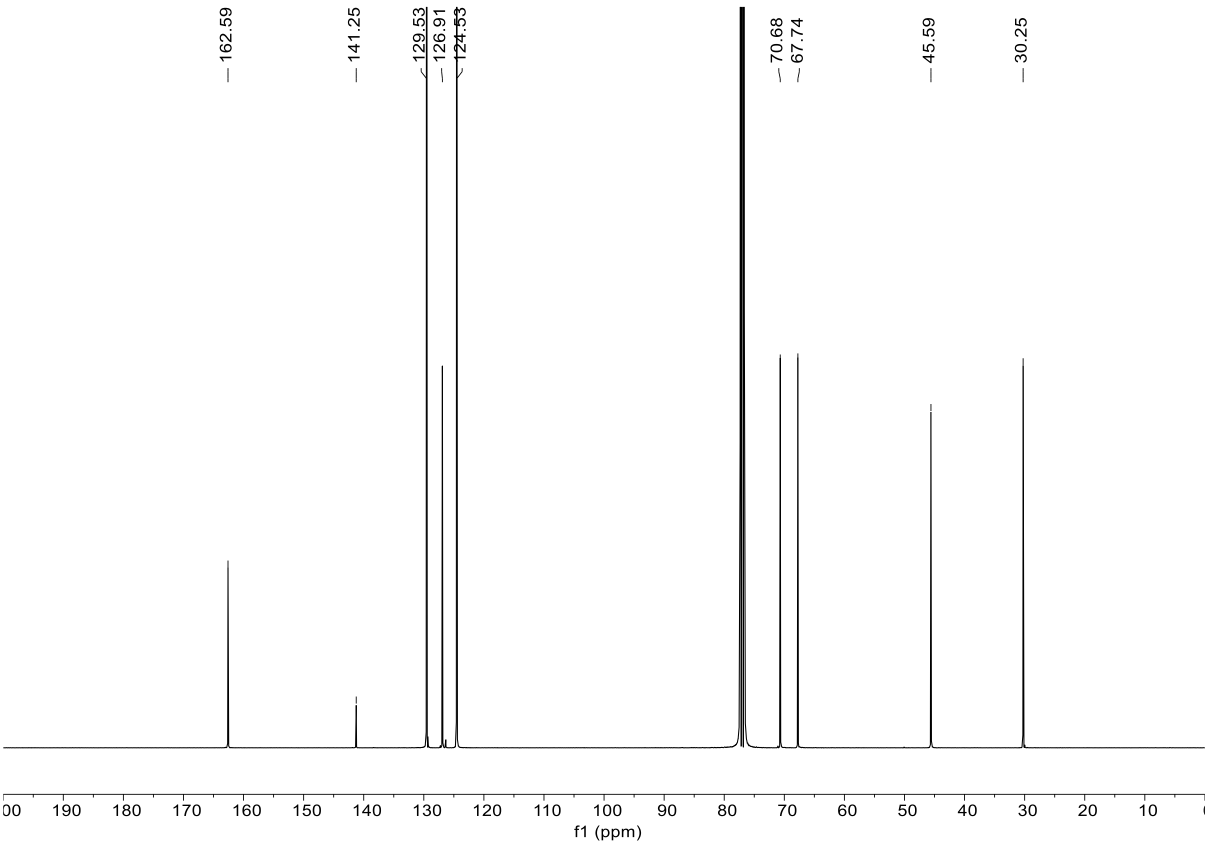

Supplementary Figure 60. ^{1}H and ^{13}C NMR spectra of **4j**.

N-(2-(2-Bromoethoxy)ethyl)-*N*-(3,3-dimethylbutyl)formamide (4k)

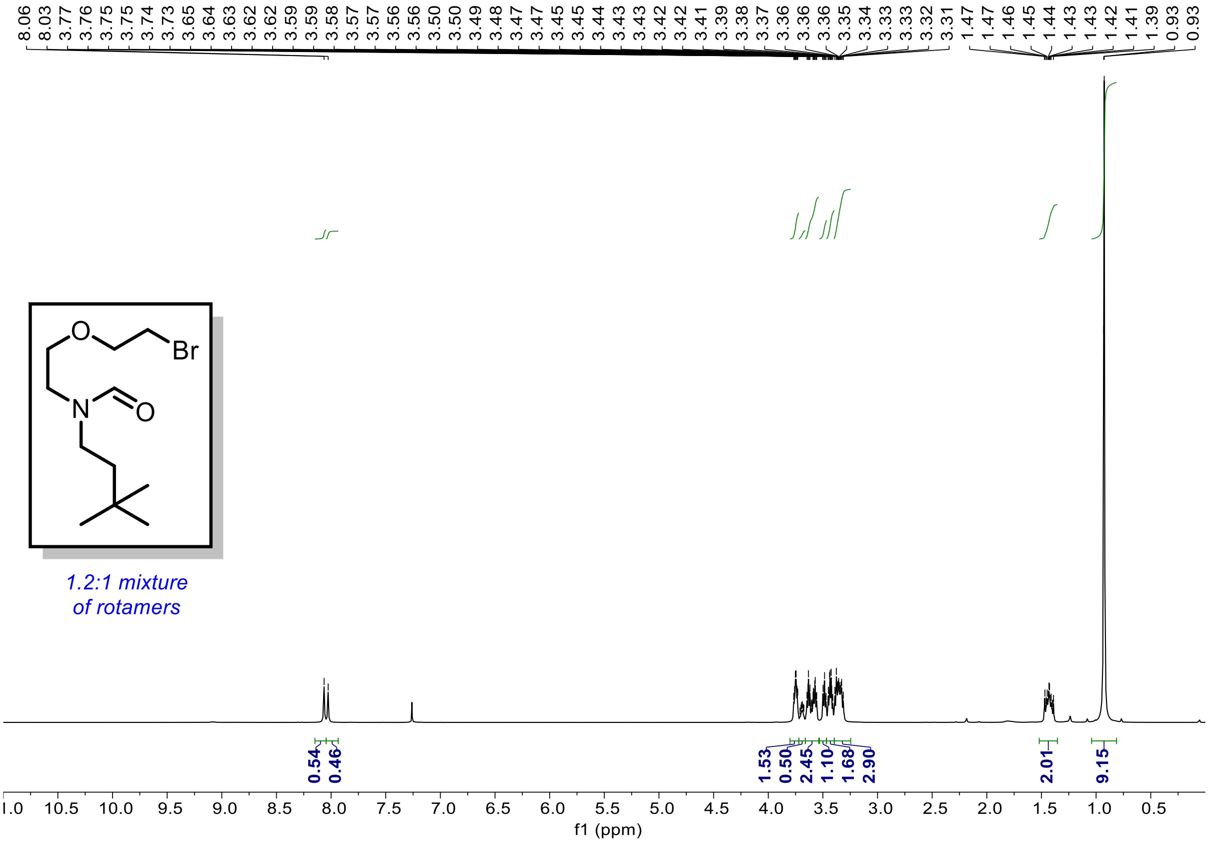

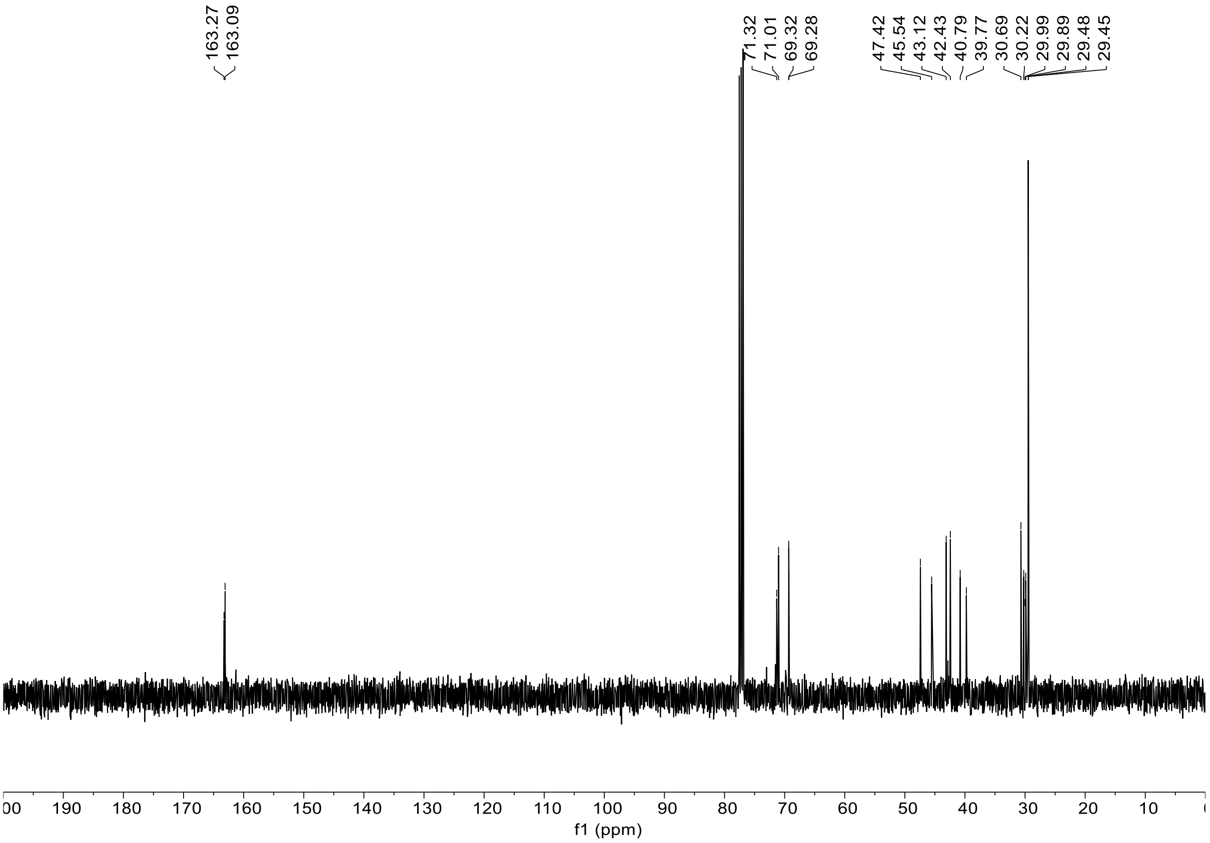

Supplementary Figure 61. ^{1}H and ^{13}C NMR spectra of **4k**.

N-(2-{[N-(2-Bromoethyl)-4-methylphenyl]sulfonamide}ethyl)-N-phenylformamide (4l)

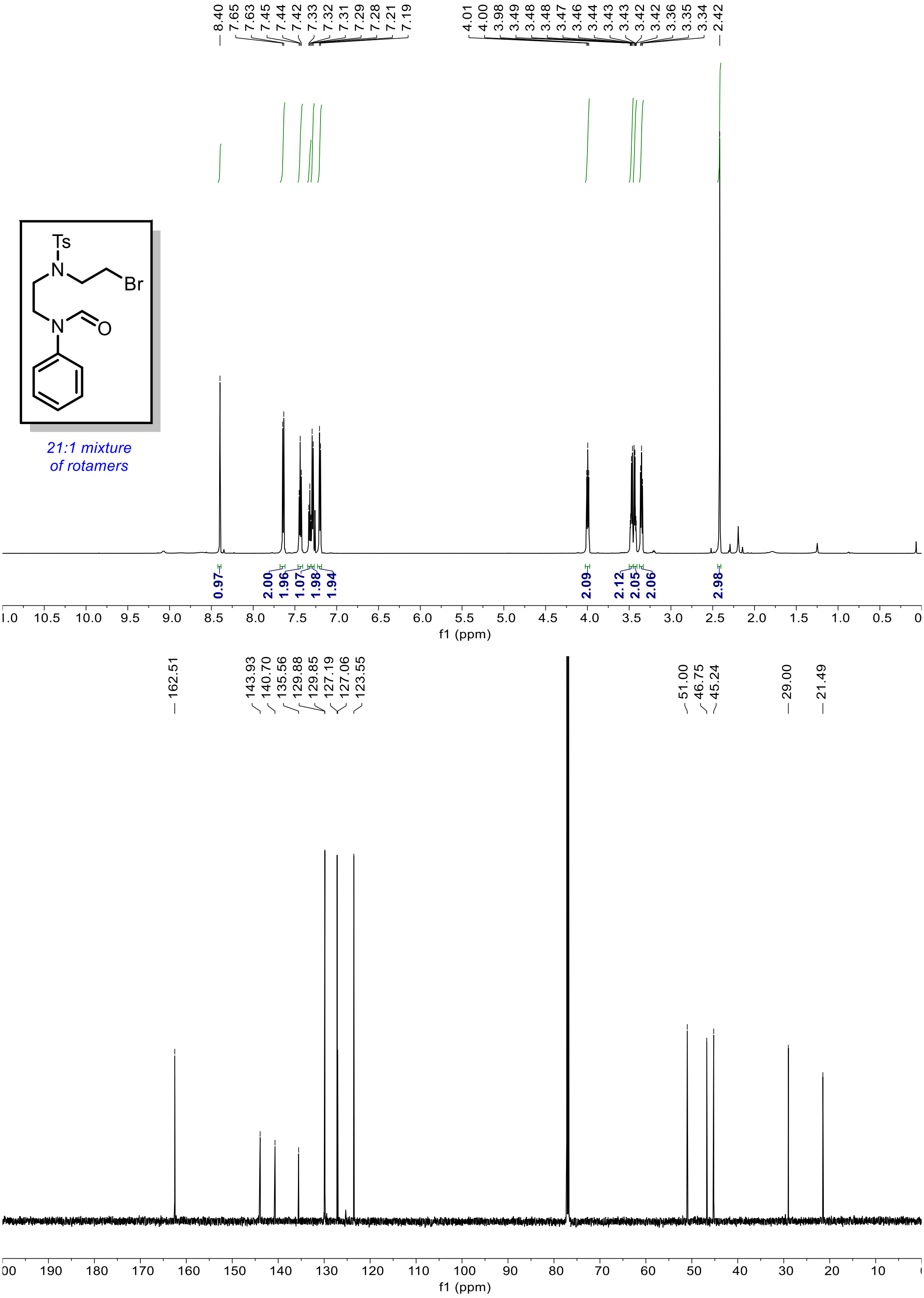

Supplementary Figure 62. ^{1}H and ^{13}C NMR spectra of **4l**.

***N*-(5-Bromopentyl)-*N*-phenylformamide (4m)**

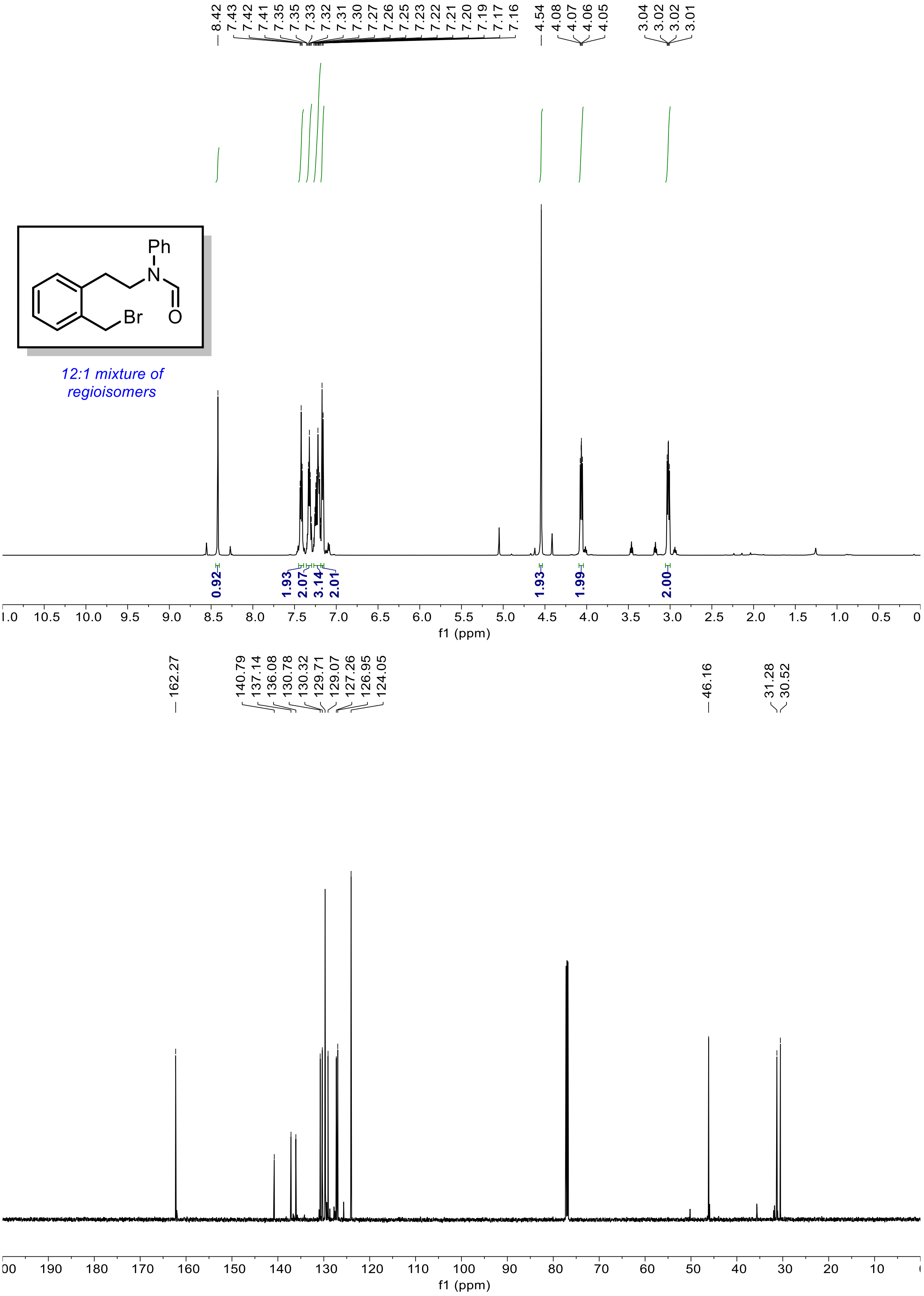

Supplementary Figure 63. ^{1}H and ^{13}C NMR spectra of **4m**.

HSQC (400MHz)

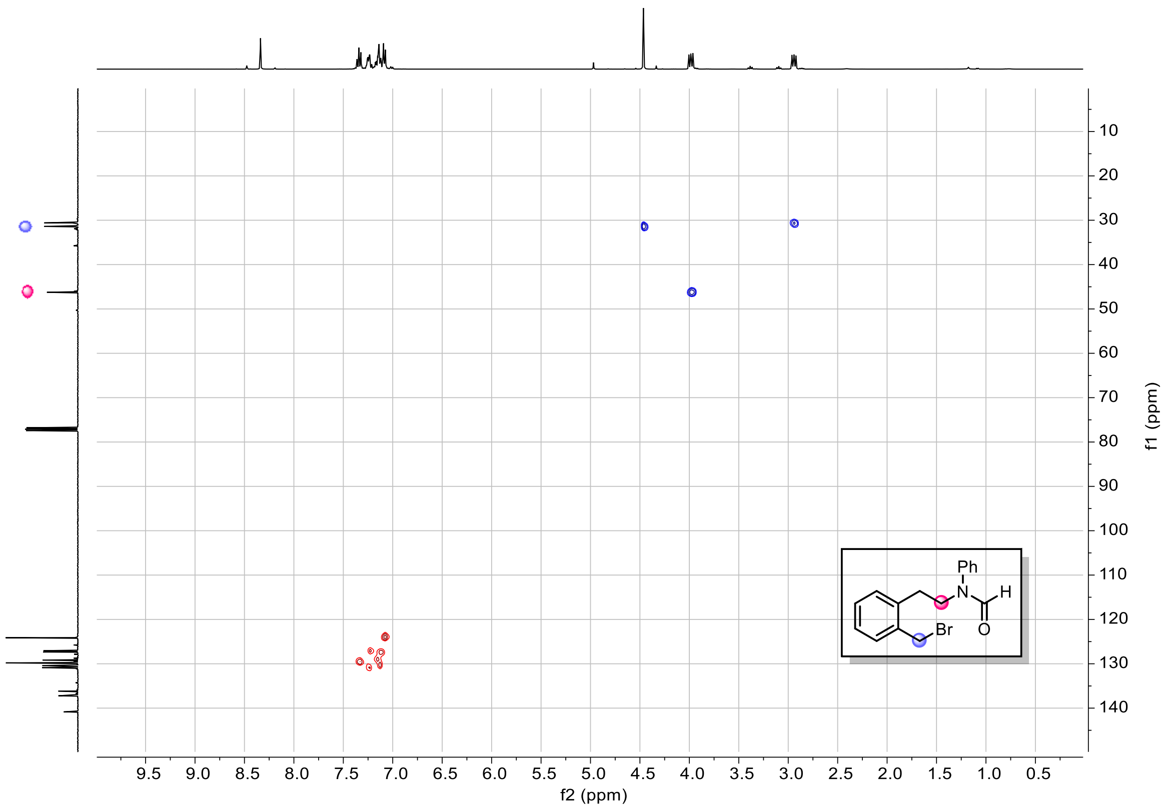

Supplementary Figure 64. HSQC of **4m**.

***trans*-*N*-{[2-(2-Bromoethyl)cyclohexyl]methyl}-*N*-(4-methoxyphenyl)formamide (4n)**

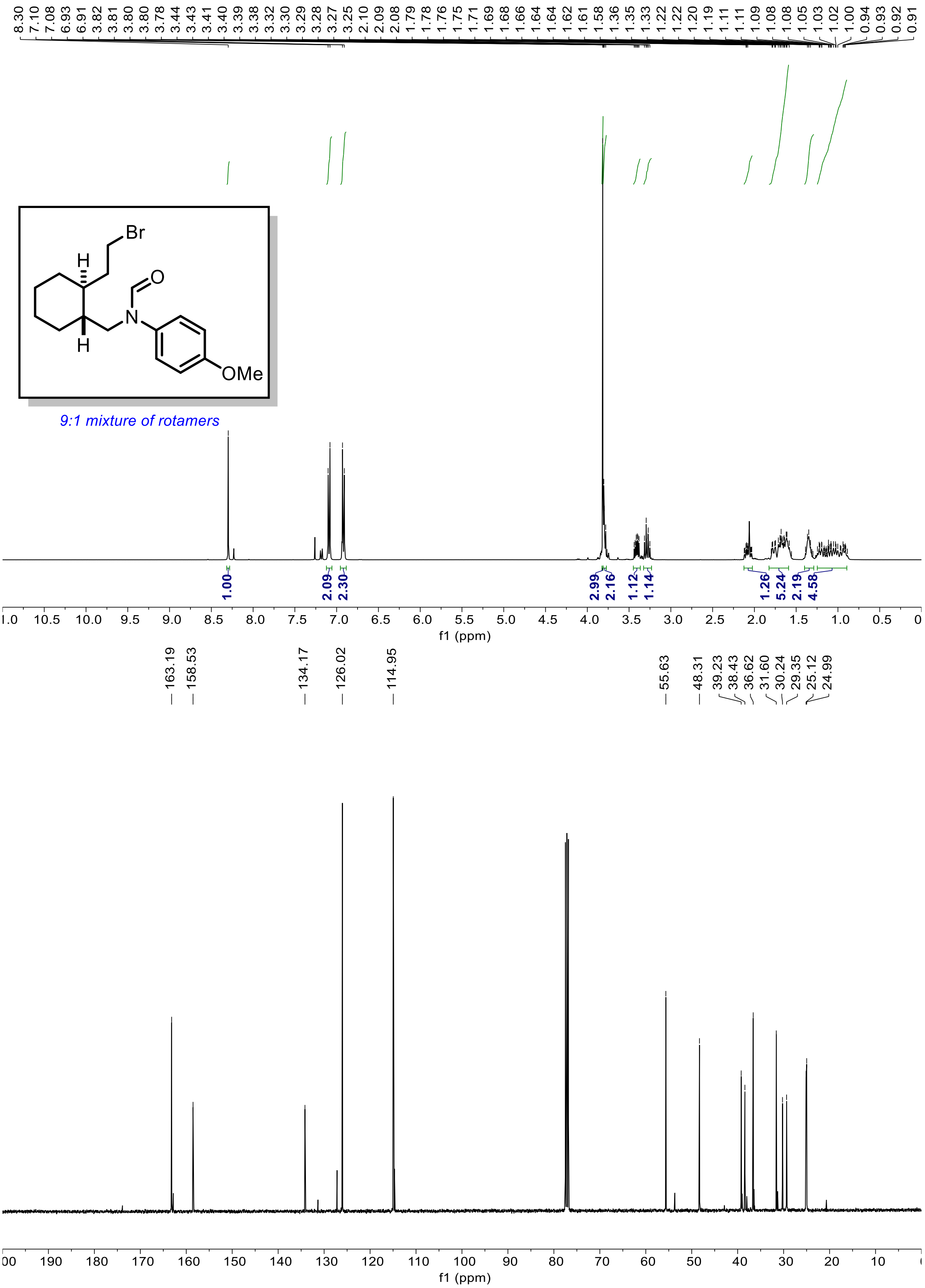

Supplementary Figure 65. ^{1}H and ^{13}C NMR spectra of **4n**.

8-(3-Bromopropyl)-3,4-dihydroquinoline-1(2*H*)-carbaldehyde (4o)

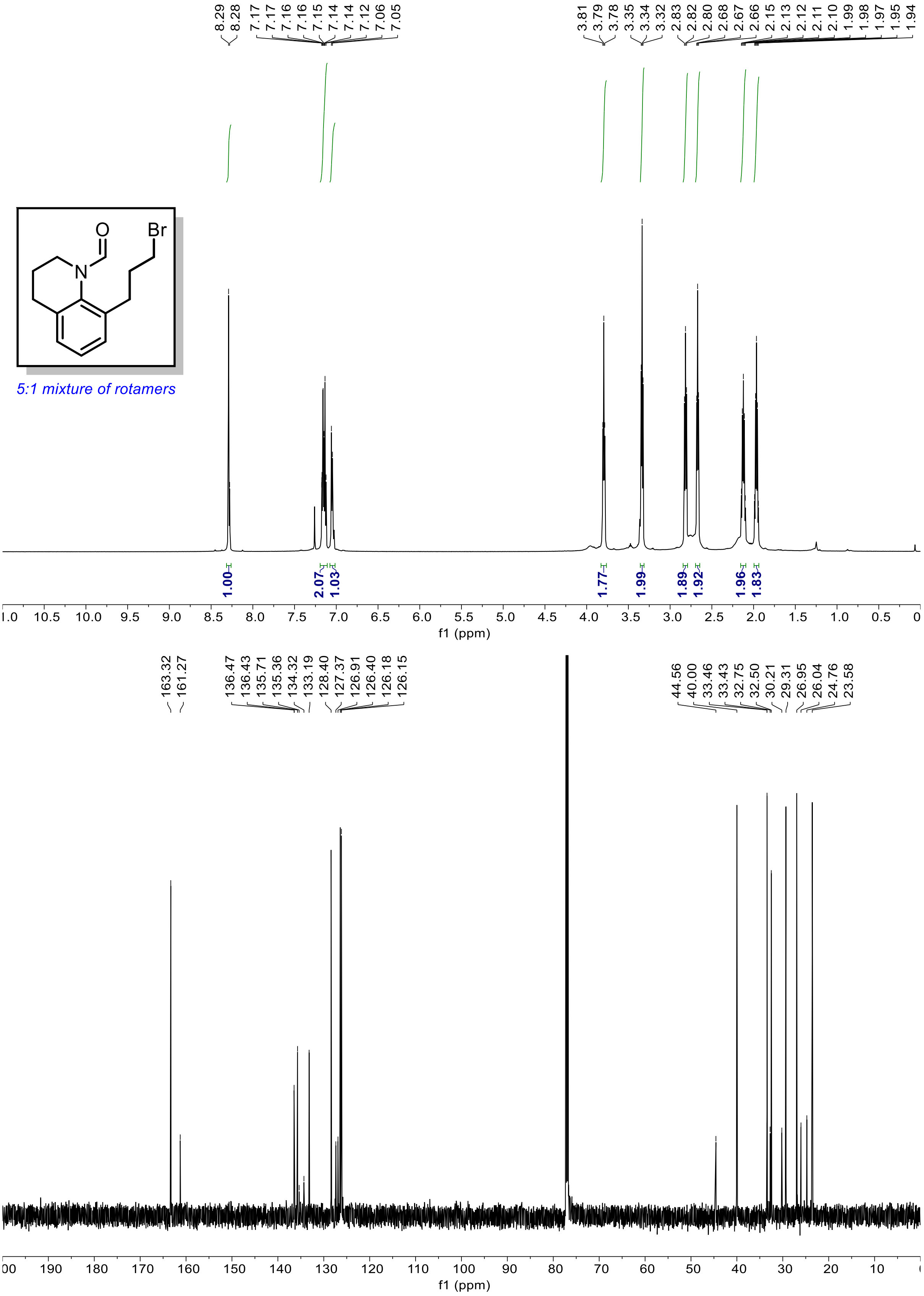

Supplementary Figure 66. ^{1}H and ^{13}C NMR spectra of **4o**.

4-(2-Bromoethyl)piperidine-1-carbaldehyde (4p)

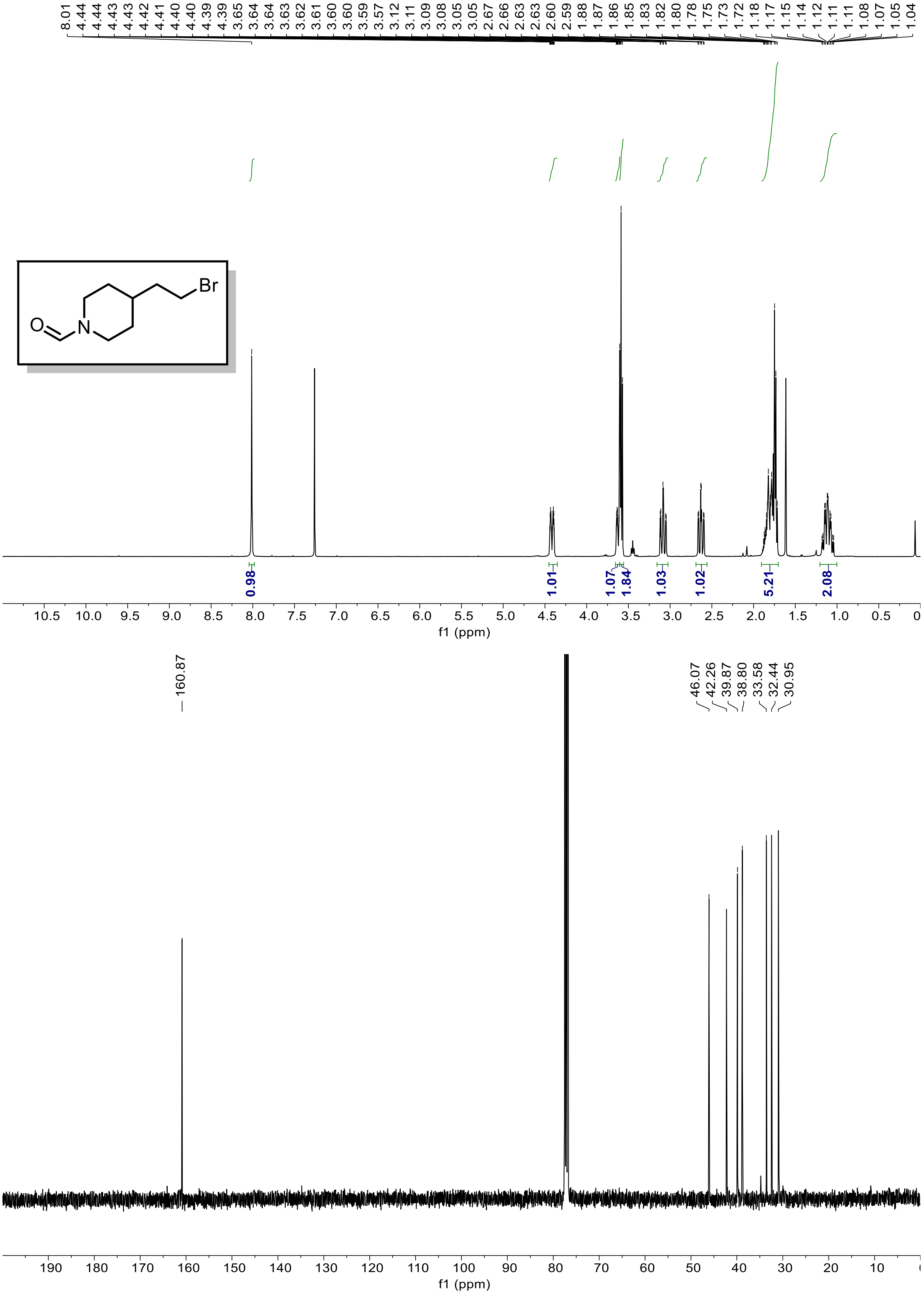

Supplementary Figure 67. ^{1}H and ^{13}C NMR spectra of **4p**.

Methyl (*Z*)-2-methylene-5-(*N*-methylformamido)pent-3-enoate (4q)

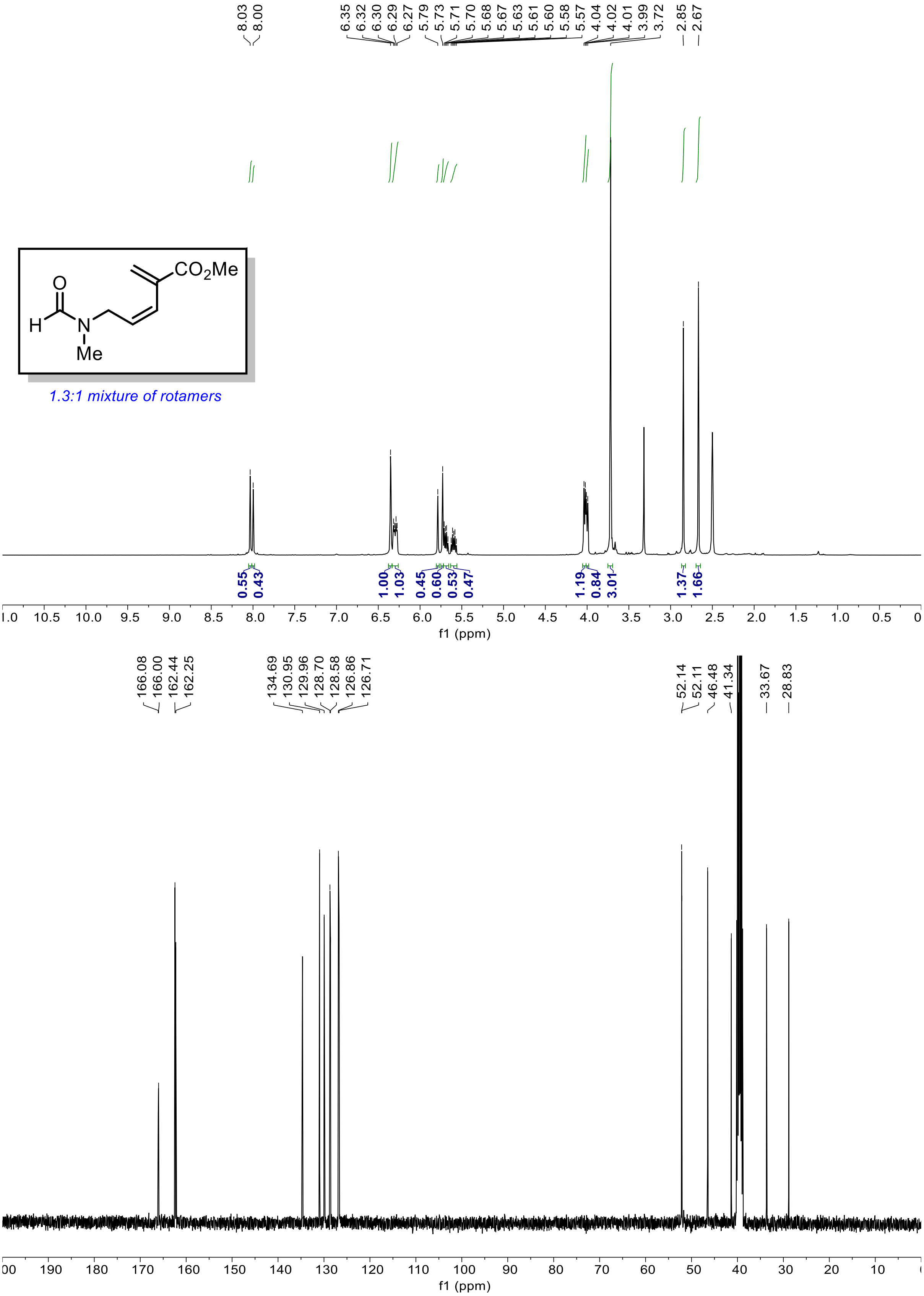

Supplementary Figure 68. ^{1}H and ^{13}C NMR spectra of **4q**.

NOESY (400MHz)

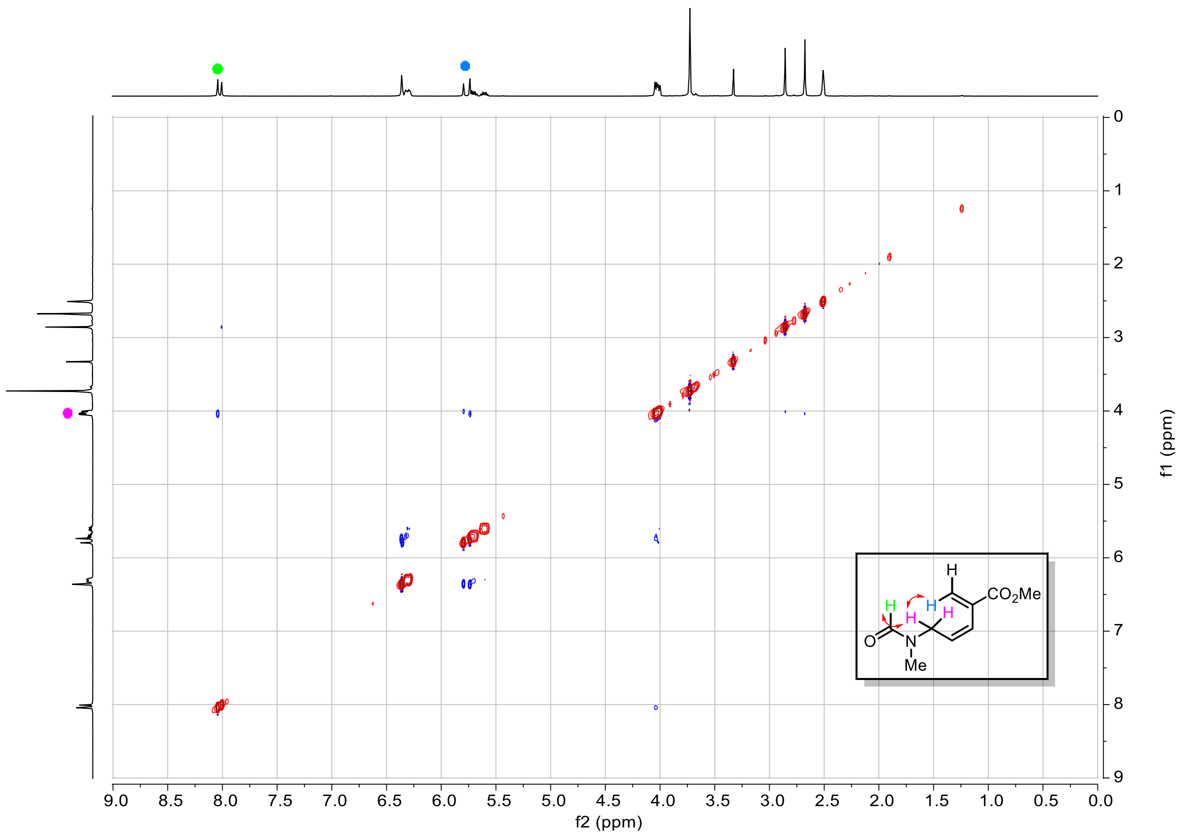

HMBC (400MHz)

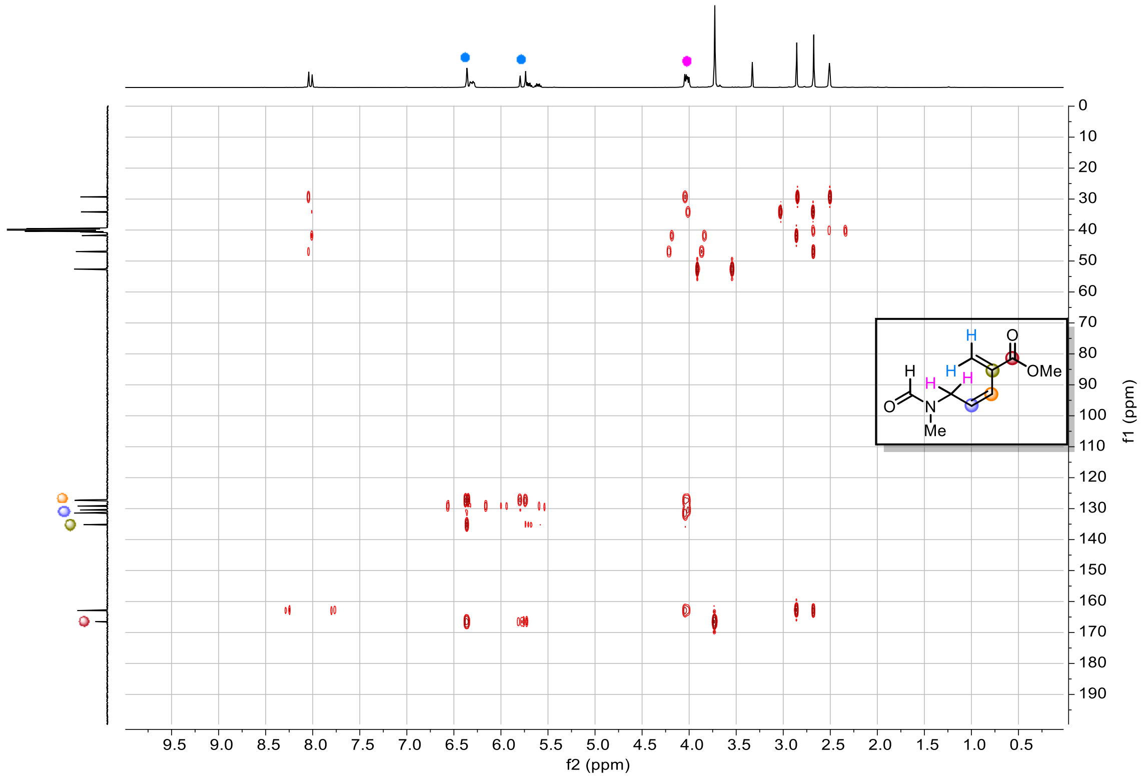

Supplementary Figure 69. NOESY and HMBC of **4q**.

***N*-(5-Bromo-5-phenylpentyl)-*N*-butylformamide (4r)**

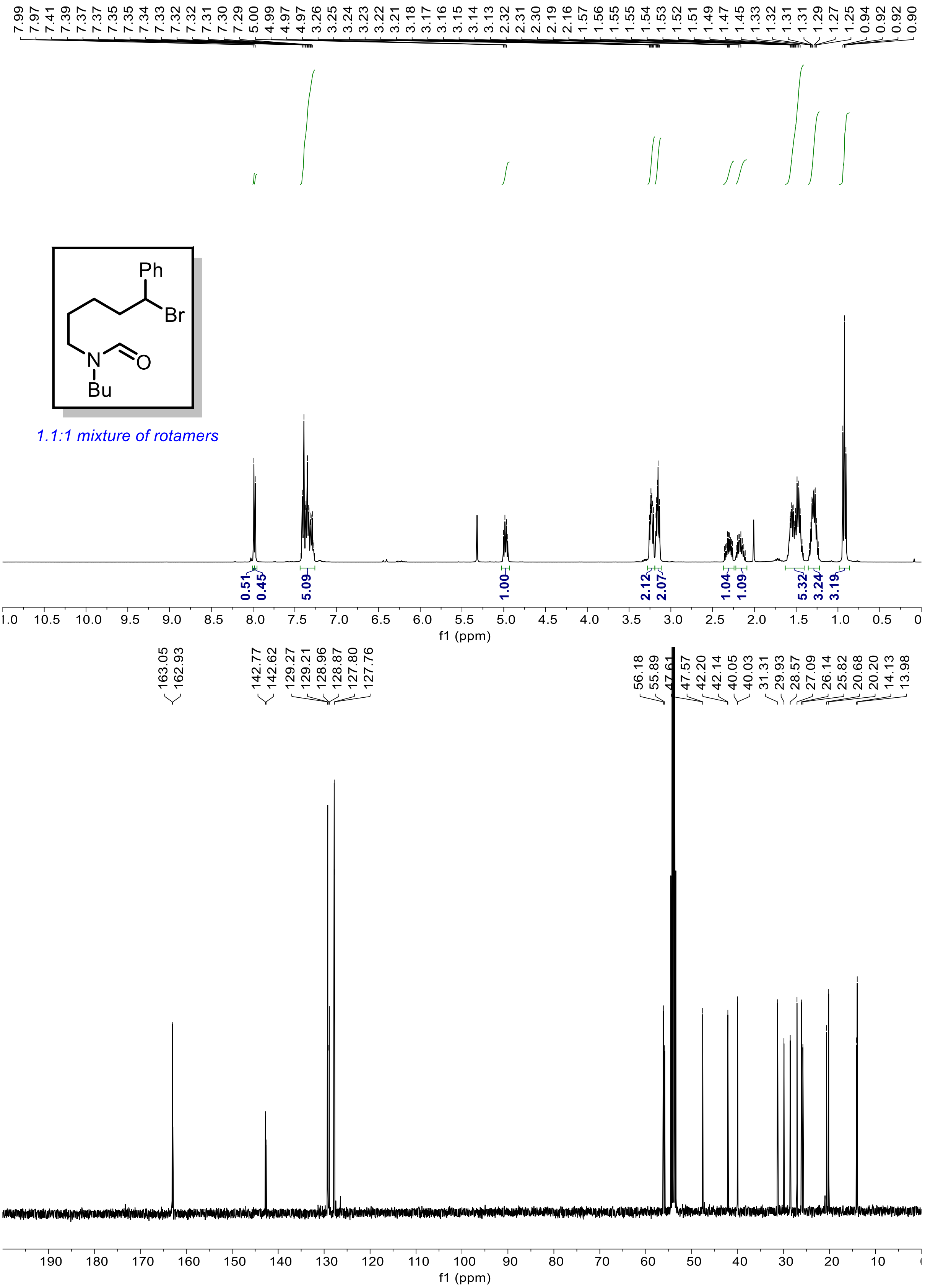

Supplementary Figure 70. ^{1}H and ^{13}C NMR spectra of **4r**.

HMBC (400MHz)

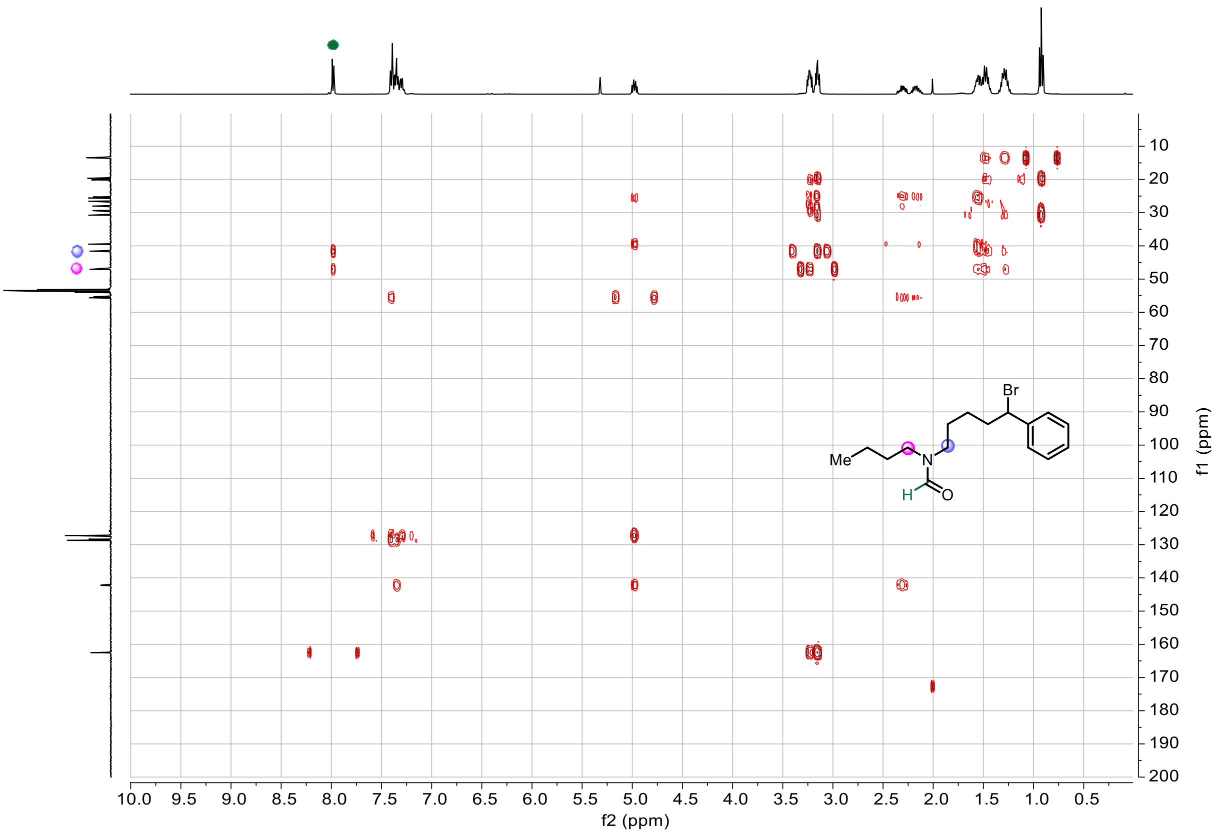

Supplementary Figure 71. HMBC of **4r**.

***N*-(6-Bromohexyl)-*N*-phenylformamide (7a)**

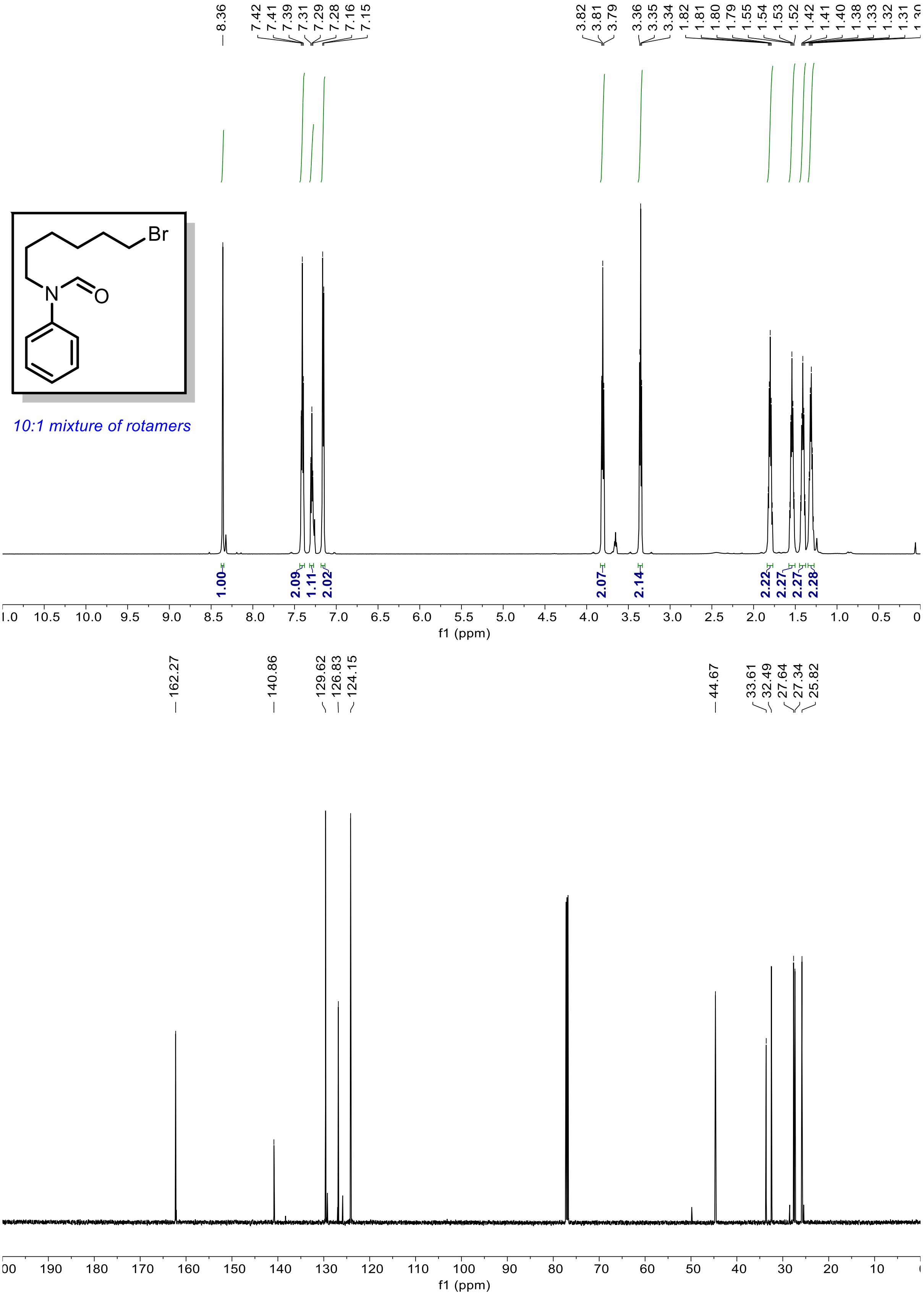

Supplementary Figure 72 ^{1}H and ^{13}C NMR spectra of **7a**.

***N*-(6-Bromohexyl)-*N*-butylformamide (7b)**

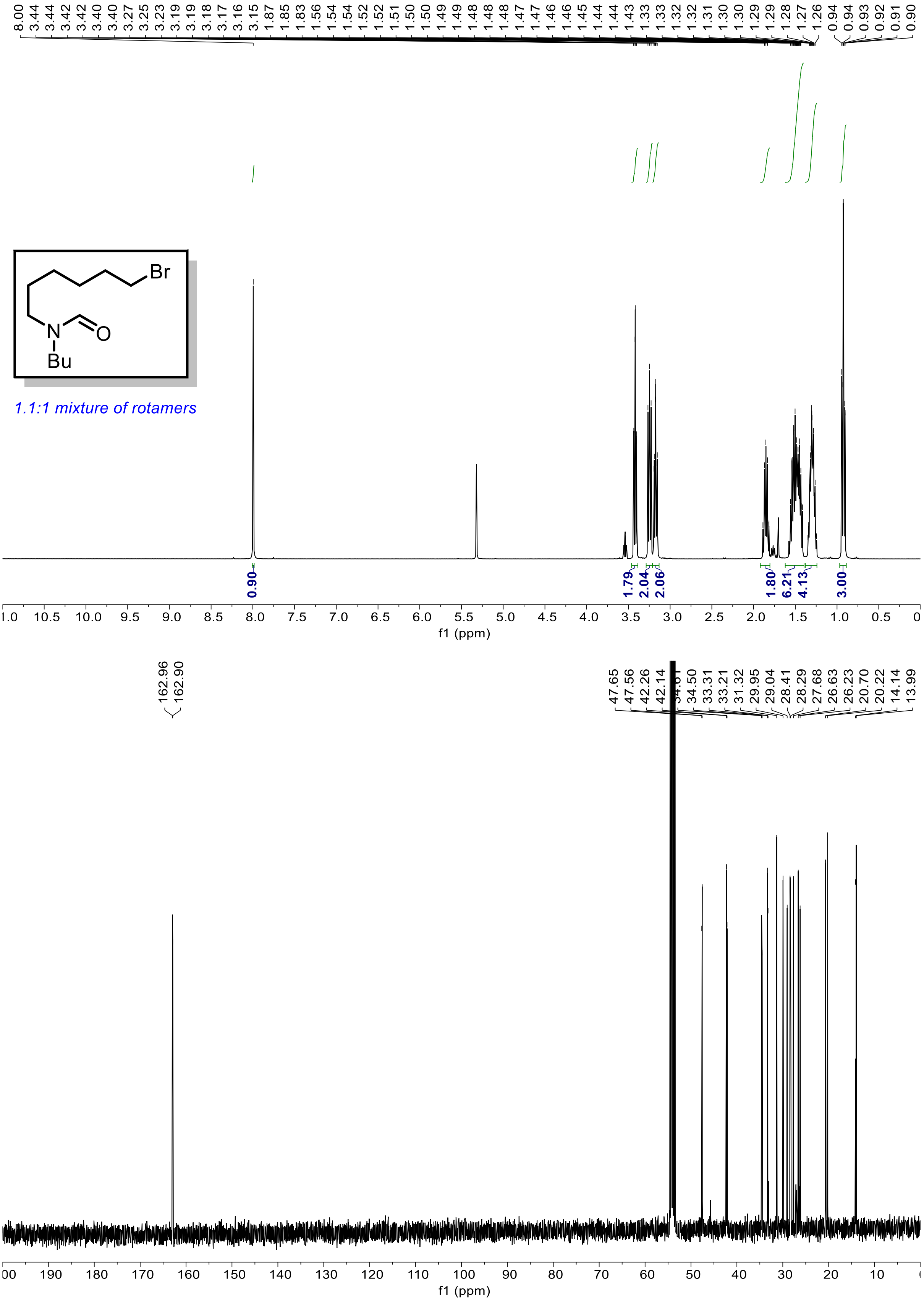

Supplementary Figure 73. ^{1}H and ^{13}C NMR spectra of **7b**.

N-(7-Bromoheptyl)-*N*-phenylformamide (9a)

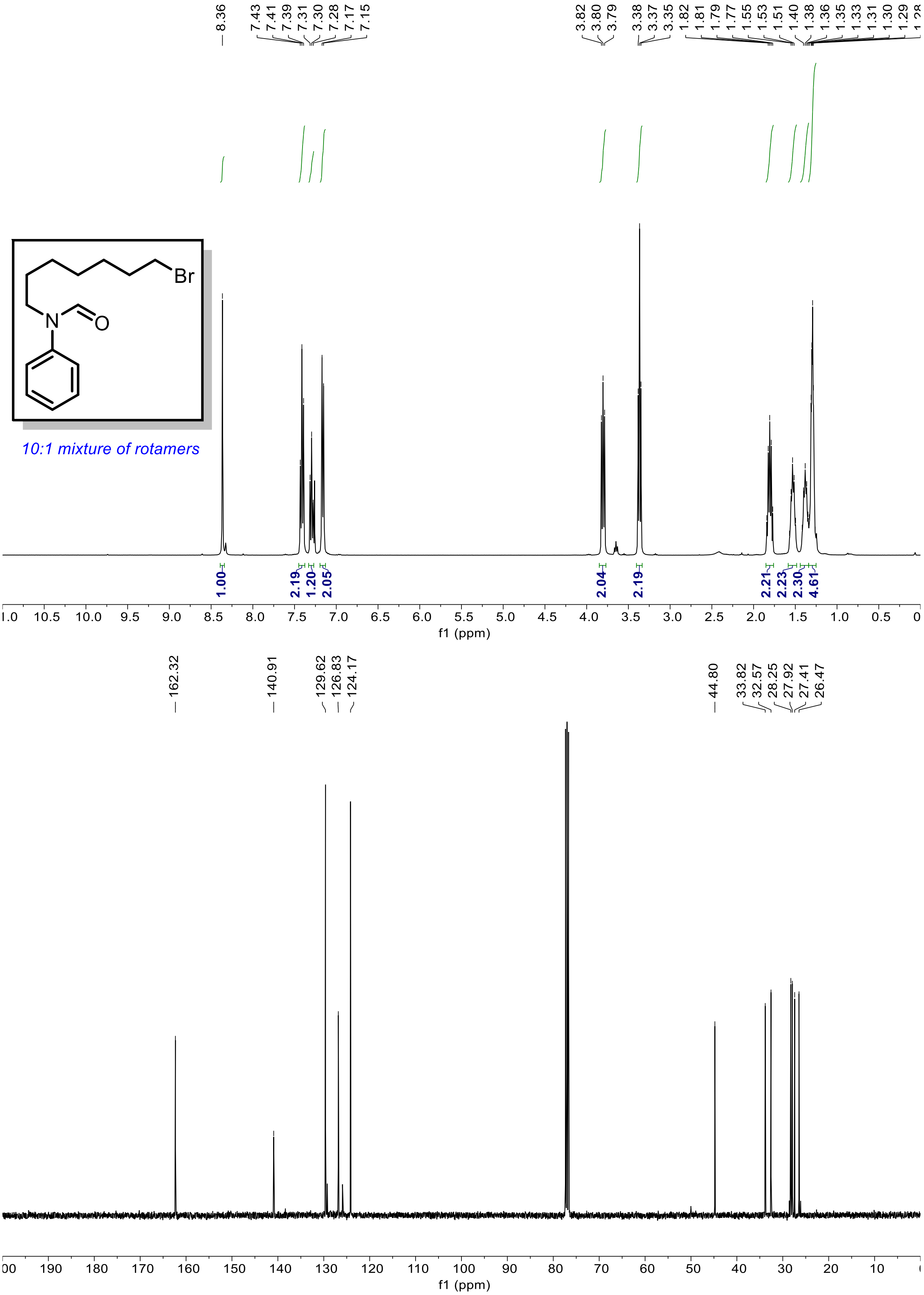

Supplementary Figure 74. ^{1}H and ^{13}C NMR spectra of **9a**.

***N*-(7- Bromoheptyl)-*N*-butylformamide (9b)**

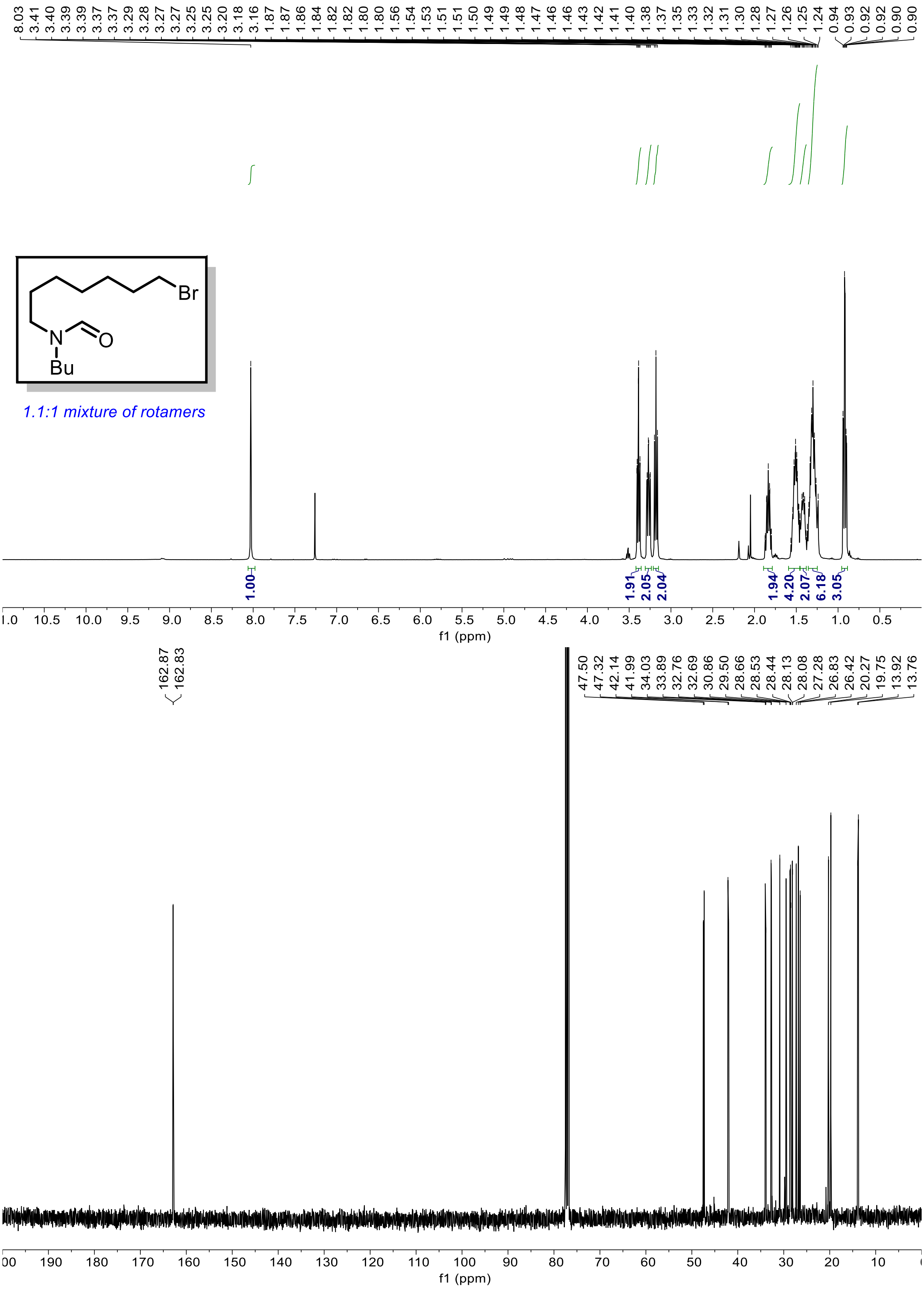

Supplementary Figure 75. ^{1}H and ^{13}C NMR spectra of **9b**.

***N*-{2-[(*N*-(2-bromoethyl)-4-ethoxy-3-(1-methyl-7-oxo-3-propyl-6,7-dihydro-1*H*-pyrazolo[4,3-*d*]pyrimidin-5-yl)phenyl)sulfonamide]ethyl}-*N*-phenylformamide (11)**

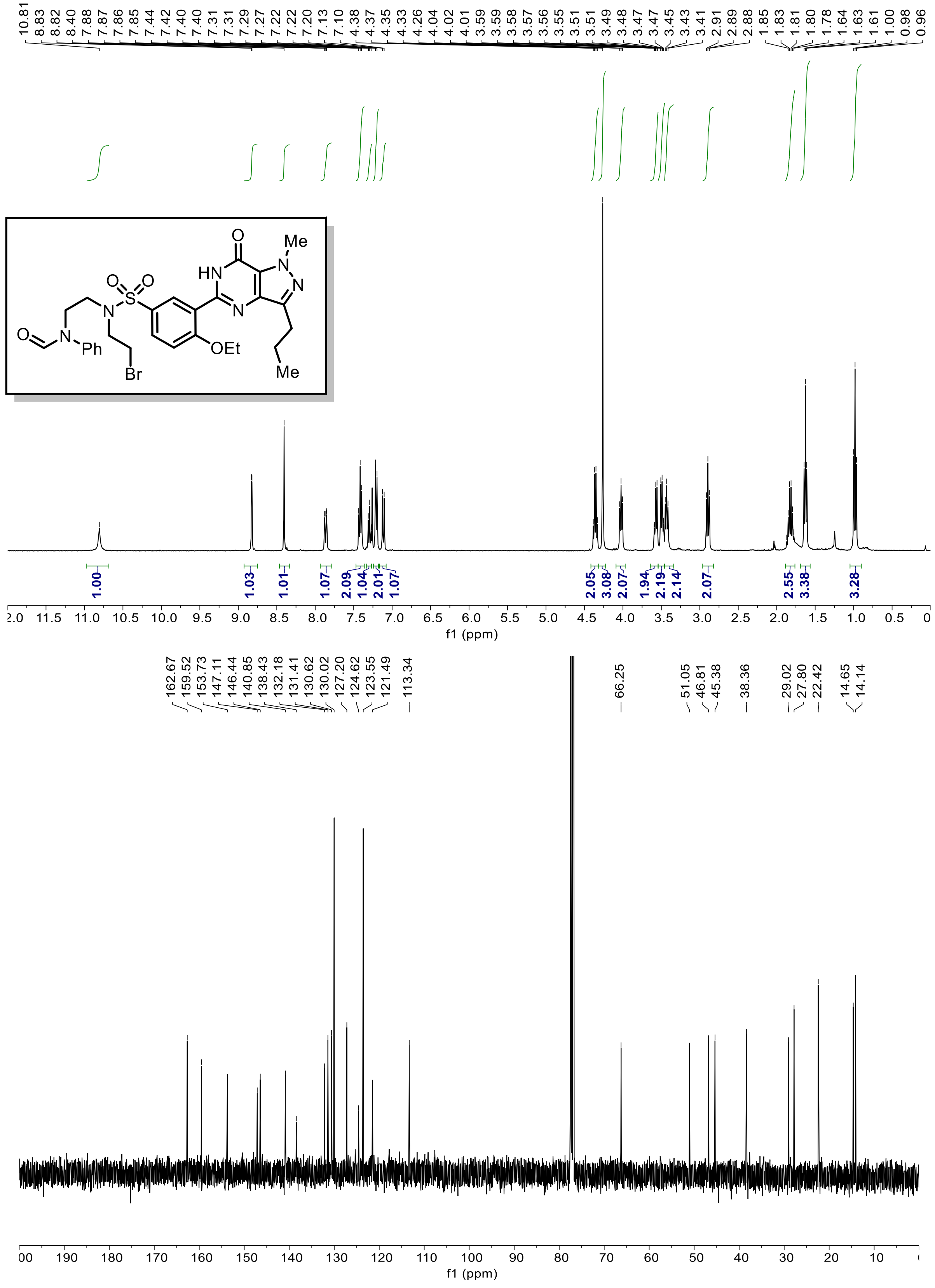

Supplementary Figure 76. ^{1}H and ^{13}C NMR spectra of **11**.

Methyl (1*S*,2*R*,3*R*,4a*S*,14*S*,15a*S*)-14-acetoxy-6-formyl-2,11-dimethoxy-3-[(3,4,5-trimethoxy-benzoyl)oxy]-2,3,4,4a,5,6,7,8,13,14,15,15a-dodecahydro-1*H*-benzo[8,9]azecino[5,4-*b*]indole-1-carboxylate (12)

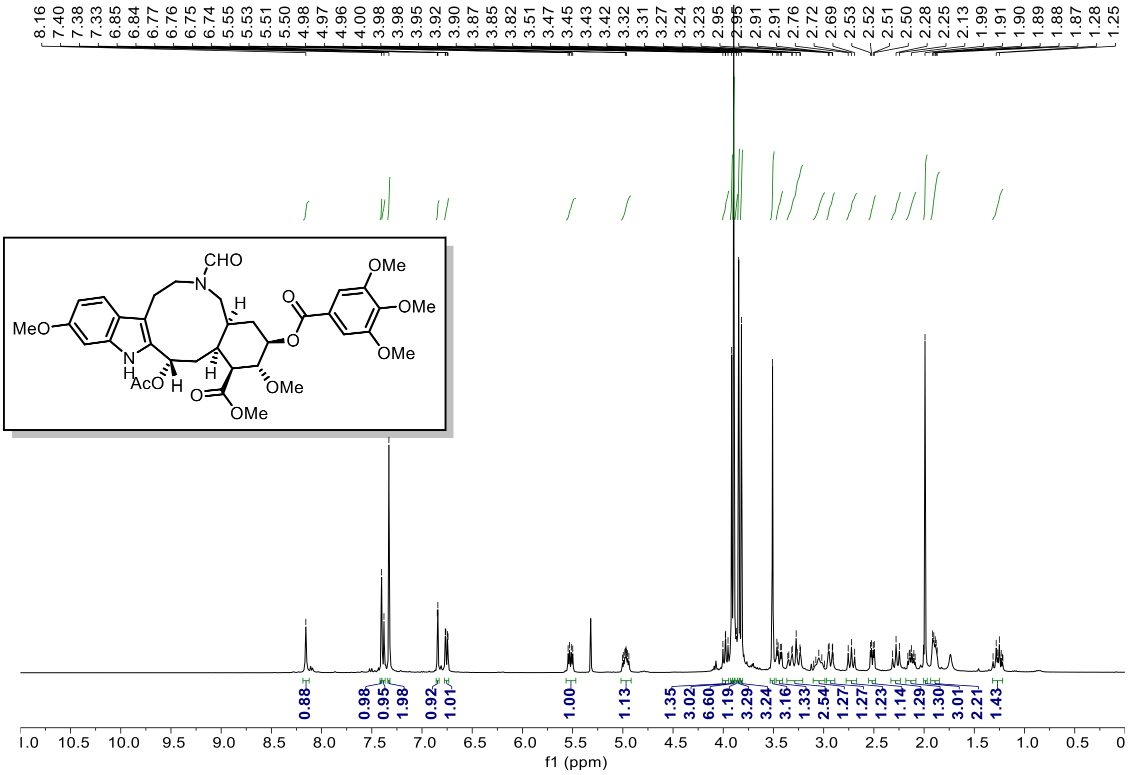

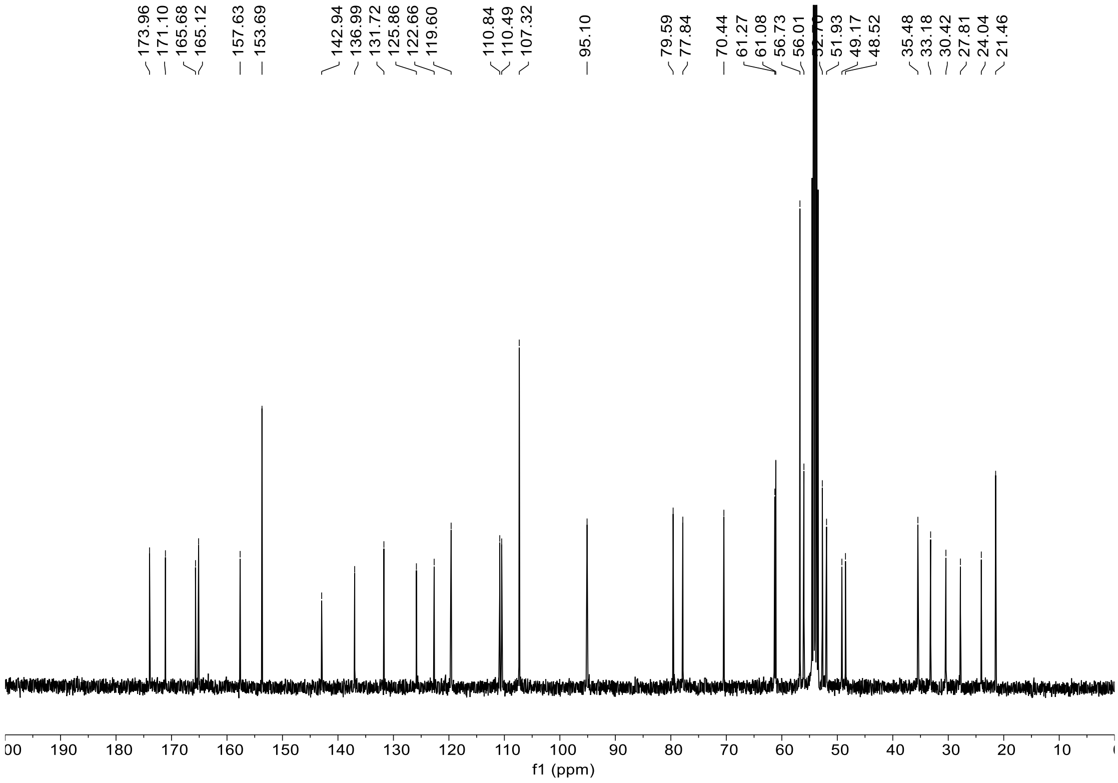

Supplementary Figure 77. ^{1}H and ^{13}C NMR spectra of **12**.

HMBC (400MHz)

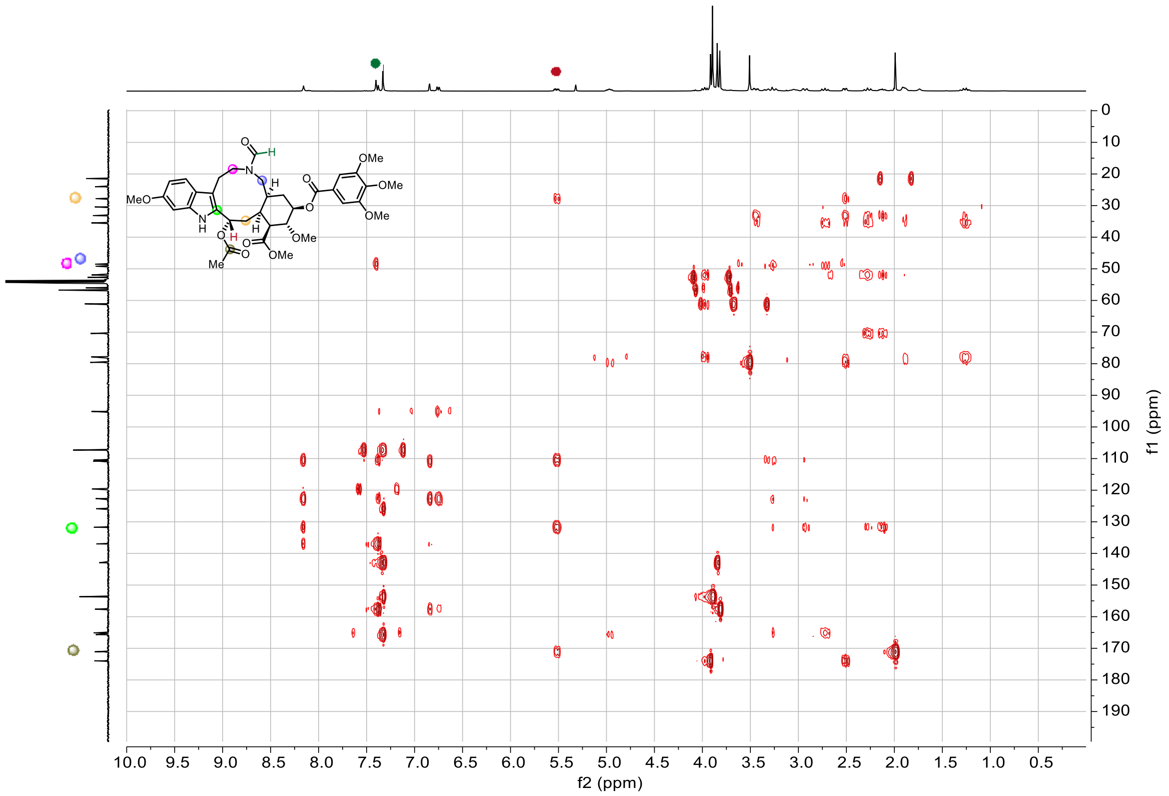

1D NOESY (600MHz)

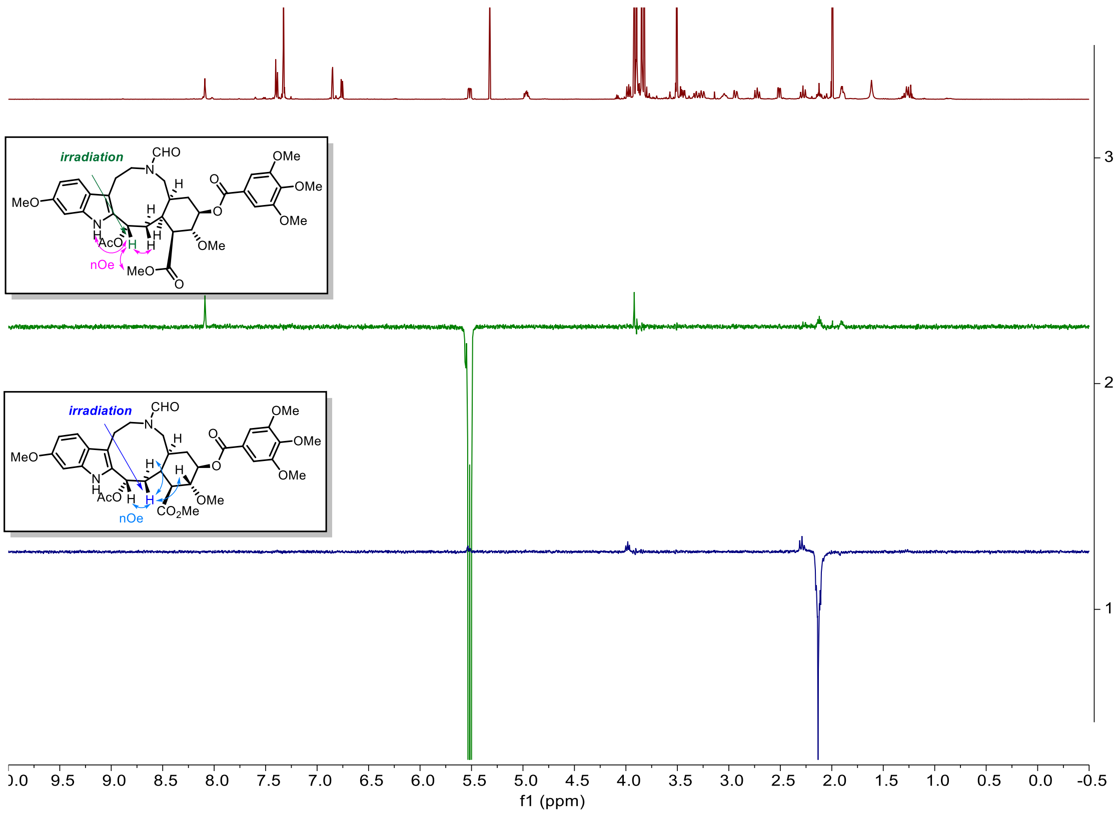

Supplementary Figure 78. HMBC and 1D NOESY of **12**.

N-(5-Bromopentyl)-*N*-(3,3-diphenylpropyl)formamide (13)

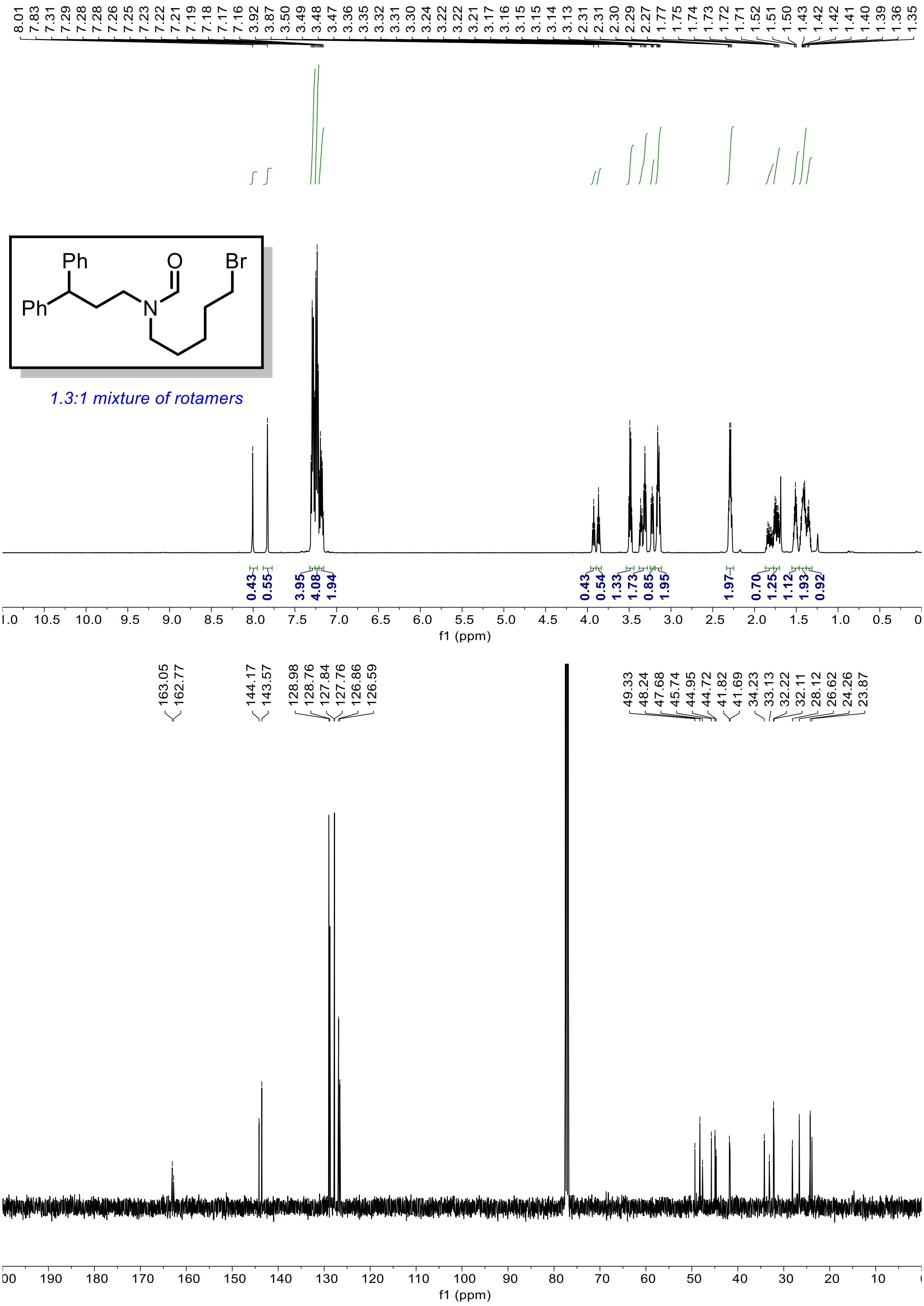

Supplementary Figure 79. ^{1}H and ^{13}C NMR spectra of **13**.

***N*-(6-Bromohexyl)-*N*-(3,3-diphenylpropyl)formamide (14)**

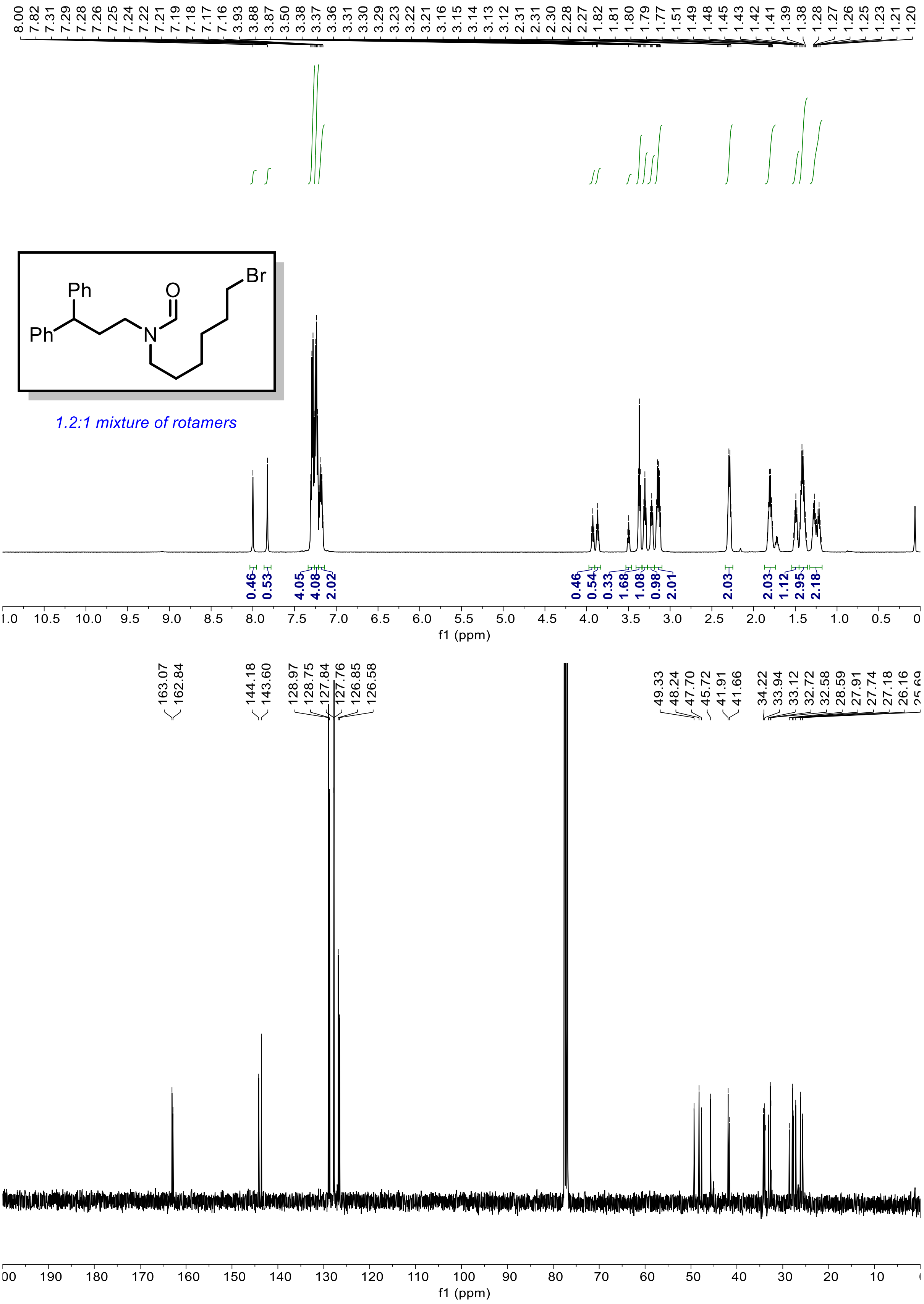

Supplementary Figure 80. ^{1}H and ^{13}C NMR spectra of **14**.

(*S*)-*N*-[2-(2-Bromoethoxy)ethyl]-*N*-(3,7-dimethyloct-6-en-1-yl)formamide (15)

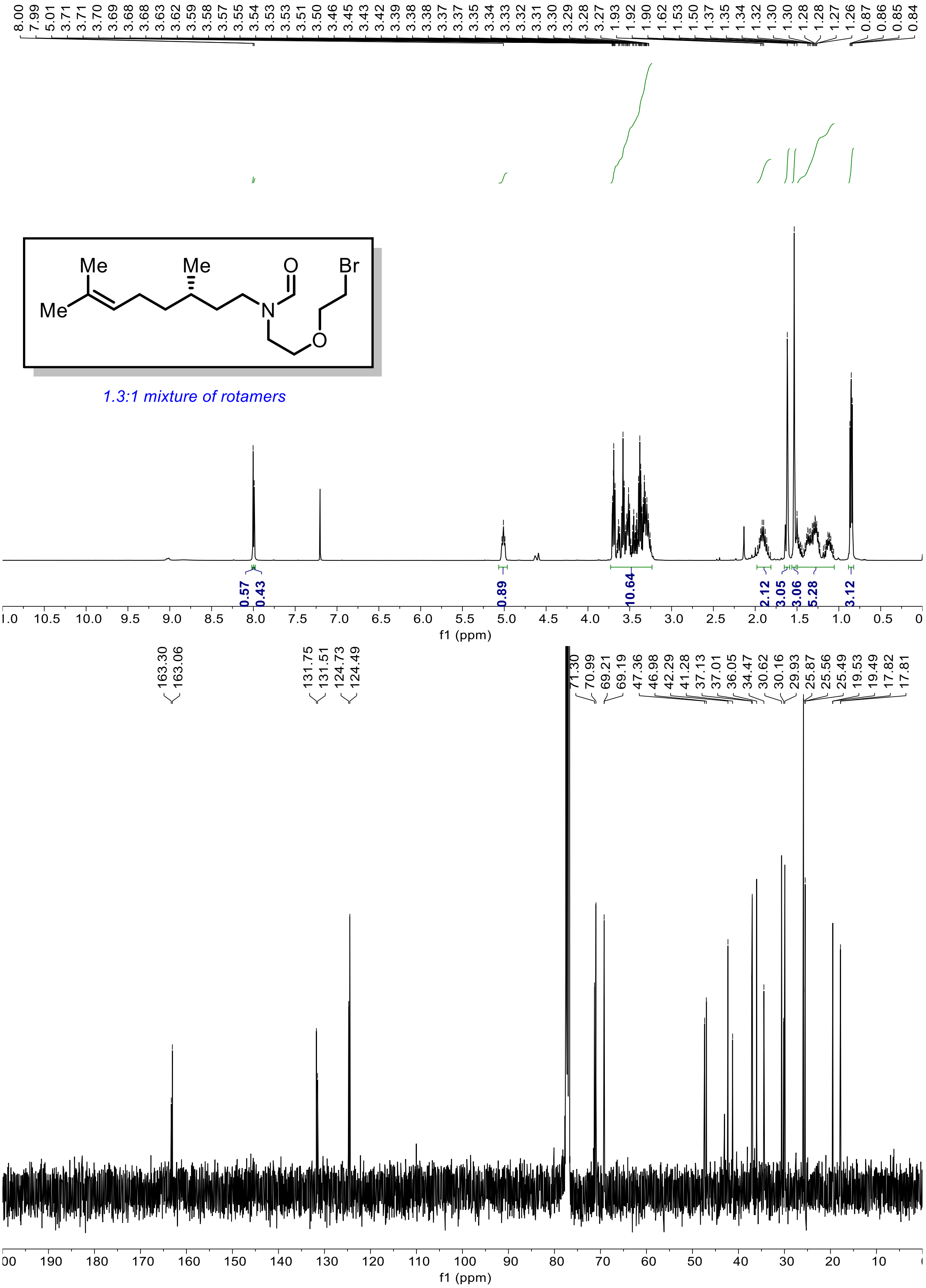

Supplementary Figure 81. ^{1}H and ^{13}C NMR spectra of **15**.

1-(4-Methoxyphenyl)piperidine (16)

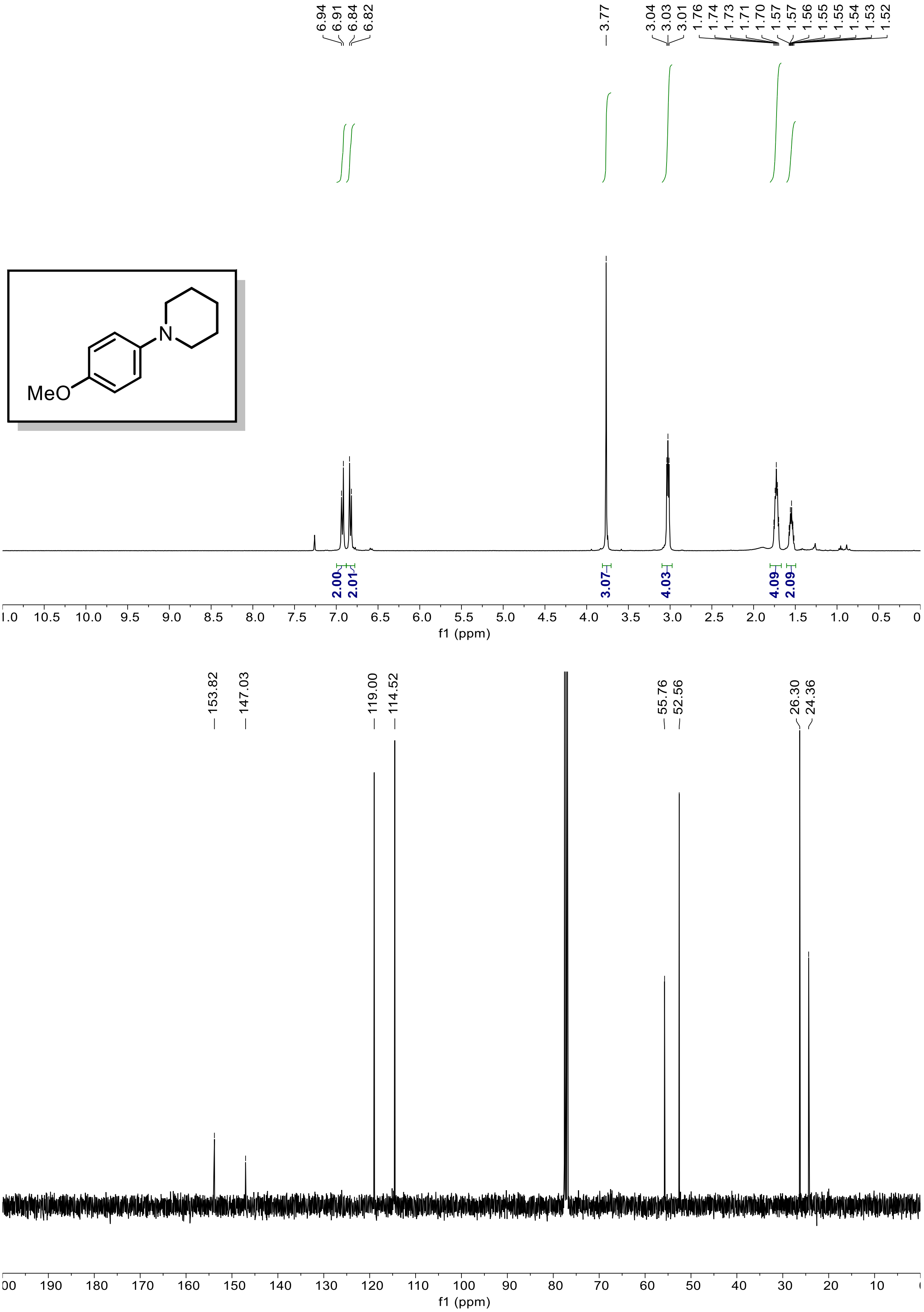

Supplementary Figure 82. ^{1}H and ^{13}C NMR spectra of **16**.

2-(4-Bromobutyl)-6,7-dimethoxy-3,4-dihydroisoquinolin-2-ium chloride

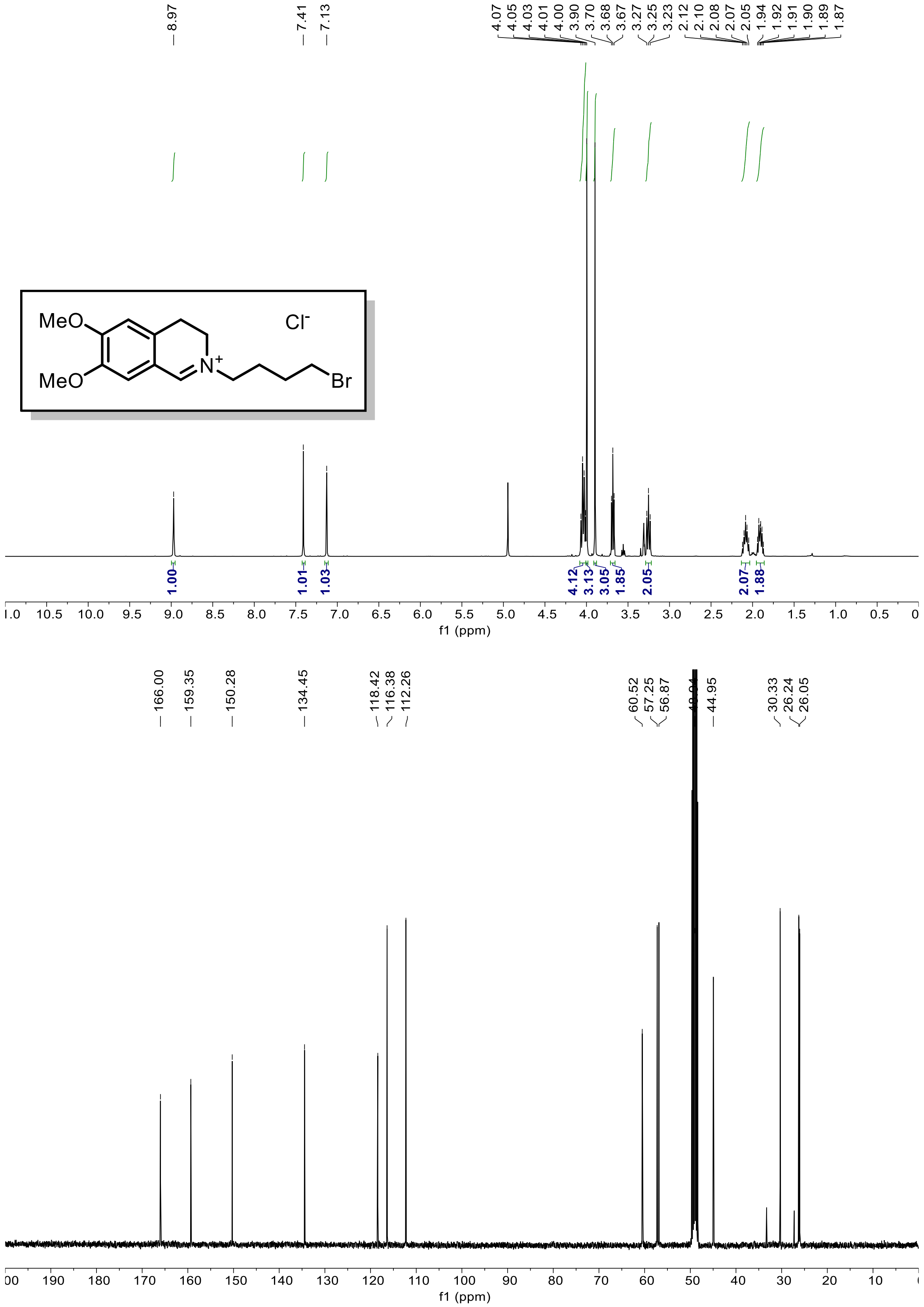

Supplementary Figure 83. ^{1}H and ^{13}C NMR spectra of 2-(4-bromobutyl)-6,7-dimethoxy-3,4-dihydroisoquinolin-2-ium chloride.

6,7-Dimethoxy-3,4-dihydro-1*H*-spiro[isoquinoline-2,1'-pyrrolidin]-2-ium chloride (17)

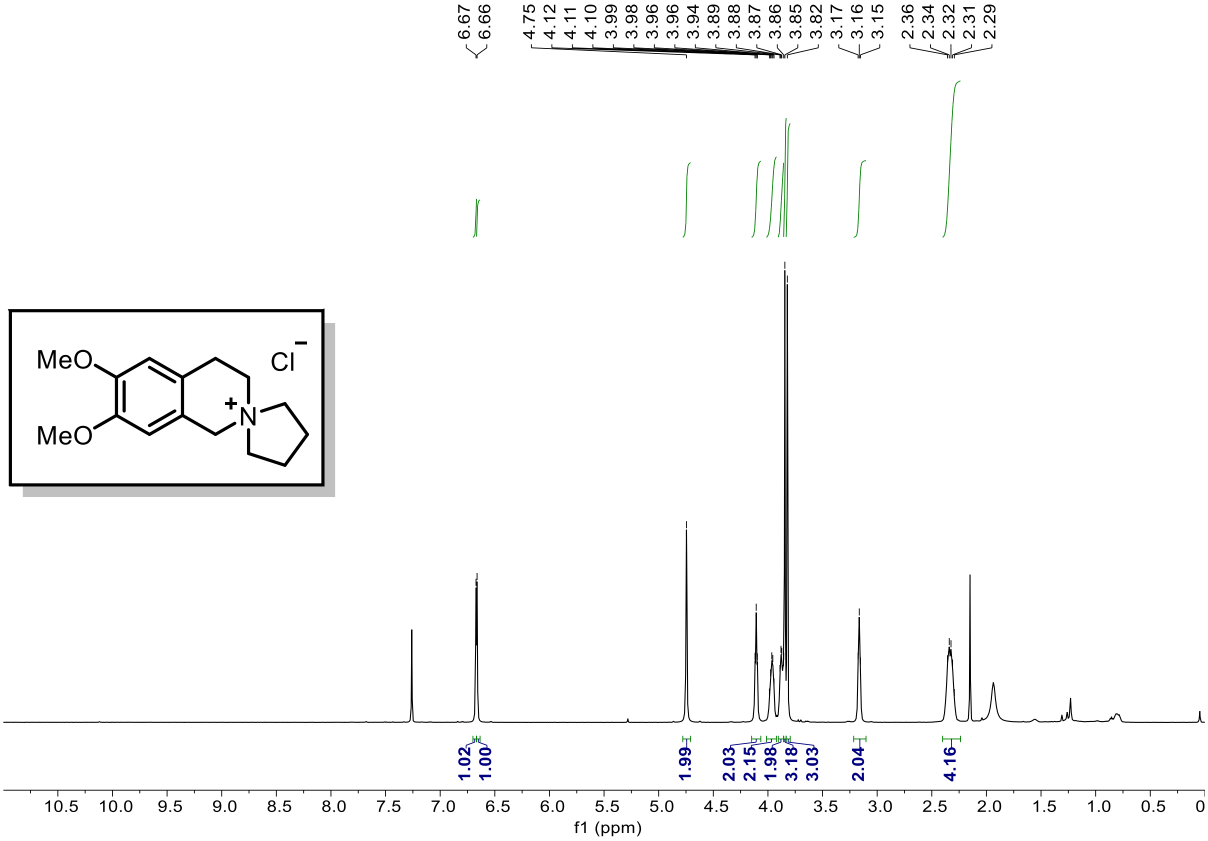

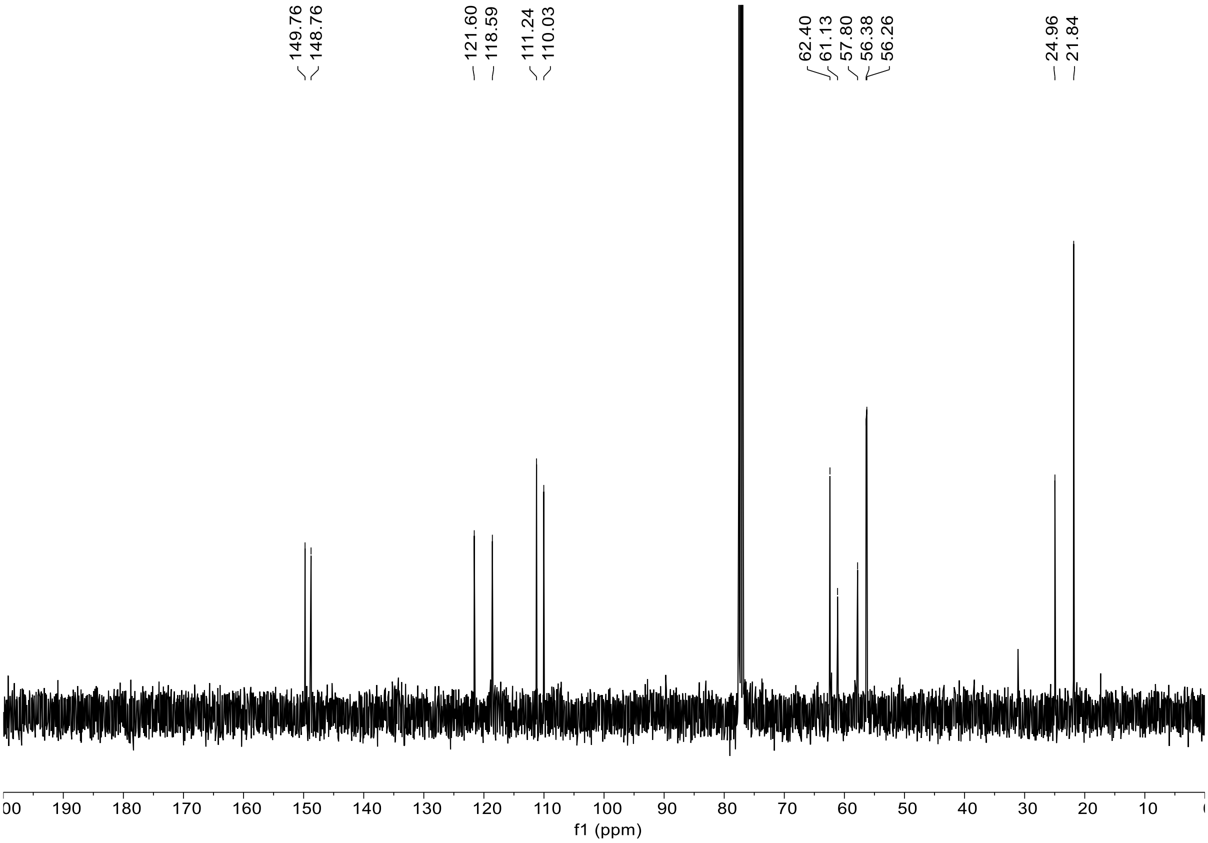

Supplementary Figure 84. ^{1}H and ^{13}C NMR spectra of **17**.

N-(But-3-en-1-yl)-*N*-(4-methoxyphenyl)formamide

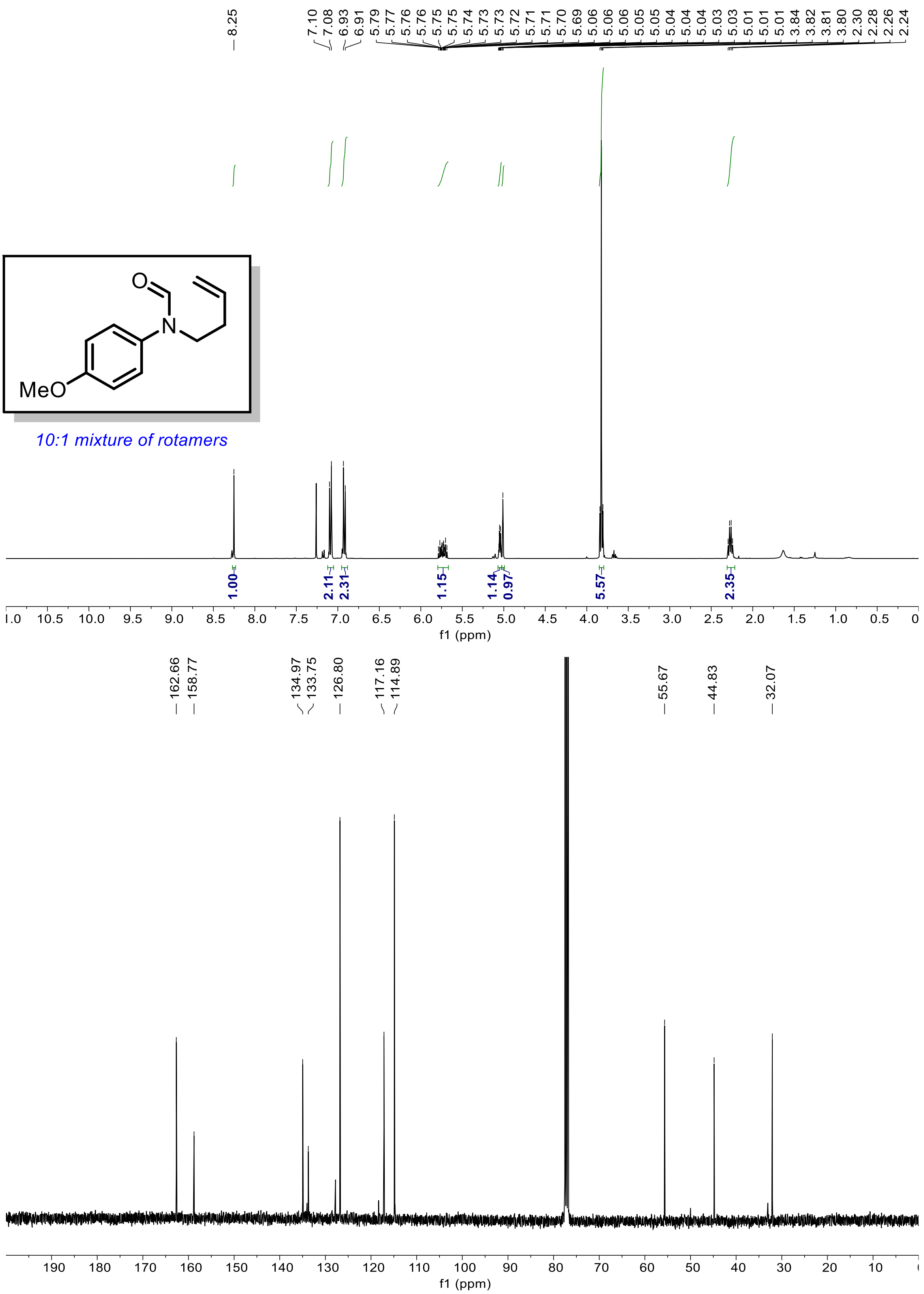

Supplementary Figure 85. ^{1}H and ^{13}C NMR spectra of *N*-(but-3-en-1-yl)-*N*-(4-methoxyphenyl)formamide.

N-(But-3-en-1-yl)-N-(3,4-dimethoxyphenethyl)formamide

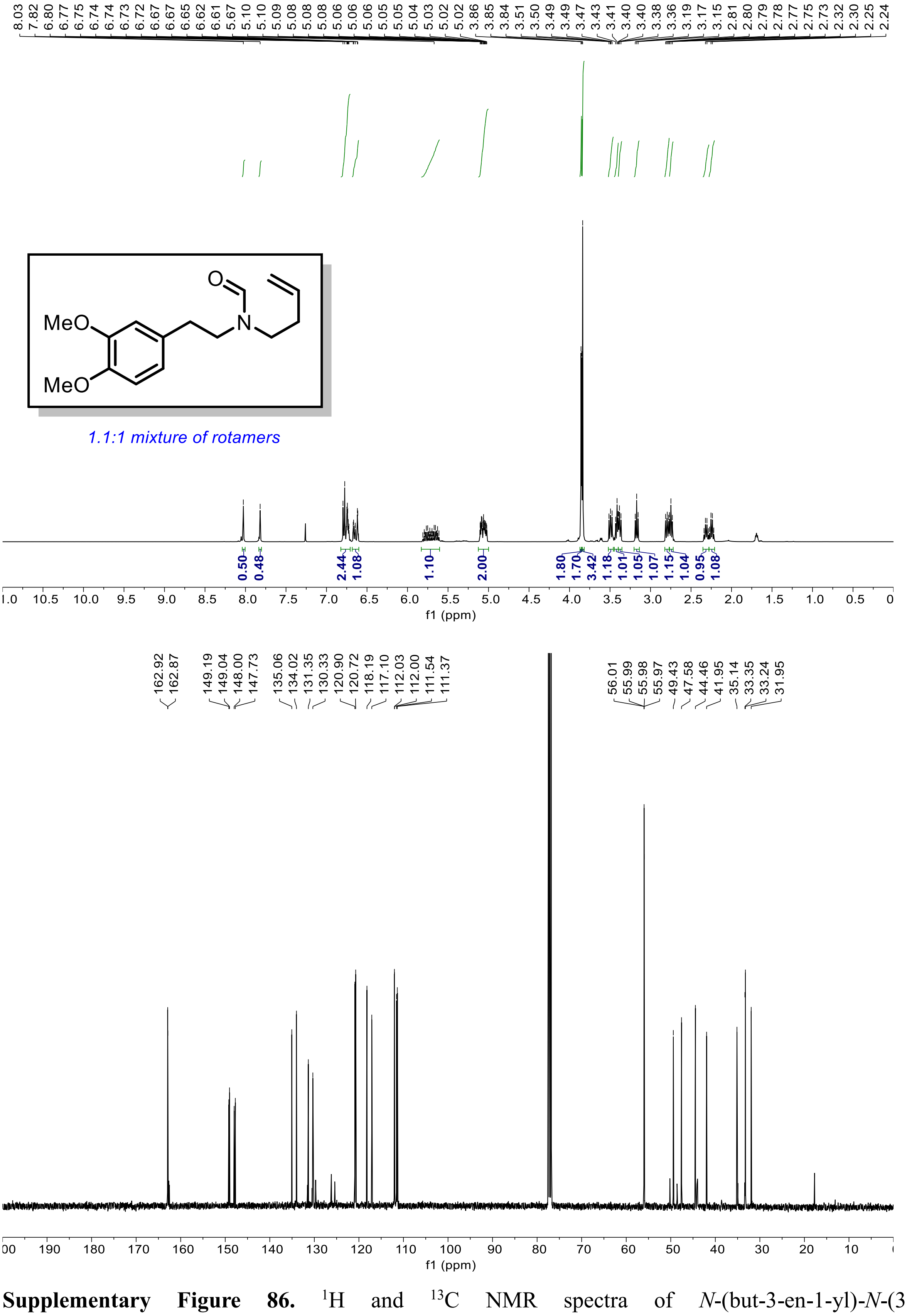

Supplementary Figure 86. ^{1}H and ^{13}C NMR spectra of *N*-(but-3-en-1-yl)-*N*-(3,4-dimethoxyphenethyl)formamide.

1-(4-Methoxyphenyl)-3-methylpyrrolidin-2-one (18)

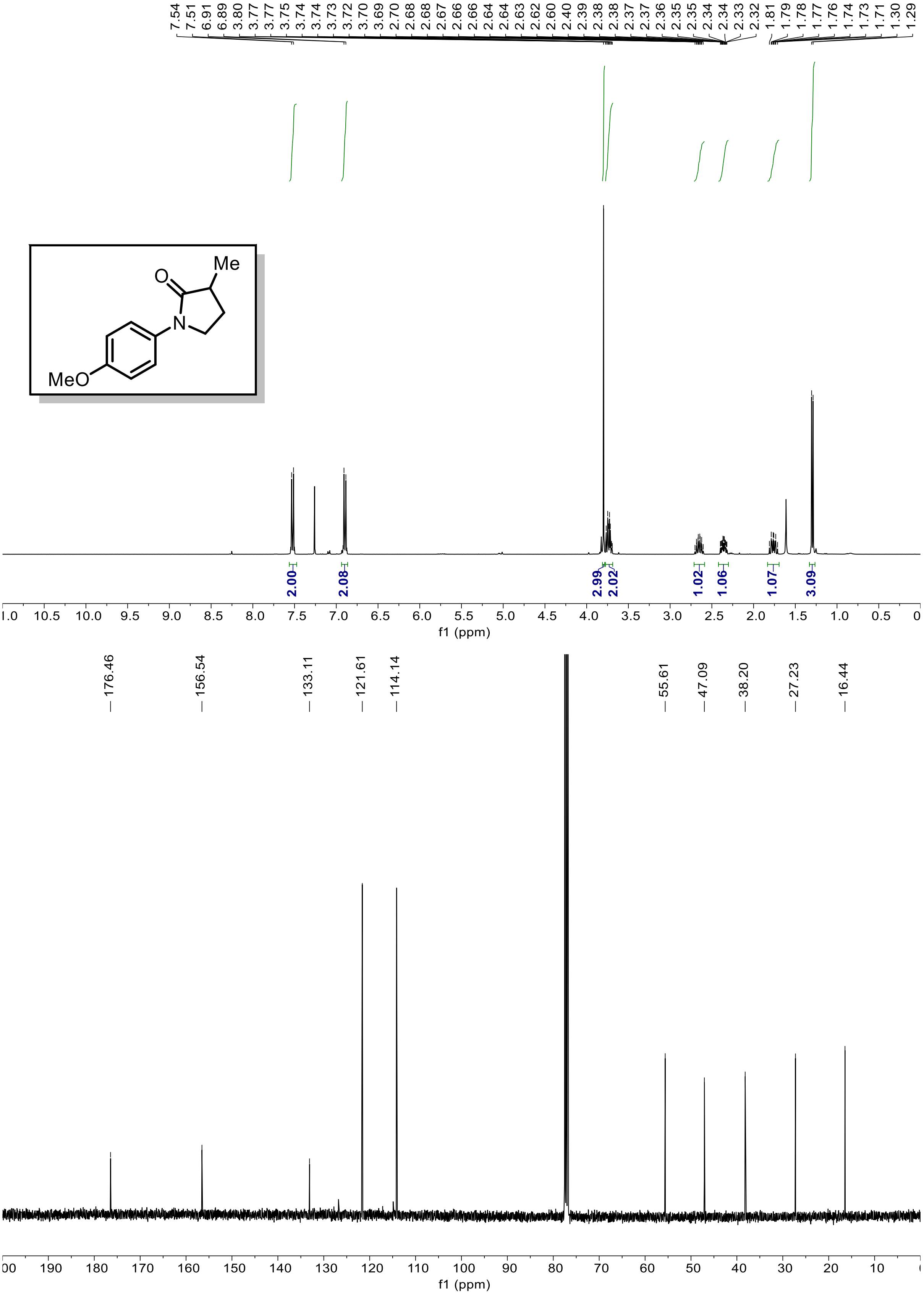

Supplementary Figure 87. ^{1}H and ^{13}C NMR spectra of **18**.

1-(3,4-Dimethoxyphenethyl)-3-methylpyrrolidin-2-one (19)

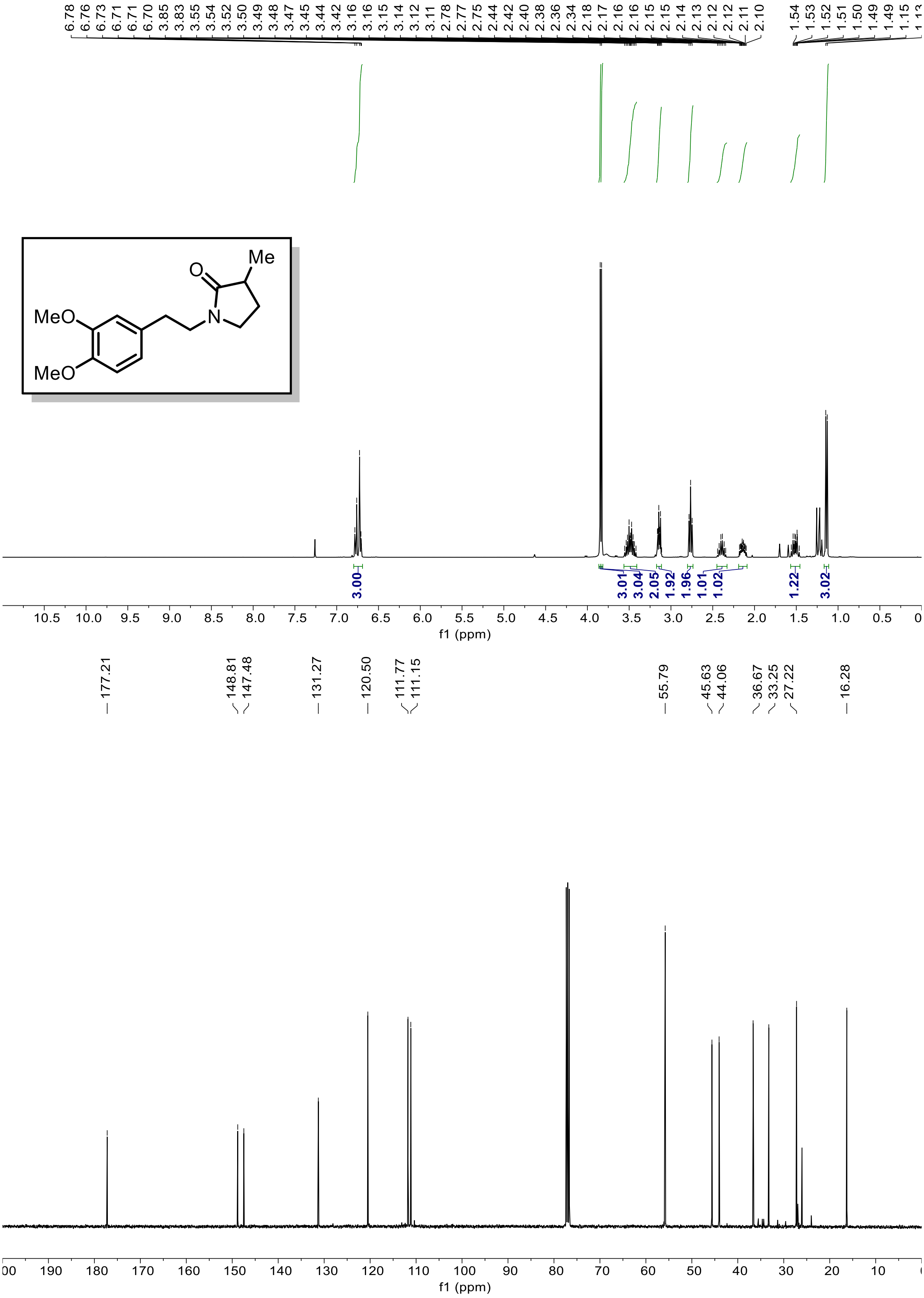

Supplementary Figure 88. ^{1}H and ^{13}C NMR spectra of **19**.

2-(4-Methoxyphenyl)-2-azabicyclo[3.1.0]hexane (20)

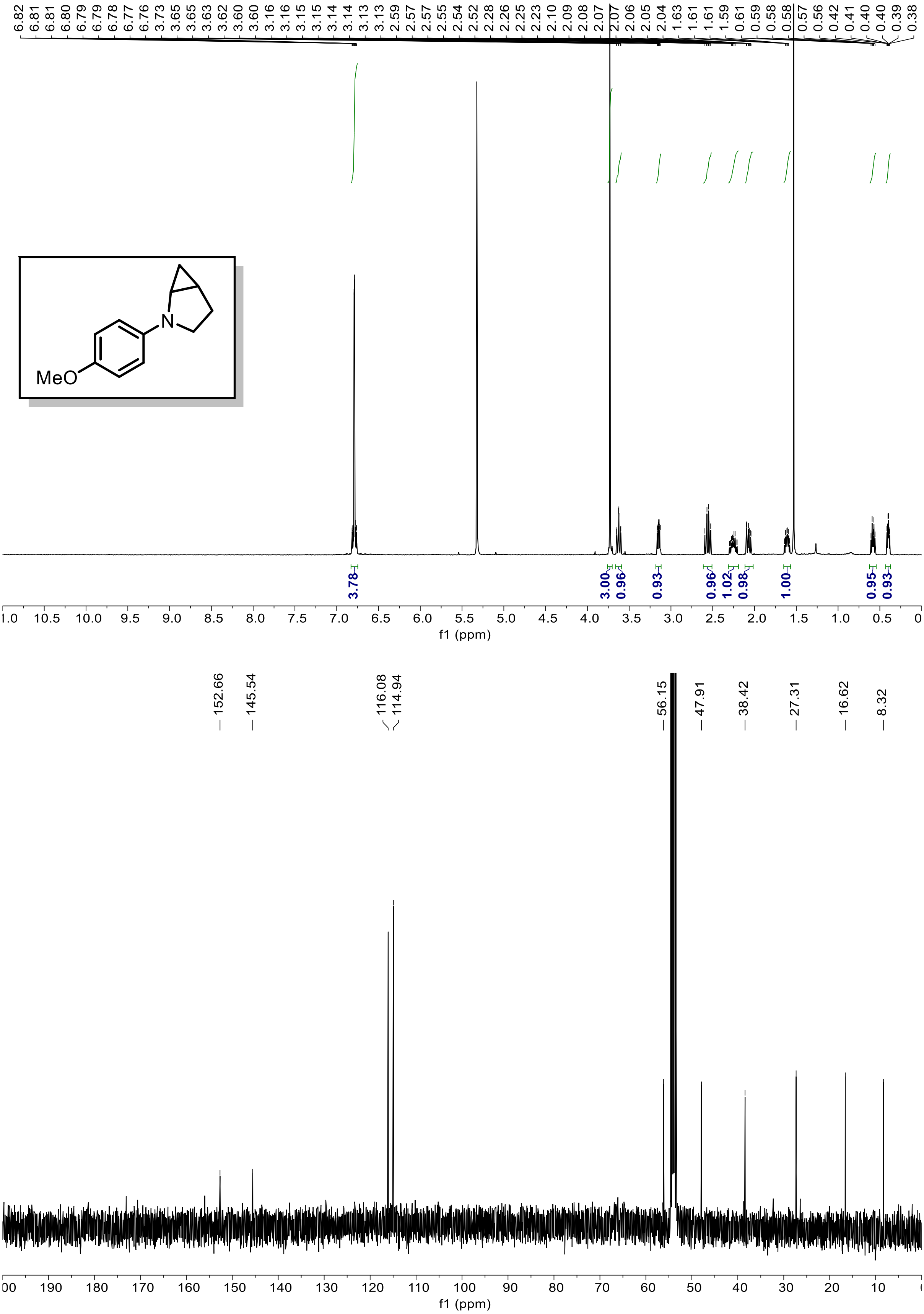

Supplementary Figure 89. ^{1}H and ^{13}C NMR spectra of **20**.

2-(3,4-Dimethoxyphenethyl)-2-azabicyclo[3.1.0]hexane (21)

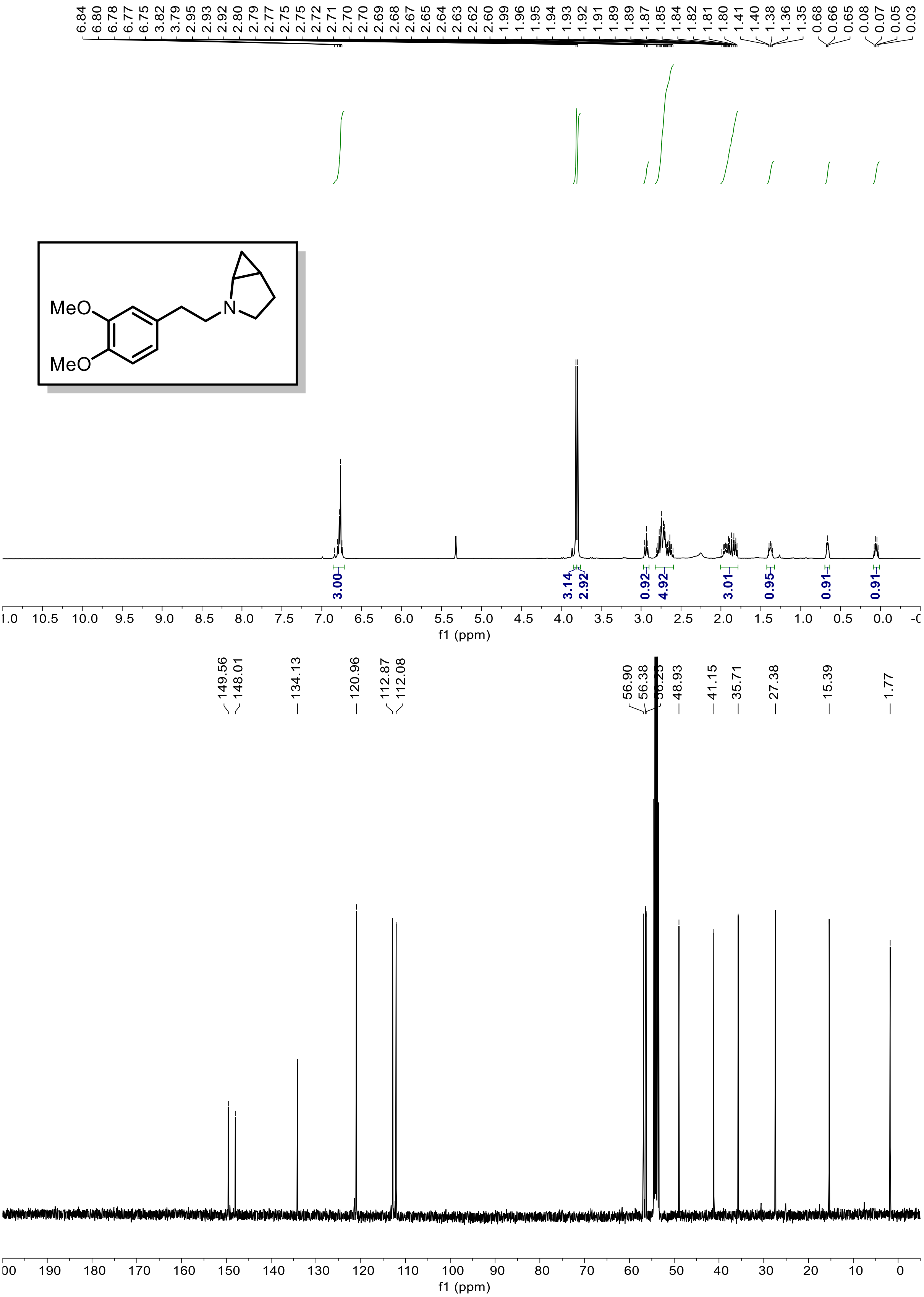

Supplementary Figure 90. ^{1}H and ^{13}C NMR spectra of **21**.

N-(4-Methoxyphenyl)-*N*-[2-(2-oxotetrahydrofuran-3-yl)ethyl]formamide (22)

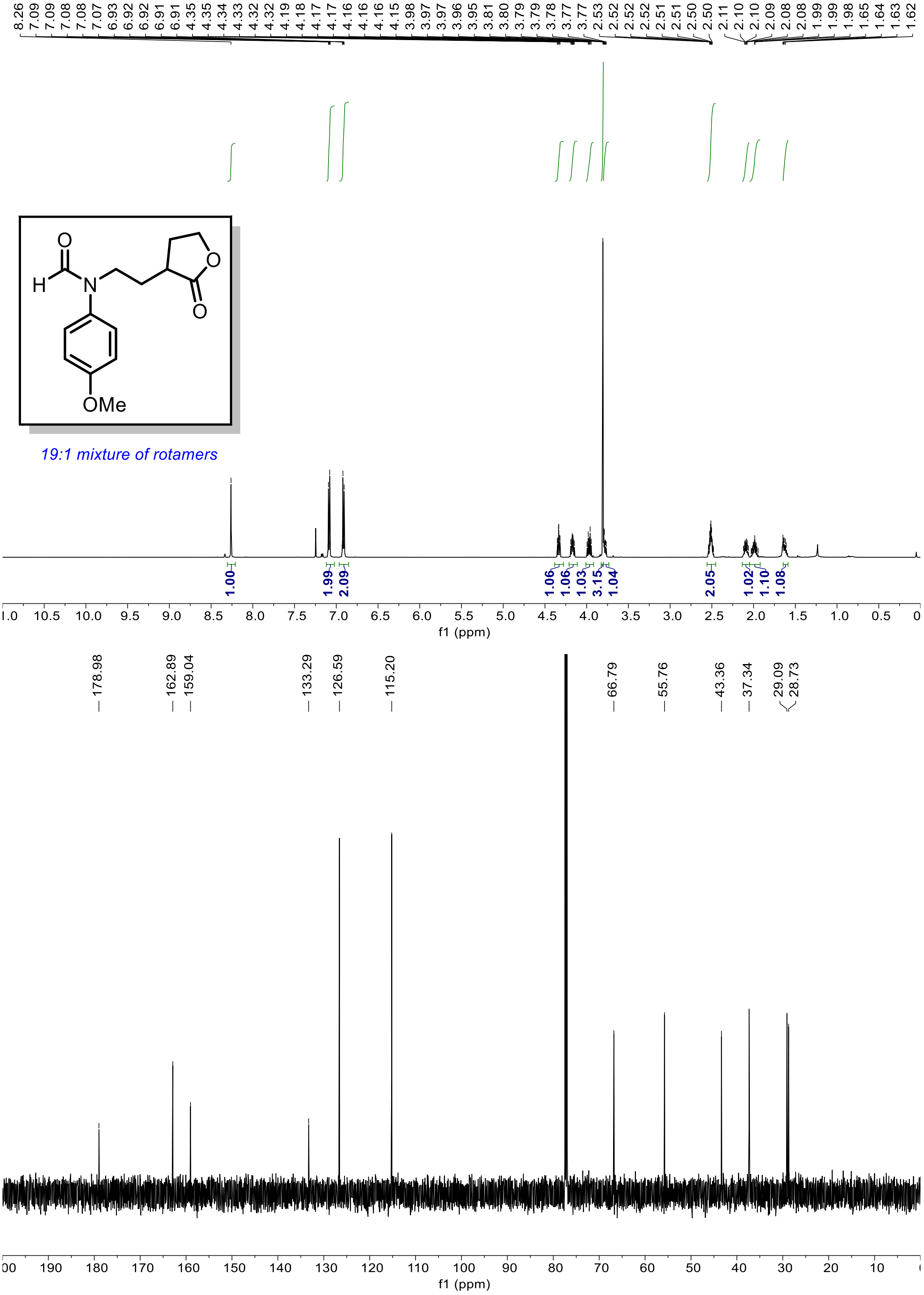

Supplementary Figure 91. ^{1}H and ^{13}C NMR spectra of **22**.

N-[2-(2-Oxooxazolidin-3-yl)ethyl]-*N*-phenylformamide (23)

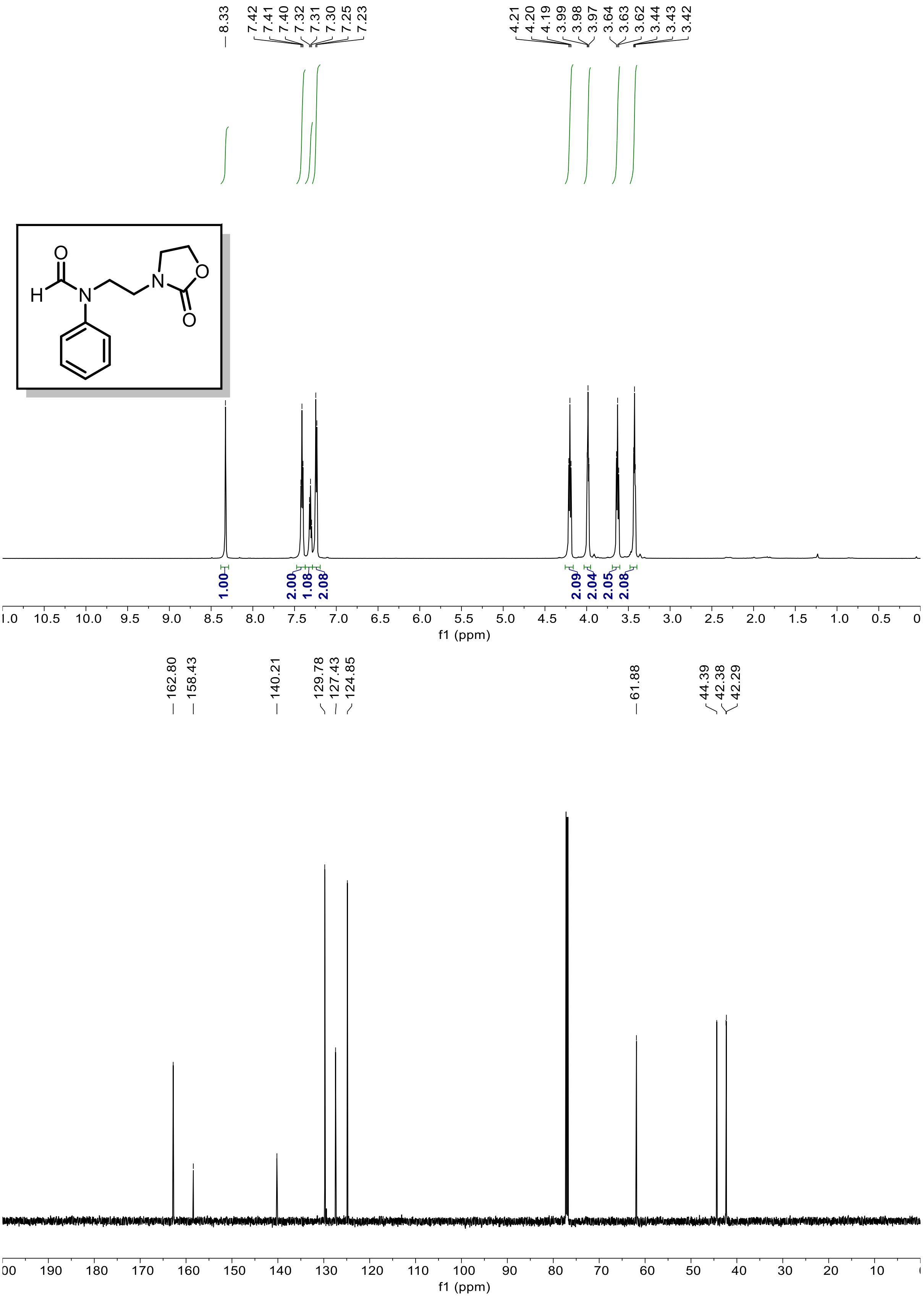

Supplementary Figure 92. ^{1}H and ^{13}C NMR spectra of **23**.

(*E*)-*N*-[2-(3,4-Dimethoxystyryl)-4,5-dimethoxyphenethyl]-*N*-methylformamide (24)

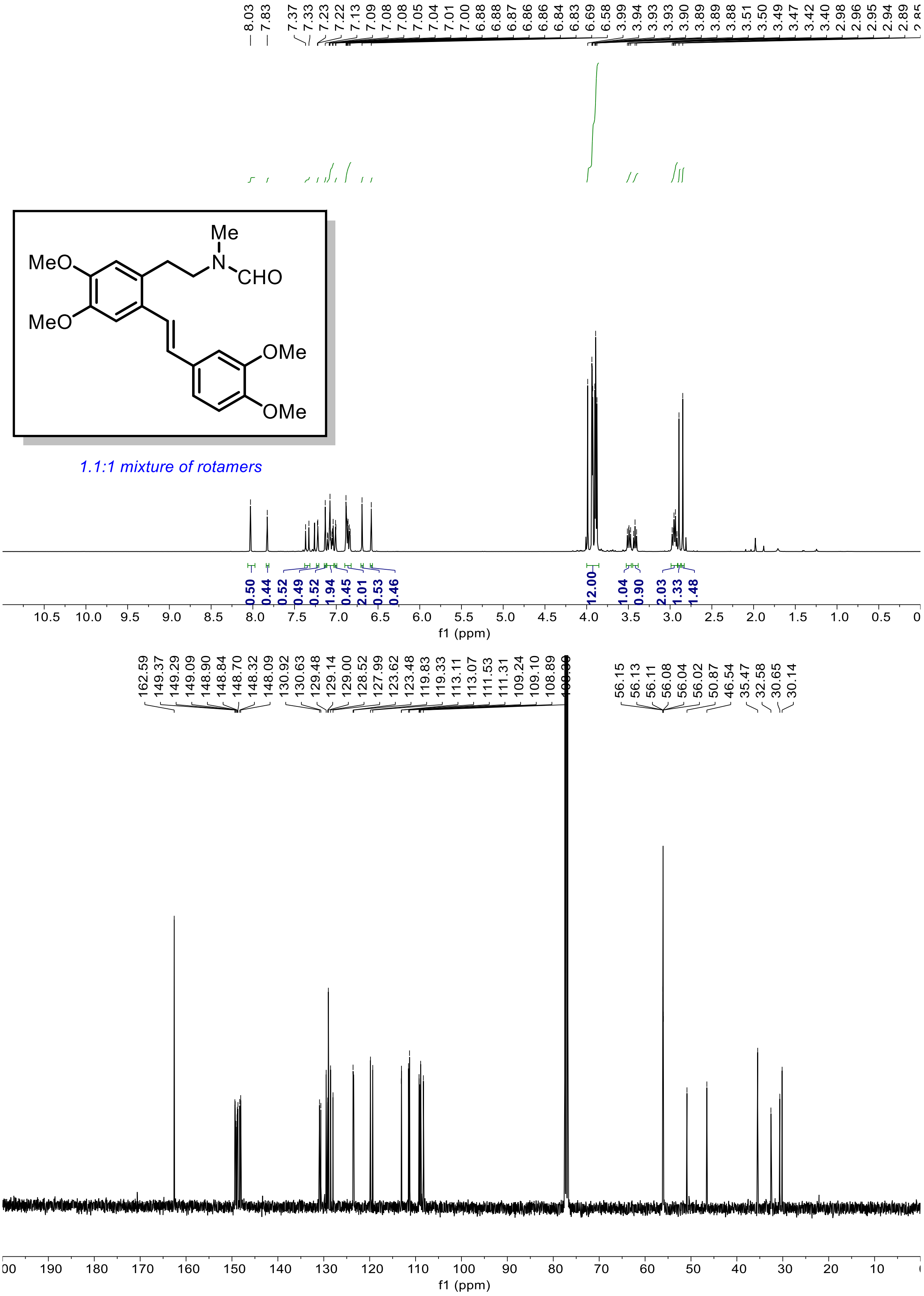

Supplementary Figure 93. ^{1}H and ^{13}C NMR spectra of **24**.

(*E*)-1-(3,4-Dimethoxybenzylidene)-7,8-dimethoxy-3-methyl-2,3,4,5-tetrahydro-1H-benzo[*d*]-azepine (25)

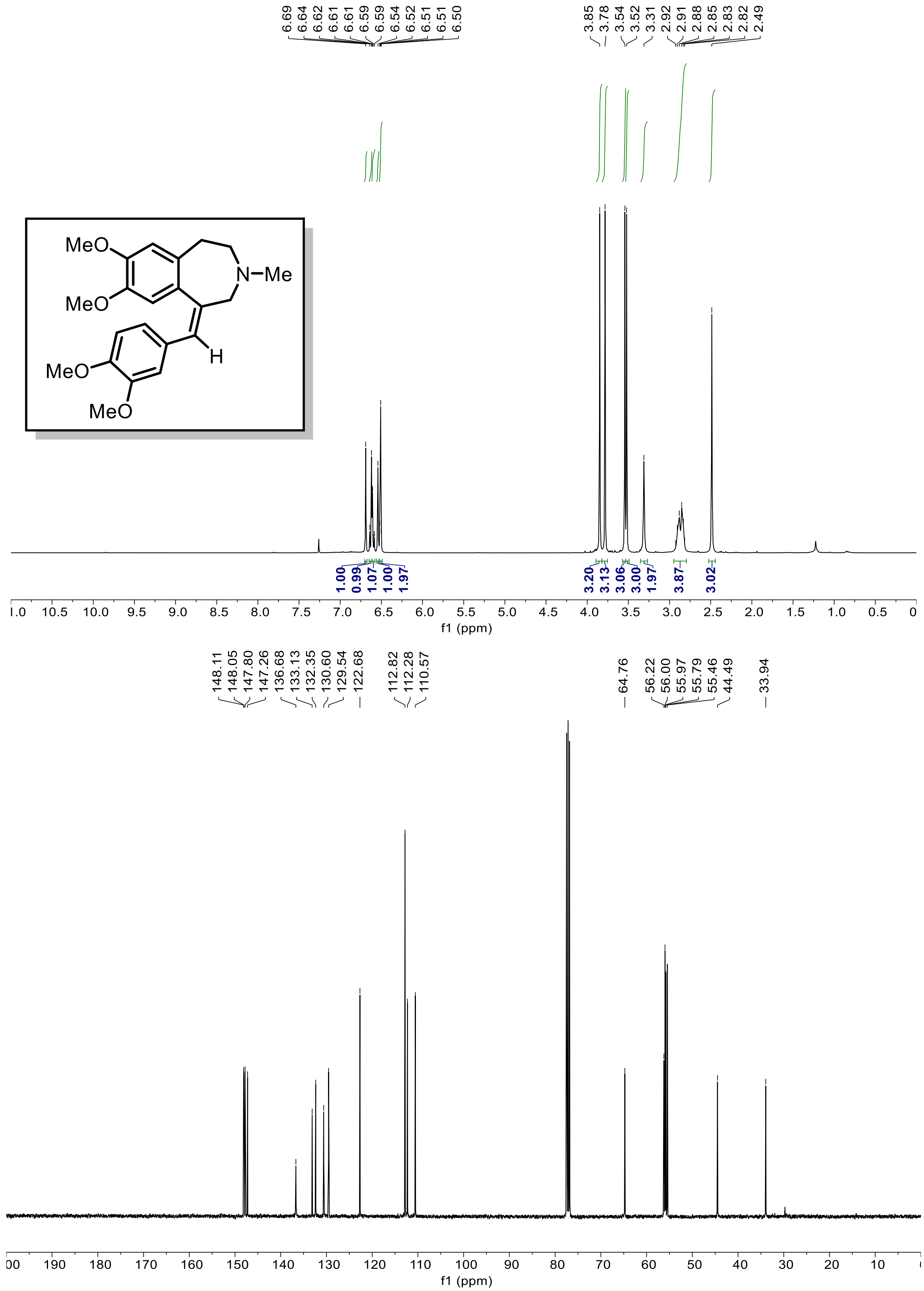

Supplementary Figure 94. ^{1}H and ^{13}C NMR spectra of **25**.

NOESY (400MHz)

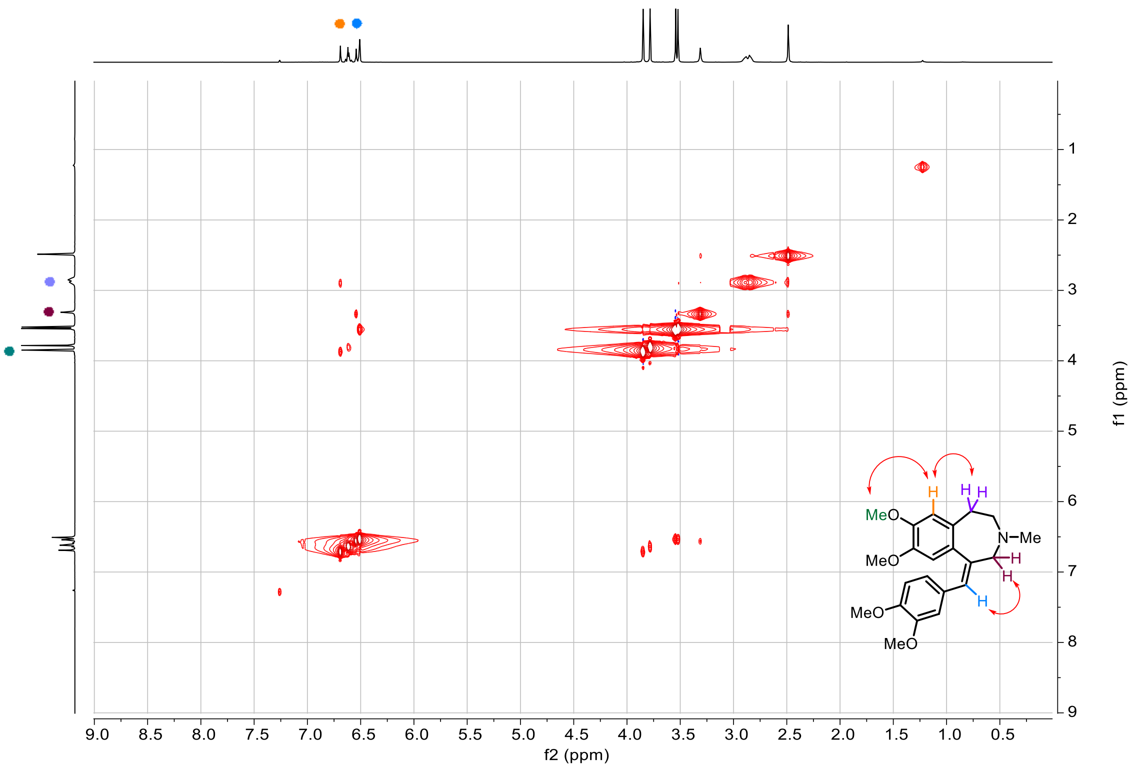

HMBC (400MHz)

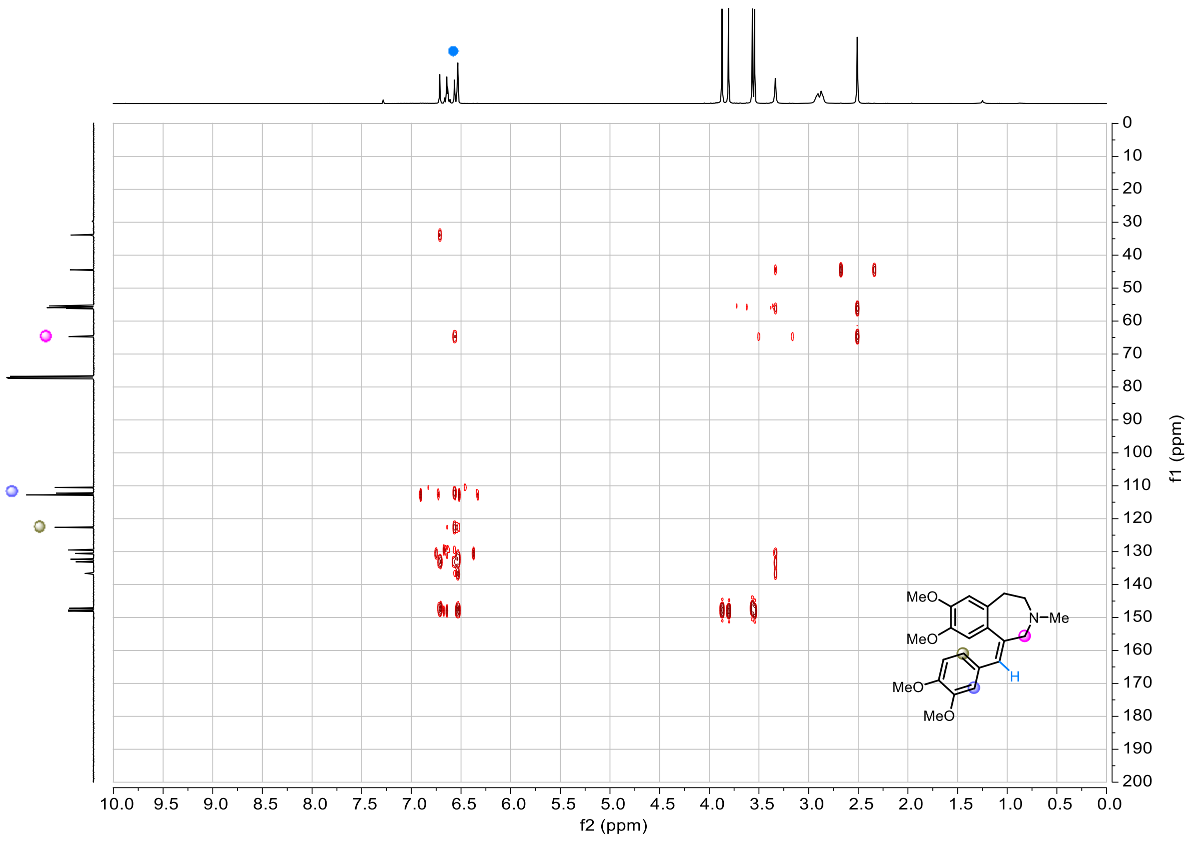

Supplementary Figure 95. NOESY and HMBC of **25**.

(*Z*)-1-(3,4-Dimethoxybenzylidene)-7,8-dimethoxy-3-methyl-2,3,4,5-tetrahydro-1H-benzo[*d*]-azepine (25’)

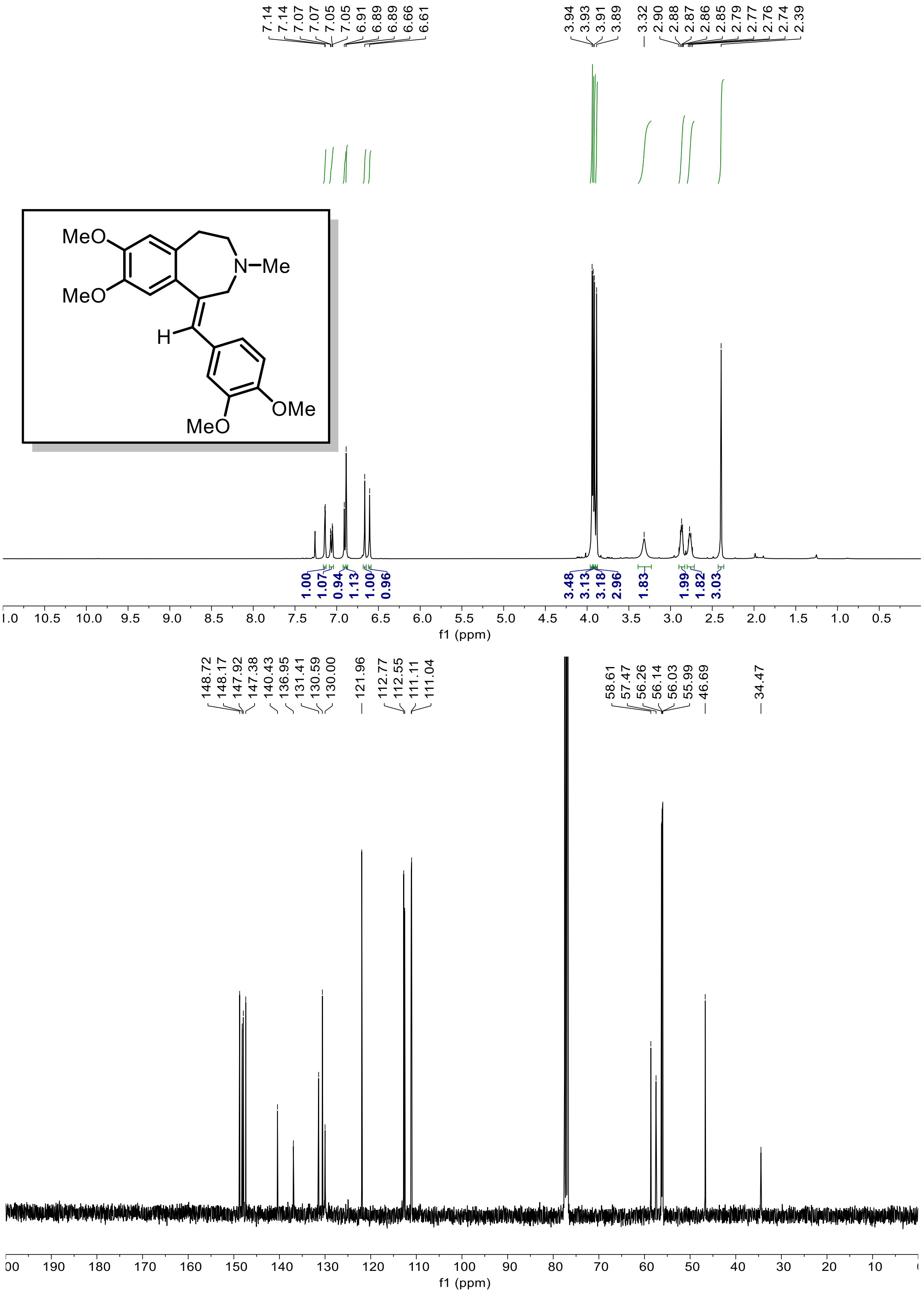

Supplementary Figure 96. ^{1}H and ^{13}C NMR spectra of **25’**.

NOESY (400MHz)

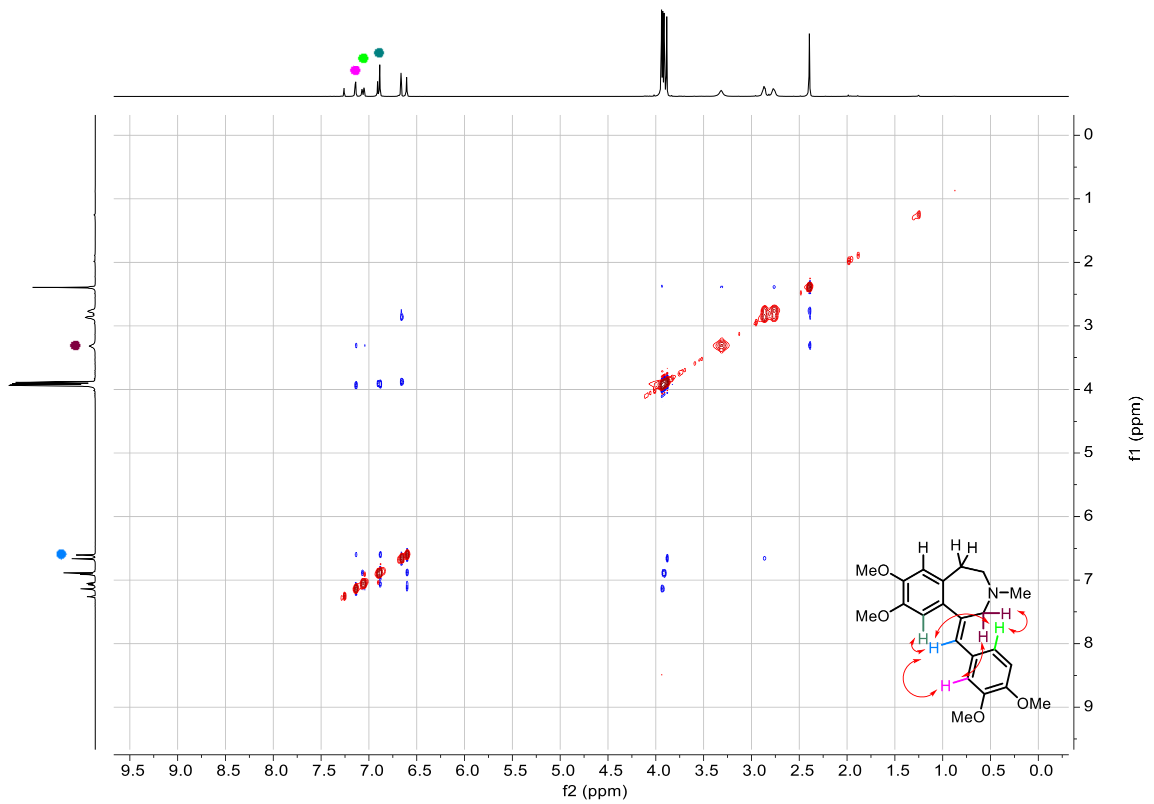

HMBC (400MHz)

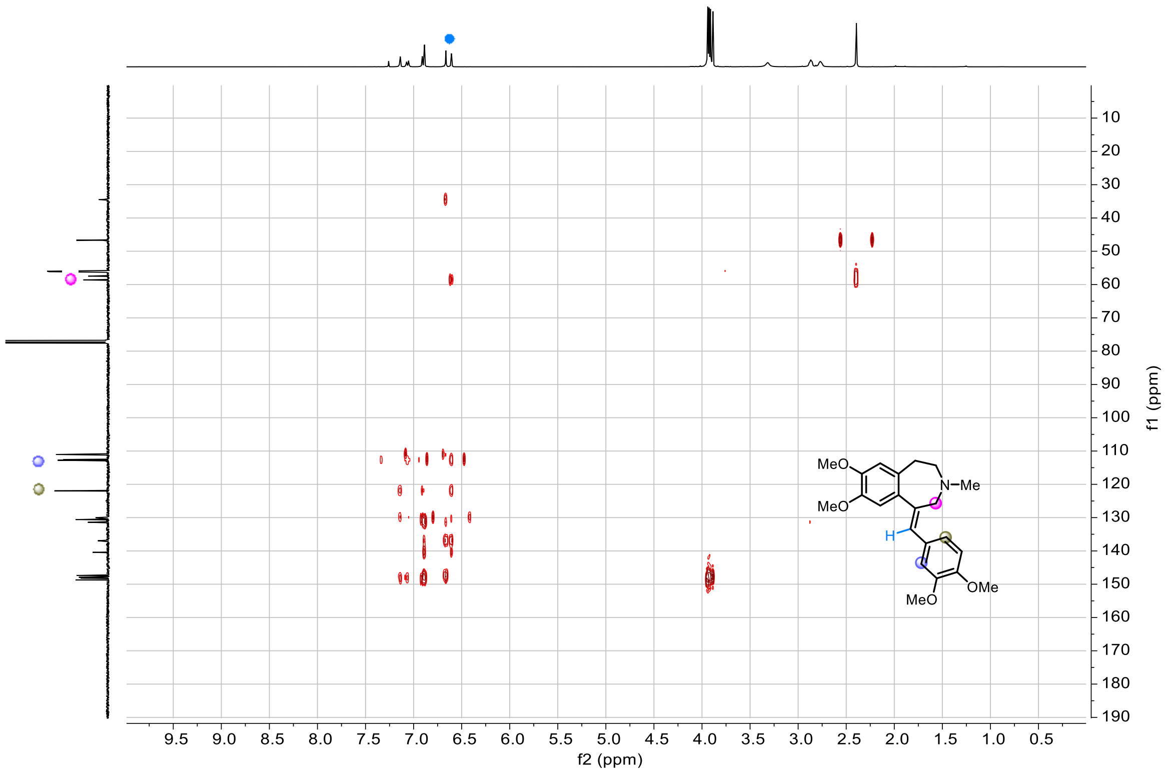

Supplementary Figure 97. NOESY and HMBC of **25'**.

X-ray Crystallographic Data

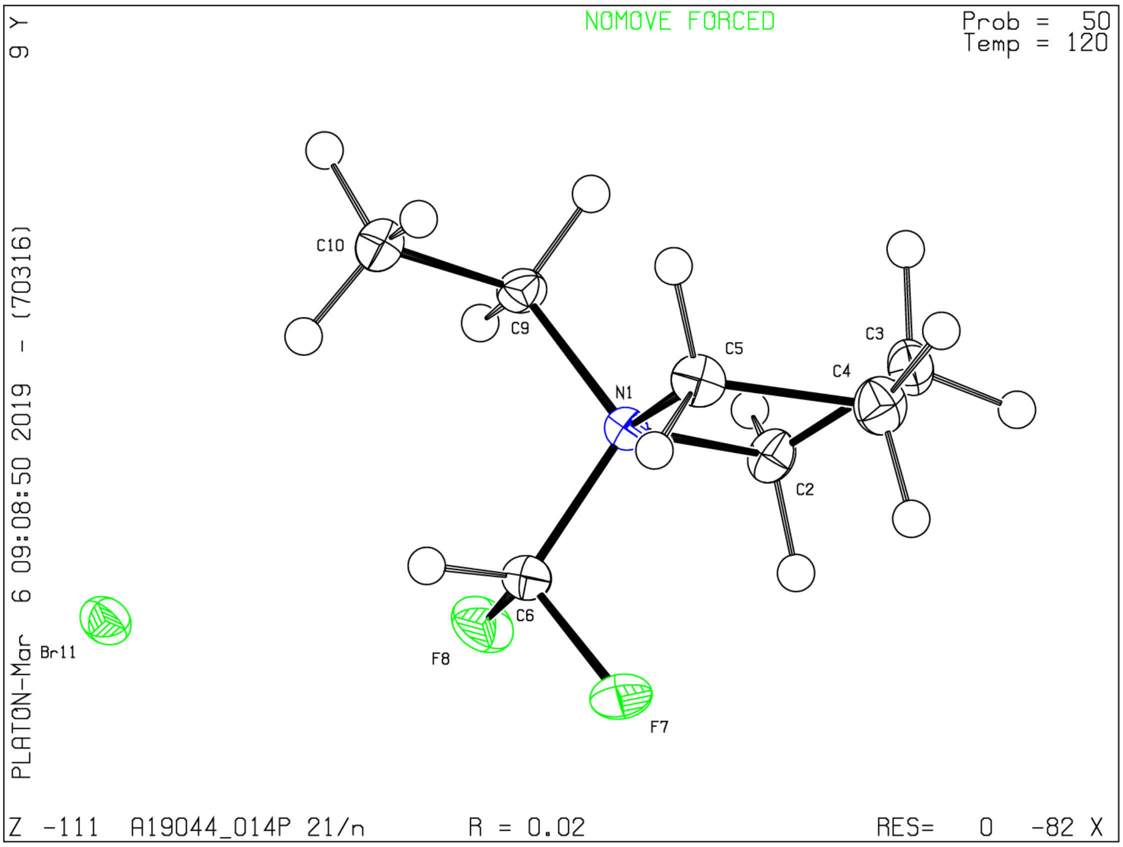

Supplementary Figure 98. Crystallographic data of **int1**.

Crystal data and structure refinement for **int1** (CCDC 1977901).

Empirical formula	$C_7H_{14}NF_2Br$	
Formula weight	230.10	
Temperature	120(2) K	
Wavelength	0.71073 Å	
Crystal system	Monoclinic	
Space group	$P2_1/n$	
Unit cell dimensions	a = 7.7404(4) Å	α = 90°
	b = 8.9207(5) Å	β = 93.980(3)°
	c = 13.4966(8) Å	γ = 90°
Volume	929.69(9) $Å^3$	
Z	4	
Density (calculated)	1.644 Mg/m^3	
Absorption coefficient	4.394 mm^{-1}	
F(000)	464	
Crystal size	0.323 x 0.281 x 0.087 mm^3	
Theta range for data collection	2.739 to 26.994°.	
Index ranges	-9<=h<=9, -11<=k<=11, -17<=l<=17	
Reflections collected	14550	
Independent reflections	2029 [R(int) = 0.0517]	
Completeness to theta = 25.242°	100.0 %	
Absorption correction	Semi-empirical from equivalents	
Max. and min. transmission	0.7458 and 0.5359	
Refinement method	Full-matrix least-squares on F^2	
Data / restraints / parameters	2029 / 0 / 104	
Goodness-of-fit on F^2	1.037	
Final R indices [I>2sigma(I)]	R1 = 0.0240, wR2 = 0.0518	
R indices (all data)	R1 = 0.0337, wR2 = 0.0552	
Largest diff. peak and hole	0.319 and -0.339 e.$Å^{-3}$	

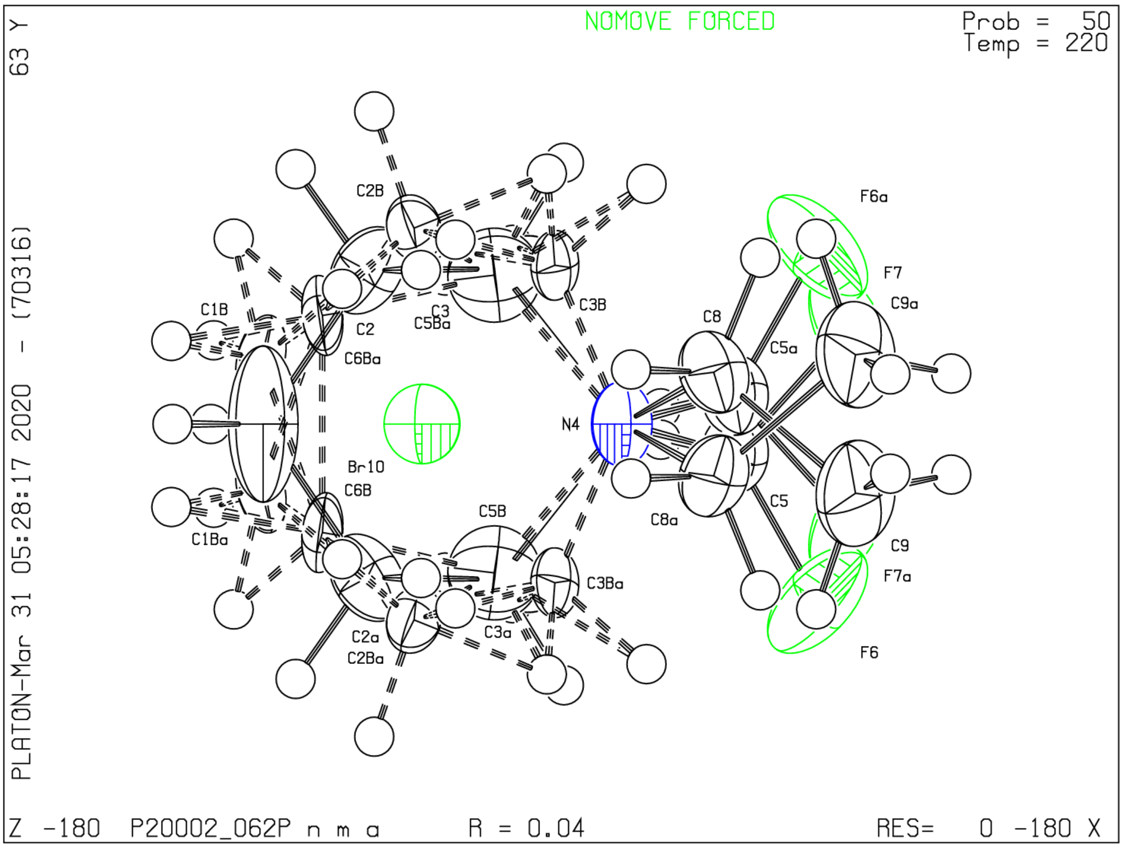

Supplementary Figure 99. Crystallographic data of **int2**.

Crystal data and structure refinement for **int2** (CCDC 2000476).

Empirical formula	$C_8H_{16}NF_2Br$	
Formula weight	244.13	
Temperature	220(2) K	
Wavelength	0.630 Å	
Crystal system	Orthorhombic	
Space group	*Pnma*	
Unit cell dimensions	a = 14.461(3) Å	α = 90°
	b = 8.8210(18) Å	β = 90°
	c = 8.3710(17) Å	γ = 90°
Volume	1067.8(4) $Å^3$	
Z	4	
Density (calculated)	1.519 Mg/m^3	
Absorption coefficient	2.796 mm^{-1}	
F(000)	496	
Crystal size	0.075 x 0.069 x 0.057 mm^3	
Theta range for data collection	2.492 to 25.997°.	
Index ranges	–20<=h<=20, –12<=k<=12, –11<=l<=11	
Reflections collected	10569	
Independent reflections	1598 [R(int) = 0.0679]	
Completeness to theta = 22.210°	99.7 %	
Absorption correction	Empirical	
Max. and min. transmission	1.000 and 0.916	
Refinement method	Full-matrix least-squares on F^2	
Data / restraints / parameters	1598 / 6 / 131	
Goodness-of-fit on F^2	1.140	
Final R indices [I>2sigma(I)]	R1 = 0.0362, wR2 = 0.1168	
R indices (all data)	R1 = 0.0410, wR2 = 0.1204	
Largest diff. peak and hole	1.320 and –0.754 $e·Å^{-3}$	

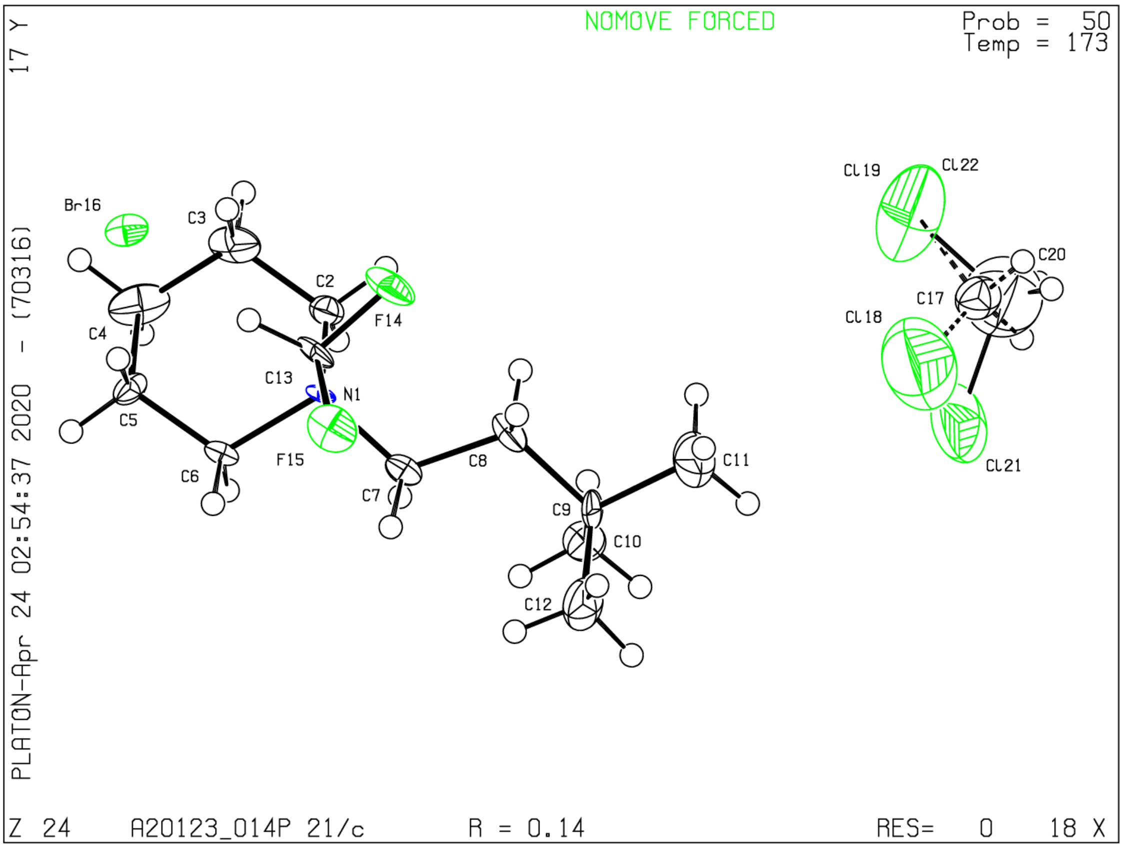

Supplementary Figure 100. Crystallographic data of **int3**.

Response to PLAT341_Alert_3_B in checkCIF report: The weak diffraction based on small size and asymmetric shape of crystal caused low bond precision. This is the best data obtained after our much efforts, for example by directly coating the crystal and quickly mounting it on diffractometer.

Crystal data and structure refinement for **int3** (CCDC 2000477).

Empirical formula	$C_{13}H_{26}F_2NCl_2Br$	
Formula weight	385.16	
Temperature	173(2) K	
Wavelength	0.71073 Å	
Crystal system	Monoclinic	
Space group	$P2_1/c$	
Unit cell dimensions	a = 12.0747(12) Å	α = 90°
	b = 18.6335(17) Å	β = 101.312(4)°
	c = 8.2072(8) Å	γ = 90°
Volume	1810.7(3) $Å^3$	
Z	4	
Density (calculated)	1.413 Mg/m^3	
Absorption coefficient	2.572 mm^{-1}	
F(000)	792	
Crystal size	0.245 x 0.071 x 0.024 mm^3	
Theta range for data collection	2.782 to 25.250°.	
Index ranges	–14<=h<=14, –22<=k<=21, –9<=l<=9	
Reflections collected	27875	
Independent reflections	3276 [R(int) = 0.1767]	
Completeness to theta = 25.242°	99.8 %	
Absorption correction	Semi-empirical from equivalents	
Max. and min. transmission	0.7454 and 0.6321	
Refinement method	Full-matrix least-squares on F^2	
Data / restraints / parameters	3276 / 54 / 203	
Goodness-of-fit on F^2	1.248	
Final R indices [I>2sigma(I)]	R1 = 0.1415, wR2 = 0.2273	
R indices (all data)	R1 = 0.1751, wR2 = 0.2406	
Largest diff. peak and hole	1.052 and –2.619 e·$Å^{-3}$	

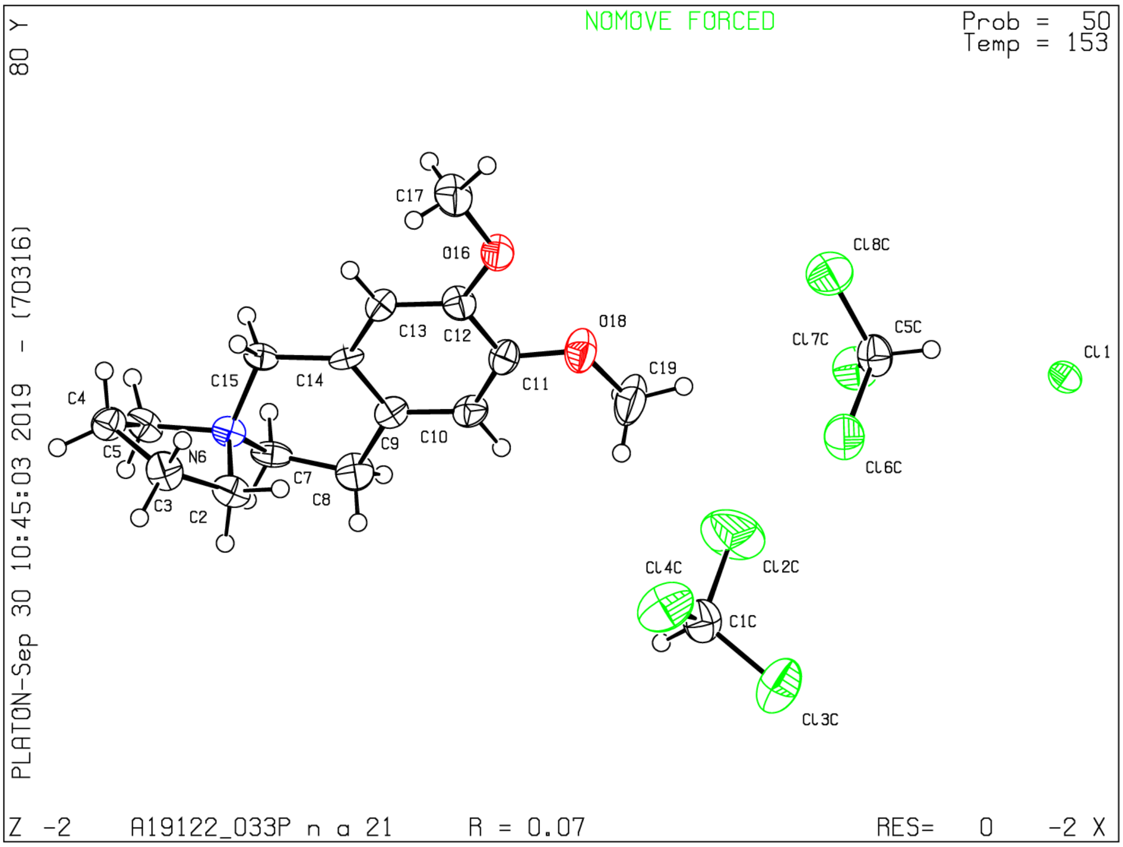

Supplementary Figure 101. Crystallographic data of **17**.

Response to PLAT340_Alert_3_B in checkCIF report: The low data quality is due to the instability of the analysed crystal. This is the best data obtained after our much efforts, for example by directly coating the crystal and quickly mounting it on diffractometer.

Crystal data and structure refinement for **17** (CCDC 1977903).

Empirical formula	$C_{17}H_{24}NO_2Cl_7$	
Formula weight	522.52	
Temperature	153(2) K	
Wavelength	0.71073 Å	
Crystal system	Orthorhombic	
Space group	$Pna2_1$	
Unit cell dimensions	a = 12.1703(12) Å	α = 90°
	b = 6.4920(7) Å	β = 90°
	c = 30.032(3) Å	γ = 90°
Volume	2372.8(4) $Å^3$	
Z	4	
Density (calculated)	1.463 Mg/m^3	
Absorption coefficient	0.850 mm^{-1}	
F(000)	1072	
Crystal size	0.321 x 0.247 x 0.087 mm^3	
Theta range for data collection	3.210 to 27.520°.	
Index ranges	–15<=h<=15, –8<=k<=7, –30<=l<=38	
Reflections collected	13108	
Independent reflections	4775 [R(int) = 0.0473]	
Completeness to theta = 25.242°	99.2 %	
Absorption correction	Semi-empirical from equivalents	
Max. and min. transmission	0.7456 and 0.5145	
Refinement method	Full-matrix least-squares on F^2	
Data / restraints / parameters	4775 / 1 / 246	
Goodness-of-fit on F^2	1.175	
Final R indices [I>2sigma(I)]	R1 = 0.0670, wR2 = 0.1504	
R indices (all data)	R1 = 0.0712, wR2 = 0.1521	
Absolute structure parameter	0.12(4)	
Largest diff. peak and hole	0.385 and –0.610 e·$Å^{-3}$	

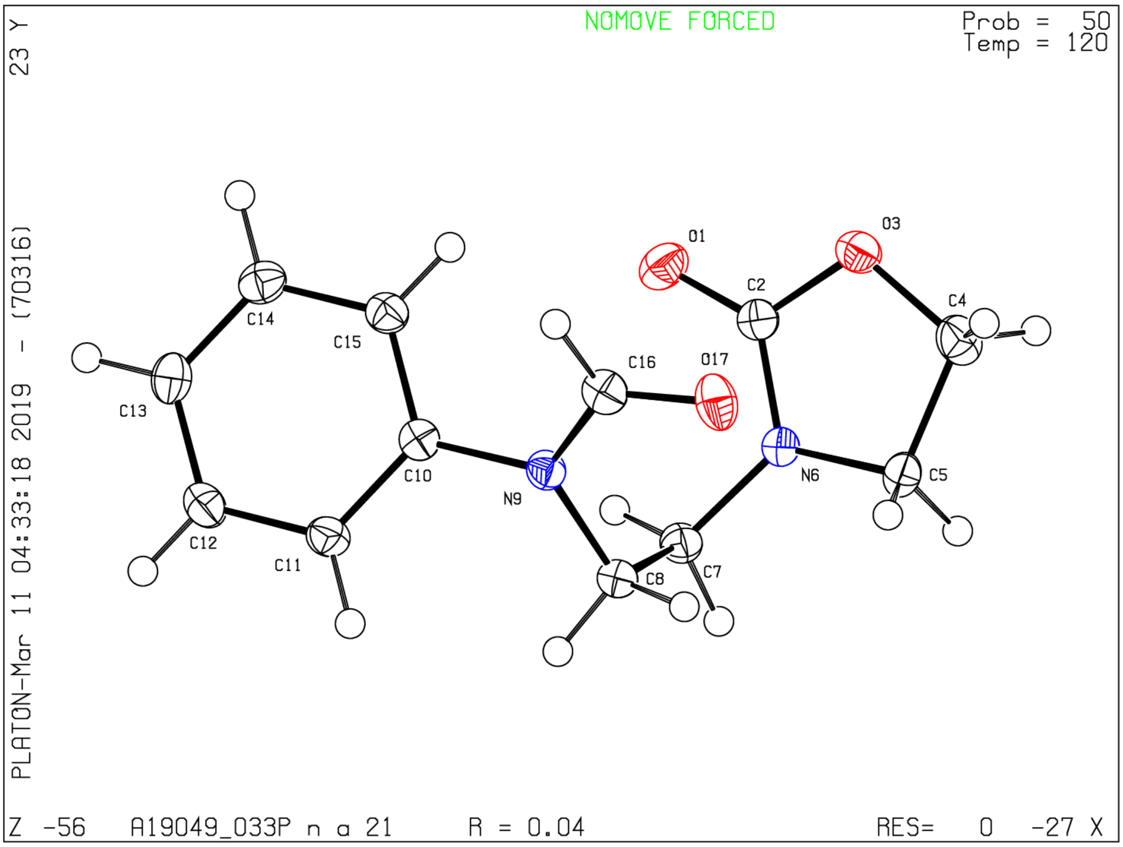

Supplementary Figure 102. Crystallographic data of **23**.

Crystal data and structure refinement for **23** (CCDC 1977902).

Empirical formula	$C_{12}H_{14}N_2O_3$	
Formula weight	234.25	
Temperature	120(2) K	
Wavelength	0.71073 Å	
Crystal system	Orthorhombic	
Space group	$Pna2_1$	
Unit cell dimensions	a = 14.3513(10) Å	α = 90°
	b = 8.3278(6) Å	β = 90°
	c = 9.1845(6) Å	γ = 90°
Volume	1097.68(13) $Å^3$	
Z	4	
Density (calculated)	1.417 Mg/m^3	
Absorption coefficient	0.103 mm^{-1}	
F(000)	496	
Crystal size	0.128 x 0.096 x 0.087 mm^3	
Theta range for data collection	2.828 to 27.999°.	
Index ranges	–17<=h<=18, –8<=k<=10, –12<=l<=12	
Reflections collected	8732	
Independent reflections	2594 [R(int) = 0.0479]	
Completeness to theta = 25.242°	100.0 %	
Absorption correction	Semi-empirical from equivalents	
Max. and min. transmission	0.7457 and 0.6635	
Refinement method	Full-matrix least-squares on F^2	
Data / restraints / parameters	2594 / 1 / 154	
Goodness-of-fit on F^2	1.002	
Final R indices [I>2sigma(I)]	R1 = 0.0371, wR2 = 0.0729	
R indices (all data)	R1 = 0.0515, wR2 = 0.0776	
Largest diff. peak and hole	0.158 and –0.191 $e·Å^{-3}$	

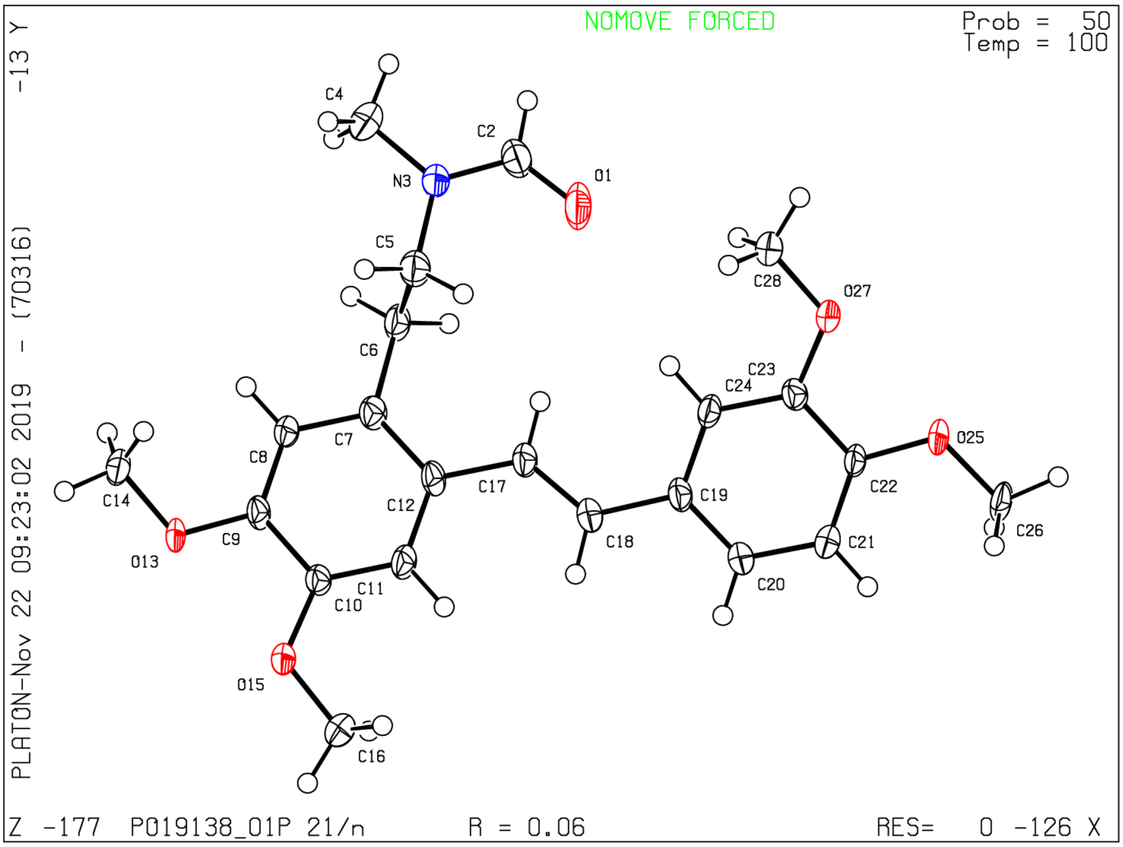

Supplementary Figure 103. Crystallographic data of **24**.

Crystal data and structure refinement for **24** (CCDC 1977905).

Empirical formula	$C_{22}H_{27}NO_5$	
Formula weight	385.44	
Temperature	100(2) K	
Wavelength	0.800 Å	
Crystal system	Monoclinic	
Space group	$P2_1/n$	
Unit cell dimensions	a = 13.999(3) Å	α = 90°
	b = 9.1720(18) Å	β = 102.19(3)°
	c = 15.748(3) Å	γ = 90°
Volume	1976.4(7) $Å^3$	
Z	4	
Density (calculated)	1.295 Mg/m^3	
Absorption coefficient	0.118 mm^{-1}	
F(000)	824	
Crystal size	0.038 x 0.016 x 0.015 mm^3	
Theta range for data collection	1.993 to 29.996°.	
Index ranges	–17<=h<=17, –11<=k<=11, –19<=l<=19	
Reflections collected	13469	
Independent reflections	3971 [R(int) = 0.1224]	
Completeness to theta = 28.685°	99.6 %	
Absorption correction	Empirical	
Max. and min. transmission	1.000 and 0.970	
Refinement method	Full-matrix least-squares on F^2	
Data / restraints / parameters	3971 / 0 / 258	
Goodness-of-fit on F^2	0.952	
Final R indices [I>2sigma(I)]	R1 = 0.0552, wR2 = 0.1200	
R indices (all data)	R1 = 0.1274, wR2 = 0.1454	
Largest diff. peak and hole	0.405 and –0.267 e·$Å^{-3}$	

Cartesian Coordinates of DFT-Optimized Structures

int1

Charge: 0, Multiplicity: 1

6	-2.617582000	0.177813000	0.164910000
7	-1.137000000	0.357820000	-0.177990000
6	-0.678901000	-0.957100000	-0.829226000
6	-0.330229000	0.588234000	1.071577000
6	-0.974120000	1.540854000	-1.116167000
1	-2.696583000	0.068404000	1.246318000
1	-3.150875000	1.077430000	-0.140892000
6	-3.039580000	-1.098302000	-0.554968000
1	-3.306755000	-0.884813000	-1.595762000
1	-3.904329000	-1.553989000	-0.068825000
6	-1.774511000	-1.954945000	-0.492147000
1	-1.627586000	-2.363250000	0.511606000
1	-1.777683000	-2.782546000	-1.203676000
1	-0.640196000	-0.760470000	-1.903411000
1	0.335593000	-1.189597000	-0.482099000
1	0.749628000	0.602153000	0.846944000
9	-0.615254000	-0.419967000	1.911484000
9	-0.794027000	1.729745000	1.620824000
1	-1.600937000	1.289464000	-1.976923000
1	-1.419505000	2.393767000	-0.599981000
6	0.455366000	1.834141000	-1.530525000
1	0.415102000	2.614020000	-2.295904000
1	0.972864000	0.962317000	-1.935717000
1	1.063293000	2.200814000	-0.701598000
35	2.703989000	-0.463201000	0.003360000

TS1-int1

Charge: 0, Multiplicity: 1

6	-2.255655000	-0.377752000	1.096095000
6	-2.837189000	-1.550859000	0.314761000
7	-1.494327000	0.420433000	0.096374000
6	-3.174756000	-0.896622000	-1.026684000
6	-1.980376000	0.032305000	-1.259029000
1	-1.593054000	-0.662933000	1.917853000
1	-3.705957000	-1.983728000	0.814812000
1	-4.095835000	-0.309869000	-0.936025000
1	-2.087376000	-2.335429000	0.183815000
1	-3.306803000	-1.610464000	-1.841980000
1	-2.236329000	0.923051000	-1.838110000
1	-1.174562000	-0.492935000	-1.786807000
1	-3.063650000	0.244805000	1.503624000
6	0.426087000	-0.084493000	0.190070000
9	0.579304000	0.463741000	1.370855000
9	0.178302000	-1.370809000	0.229235000
1	0.583992000	0.433379000	-0.738507000
35	2.891904000	-0.328425000	-0.198357000
6	-1.585000000	1.870114000	0.372684000
1	-2.618339000	2.190765000	0.172135000
1	-1.401253000	2.002551000	1.442707000
6	-0.600441000	2.715629000	-0.426956000
1	-0.682880000	2.535786000	-1.503492000
1	0.434795000	2.541633000	-0.120497000
1	-0.817809000	3.772205000	-0.254446000

TS2-int1

Charge: 0, Multiplicity: 1

6	1.544302000	0.958527000	-1.087106000
6	0.861449000	2.265427000	-0.689146000
7	1.416806000	0.127820000	0.142045000
6	1.152219000	2.408956000	0.824794000
6	1.712162000	1.043288000	1.274136000
1	2.608153000	1.108891000	-1.302027000
1	-0.213827000	2.203644000	-0.876287000
1	0.245017000	2.665041000	1.375649000
1	1.251776000	3.102782000	-1.269485000
1	1.885618000	3.192718000	1.022250000
1	1.244314000	0.656338000	2.183766000
1	2.795447000	1.088517000	1.427494000
1	1.078134000	0.445476000	-1.929682000
6	2.239481000	-1.051378000	0.125803000
9	1.860097000	-1.822395000	-0.913579000
9	3.547897000	-0.729985000	-0.073339000
1	2.160649000	-1.613843000	1.057796000
35	-3.076514000	-0.190879000	-0.205835000
6	-0.480791000	-0.355728000	0.252812000
1	-0.744366000	0.626583000	0.612589000
1	-0.542633000	-0.537406000	-0.809234000
6	-0.476991000	-1.504335000	1.224490000
1	0.145510000	-1.284379000	2.099594000
1	-0.135722000	-2.426968000	0.749905000
1	-1.495195000	-1.660510000	1.578278000

TS3-int1

Charge: 0, Multiplicity: 1

6	0.452821000	0.154219000	-0.054635000
6	0.601216000	1.544025000	0.518050000
7	-1.465122000	0.165670000	-0.094511000
6	-0.556390000	2.389043000	-0.014162000
6	-1.814025000	1.572618000	0.251458000
1	0.578515000	-0.730422000	0.552519000
1	1.566062000	1.947847000	0.216026000
1	-0.430842000	2.559384000	-1.090244000
1	0.584335000	1.494758000	1.611512000
1	-0.615475000	3.367537000	0.468322000
1	-2.685115000	1.896311000	-0.326778000
1	-2.065909000	1.612662000	1.314446000
1	0.573458000	0.017522000	-1.121815000

6	-1.965118000	-0.769060000	0.897415000
9	-1.628281000	-2.016679000	0.519190000
9	-1.364318000	-0.505919000	2.076287000
1	-3.050400000	-0.717172000	1.040939000
35	3.077658000	-0.329161000	-0.266935000
6	-1.866682000	-0.219352000	-1.475565000
1	-1.534829000	0.602382000	-2.116217000
1	-1.283883000	-1.102791000	-1.745633000
6	-3.357013000	-0.485368000	-1.657623000
1	-3.973472000	0.352357000	-1.318100000
1	-3.673071000	-1.391926000	-1.134978000
1	-3.560868000	-0.635472000	-2.720112000

***N*-Ethylpyrrolidine, 1b**

Charge: 0, Multiplicity: 1

6	-0.867715000	-1.137427000	-0.512666000
6	-1.792666000	-0.474005000	0.534519000
7	0.306611000	-0.272595000	-0.640095000
6	-1.443708000	1.028506000	0.420160000
6	-0.291639000	1.066294000	-0.619500000
1	-1.372216000	-1.170089000	-1.485792000
1	-1.568748000	-0.850175000	1.536710000
1	-1.117628000	1.429726000	1.383251000
1	-2.847405000	-0.679753000	0.338469000
1	-2.294223000	1.631112000	0.093465000
1	0.459343000	1.830188000	-0.405450000
1	-0.695341000	1.273412000	-1.616920000
1	-0.572341000	-2.158078000	-0.256194000
6	1.256713000	-0.468445000	0.457750000
1	0.868610000	-0.102317000	1.426350000
1	1.406580000	-1.547696000	0.570824000
6	2.599608000	0.185397000	0.160666000
1	2.511210000	1.271827000	0.073620000
1	2.996147000	-0.197073000	-0.782400000
1	3.316172000	-0.024842000	0.959160000

CF_2HBr

Charge: 0, Multiplicity: 1

6	-0.437830000	-0.931928000	0.000036000
35	0.081579000	0.971941000	-0.000017000
1	-1.525067000	-0.998220000	-0.000019000
9	0.071021000	-1.526433000	1.084246000
9	0.070971000	-1.526430000	-1.084196000

***N*-Difluoromethylpyrrolidine, 1-CF_2H**

Charge: 0, Multiplicity: 1

7	0.079170000	0.051648000	-0.600662000
6	-0.484270000	1.169162000	0.187310000
6	-0.888535000	-1.069308000	-0.654479000
6	1.385474000	-0.311755000	-0.273121000
1	-0.098340000	1.157018000	1.215944000
1	-0.222458000	2.123729000	-0.273914000
6	-1.984981000	0.880585000	0.177790000
6	-2.020660000	-0.646199000	0.287068000
1	-1.251623000	-1.184400000	-1.683701000
1	-0.423346000	-2.010633000	-0.344523000
1	1.780799000	-1.100773000	-0.919209000
9	1.511371000	-0.776321000	1.035716000
9	2.197316000	0.776807000	-0.335038000
1	-2.427094000	1.203087000	-0.771168000
1	-2.512451000	1.387101000	0.991372000
1	-2.983863000	-1.086293000	0.010159000
1	-1.784866000	-0.951732000	1.315005000

Ethyl Bromide

Charge: 0, Multiplicity: 1

35	-0.001399000	0.802147000	-0.000006000
6	-0.600907000	-1.103974000	0.000018000
1	-1.21884000	-1.209442000	0.890236000
1	-1.218876000	-1.209455000	-0.890189000
6	0.581177000	-2.047948000	0.000015000
1	1.202474000	-1.901627000	0.886419000
1	1.202490000	-1.901600000	-0.886375000
1	0.214484000	-3.079961000	-0.000013000

RO1

Charge: 0, Multiplicity: 1

6	-2.184447000	-0.280761000	1.098335000
6	-0.865457000	-0.593623000	0.428356000
7	2.070553000	0.135064000	0.301270000
6	-0.271683000	0.566640000	-0.363808000
6	1.150242000	0.265843000	-0.831773000
1	-2.126958000	0.604164000	1.733834000
1	-0.974967000	-1.474039000	-0.216320000
1	-0.274240000	1.473153000	0.255591000
1	-0.164342000	-0.872848000	1.226589000
1	-0.897001000	0.784181000	-1.236760000
1	1.500172000	1.044284000	-1.522790000
1	1.159113000	-0.675849000	-1.388652000
1	-2.569506000	-1.123670000	1.668852000
35	-3.632724000	0.134960000	-0.224257000
6	2.930844000	-0.959242000	0.256762000
1	3.628441000	-0.961044000	1.098948000
9	2.221558000	-2.124402000	0.256251000
9	3.686081000	-1.024650000	-0.902960000
6	2.657159000	1.375970000	0.803109000
1	1.836076000	2.091821000	0.909796000
1	3.032699000	1.185844000	1.814383000
6	3.768224000	1.973396000	-0.062270000
1	3.434164000	2.134049000	-1.091192000
1	4.642673000	1.316577000	-0.095785000

1 4.081675000 2.939403000 0.346152000

2b

Charge: 0, Multiplicity: 1

6 1.815387000 0.462449000 1.045954000
6 0.518856000 0.682646000 0.297051000
7 -2.482855000 0.104674000 0.006660000
6 -0.104918000 -0.599985000 -0.245399000
6 -1.455490000 -0.359076000 -0.920109000
1 1.716911000 -0.273378000 1.844786000
1 0.666468000 1.403837000 -0.513921000
1 -0.221663000 -1.328946000 0.569098000
1 -0.173215000 1.158364000 1.005614000
1 0.570198000 -1.055140000 -0.978498000
1 -1.810569000 -1.284109000 -1.386626000
1 -1.354773000 0.399296000 -1.701605000
1 2.227516000 1.389178000 1.440946000
35 3.262985000 -0.266084000 -0.139395000
6 -2.749952000 1.431684000 0.107426000
1 -3.568722000 1.647952000 0.818917000
8 -2.165113000 2.313538000 -0.497612000
6 -3.252207000 -0.877855000 0.756419000
1 -2.559825000 -1.628013000 1.155783000
1 -3.696575000 -0.370480000 1.618838000
6 -4.343967000 -1.546459000 -0.075574000
1 -3.923656000 -2.060785000 -0.944231000
1 -5.058963000 -0.801169000 -0.434952000
1 -4.882974000 -2.285128000 0.524346000

***N*-formylpyrrolidine**

Charge: 0, Multiplicity: 1

6 -1.637801000 -0.946151000 -0.180812000
6 -1.889599000 0.476433000 0.336864000
6 -0.128516000 -1.132527000 0.016306000
6 -0.666182000 1.238787000 -0.179580000
7 0.389945000 0.232183000 -0.094388000
1 -1.885555000 -1.001637000 -1.246359000
1 0.115764000 -1.539258000 1.004967000
1 -1.892381000 0.482803000 1.432190000
1 -0.417641000 2.118013000 0.421383000
1 -2.830366000 0.906181000 -0.013380000
1 -0.819080000 1.560319000 -1.218537000
1 0.336297000 -1.779835000 -0.731900000
1 -2.223322000 -1.704859000 0.342100000
6 1.714035000 0.486862000 -0.013239000
8 2.572132000 -0.372093000 0.107557000
1 1.953248000 1.565695000 -0.065934000

H_2O

Charge: 0, Multiplicity: 1

8 0.142617000 -0.936468000 0.185673000
1 0.142617000 -0.168148000 0.765643000
1 0.142617000 -1.704787000 0.765643000

HF

Charge: 0, Multiplicity: 1

9 0.142617000 -0.934751000 0.185221000
1 0.142617000 -0.209482000 0.754524000

int2

Charge: 0, Multiplicity: 1

7 -1.144847000 0.333564000 -0.191459000
6 -0.341006000 0.599287000 1.064488000
6 -0.910318000 1.479551000 -1.164578000
1 0.740032000 0.508115000 0.863042000
9 -0.728145000 -0.294794000 1.985512000
9 -0.707658000 1.822360000 1.500024000
1 -1.394752000 1.180672000 -2.092354000
1 -1.458674000 2.329495000 -0.752064000
6 0.546453000 1.832717000 -1.410420000
1 0.558331000 2.592535000 -2.196556000
1 1.150704000 0.981646000 -1.730364000
1 1.029364000 2.256621000 -0.528059000
35 2.689499000 -0.638671000 0.060796000
6 -2.617655000 0.260194000 0.162164000
6 -0.684192000 -1.008839000 -0.754353000
6 -1.523823000 -1.445459000 -1.944047000
6 -3.013353000 -1.492959000 -1.601620000
6 -3.475344000 -0.152080000 -1.028165000
1 -2.899286000 1.236368000 0.561770000
1 -2.702794000 -0.481975000 0.958103000
1 0.387199000 -0.923158000 -0.964844000
1 -0.788911000 -1.716048000 0.073752000
1 -1.158655000 -2.435952000 -2.230307000
1 -1.343612000 -0.797769000 -2.809368000
1 -3.604747000 -1.750548000 -2.484438000
1 -3.186300000 -2.280554000 -0.856985000
1 -4.506454000 -0.219103000 -0.667618000
1 -3.469816000 0.623828000 -1.801329000

TS1-int2

Charge: 0, Multiplicity: 1

7 -1.470002000 1.340145000 0.992289000
6 -0.282799000 2.228580000 0.971329000
1 0.489160000 1.795790000 0.324355000
1 -0.607275000 3.163303000 0.505168000
6 0.310439000 2.529564000 2.344616000
1 1.203189000 3.143861000 2.205150000
1 0.623122000 1.626557000 2.876010000
1 -0.374193000 3.097045000 2.981011000
6 -2.172488000 1.343945000 -0.309756000
6 -1.174815000 -0.047901000 1.406847000

6	-0.363750000	-0.827980000	0.371027000
6	-1.067642000	-0.808119000	-0.988671000
6	-1.387361000	0.628466000	-1.410486000
1	-2.384060000	2.384836000	-0.570599000
1	-3.130748000	0.835658000	-0.158298000
1	-0.664708000	-0.022632000	2.374273000
1	-2.143105000	-0.539513000	1.561678000
1	-0.237216000	-1.854450000	0.728692000
1	0.643126000	-0.402521000	0.281016000
1	-0.452783000	-1.304337000	-1.745398000
1	-2.004798000	-1.376299000	-0.915023000
1	-1.985661000	0.639156000	-2.326813000
1	-0.461928000	1.172902000	-1.633883000
6	-2.709365000	2.050634000	2.395470000
9	-2.698340000	3.255412000	1.881126000
9	-3.722968000	1.316902000	2.016322000
1	-2.019490000	1.712794000	3.146459000
35	-3.751714000	2.805145000	4.559772000

TS2-int2

Charge: 0, Multiplicity: 1

7	-1.051756000	0.585885000	-0.696928000
6	-0.348067000	0.759084000	0.553108000
6	-0.568324000	2.108858000	-1.850733000
1	0.730406000	0.653583000	0.420612000
9	-0.758792000	-0.155490000	1.473924000
9	-0.631356000	1.977641000	1.054996000
1	-0.964680000	1.633703000	-2.729863000
1	-1.224389000	2.768536000	-1.303517000
6	0.924332000	2.203648000	-1.686172000
1	1.323820000	2.783634000	-2.517010000
1	1.395306000	1.214549000	-1.717904000
1	1.192328000	2.712237000	-0.757422000
35	-0.914210000	4.109818000	-3.548900000
6	-2.527436000	0.689118000	-0.494170000
6	-0.673465000	-0.716985000	-1.307443000
6	-1.409150000	-0.974229000	-2.619982000
6	-2.922650000	-0.857383000	-2.433808000
6	-3.284052000	0.487743000	-1.802767000
1	-2.732364000	1.670409000	-0.061292000
1	-2.824748000	-0.077570000	0.232666000
1	0.410878000	-0.713915000	-1.457832000
1	-0.913526000	-1.512441000	-0.588509000
1	-1.136873000	-1.978746000	-2.957817000
1	-1.065718000	-0.282438000	-3.396034000
1	-3.430407000	-0.978091000	-3.394438000
1	-3.269589000	-1.669754000	-1.780721000
1	-4.353387000	0.530278000	-1.575549000
1	-3.079813000	1.312030000	-2.495737000

TS3-int2

Charge: 0, Multiplicity: 1

7	-1.986319000	0.482222000	-0.688650000
6	-1.491428000	1.067771000	0.541961000
6	-2.394946000	1.552589000	-1.639618000
1	-0.578144000	1.640672000	0.373914000
9	-1.242230000	0.096818000	1.440240000
9	-2.433258000	1.885499000	1.087262000
1	-2.657024000	1.055820000	-2.572640000
1	-3.306405000	2.021609000	-1.247873000
6	-1.331478000	2.609768000	-1.910780000
1	-0.396178000	2.174319000	-2.271152000
1	-1.123952000	3.230929000	-1.036153000
35	1.965709000	-1.641657000	-1.480397000
6	-3.100504000	-0.473286000	-0.428982000
6	-0.434140000	-0.563476000	-1.324096000
6	-1.047750000	-1.185010000	-2.553371000
6	-2.248503000	-2.071699000	-2.213931000
6	-3.446816000	-1.296059000	-1.665657000
1	-3.969897000	0.087504000	-0.063885000
1	-2.767694000	-1.142974000	0.367137000
1	0.210106000	0.298924000	-1.398843000
1	-0.391163000	-1.131200000	-0.404682000
1	-0.269753000	-1.780170000	-3.031166000
1	-1.321990000	-0.402914000	-3.272571000
1	-2.555431000	-2.618256000	-3.110547000
1	-1.927013000	-2.824635000	-1.482579000
1	-4.240698000	-1.990673000	-1.372241000
1	-3.877992000	-0.659795000	-2.447014000
1	-1.705797000	3.273750000	-2.693145000

***N*-Ethylpiperidine, 3b**

Charge: 0, Multiplicity: 1

7	-1.725484000	1.092090000	0.919231000
6	-2.554606000	1.739783000	1.929132000
1	-1.962375000	1.793453000	2.850394000
1	-2.720177000	2.772440000	1.599749000
6	-3.906156000	1.080833000	2.231388000
1	-4.433557000	1.647849000	3.004061000
1	-3.783256000	0.056824000	2.596386000
1	-4.547362000	1.051531000	1.345326000
6	-2.322928000	1.088570000	-0.409808000
6	-1.278016000	-0.241008000	1.300674000
6	-0.207074000	-0.738511000	0.332514000
6	-0.742504000	-0.732734000	-1.100407000
6	-1.296963000	0.648929000	-1.451899000
1	-2.683175000	2.100288000	-0.628618000
1	-3.196946000	0.408148000	-0.463433000
1	-0.879543000	-0.192088000	2.320554000

1	-2.116033000	-0.967190000	1.308087000
1	0.113012000	-1.745438000	0.621826000
1	0.663824000	-0.076230000	0.404503000
1	0.039847000	-1.027633000	-1.807625000
1	-1.548105000	-1.475688000	-1.185813000
1	-1.759859000	0.639034000	-2.444713000
1	-0.480978000	1.381117000	-1.469413000

***N*-Difluoromethylpiperidine, 3-CF_2H**

Charge: 0, Multiplicity: 1

7	-1.215813000	0.137034000	-0.068910000
6	-0.407968000	0.628514000	0.962725000
1	0.652177000	0.589253000	0.701998000
9	-0.558495000	-0.070474000	2.151497000
9	-0.751272000	1.907893000	1.266904000
6	-2.652979000	0.143843000	0.226972000
6	-0.758603000	-1.145841000	-0.604320000
6	-1.488416000	-1.450154000	-1.908910000
6	-3.002827000	-1.438126000	-1.691777000
6	-3.436969000	-0.116621000	-1.055287000
1	-2.909993000	1.115308000	0.653548000
1	-2.892909000	-0.631595000	0.974247000
1	0.321742000	-1.085759000	-0.772932000
1	-0.940069000	-1.957316000	0.122082000
1	-1.158313000	-2.421476000	-2.291356000
1	-1.214638000	-0.688927000	-2.649183000
1	-3.526782000	-1.601373000	-2.638844000
1	-3.278634000	-2.267195000	-1.025516000
1	-4.507802000	-0.129738000	-0.827911000
1	-3.254469000	0.709024000	-1.753240000

RO2

Charge: 0, Multiplicity: 1

7	-2.843549000	0.188326000	0.146304000
6	-3.890989000	-0.697026000	-0.126851000
6	-3.090312000	1.577718000	-0.246490000
1	-4.859253000	-0.306527000	0.187422000
9	-3.674834000	-1.891419000	0.491595000
9	-3.998672000	-1.010635000	-1.473998000
1	-2.200912000	2.139665000	0.047553000
1	-3.173611000	1.661232000	-1.343561000
6	-4.307775000	2.201085000	0.426871000
1	-4.277028000	2.029130000	1.506368000
1	-5.250782000	1.809282000	0.036101000
6	-1.522308000	-0.302781000	-0.264794000
6	3.378291000	-0.689622000	1.017686000
6	2.002896000	-0.168738000	1.378220000
6	0.931727000	-0.447483000	0.326475000
6	-0.452509000	0.015704000	0.773948000
1	-1.249495000	0.126829000	-1.241472000
1	-1.580108000	-1.386952000	-0.399325000
1	3.362179000	-1.741041000	0.731300000
1	4.104525000	-0.531768000	1.815837000
1	2.057108000	0.906404000	1.587989000
1	1.721728000	-0.662409000	2.320566000
1	1.204971000	0.050714000	-0.611540000
1	0.906918000	-1.526976000	0.113878000
1	-0.428764000	1.095596000	0.968482000
1	-0.733026000	-0.469803000	1.715966000
35	4.153876000	0.264765000	-0.565217000
1	-4.309847000	3.279013000	0.248320000

int3

Charge: 0, Multiplicity: 1

7	-1.174156000	0.377897000	-0.068348000
6	-0.195933000	0.451932000	1.084366000
6	-1.083809000	1.670239000	-0.867746000
1	0.842027000	0.404238000	0.715464000
9	-0.450865000	-0.584100000	1.898358000
9	-0.483701000	1.581791000	1.758874000
1	-1.625196000	1.475313000	-1.791952000
1	-1.646052000	2.413258000	-0.297182000
6	0.341153000	2.131261000	-1.149696000
1	1.027519000	1.279966000	-1.235495000
1	0.700538000	2.726838000	-0.301570000
35	2.705799000	-0.533622000	-0.556015000
6	-2.576922000	0.209042000	0.473396000
6	-0.795690000	-0.836862000	-0.909758000
6	-1.802782000	-1.101248000	-2.018315000
6	-3.228334000	-1.228179000	-1.481645000
6	-3.593938000	-0.004563000	-0.640205000
1	-2.792947000	1.092723000	1.076591000
1	-2.550767000	-0.664573000	1.127598000
1	0.226132000	-0.679428000	-1.269317000
1	-0.760502000	-1.674586000	-0.207352000
1	-1.486187000	-2.028402000	-2.504689000
1	-1.746304000	-0.323948000	-2.788357000
1	-3.938598000	-1.350520000	-2.303641000
1	-3.301554000	-2.127300000	-0.856399000
1	-4.568653000	-0.138938000	-0.161729000
1	-3.677907000	0.888726000	-1.268551000
6	0.471115000	2.958067000	-2.442414000
6	1.921845000	3.449463000	-2.504255000
1	2.145785000	4.111971000	-1.661217000
1	2.617418000	2.604735000	-2.462285000
1	2.100684000	4.006374000	-3.430132000
6	-0.484331000	4.156977000	-2.438160000
1	-1.536278000	3.845162000	-2.443786000
1	-0.319650000	4.786555000	-1.556157000

1	-0.325976000	4.777169000	-3.326716000
6	0.202758000	2.070263000	-3.665191000
1	0.409747000	2.622912000	-4.587453000
1	0.846950000	1.183868000	-3.648172000
1	-0.842160000	1.741001000	-3.723539000

TS1- int3

Charge: 0, Multiplicity: 1

6	1.364945000	0.394056000	-0.428357000
1	2.453055000	0.378232000	-0.562177000
1	0.926840000	0.617299000	-1.405406000
6	0.998772000	1.482693000	0.581228000
1	1.665274000	1.413437000	1.451537000
1	-0.023946000	1.347191000	0.955128000
6	1.291153000	-1.955760000	-1.090136000
6	1.280274000	-1.414751000	1.275589000
6	2.780050000	-1.683557000	1.393168000
6	3.215038000	-2.715243000	0.348505000
6	2.788734000	-2.275166000	-1.054600000
1	0.982145000	-1.575578000	-2.068382000
1	0.721669000	-2.869728000	-0.891495000
1	0.946771000	-0.670829000	2.004936000
1	0.722893000	-2.339246000	1.471083000
1	2.997042000	-2.038641000	2.405194000
1	3.339643000	-0.748787000	1.259005000
1	4.296995000	-2.871436000	0.388593000
1	2.743574000	-3.679159000	0.582629000
1	2.989587000	-3.064770000	-1.785309000
1	3.374547000	-1.403640000	-1.368702000
6	1.082970000	2.915956000	0.012498000
6	0.871637000	3.889875000	1.178016000
1	-0.097114000	3.719569000	1.660029000
1	1.655566000	3.775653000	1.935067000
1	0.893297000	4.925307000	0.823005000
6	-0.020855000	3.145386000	-1.028863000
1	0.103990000	2.514657000	-1.914774000
1	-1.010657000	2.939880000	-0.606891000
1	-0.006439000	4.186427000	-1.367917000
6	2.458576000	3.171512000	-0.615645000
1	2.545919000	4.218941000	-0.922125000
1	3.264480000	2.962890000	0.098418000
1	2.621533000	2.556007000	-1.507022000
7	0.883301000	-0.961573000	-0.072719000
9	-1.203593000	-0.347125000	-1.220548000
6	-1.134964000	-0.886633000	-0.031234000
9	-1.280940000	-2.185005000	-0.024214000
1	-1.071542000	-0.325560000	0.880347000
35	-3.531989000	-0.348823000	0.540798000

TS2- int3

Charge: 0, Multiplicity: 1

6	0.364064000	1.136343000	0.600921000
1	0.982512000	1.102350000	-0.300196000
9	0.942528000	0.353928000	1.554204000
9	0.340298000	2.390706000	1.093478000
6	-0.864250000	1.711547000	-2.297061000
1	-1.540587000	1.408041000	-3.102382000
1	-0.218467000	0.839136000	-2.126928000
6	-1.868646000	0.895309000	1.515848000
6	-0.974602000	-0.717747000	-0.115166000
6	-2.376920000	-1.216460000	-0.442569000
6	-3.295437000	-1.054856000	0.770047000
6	-3.288724000	0.392207000	1.265056000
1	-1.870841000	1.960486000	1.754021000
1	-1.418667000	0.353232000	2.358003000
1	-0.306132000	-0.802977000	-0.977339000
1	-0.548154000	-1.321996000	0.696330000
1	-2.297036000	-2.269626000	-0.728414000
1	-2.782266000	-0.684609000	-1.310649000
1	-4.313922000	-1.364152000	0.520686000
1	-2.944532000	-1.717100000	1.573226000
1	-3.832083000	0.473974000	2.211316000
1	-3.808675000	1.047268000	0.557323000
6	0.035108000	2.892252000	-2.797044000
6	1.451980000	2.351612000	-3.041075000
1	1.928811000	2.022411000	-2.108236000
1	1.438227000	1.502268000	-3.734388000
1	2.090207000	3.127824000	-3.475548000
6	0.111184000	4.053477000	-1.800120000
1	-0.874046000	4.505615000	-1.647660000
1	0.516713000	3.741399000	-0.831186000
1	0.778507000	4.825004000	-2.198298000
6	-0.521899000	3.416759000	-4.127961000
1	0.105452000	4.233062000	-4.502858000
1	-0.529259000	2.623241000	-4.884698000
1	-1.542899000	3.784199000	-3.997635000
7	-0.985496000	0.703004000	0.324943000
6	-1.725119000	1.904281000	-1.072873000
1	-1.620262000	2.766848000	-0.428615000
1	-2.659061000	1.378768000	-1.026612000
35	-3.606706000	3.476164000	-2.125824000

TS3- int3

Charge: 0, Multiplicity: 1

7	-2.057864000	0.842596000	-0.571934000
6	-1.743918000	1.467365000	0.697651000
6	-2.351692000	1.882199000	-1.597757000
1	-0.833521000	2.065507000	0.635191000
9	-1.587725000	0.521969000	1.643754000

9	-2.770739000	2.266759000	1.099017000
1	-2.481199000	1.355378000	-2.542262000
1	-3.314783000	2.342400000	-1.342135000
6	-1.272107000	2.953288000	-1.743394000
1	-0.276412000	2.493019000	-1.744245000
1	-1.314224000	3.639025000	-0.887048000
35	1.982290000	-1.271933000	-0.787520000
6	-3.179148000	-0.129725000	-0.439623000
6	-0.418427000	-0.202379000	-0.966761000
6	-0.854529000	-0.842173000	-2.259960000
6	-2.077747000	-1.742844000	-2.071072000
6	-3.348957000	-0.980669000	-1.694221000
1	-4.098899000	0.419129000	-0.201934000
1	-2.940499000	-0.778331000	0.406404000
1	0.224902000	0.662952000	-0.963418000
1	-0.502096000	-0.754976000	-0.041087000
1	-0.013260000	-1.431763000	-2.623770000
1	-1.039620000	-0.068446000	-3.016109000
1	-2.256633000	-2.304789000	-2.992552000
1	-1.843801000	-2.481743000	-1.293431000
1	-4.161439000	-1.686064000	-1.491211000
1	-3.687918000	-0.366390000	-2.536313000
6	-1.399808000	3.794011000	-3.033168000
6	-0.345274000	4.904844000	-2.962524000
1	-0.520649000	5.561109000	-2.103069000
1	0.662520000	4.485319000	-2.870600000
1	-0.372670000	5.520021000	-3.867745000
6	-2.793387000	4.426090000	-3.134116000
1	-3.576013000	3.673260000	-3.278286000
1	-3.032543000	5.000928000	-2.231852000
1	-2.838096000	5.109151000	-3.988518000
6	-1.126892000	2.932589000	-4.273108000
1	-1.129814000	3.556534000	-5.172766000
1	-0.148200000	2.444001000	-4.206913000
1	-1.887951000	2.158363000	-4.419077000

***N*-(3,3-dimethylbutyl)piperidine, 3f**

Charge: 0, Multiplicity: 1

6	0.269582000	0.550153000	0.247213000
1	0.429192000	1.620891000	0.076400000
1	0.376842000	0.384433000	1.336697000
6	1.328536000	-0.231008000	-0.531090000
1	1.254754000	0.075670000	-1.581701000
1	1.096402000	-1.303197000	-0.507430000
6	-2.022490000	1.296133000	0.180872000
6	-1.545392000	-1.044708000	0.344889000
6	-2.916930000	-1.419231000	-0.211865000
6	-3.938689000	-0.329337000	0.110502000
6	-3.417777000	1.023393000	-0.375753000
1	-1.639832000	2.243858000	-0.212144000
1	-2.083494000	1.399057000	1.284940000
1	-0.816713000	-1.820287000	0.088876000
1	-1.600356000	-1.000216000	1.452474000
1	-3.229716000	-2.382654000	0.206895000
1	-2.832958000	-1.540388000	-1.298280000
1	-4.909175000	-0.556918000	-0.339319000
1	-4.090272000	-0.288172000	1.199630000
1	-4.095077000	1.831195000	-0.079185000
1	-3.360107000	1.022692000	-1.470630000
6	2.784688000	-0.054360000	-0.051340000
6	3.700070000	-0.782371000	-1.042813000
1	3.439247000	-1.845258000	-1.108740000
1	3.612495000	-0.351608000	-2.046260000
1	4.748374000	-0.709159000	-0.732143000
6	2.980260000	-0.670291000	1.340307000
1	2.380856000	-0.161250000	2.101599000
1	2.702846000	-1.730096000	1.341995000
1	4.030822000	-0.597877000	1.646443000
6	3.172978000	1.430464000	-0.013901000
1	4.240661000	1.540027000	0.210444000
1	2.978896000	1.911925000	-0.980081000
1	2.617196000	1.975408000	0.756189000
7	-1.089027000	0.236775000	-0.188191000

1-Bromo-3,3-dimethylbutane

Charge: 0, Multiplicity: 1

6	0.504178000	0.533484000	0.363770000
1	0.398621000	0.463854000	1.443954000
1	0.472818000	1.578049000	0.081863000
6	-0.533023000	-0.304876000	-0.384656000
1	-0.341871000	-1.367989000	-0.209419000
1	-0.426959000	-0.139898000	-1.464735000
6	-2.022180000	-0.025791000	-0.013938000
6	-2.888127000	-0.941862000	-0.893778000
1	-2.577187000	-0.896225000	-1.943768000
1	-2.801398000	-1.991282000	-0.581651000
1	-3.947629000	-0.662099000	-0.833134000
6	-2.416247000	1.447469000	-0.367210000
1	-3.070918000	1.900538000	0.406421000
1	-1.531544000	2.118862000	-0.476687000
1	-2.962561000	1.488908000	-1.337606000
6	-2.322116000	-0.416760000	1.478081000
1	-3.211821000	-1.083284000	1.538296000
1	-1.484013000	-0.980391000	1.938661000
1	-2.527194000	0.466353000	2.118677000
35	2.345231000	-0.076124000	-0.050790000

RO3

Charge: 0, Multiplicity: 1

7	1.666691000	1.229023000	0.124471000
6	2.411994000	2.392368000	-0.105471000
6	2.282373000	0.005525000	-0.399288000
1	3.465720000	2.268744000	0.147366000
9	1.901258000	3.430076000	0.613230000
9	2.357798000	2.818683000	-1.425491000
1	1.592879000	-0.806977000	-0.156181000
1	2.350731000	0.047770000	-1.498268000
6	3.650260000	-0.293050000	0.215585000
1	3.615530000	-0.032201000	1.281002000
1	4.415912000	0.342066000	-0.250821000
6	0.245516000	1.355265000	-0.225331000
6	-4.496901000	0.087571000	1.103938000
6	-3.015565000	-0.017601000	1.396125000
6	-2.119337000	0.693610000	0.384676000
6	-0.644761000	0.623702000	0.774711000
1	0.072207000	0.974951000	-1.245400000
1	-0.017891000	2.415899000	-0.233372000
1	-4.821750000	1.119254000	0.961916000
1	-5.104594000	-0.389834000	1.872723000
1	-2.730575000	-1.072682000	1.475180000
1	-2.863349000	0.428618000	2.390585000
1	-2.262906000	0.245977000	-0.606047000
1	-2.430276000	1.745292000	0.301504000
1	-0.339937000	-0.428934000	0.848306000
1	-0.488046000	1.067258000	1.764770000
6	4.113687000	-1.757781000	0.076546000
6	5.558483000	-1.843767000	0.582121000
1	6.224898000	-1.217722000	-0.021223000
1	5.629164000	-1.510334000	1.623483000
1	5.926210000	-2.875681000	0.531626000
6	4.068062000	-2.203909000	-1.391186000
1	4.488371000	-3.209028000	-1.498466000
1	3.043012000	-2.230873000	-1.775234000
1	4.650956000	-1.524516000	-2.025632000
6	3.239615000	-2.690888000	0.924301000
1	3.618642000	-3.718693000	0.871446000
1	3.246668000	-2.384691000	1.977138000
1	2.200366000	-2.711165000	0.582354000
35	-5.002564000	-0.847270000	-0.593598000

***N*-formylpiperidine, 5**

Charge: 0, Multiplicity: 1

7	0.650140000	0.266738000	0.273421000
6	-0.316933000	1.338806000	0.432817000
6	0.175937000	-1.078672000	0.566886000
6	-1.061300000	-1.397373000	-0.272894000
6	-2.133208000	-0.318530000	-0.090054000
6	-1.564690000	1.067737000	-0.408922000
1	0.158995000	2.282308000	0.148553000
1	-0.597185000	1.412589000	1.494566000
1	0.998905000	-1.766054000	0.365751000
1	-0.073366000	-1.137122000	1.637038000
1	-1.450294000	-2.380960000	0.010442000
1	-0.766022000	-1.450966000	-1.328084000
1	-3.001845000	-0.528913000	-0.721958000
1	-2.485686000	-0.332142000	0.951185000
1	-2.310578000	1.847028000	-0.219677000
1	-1.292802000	1.119301000	-1.470772000
6	1.914436000	0.480869000	-0.171017000
8	2.758540000	-0.379961000	-0.344234000
1	2.115126000	1.550432000	-0.368017000

4b

Charge: 0, Multiplicity: 1

7	3.198159000	-0.128558000	-0.329976000
6	3.609993000	-1.487296000	0.008048000
1	2.876285000	-1.898782000	0.714177000
1	3.549682000	-2.095963000	-0.903531000
6	5.003083000	-1.618730000	0.607361000
1	5.096185000	-1.069775000	1.548210000
1	5.778461000	-1.270859000	-0.080280000
6	1.830644000	0.041541000	-0.806643000
6	-2.967298000	1.122217000	0.487816000
6	-1.540363000	0.899175000	0.942595000
6	-0.574912000	0.522030000	-0.177826000
6	0.857764000	0.367193000	0.325604000
1	1.535218000	-0.882929000	-1.316940000
1	1.828743000	0.854492000	-1.538609000
1	-3.032633000	1.817656000	-0.349318000
1	-3.615610000	1.451965000	1.297814000
1	-1.521127000	0.138801000	1.732527000
1	-1.208656000	1.841999000	1.400962000
1	-0.908425000	-0.411683000	-0.647344000
1	-0.606646000	1.296731000	-0.956431000
1	0.898962000	-0.426726000	1.083912000
1	1.194565000	1.294694000	0.803461000
35	-3.816962000	-0.567880000	-0.179123000
1	5.191183000	-2.674552000	0.815546000
6	3.937027000	0.990876000	-0.130747000
8	3.532674000	2.123341000	-0.350798000
1	4.953361000	0.801871000	0.244130000

4f

Charge: 0, Multiplicity: 1

7	1.801212000	1.738285000	-0.346784000
6	2.537657000	0.553912000	-0.780857000

1	1.865316000	-0.033734000	-1.415372000
1	3.376847000	0.895894000	-1.394300000
6	3.057655000	-0.270096000	0.399579000
1	2.212220000	-0.538146000	1.048110000
1	3.718716000	0.379879000	0.985604000
6	0.352289000	1.755122000	-0.431837000
6	-4.052891000	-0.125774000	1.269586000
6	-2.541523000	-0.223628000	1.257725000
6	-1.849942000	0.820850000	0.385648000
6	-0.328222000	0.739619000	0.484765000
1	0.049633000	1.569838000	-1.472359000
1	0.014225000	2.766063000	-0.176214000
1	-4.402590000	0.875687000	1.521369000
1	-4.510228000	-0.857432000	1.933345000
1	-2.241147000	-1.231469000	0.947887000
1	-2.213963000	-0.101029000	2.300405000
1	-2.160022000	0.684304000	-0.657403000
1	-2.186556000	1.823611000	0.684396000
1	-0.000372000	-0.273706000	0.215925000
1	-0.000725000	0.916252000	1.517593000
6	3.818239000	-1.554823000	0.021585000
6	4.336676000	-2.191547000	1.316696000
1	5.023423000	-1.514960000	1.836963000
1	3.510611000	-2.426243000	1.997847000
1	4.873673000	-3.122495000	1.103791000
6	5.012009000	-1.233923000	-0.887253000
1	5.625320000	-2.129053000	-1.039089000
1	4.691617000	-0.885848000	-1.874554000
1	5.644887000	-0.457473000	-0.443235000
6	2.887286000	-2.550578000	-0.682046000
1	3.414408000	-3.489206000	-0.885574000
1	2.017149000	-2.783654000	-0.056536000
1	2.525185000	-2.166329000	-1.641482000
35	-4.844746000	-0.510314000	-0.531285000
6	2.509288000	2.816331000	0.073080000
8	3.726216000	2.863632000	0.133111000
1	1.869149000	3.670419000	0.360464000

Supplementary References

[1] Xu, G.-Q., Xu, J.-T., Feng, Z.-T., Liang, H., Wang, Z.-Y., Qin, Y. & Xu, P.-F. Dual C(sp^3)−H bond functionalization of N-heterocycles through sequential visible-light photocatalyzed dehydrogenation/[2+2] cycloaddition reactions. *Angew. Chem. Int. Ed.* **57**, 5110-5114 (2018).

[2] De Souza, T. M., Bieber, L. W., Longo, R. L. & Malvestiti, I. Microwave-assisted synthesis of N-substituted-2,5-dihydro-1H-pyrroles and N-substituted-1H-pyrroles in water. *ChemistrySelect* **3**, 34-39 (2018).

[3] Orejarena Pacheco, J. C., Lipp, A., Nauth, A. M., Acke, F., Dietz, J.-P. & Opatz, T. A highly active system for the metal-free aerobic photocyanation of tertiary amines with visible light: application to the synthesis of Tetraponerines and Crispine A. *Chem. Eur. J.* **22**, 5409-5415 (2016).

[4] Kamal, A., Reddy, J. S., Bharathi, E. V. & Dastagiri, D. Base-free monosulfonylation of amines using tosyl or mesyl chloride in water. *Tetrahedron Lett.* **49**, 348-353 (2008).

[5] Tanoue, A., Yoo, W.-J. & Kobayashi, S. Sulfuryl chloride as an efficient initiator for the metal-free aerobic cross-dehydrogenative coupling reaction of tertiary amines. *Org. Lett.* **16**, 2346-2349 (2014).

[6] Nauth, A. M., Otto, N. & Opatz, T. α-Cyanation of aromatic tertiary amines using ferricyanide as a non-toxic cyanide source. *Adv. Synth. Catal.* **357**, 3424-3428 (2015).

[7] Flores Toque, H. A., Priviero, F. B. M., Teixeira, C. E., Perissutti, E., Fiorino, F., Severino, B., Frecentese, F., Lorenzetti, R., Baracat, J. S., Santagada, V., Caliendo, G., Antunes, E. & De Nucci, G. Synthesis and pharmacological evaluations of Sildenafil analogues for treatment of erectile dysfunction. *J. Med. Chem.* **51**, 2807-2815 (2008).

[8] Wang, W., Ding, C., Li, Y., Li, Z., Li, Y., Peng, L. & Yin, G. Migratory arylboration of unactivated alkenes enabled by nickel catalysis. *Angew. Chem. Int. Ed.* **58**, 4612-4616 (2019).

[9] Wang, F., Li, L., Ni, C. & Hu, J. Deoxygenative gem-difluoroolefination of carbonyl compounds with (chlorodifluoromethyl)trimethylsilane and triphenylphosphine. *Beilstein J. Org. Chem.* **10**, 344-351 (2014).

[10] Kosobokov, M. D., Levin, V. V., Struchkova, M. I. & Dilman, A. D. Nucleophilic bromo- and iododifluoromethylation of aldehydes. *Org. Lett.* **16**, 3784-3787 (2014).

[11] Mujahidin, D. & Doye, S. Enantioselective synthesis of (+)-(*S*)-Laudanosine and (–)-(*S*)-Xylopinine. *Eur. J. Org. Chem.* **2005**, 2689-2693 (2005).

[12] Zhang, H., Cai, Q. & Ma, D. Amino acid promoted CuI-catalyzed C−N bond formation between aryl halides and amines or N-containing heterocycles. *J. Org. Chem.* **70**, 5164-5173 (2005).

[13] Xia, Q., Tian, H., Dong, J., Qu, Y., Li, L., Song, H., Liu, Y. & Wang, Q. N-Arylamines coupled with aldehydes, ketones, and imines by means of photocatalytic proton-coupled electron transfer. *Chem. Eur. J.* **24**, 9269-9273 (2018).

[14] Fountoulaki, S., Daikopoulou, V., Gkizis, P. L., Tamiolakis, I., Armatas, G. S. & Lykakis, I. N. Mechanistic studies of the reduction of nitroarenes by $NaBH_4$ or hydrosilanes catalyzed by supported gold nanoparticles. *ACS Catal.* **4**, 3504-3511 (2014).

[15] Huang, P., Wang, Y.-X., Yu, H.-F. & Lu, J.-M. N-Heterocyclic carbene–palladium(II)–4,5-dihydrooxazole complexes: synthesis and catalytic activity toward amination of aryl chlorides.

Organometallics **33**, 1587-1593 (2014).

[16] Jette, C. I., Geibel, I., Bachman, S., Hayashi, M., Sakurai, S., Shimizu, H., Morgan, J. B. & Stoltz, B. M. Palladium-catalyzed construction of quaternary stereocenters by enantioselective arylation of γ-lactams with aryl chlorides and bromides. *Angew. Chem. Int. Ed.* **58**, 4297-4301 (2019).

[17] Parr, R. G. & Yang, W. *Density Functional Theory of Atoms and Molecules* (Oxford Univ. Press, Oxford, 1989).

[18] Gaussian 09, Revision D01 (Gaussian Inc., Wallingford, CT, 2009).

[19] Zhao, Y. & Truhlar, D. G. The M06 suite of density functionals for main group thermochemistry, thermochemical kinetics, noncovalent interactions, excited states, and transition elements: two new functionals and systematic testing of four M06-class functionals and 12 other functionals and 12 other functionals. *Theor. Chem. Acc.* **120**, 215-241 (2008).

[20] Ditchfield, R., Hehre, W. J. & Pople, J. A. Self-consistent molecular-orbital methods. IX. an extended gaussian-type basis for molecular-orbital studies of organic molecules. *J. Chem. Phys.* **54**, 724-728 (1971).

[21] Hariharan, P. C. & Pople, J. A. The influence of polarization functions on molecular orbital hydrogenation energies. *Theor. Chim. Acta.* **28**, 213-222 (1973).

[22] Hehre, W. J., Ditchfield, R. & Pople, J. A. Self-consistent molecular orbital methods. XII. further extensions of gaussian-type basis sets for use in molecular orbital studies or organic molecules. *J. Chem. Phys.* **56**, 2257-2261 (1972).

[23] Clark, T., Chandrasekhar, J., Spitznagel, G. W. & Schleyer, P. V. R. Efficient diffuse function-augmented basis sets for anion calculations. III. the 3-21+G basis set for first-row elements, Li–F. *J. Comput. Chem.* **4**, 294-301 (1983).

[24] Hay, P. J. & Wadt, W. R. Ab initio effective core potentials for molecular calculations. potentials for the transition metal atoms Sc to Hg. *J. Chem. Phys.* **82**, 270-283 (1985).

[25] Krishnan, R., Binkley, J. S., Seeger, R. & Pople, J. A. Self-consistent molecular orbital methods. XX. a basis set for correlated wave functions. *J. Chem. Phys.* **72**, 650-654 (1980).

[26] Chan, H. S. S., Nguyen, Q. N. N., Paton, R. S. & Burton, J. W. Synthesis, characterization, and reactivity of complex tricyclic oxonium ions, proposed intermediates in natural product biosynthesis. *J. Am. Chem. Soc.* **141**, 15951-15962 (2019).
